# Revision of the Neotropical green lacewing genus *Ungla* (Neuroptera, Chrysopidae)

**DOI:** 10.3897/zookeys.674.11435

**Published:** 2017-05-16

**Authors:** Catherine A. Tauber, Francisco Sosa, Gilberto S. Albuquerque, Maurice J. Tauber

**Affiliations:** 1 Department of Entomology, Comstock Hall, Cornell University, Ithaca, NY 14853-2601 and Department of Entomology, University of California, Davis, CA, USA 95616; 2 Museo Entomológico “Dr. José Manuel Osorio” (MJMO), Universidad Centroccidental “Lisandro Alvarado”, Venezuela; 3 Laboratório de Entomologia e Fitopatologia, CCTA, Universidade Estadual do Norte Fluminense, Campos dos Goytacazes, RJ, Brazil 28013-602

**Keywords:** Chrysopinae, Chrysopini, Neotropics, Nomenclature, Systematics, *Ungla*

## Abstract

Here, *Ungla* Navás, 1914, a poorly known Neotropical genus is reviewed. Twenty-five valid species are recognized; seven of them are new to science: *Ungla adamsi* **sp. n.**, *U. elbergi* **sp. n.**, *U. grandispiracula* **sp. n.**, *U. mexicana* **sp. n.**, *U. pennyi* **sp. n.**, *U. quchapampa* **sp. n.**, *U. stangei* **sp. n.**; and five are transferred to *Ungla* from other genera: *U. bolivari* (Banks), *U. chacranella* (Banks), *U. siderocephala* (Navás), *U. steinbachi* (Navás), and *U. banksi* Tauber, new replacement name. In addition, ten new synonymies are identified. For each species, a full nomenclatural history, diagnosis, description or redescription with images, literature citations, and available information on the distribution and biology are provided. Name-bearing types were examined for each species, and images of most are included. Keys based on external features are provided for species identifications.

As a result of this study, three generalizations appear: (1) The genital morphology of both males and females of *Ungla* species is very conserved. All species express a common structural pattern, the components of which vary only slightly among species. (2) *Ungla* species appear to fall into two geographically distinct groups: about one third (n=7) of the species are recorded from southern South America (specifically Argentina and Brazil) and the other approximately two thirds of the species (n=18) from more northern regions of Neotropical America [Andean and Caribbean regions, Central America, and southern Mexico (Chiapas)]. None of the species from either of the regions is known to overlap into the other region. (3) Available information on the immature stages and natural history of species in *Ungla* is meagre.

## Introduction

The genus *Ungla* Navás. 1914 (Neuroptera: Chrysopidae: Chrysopinae: Chrysopini) is an intriguing and yet neglected group of Neotropical green lacewings. It was described for a single species, *Ungla annulata* Navás, and for ~85 years, the number of species assigned to the genus grew very slowly; by 1998 it included only seven species. All were from southern and western South America (Argentina, Colombia, Peru). Subsequently, two additional *Ungla* species were reported – one from Costa Rica and another from Brazil (Penny 1998, Freitas 2007). And very recently, seven new species were described from Venezuela (Sosa 2015). As a result of this relatively recent work, the genus *Ungla* now ranks as a diverse, broad-ranging, and important component of the Neotropical chrysopid fauna.

Despite the above, the systematics of the genus has been confusing, and attempts to identify specimens to species can be exasperating. A number of factors underlie the difficulties. (1) The original seven *Ungla* species were described on the basis of external features (e.g., coloration, venational differences) that either fade with age and/or that show significant individual variation. (2) Subsequent studies that examined genital characteristics demonstrated very little interspecific variation, especially among females. (3) Many of the type specimens are females and their coloration is faded; thus they were not readily distinguished from other species. (4) The type specimens of currently described *Ungla* species, as well as those of species that should be transferred to *Ungla*, are dispersed among museums in the New and Old Worlds (including South America); they have received little comparative study. Thus, when we initiated this revision we recognized that we were faced with serious constraints and that our goals should be appropriately targeted.

Here, we present an historical account of the genus, and for each named species, we provide images and/or information on the type as well as our evaluation of the species’ taxonomic status. For some species, we were able to borrow or photograph the types and the information is detailed; for others it is less detailed. In addition, for those species that we can identify confidently, and for which we have sufficient material, we offer notes and images of recently collected (non-type) material for use in identifications. To facilitate comparisons, we redescribed all previously described species in the same format as the new species descriptions. This includes species recently described by Sosa (2015), but for these species, reference is made to the earlier work to avoid redundancy. Because female genital characteristics show only subtle interspecific variation, in the interest of time, we did not include them. Finally, the results presented below are largely meant as an aid in identifying species and as a springboard for future studies. The species-delineations are our current interpretations; they are proposed for future studies to challenge and correct.

## Materials and methods

We based this review on four resources: published literature, type specimens, unpublished notes compiled by Phillip A. Adams, and non-type specimens from numerous museums.

### Specimens examined

Most specimens were studied by C. A. Tauber; those examined only by F. Sosa are noted as (FS). In some cases, specimens were also examined or photographed by G. S. Albuquerque (GSA) and/or M. J. Tauber.

We examined (or refer to) specimens from the following museums:

**AMNH** American Museum of Natural History, New York, NY (D. A. Grimaldi, T. Nguyen)

**APTA** Agência Paulista de Tecnologia dos Agronegócios, Ribeirão Preto, SP, Brazil

**BMNH** Natural History Museum (formerly, British Museum of Natural History) London, United Kingdom (D. Goodger, S. Brooks, B. W. Price)

**CAS** California Academy of Sciences, San Francisco, CA (N. D. Penny)

**CUIC** Cornell University Insect Collection, Ithaca, NY (J. K. Liebherr, E. R. Hoebecke, J. Dombroskie)

**DEBU** University of Guelph, ON, Canada (S. A. Marshall)

**EMEC** Essig Museum of Entomology, University of California, Berkeley, CA (C. B. Barr, P. T. Oboyski)

**EMUS** Utah State University Insect Collection, Logan, UT (C. D. von Dohlen)

**FSCA** Florida State Collection of Arthropods, Gainesville, FL (L. A. Stange)

**IFML** Instituto-Fundación Miguel Lillo, San Miguel de Tucumán, Tucumán, Argentina (E. González Olazo, C. Reguilón)

*IIES Museo de Ciencias Naturales (Instituto de Investigaciones Entomológicas de Salta), Salta, Argentina

**INBio** Instituto Nacional de Biodiversidad, Santo Domingo de Heredia, Costa Rica

**MACN** Museo Argentino de Ciencias Naturales, Buenos Aires, Argentina (A. Roig Alsina)

**MCZ** Museum of Comparative Zoology, Harvard University, Cambridge, MA (P. Perkins, S. Cover, B. Farrell)

**MIZA** Museo del Instituto de Zoología Agrícola “Dr. Francisco Fernández Yépez”, Universidad Central de Venezuela, Maracay, Aragua, Venezuela

**MJMO** Museo Entomológico “José Manuel Osorio”, Universidad Centrooccidental “Lisandro Alvarado”, Cabudare, Lara, Venezuela

**MLPA** Museo de la Plata, La Plata, Buenos Aires, Argentina (A. Lanteri)

**MNHN** Muséum national d’histoire naturelle, Paris, France (J. Legrand)

**MSNG** Museo Civico di Storia Naturale “Giacomo Doria”, Genoa, Italy (G. Doria, M. Tavano)

**MZBS** Museu de Ciències Naturals de Barcelona (formerly Museo de Zoología de Barcelona), Spain (V. Monserrat, Universidad Complutense, Madrid)

**MZSP** Museu de Zoologia, Universidade de São Paulo, SP, Brazil (S. Vanin, C. F. Einicker Lamas, C. Campaner)

**SDMC** San Diego Natural History Museum, San Diego, CA (M. Wall, J, Berrian)

**SEMC** Snow Entomological Museum, University of Kansas, Lawrence, KS (Z. H. Falin)

**SFC** Sérgio de Freitas Collection, Faculdade de Ciências Agrárias e Veterinárias, Universidade Estadual Paulista “Júlio de Mesquita Filho”, Jaboticabal, SP, Brazil

**UCDC** Bohart Museum, University of California, Davis, CA (S. L. Heyden, L. S. Kimsey

**UGCA** University of Georgia Collection of Arthropods, Athens, GA (J. V. McHugh, E. R. Hoebecke)

**UMSP** University of Minnesota Insect Collection, Saint Paul, MN (P. J. Clausen, R. E. Thomson)

**USNM** National Museum of Natural History, Washington DC (O. S. Flint, Jr.)

**ZMB** Museum für Naturkunde Berlin, Berlin, Germany (M. Ohl)

*Stange (1967) reported a number of Argentinian types of Neuroptera as being deposited in “San Miguel” (= “Observatorio de Física Cósmica, San Miguel, ARGENTINA”, probably = “Col. Entomológica del Colegio Máximo de San José, San Miguel, provincia de Buenos Aires” of González Olazo 1996). Subsequently, these same specimens were reported to be in the “INESALT” collection (= “Instituto Entomológico de Salta, Rosario de Lerma, provincia de Salta”) (González Olazo 1996). According to González Olazo (1996), the collection in the entomological institute at San Miguel became part of the INESALT in 1979; later, in 1989, Father Gregorio Williner moved the collection (including types) to Buenos Aires, where he resided when González Olazo published his catalog. These specimens then were housed in the Museo de Ciencias Naturales in Salta, Argentina, but are now deposited in the MACN (von Ellenrieder 2009 and personal communication). We were unaware of the location of those types when we visited Argentina, and we have not seen them (listed below under *Chrysopa coronata* Navás and *C. reboredina* Navás).

### Notes of Phillip A. Adams

Phillip A. Adams’ notes consist of sketches and brief descriptive statements regarding the specimens (mostly types) that he examined during his trips to museums in Europe, South America, and North America. These were very valuable in identifying the location and condition of types, and in providing his thoughts on the similarities and differences between selected species. Upon Adams’ death, his notes became the property of the California Academy of Sciences; sections were made available to CAT and MJT by N. D. Penny (CAS).

At the time during which most of the notes were written, Adams considered the species that now are included in *Ungla* to belong in the Old World genus *Suarius*. In all cases, unless specifically noted otherwise, we verified his observations by our own (CAT) examination of the types.

### Lectotype designations

The *Ungla* species that were described before Penny (1998), as well as all of the species later transferred to *Ungla* were described by either L. Navás (Spain) or N. Banks (USA). Each of these authors had a distinct manner of treating types. Navás often described species on the basis of several specimens, and he often labeled several of these specimens as “Typus” without mentioning in the description how many specimens he had or which one he considered to be the primary type. In some cases, subsequent authors (e.g., Stange 1967, González Olazo 1997) considered single known types of Navás as holotypes. However here, consistent with our previous treatments of his types (e.g., Legrand et al. 2008, Tauber et al. 2017), unless Navás specifically mentioned having a single specimen, we assumed that he had others. Thus, to be in accord with Article 74.6 of the International Code of Zoological Nomenclature, rather than identifying holotypes, we designated lectotypes.

Banks, too, often used more than one specimen in his descriptions, but he usually labeled specific specimens as holotypes, or otherwise indicated which specimen he considered to be the primary type. Thus, if we were reasonably certain that his labels indicated his intent to identify a holotype, we considered it as such. If the labeling left us uncertain, we designated a lectotype.

### Measurements, terminology, abbreviations

**Figure 1.**
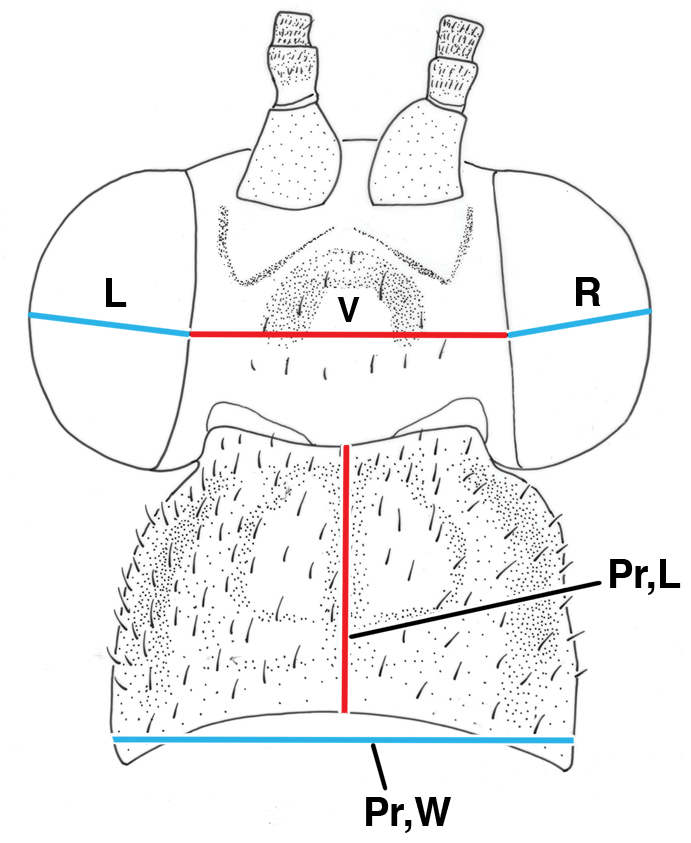
Measurements of head, dorsal. **L** width of left eye (blue) **R** width of right eye (blue) **Pr,L** pronotal length (red) **Pr,W** pronotal width (blue) **V** width of vertex (red).

**Figure 2.**
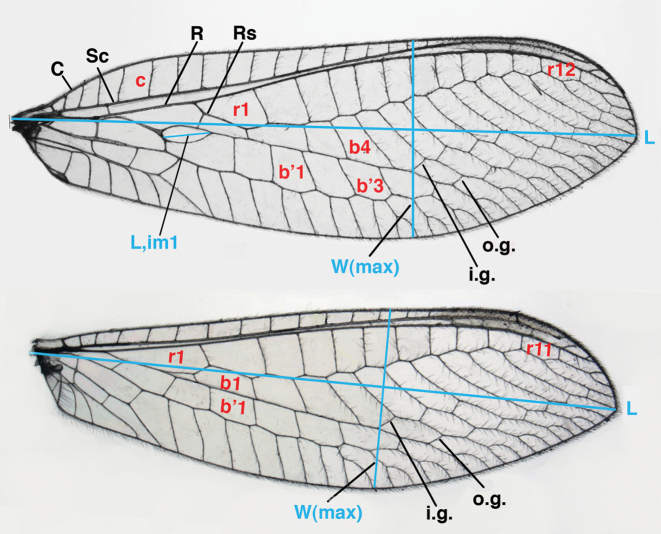
Wing structures and measurements; upper, forewing; lower, hindwing. Veins (black lettering): **C** Costa **Sc** Subcosta **R** Radius **Rs** Radial sector **i.g**. inner gradate **o.g.** outer gradate. Cells (red lettering): **b1, b4** first, fourth upper Banksian cells **b’1, b’3** first, third lower Banksian cells **c** costal cell **r1, r11, r12** first, eleventh, twelfth radial cell. Measurements (blue lines): **L** length of wing **L,im1** length of first intramedian cell **W**(**max**) maximum width of wing.

**Figure 3.**
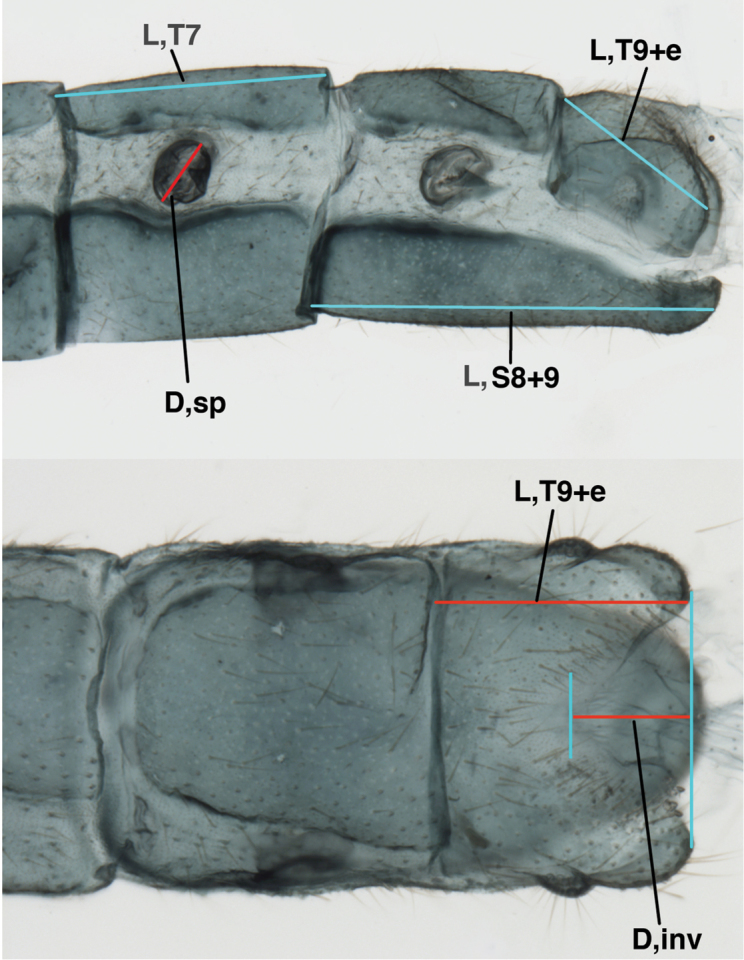
Measurements of male abdomen; upper, lateral; lower, dorsal. **D,inv** depth of invagination **D,sp** maximum diameter of spiracular opening **L,T7** length of seventh tergite **L,T9+e** length of ninth tergite plus ectoproct **L,S8+9** length of fused eighth and ninth sternites.

We made selected measurements on the wing and head. Our techniques are illustrated in Figs 1–3.

In addition to the abbreviations for the museums above, we used several other abbreviations, as follows. For morphological structures: Wing veins (forward to hind) – C = Costa, Sc = Subcosta, R = Radius, Rs = Radial sector, M = Media, Psm = Pseudomedia, Cu = Cubitus, Psc = Pseudocubitus, x = crossvein [e.g., R-Rsx = crossvein between R and Rs; cux1 = first intracubital crossvein]. Cells – b = upper Banksian cell, b’ = lower Banksian cell, im1 = first intramedian cell, m3 = third median cell. Abdominal parts – A = abdominal segment, S = sternite, T = tergite, ect = ectoproct [e.g., S8+9 = fused eighth and ninth sternites, T9+ect = fused ninth tergite and ectoproct].

To categorize literature: biol = biological information; catalog = species included in a catalog; desc = description of adult; dist = distribution records; larval desc = description of larva; list = species name included in a list of species; redesc = redescription of adult; tax = taxonomic information; type(s) = information on type specimen(s).

## The genus *Ungla*

### Current status

**Original description.** *Ungla* Navás, 1914. *Broteria (Zool.)* 12: 224. Type species – *Ungla annulata* Navás, 1914 by original designation and monotypy.

**Taxonomic placement.** Subfamily – Chrysopinae; tribe – Chrysopini. Based on male morphological characteristics, the genus has been suggested as related to *Kostka* and *Chrysopidia* (Brooks 1997: 273). It has not yet been included in published phylogenetic studies using molecular methods.

### Taxonomic history

Originally Navás (1914) established the genus *Ungla* based on a single specimen that had an extremely unusual type of foreleg – one that he had not seen on a chrysopid previously. He stated: “The peculiar form of the anterior leg with long and thin tibial spurs and claws, a thing which is not seen in any other genus of chrysopids; the same with the elongate tarsus, is characteristic of this new genus” (translation by Adams 1975). He described the specimen with the unusual foreleg as *Ungla annulata* Navás, 1914 and designated that species as the type species of the genus.

Navás (1914) did not mention the origin of the genus name “*Ungla*”. Because the unusual leg on the type specimen had long spurs and claws, we suspect that it probably originated from the Catalan word “ungla”, which means nail or claw (information provided by R. Pantaleoni).

*Ungla* persisted as a monotypic genus until the mid-1970s when Adams (1975) demonstrated that the leg that Navás described actually belonged to another insect (an antlion). As a result of his discovery, Adams designated the genus name as invalid; in the same paper he also considered the type species as synonymous with another Argentinian species described earlier (*Hypochrysa argentina* Navás, 1911). He transferred that species to the Old World genus *Suarius* Navás, 1914, and for many years he continued to identify Neotropical species that express similar features as New World representatives of *Suarius*.

Later, Brooks and Barnard (1990) re-evaluated Adams’ generic determinations, and they decided that the New World species were distinct; as a result, they reinstated *Ungla* as a valid genus [for nomenclatural specifics, see Oswald and Penny (1991: 59)]. They described distinctive diagnostic characteristics for the genus and listed four species and one synonym [they missed one synonym previously proposed by Adams (1975)]. During the fifteen years that followed the publication of Brooks and Barnard (1990), three species previously assigned to *Chrysopa* were moved to *Ungla* (González Olazo 1996), and one new species was described (Penny 1998). Thus, when work on this study began (~2006), the genus contained eight recognized species. During the course of the study, Freitas (2007) described a species from Brazil, two species previously assigned to *Chrysopa* were moved to *Ungla* (Legrand et al. 2008), and seven species were described from Venezuela (Sosa 2015). Now, at the conclusion of our study here, the genus contains 25 described species that we consider to be valid biological entities and 15 new synonymies.

### Generic characteristics

Males have distinctive abdominal and genitalic features that differentiate them from males of other chrysopid genera (see below). In contrast, females share a very simple set of reproductive structures that occurs repeatedly among chrysopid genera [*Chrysoperla*, *Pseudomallada*, *Meleoma*, others; see Brooks and Barnard (1990)]. The larvae of two species have been described, but generic level features have not yet been identified.

Externally, *Ungla* adults generally express the following traits: adult body color green to brown, anterior wing length 8-18 mm; head with vertex raised, up-folded posteriorly, surface smooth, without striations, usually with red, brown, or black, inverted U-shaped mark; mark sometimes divided mesally or reduced to a pair of spots; gena usually marked with black, brown, or occasionally red, rarely unmarked; area between antennae sometimes marked; scape broad, strongly curved mesally; palpi flattened apically, usually marked with black, pale at joints; maxillary palpi usually with basal two palpomeres pale, distal three black or marked with black; labial palpi usually with basal palpomere pale, distal two black; cervix usually with dark lateral mark covered by pronotum; pronotum usually with longitudinal, reddish or brownish lateral band, usually bearing long pale setae, sometimes short, black setae; mesonotum, metanotum marked or unmarked; legs usually unmarked, with short pale to black hairs, claws without dilatation at the base. Forewing rounded to acute, without marks but sometimes with brown suffusion around crossveins and gradate veins and in pterostigma, first gradate vein often not meeting Psm, intramedian cell usually ovate, rarely quadrate, with three intracubital cells, distal one open. Hindwing narrow, usually without marks, usually acute, occasionally rounded, with two intracubital cells; venation usually green, occasionally with some veins dark.

*Male abdomen.* Abdominal segments (A1–A8) often with spiracles enlarged (e.g., A7: with largest diameter of spiracle 0.15–0.30× length of segment); left, right sections of ninth tergite and ectoproct (T9+ect) fused dorsally, with broad, often deep, dorsal invagination surrounding large anus; ectoproct and dorsal apodeme greatly shortened, not extending anteriorly below T9, dorsal apodeme and distal ~one-half of lower margin of ectoproct sclerotized, usually terminating in small posterior projection that bends mesally; subanal plate lightly to moderately setose; membrane between subrectal plate and gonarcus short, thus gonarcus closely attached to abdomen. Eighth, ninth sternites fused (S8+9), usually elongate and tapering distally (lateral view); distal margin rounded and concave (posterior view), with low profile; upper margin of S8+9 (lateral view) usually well sclerotized (= ventral apodeme), with terminus usually extended posteriorly at least somewhat beyond T9+ect; terminal setae dense, enlarged, distolateral ones on dorsolateral margin of S8+9 often flanged or winged basally; microtholi absent; patches of microsetae often present.

*Male genitalia.* Tignum, gonapsis, gonocornua, gonocristae, pseudopenis, entoprocessus, parameres absent; gonarcus arched or partially flattened transversely (arch often becoming expanded laterally when gonarcus is excised from abdominal cavity), usually with elongate sclerotized process extending forward from gonarcal apodeme; mediuncus narrow distally, extending forward or downward from dorsum of gonarcal bridge via flexible hinge; gonosaccus large, bilobed, each lobe with patch of long, straight or bent gonosetae arising from large setal bases. Hypandrium internum V- to U-shaped (often difficult to find), with mesal comes.

*Female genitalia.* Spermatheca round, pillbox-shaped, with or without identifiable invagination; velum tall, opening directly to bursa via elongate dorsal slit or opening on the side; bursa unenlarged, simple, without obvious glands; spermathecal duct of moderate length, with two to three curves, distal end with setae-like, filamentous ducts; praegenitale absent; subgenitale with base broad, weakly to well sclerotized, with protrusion short to slightly elongated, bilobed distally, with simple, broad pocket or lobe below, sometimes invaginated; colleterial complex delicate, with elongate gland, small reservoir; transverse sclerite somewhat broad, slightly bowed to flat, with elongate filamentous projections. Adams (1975) reported “slender dorsal bursal glands” on female *Ungla argentina* (as *Suarius*), but in his notes for this species, he states that there are “no bursal glands” and none are shown in his sketch. We have not found any on this species or other *Ungla* species.

### Geographic distribution

*Ungla*, which now contains 25 valid species, is known to occur from southern Mexico (Chiapas) and Central America (Costa Rica) throughout most of South America. It is not yet known from the Caribbean islands or Chile. And, it appears most diverse in northern South America (Venezuela, Ecuador, Peru, Bolivia); however, this pattern may be the result of bias in collecting. For example, the fauna of Argentina and parts of Brazil has not been explored well and we suspect that undescribed *Ungla* species occur there.

From the beginning of the study, we noted that specimens fell into two distinct and non-overlapping groups. Now at the conclusion of the study, we recognize: (1) seven species in southern South America (Argentina: six species; southern Brazil: one species) and (2) 18 species in more northern regions of South America (Peru, Brazil, Bolivia, Ecuador, Colombia, Venezuela: 15 species), and extending into Central America (Costa Rica: 2 species) and southern Mexico (Chiapas: 1 species). The species from the southern region were by far the more problematic, and now that our study is complete, we are unsatisfied with our understanding of this group of species. Clearly, additional specimens and more intense examination of the types from this part of the continent are necessary for a satisfactory classification. In contrast, although the availability of specimens from the more northern regions was relatively scanty, the species were more readily distinguishable on the basis of external characteristics.

For convenience, we divided our treatment of the genus along geographic lines. We begin with the northern group and then deal with the species from Argentina and Brazil separately in the latter part of the revision. We do not expect that the relatively distinct geographic separation that we found among the *Ungla* species we studied will persist when additional specimens become available. Thus, we encourage readers to refer to both sections below when identifying or otherwise studying *Ungla* species.

### Classification of *Ungla* species

**Species from northern South America, Central America, southern Mexico**

*Ungla adamsi* Tauber, **new species**

*Ungla banksi* Tauber, **new replacement name**

(for junior homonym, *Nothochrysa tibialis* Banks, 1914)

*Ungla bolivari* (Banks, 1913), **new combination**

(for *Chrysopa bolivari* Banks)

*Ungla curimaguensis* Sosa, 2015

*Ungla demarmelsi* Sosa, 2015

*Ungla diazi* Sosa, 2015

*Ungla favrei* (Navás, 1935)

=*Chrysopa nesotala* Banks, 1944, **new synonym**

*Ungla grandispiracula* Tauber, **new species**

*Ungla laufferi* (Navás, 1922)

=*Chrysopa aroguesina* Navás, 1929

*Ungla martinsi* Sosa, 2015

=*Ungla rubricosa* Sosa, 2015, **new synonym**

*Ungla mexicana* Tauber, **new species**

*Ungla nigromaculifrons* Sosa, 2015

*Ungla pallescens* Penny, 1998

*Ungla pennyi* Tauber, **new species**

*Ungla quchapampa* Tauber, **new species**

*Ungla siderocephala* (Navás, 1933), **new combination**

(for *Chrysopa siderochephala* Navás)

=*Chrysopa lambda* (Navás, 1933), **new synonym**

*Ungla stangei* Tauber, **new species**

*Ungla yutajensis* Sosa, 2015

**Species from southern South America (Argentina, southern Brazil)**

*Ungla annulata* Navás, 1914

=*Cintameva lurida* Navás, 1930, **new synonym**

*Ungla argentina* (Navás, 1911)

*Ungla chacranella* (Banks, 1915), **new combination**

(for *Chrysopa chacranella* Banks)

=*Chrysopa mendocensis* Navás, 1918, **new synonym**

=*Chrysopa plesia* Navás, 1918, **new synonym**

=*Chrysopa metanotalis* Navás, 1924, **new synonym**

=*Chrysopa villica* Navás, 1929, **new synonym**

*Ungla confraterna* (Banks, 1913)

=*Chrysopa scalai* Navás, 1917, **new synonym**

=*Chrysopa binaria* Navás, 1923, **new synonym**

*Ungla elbergi* Tauber, **new species**

*Ungla ivancruzi* Freitas, 2007

*Ungla steinbachi* Navás, 1925, **new combination**

(for *Chrysopa steinbachi* Navás)

***Species inquirendae***

*Chrysopa venulosa* Navás, 1918

*Chrysopa graciana* Navás, 1919

*Chrysopa nervulosa* Navás, 1924

*Chrysopa coronata* Navás, 1930

***Species incertae sedis* (probably *Ungla*)**

*Chrysopa dichroa* Navás, 1923

*Chrysopa reboredina* Navás, 1933

## Systematic treatment of species

### Part 1. *Ungla* species from southern Mexico, Central America, and northern South America (Bolivia and northward)

**Key to adults**

**Table:** 

1	Frons with dark (brown, red or black) mesal spot (Figs 4a, b, 18f, 38b, c, 64a, 77a, 88b)	**2**
1’	Frons without mesal spot	**7**
2(1)	Antenna with flagellum black at least basally or with base black (Figs 18, 78)	**3**
2’	Antenna with flagellum cream-colored throughout	**4**
3(2)	Frons with prominent mesal protuberance below frontal spot (at least in male); forewing ~ 10 mm long, with ~ 3–4 gradate veins per series (Fig. 17)	***U. bolivari* (Banks)**
3’	Frons without protuberance; forewing ~13–14 mm long, with ~ 5–7 gradate veins per series (Figs 77, 78)	***U. quchapampa* Tauber, sp. n.**
4(2)	Forewing long: ~17–18 mm long, with 8–11 gradate veins per series (Fig. 89); U-shaped mark on vertex reduced to pair of crescent-shaped anterior spots (Fig. 88)	***U. stangei* Tauber, sp .n.**
4’	Forewing shorter: ~11–15.5 mm long, with 4–8 gradate veins per series; U-shaped mark on vertex separated mesally, but arms visible	**5**
5(4)	Hindwing with gradate veins dark (brown or black) (Fig. 65); male with frons not swollen (Fig. 64)	***U. nigromaculifrons* Sosa**
5’	Hindwing with gradate veins pale (green) (Figs 6, 35i); male with frons swollen (Figs 4a, 38a)	**6**
6(5)	Dorsal surface of scape with brown distal stripe (Fig. 38d); if frontal spot extended transversely, then in a triangular shape (Fig. 38c)	***U. favrei* (Navás)**
6’	Dorsal surface of scape tinged with red, but without distinct stripe (Fig. 4e, f); female with frontal marking extended transversely into an inverted V-shape (Fig. 4b)	***U. adamsi* Tauber, sp. n.**
7(1)	Flagellum black or partially black	**8**
7’	Flagellum cream-colored, unmarked	**9**
8(7)	Scape with dorsal surface unmarked, ventral surface with large black mark distally; foretibia without black spot (Fig. 31)	***U. diazi* Sosa**
8’	Scape with dorsal surface almost entirely black, ventral surface unmarked (Fig. 11); foretibia with small black frontal mark (Fig. 16)	***U. banksi* Tauber, new replacement name**
9(7)	Prothorax with lateral stripe red, extending only along anterior half of segment (Fig. 61b, c)	***U. mexicana* Tauber, sp. n.**
9’	Prothorax with lateral stripe red to brown, extending full length of segment	**10**
10(9)	Dorsum of scape with distinct reddish, brown, or black stripe or mark	**11**
10’	Dorsum of scape cream-colored or slightly tinged with red, without marks	**17**
11(10)	Mark on vertex reduced to pair of small or diffuse red marks	**12**
11’	Mark on vertex distinct, robust, with crescent-shaped arms at least anteriorly, either diffuse or heavy posteriorly	**14**
12(11)	Forewing venation (males) robust, green except gradate veins dark, with at least some brown suffusion on surrounding membrane (Fig. 22b); dorsum of head sometimes with prominent red mark between eyes and posterolateral margin of vertex (Fig. 21b)	**13**
12’	Forewing with venation thin, delicate; longitudinal veins mostly green; most crossveins, including gradates reddish, without suffusion (Fig. 27b); dorsum of head without prominent mark between eyes and lateral margin of vertex (Fig. 26)	***U. demarmelsi* Sosa**
13(12)	Gena and palpi light or with no marks; region between lateral margin of eyes and vertex marked with red (Fig. 21)	***U. curimaguensis* Sosa**
13’	Gena and palpi heavily marked with black or reddish black; region between lateral margin of eyes and vertex cream-colored, unmarked (Figs 43, 44)	***U. grandispiracula* Tauber, sp. n.**
14(11)	Forewing with crossveins mostly brown or black, some with suffusion on surrounding membrane; dorsum of head with prominent reddish brown mark between eyes and margin of raised vertex	**15**
14’	Forewing with crossveins mostly reddish, none with suffusion; area between eyes and lateral margin of vertex without prominent mark	**16**
15 (14)	Head cream-colored to yellow; gena, lateral margin of clypeus with black stripe; antenna with dorsal stripe extending full length of scape and onto antennal fossa (Fig. 57)	***U. martinsi* Sosa**
15’	Head orange, gena, clypeus pale, without black stripe; scape with dorsal stripe extending ~3/4^th^ distance from apex to base, not on antennal fossa (Fig. 73)	***U. pennyi* Tauber, sp. n.**
16(14)	Dorsum of scape with dark brown distal stripe; U-shaped mark on vertex brown (Figs 37d, 38d); crossveins below stigma of forewing without fumose marks (Fig. 39)	***U. favrei* (Navás)**
16’	Dorsum of scape with red or reddish brown distal stripe (Fig. 93); U-shaped mark on vertex dark red; four crossveins below stigma of forewing with fumose markings (Fig. 94)	***U. yutajensis* Sosa**
17(10)	Gena unmarked (Fig. 68e); forewing with entirely green venation, no suffusion (Fig. 69)	***U. pallescens* Penny**
17’	Gena with black, brown or reddish mark; some veins of forewing with brown marks (small or extensive)	**18**
18(17)	Vertex with U-shaped mark prominent, thick, with mesal arm extending laterally to eyes (Fig. 85); longitudinal veins of forewing with extensive dark marks (Fig. 86)	***U. siderocephala* (Navás)**
18’	Vertex with U-shaped mark reduced to a pair of small, red spots on anterolateral margin, far removed from eye (Fig. 53); longitudinal veins of forewing entirely green, or with very small brown marks only at intersections with transverse veins (Fig. 54a)	***U. laufferi* (Navás)**

#### Ungla adamsi

Taxon classificationAnimaliaNeuropteraChrysopidae

Tauber sp. n.

http://zoobank.org/14401863-D6B9-4BAD-8C57-685957EC1D69

Figs 4 , 5 , 6 , 7 , 8 , 9 , 10 , 144a

##### Holotype

**Figure 4.**
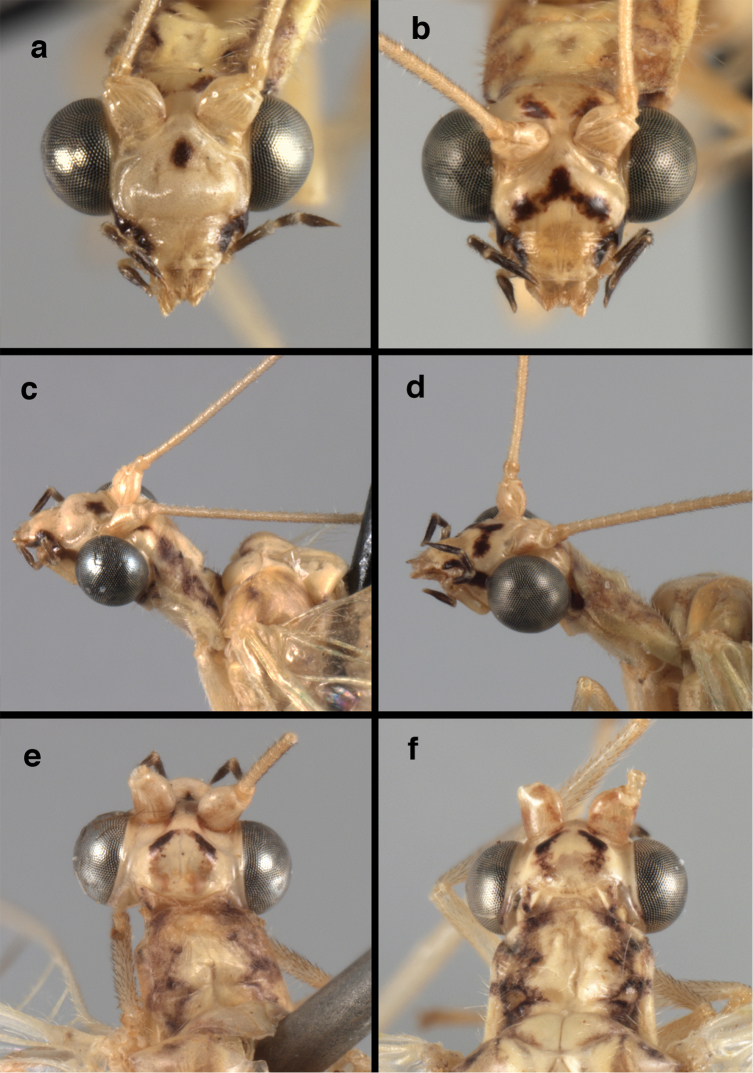
*Ungla adamsi* Tauber, sp. n.: Sexual dimorphism; male on left, female on right, (**a, b**) head, frontal (**c, d**) head, prothorax, lateral (**e, f**) head, prothorax, dorsal. Note: male, frons inflated distally, mesal spot round; female, frons not inflated, marking extended laterally (all: Peru, Machu Picchu; **a, c** holotype, FSCA; **b, d** paratype, FSCA; **e** paratype, BMNH; **f** paratype, BMNH).

**Figure 5.**
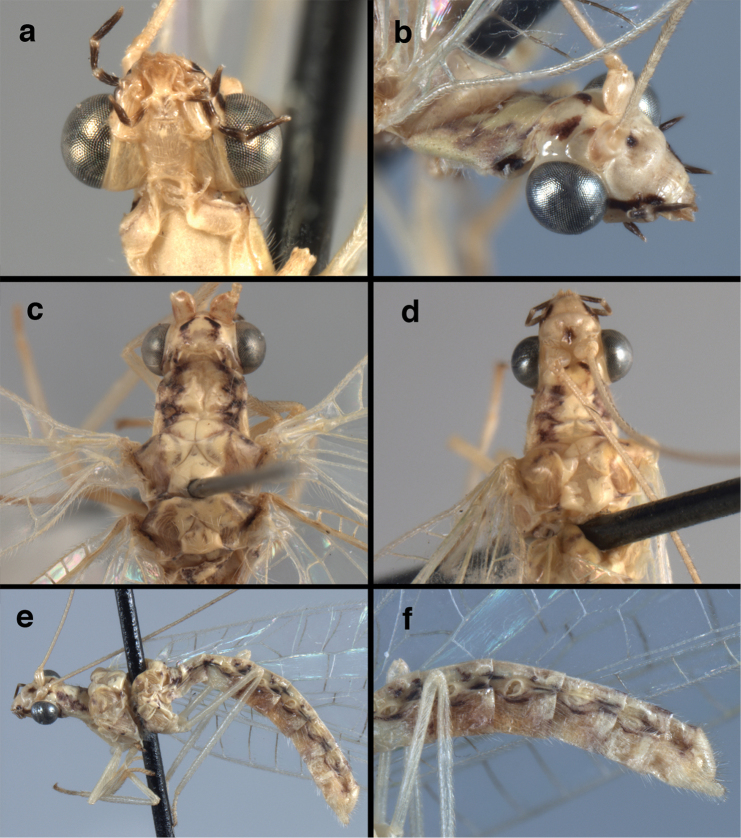
*Ungla adamsi* Tauber, sp. n. External features, (**a**) head, ventral (**b**) head, dorsolateral (**c, d**) head, thorax, dorsal (**e**) body, lateral (**f**) abdomen, lateral (all: Peru, Machu Picchu; **a, b** paratype, male, FSCA; **c** paratype, female, BMNH; **d, e, f** holotype, male, FSCA).

(Figs 4a, c, 5d, e, f, 7, 9b–e; labels: Fig. 144a). FSCA, male, Machu Picchu, 1/XII/1965, H. & M. Townes.

##### Etymology.

The species is named for Phillip A. Adams, an intense and talented contributor to systematics and evolutionary studies of Neuroptera. His publications, collection, and unpublished work on the lacewings of Latin America were of great help to this study, especially during the early stages. Indeed, Adams identified this species as new, thus the name “*adamsi*”. From his notes, it is clear that he, as did we, often found working with this genus to be perplexing.

##### Diagnosis.

*Ungla adamsi*, currently known only from the Andean archeological site of Machu Picchu, Peru, has several distinguishing features. Most notably, the males and females are strongly dimorphic in head markings and frontal modifications. In males the frons is enlarged and inflated above the clypeus, and it is marked with a prominent dark, central spot. Females also have a dark frontal spot, but in the female, the spot extends transversely towards the distal margin of the frons, forming a thick, inverted “V-shaped” mark. The female frons is not enlarged.

*Ungla adamsi* resembles three other *Ungla* species that have frontal spots and cream-colored flagella – *U. stangei* sp. n., *U. favrei*, and *U. nigromaculifrons*. However, none of these species express sexual dimorphism in the frons. *Ungla adamsi* also differs in that it lacks the features that distinguish the other three species. That is, it has diffuse reddish marks on the scape, not a distinct stripe like the one that typifies *U. favrei*. It has moderately sized forewings (< 15 mm), with 7-8 gradate veins per series, not the very large wings that distinguish *U. stangei*; and the veins on its hindwings are green, unlike the very dark transverse veins on the fore and hindwings of *U. nigromaculifrons*.

**Figure 6.**
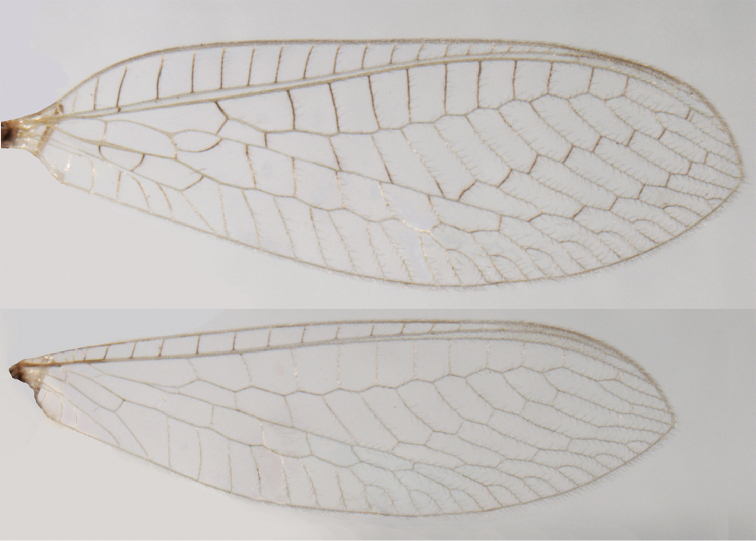
*Ungla adamsi* Tauber, sp. n. Wings (Peru, Machu Picchu, paratype, female, FSCA).

**Figure 7.**
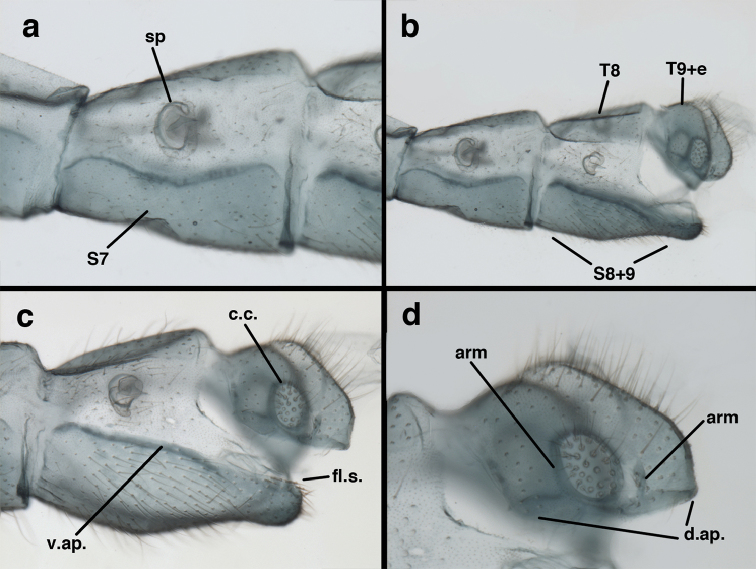
*Ungla adamsi* Tauber, sp. n. Mature male abdomen, lateral, (**a**) segment A7, with enlarged spiracle and concave sternite (**b**) segments A7-A9 (**c**) terminus (**d**) ninth tergite + ectoproct. **c.c.** callus cerci **d.ap.** dorsal apodeme **fl.s.** flanged setae **sp** spiracle **S7** seventh sternite **S8+9** fused eighth and ninth sternites **T8** eighth tergite **T9+e** fused ninth tergite and ectoproct **v.ap.** ventral apodeme (all: Peru, Machu Picchu, holotype, FSCA).

##### Description.

Head cream-colored with dark to light reddish black markings; vertex with anterior marking dark, prominent, separated or contiguous mesally, with diffuse coloration, not extending anteriorly toward antennal base; lateral marks on vertex diffuse, extending from anterior dark mark to base of vertex; dorsal fossa with small red stripe mesally (usually hidden by scapes); posterior parts of vertex unmarked; frons swollen distally (male), smooth distally (female), with large, prominent mesal mark, otherwise whitish (male), cream-colored with inverted V-shaped mark extending from mesal spot (female); gena, lateral margin of clypeus with black stripe throughout; tentorial pits amber-colored. Antenna: scape cream-colored, dorsum with diffuse, light reddish marks; pedicel amber or with amber-colored ring, flagellum cream-colored; maxillary palp with basal two segments pale, three distal segments black, articulations pale; labial palp with basal segment pale, distal two segments marked with black, articulation pale. Corner of cervix with large, dark brown to black mark.

Thorax with distinct longitudinal cream-colored stripe mesally. Prothorax with pair of broad, dark reddish brown stripes laterally; small transverse furrow in posterior region of segment, extending to pair of pale spots within sublateral stripe; setae mostly elongate, pale. Measurements: head width: 1.3 mm; ratio head width : eye width: 2.7–2.8 : 1; prothorax width: 0.6–0.8 mm, length: 0.9–1.0 mm.

Forewing somewhat broad, apex broadly subacute to rounded, membrane clear, hyaline, with light to very light suffusion of brown surrounding gradate veins; stigma very slightly opaque; longitudinal veins green; transverse veins brown or marked with brown; forewing with veins robust to slender, not crassate; Rs straight; first intramedian cell ovate; basal inner gradate vein meeting Psm; gradate veins, subcostal crossveins below stigma dark brown, costal crossveins, R-Rs crossveins, intracubital crossveins marked with brown basally. Hindwing narrow, apex subacute, membrane clear, hyaline, without markings; venation green, except costal crossveins, subcostal crossvein with brown at least basally. Forewing 13.5–14.8 mm long, 4.5–4.8 mm wide [ratio (length to width), L : W = 3.0–3.1: 1]; height of tallest costal cell 0.9 mm (cell number 5); width of first intramedian cell 0.9–1.2 mm; 11–12 radial cells (closed cells between Radius and Radial sector); third gradate cell 1.7–1.8 mm long, 0.4 mm wide (ratio, L : W = 3.9–4.4 : 1); fourth gradate cell 1.5–1.8 mm long, 0.4 mm wide (ratio, L : W = 3.6–4.2 : 1); 4 Banksian cells, 4 b’ cells; 7 inner gradates, 7–8 outer gradates. Hindwing 12.3–13.4 mm long, 3.6–4.2 mm wide (ratio, L : W = 3.2–3.4 : 1), 11–12 radial cells, 3 Banksian (b) cells, 4 b’ cells, 5–6 inner gradates, 7 outer gradates.

Male: Abdomen with large spiracles, elongate segments (e.g., A7: spiracle diameter ~0.14-0.18x length of sternite); T9+ect relatively long (~0.5 length of T7), with dorsal invagination moderately deep (~0.5× dorsal length of T9+ect), margins of invagination almost straight, base rounded; dorsal margin of T9+ect straight basally, rounded distally, posterior margin of ectoproct slightly convex, without knob or extension; ventral margin convex (teneral) to straight (mature); dorsal apodeme extending along full ventral margin of T9+ect, with three arms projecting dorsally (mature): first arm extending along posterior margin of segment, second arm contiguous with sclerotization around callus cerci, third arm extending dorsally distal to callus cerci; callus cerci large, slightly ovate, with discrete, well separated trichobothria. S8+9 fused, with line of fusion not detectible; ventral apodeme extending along dorsal margin for full length of A8; dorsum of S8+9 tapering gradually, then forming concave ledge at terminus; terminus blunt (lateral view), extending slightly beyond distal margin of T9+ect; setae slender, mostly long, simple, those along distolateral margin large, flanged. Subanal plate fairly large, triangular in shape, with ~9 setae of medium length. Gonarcus broadly arcuate, rounded mesally, with bridge relatively slender, arms elongate, narrow throughout (lateral view), extending straight downward from gonarcal bridge, margin rounded distally, mesal section with angular enlargement, distinct digitiform process extending posteriorly, inward; mediuncus with quadrate base closely attached to gonarcal bridge, narrow distally, with terminus spoon-shaped, slightly curved downward throughout (lateral view); gonosaccus bilobed, with lobes closely aligned, when unexpanded forming a triangular envelope around tip of mediuncus, when expanded consisting of two lobes, rounded dorsally, mesally, laterally, but flat distally; each lobe with large, dense patch of gonosetae arising from prominent setal bases facing inward when uneverted, outward when everted; gonosetae moderate length (shorter than length of mediuncus); hypandrium internum small, with two short, dense arms, with narrow, rounded junction.

**Figure 8.**
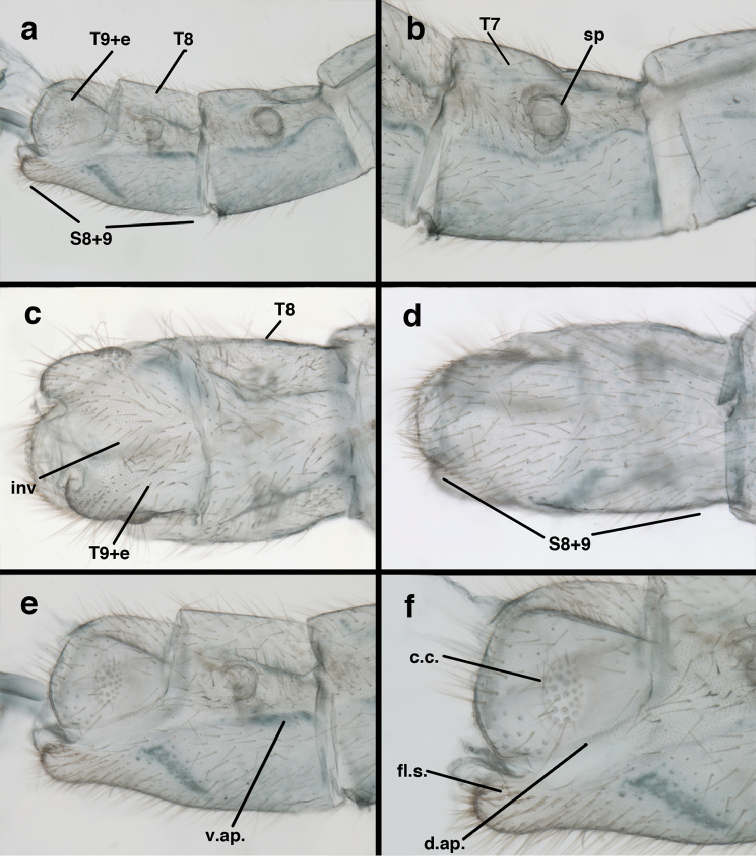
*Ungla adamsi* Tauber, sp. n. Slightly teneral male abdomen, (**a**) segments A7-terminus, lateral (**b**) segment A7 with enlarged spiracle, lateral (**c**) segments A8, A9+ectoproct, dorsal [Note deep mesal invagination of posterior margin.] (**d**) fused eighth and ninth sternites, ventral (**e**) segments A8-A9, lateral (**f**) terminus, lateral. **c.c.** callus cerci **d.ap.** dorsal apodeme (longitudinal apodeme along ventral margin of ectoproct) **fl.s**. flanged setae **inv** invagination of T9+ectoproct **sp** spiracle **S8+9** fused eighth and ninth sternites **T7** seventh tergite **T8** eighth tergite **T9+e** fused ninth tergite and ectoproct **v.ap.** ventral apodeme (apodeme along dorsal margin of S8+9) (all: Peru, Machu Picchu, paratype, FSCA).

**Figure 9.**
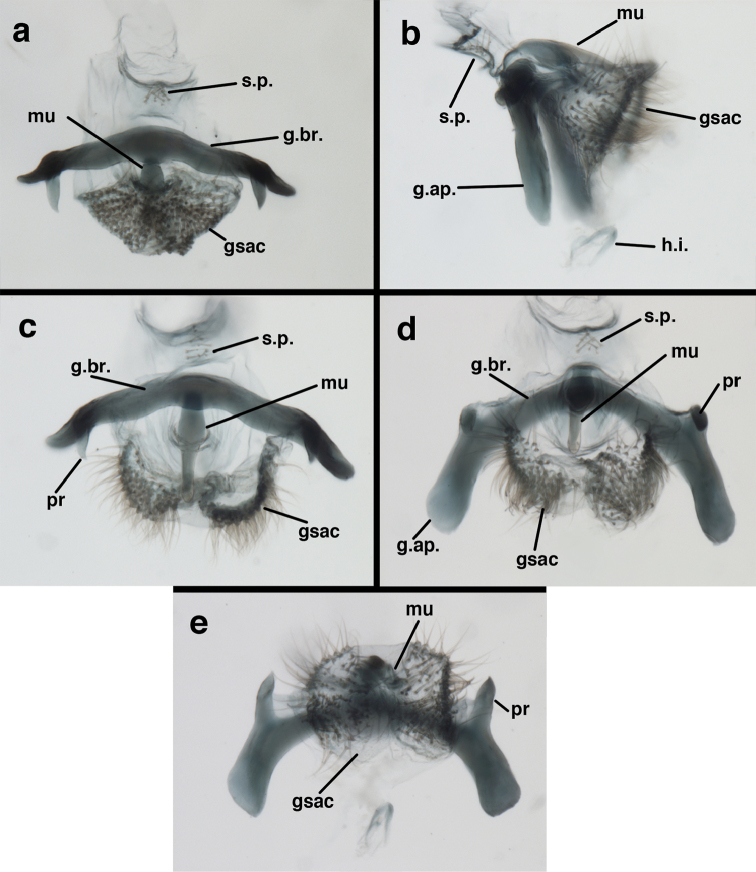
*Ungla adamsi* Tauber, sp. n. Male genitalia (mature), (**a, b**) gonosaccus unexpanded, dorsal (**a**) and lateral (**b**) (**c, d, e**) gonosaccus expanded, sequence of gonarcus rolling backward from dorsal view (**c**) to frontal view (**e**). **g.ap.** gonarcal apodeme **g.br.** gonarcal bridge **gsac** gonosaccus **h.i.** hypandrium internum **mu** mediuncus **pr** unarticulated process on frontal margin of gonarcal apodeme **s.p.** setose subanal plate (all: Peru, Machu Picchu; **a** paratype, FSCA; **b–e** holotype, FSCA).

**Figure 10.**
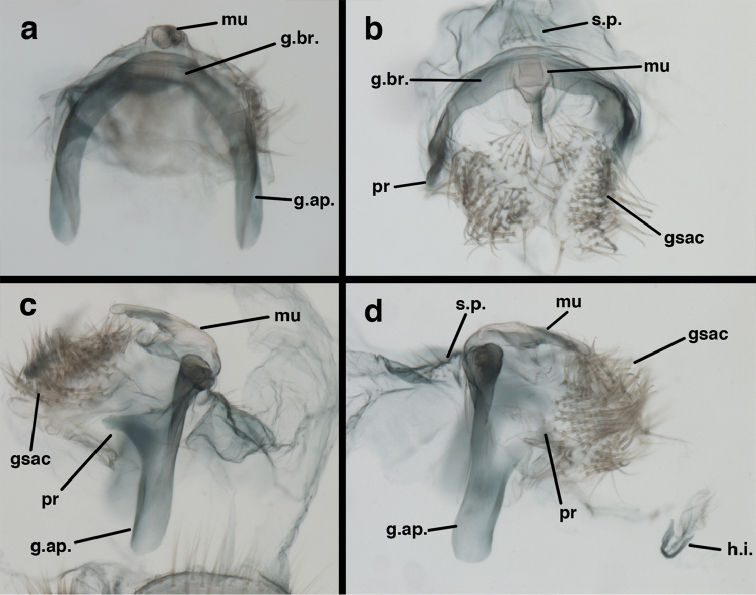
*Ungla adamsi* Tauber, sp. n. Male genitalia (slightly teneral), (**a**) gonarcus, posterior (mediuncus extending into photo) (**b**) gonarcus, dorsofrontal (**c**) gonarcus, left, lateral (**d**) gonarcus, right, lateral. **g.ap.** gonarcal apodeme **g.br.** gonarcal bridge **gsac** gonosaccus **h.i.** hypandrium internum **mu** mediuncus **pr** unarticulated process on frontal margin of gonarcal apodeme **s.p.** setose subanal plate (all: Peru, Machu Picchu, paratype, FSCA).

##### Known distribution.

PERU: Department of Cuzco.

##### Specimens examined

(in addition to the holotype; all paratypes). Peru; Cuzco, Machu Picchu museum, 1,385 m., 11-14/VIII/1971 at light, C. & M. Vardy. B.M. 1971-533 (M, BMNH); Machu Picchu, 6/VIII/1971, B.V. Ridout, B.M.1971-, n. sp., F., det. P. Adams (F, BMNH); Machupicchu, nr. Cuzco, 5-8/VIII/1973, B. V. Ridout, B.M.1974-181 (M, BMNH); Machu Picchu, 28/XI/1965 (F, FSCA), 1/XII/1965, H. & M. Townes (2M, FSCA).

#### Ungla banksi

Taxon classificationAnimaliaNeuropteraChrysopidae

Tauber, new replacement name

**Figure 11.**
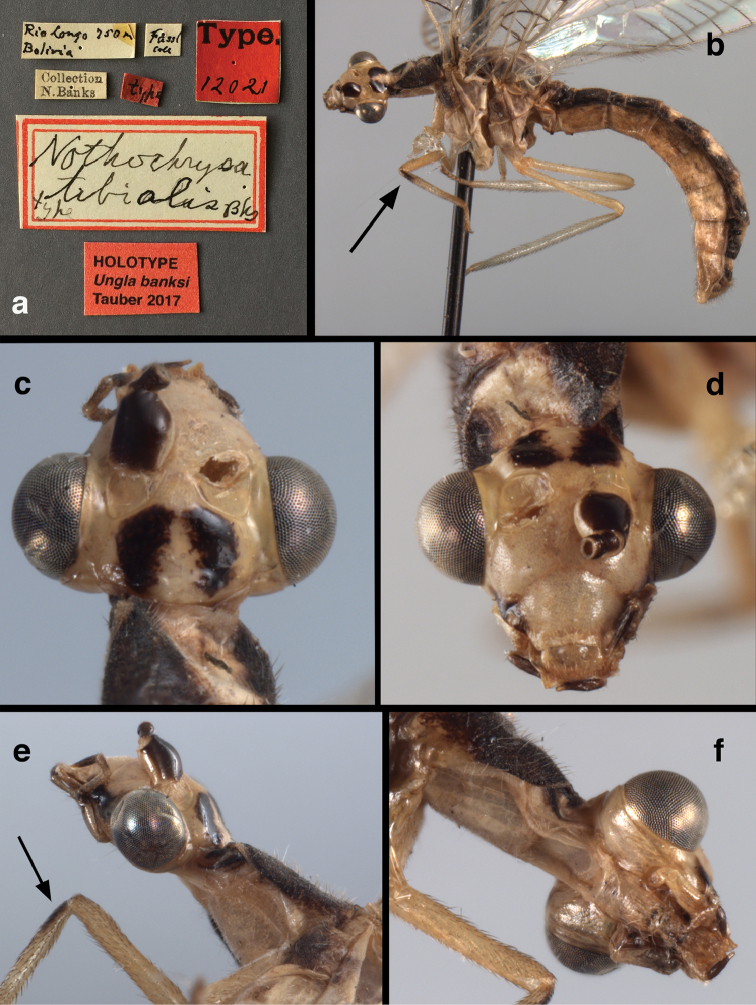
*Ungla banksi* Tauber, new replacement name: External features, (**a**) labels (**b**) habitus, lateral (**c**) head, dorsal (**d**) head, frontal (**e**) head, prothorax, lateral (**f**) head, prothorax, ventral. Arrows indicate dark, foretibial spot (Bolivia, Rio Zongo, holotype, male, MCZ).

**Figure 12.**
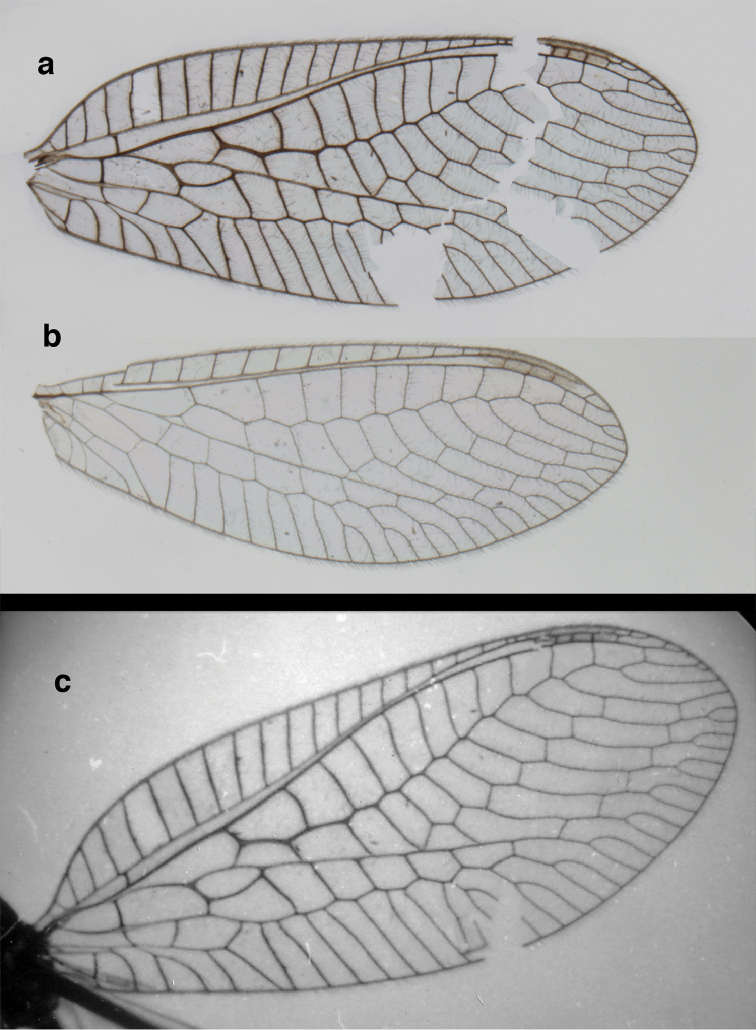
*Ungla banksi* Tauber, new replacement name: Wings, (**a**) forewing (damaged) (**b**) hindwing (**c**) photo of forewing taken by P. A. Adams (~1970) before severe damage (Bolivia, Rio Zongo, holotype, male, MCZ).

**Figure 13.**
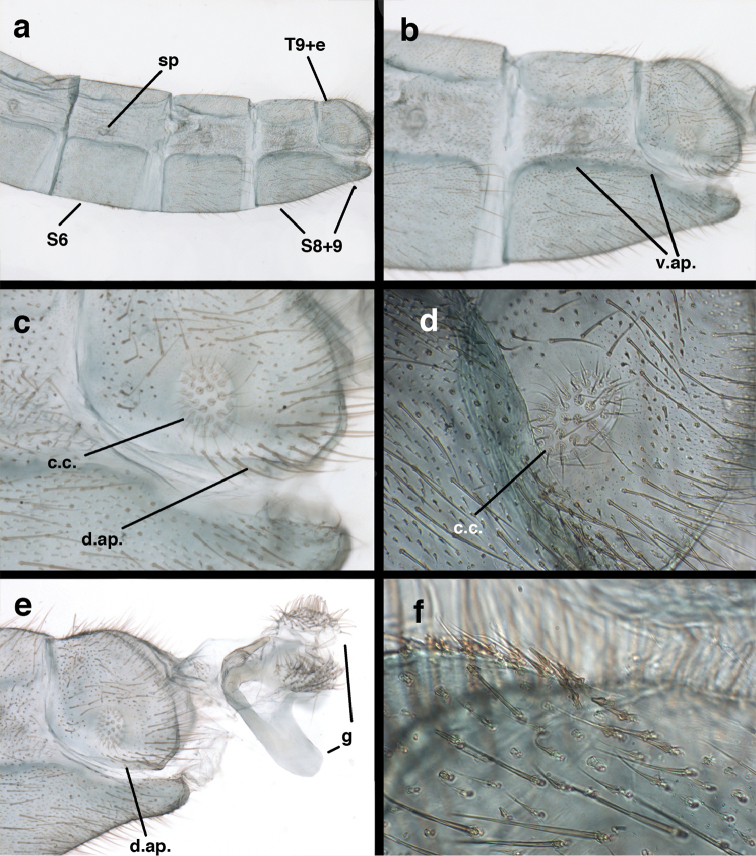
*Ungla banksi* Tauber, new replacement name: Male abdomen, (**a**) segments A6-terminus, lateral [Note slightly enlarged spiracles.] (**b**) terminalia, lateral (**c**) tip of tergite 9+ectoproct, lateral (**d**) callus cerci (**e**) tip of abdomen with gonarcal complex everted (**f**) flanged setae at tip of S9. **c.c.** callus cerci **d.ap.** apodeme on ventral margin of T9+ectoproct **g** gonarcus **sp** spiracle **S6** sixth sternite **S8+9** fused eighth and ninth sternites **T9+e** fused ninth tergite and ectoproct **v. ap.** ventral apodeme (Bolivia, Rio Zongo, holotype, MCZ).

**Figure 14.**
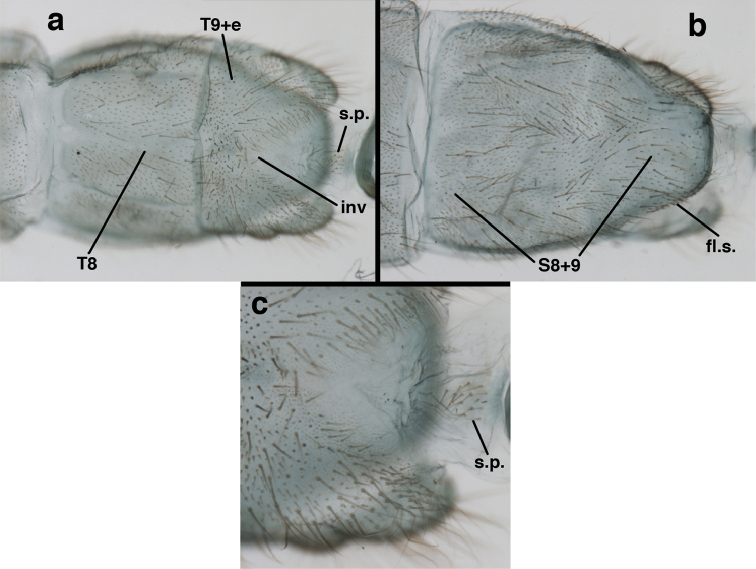
*Ungla banksi* Tauber, new replacement name: Male abdomen, (**a**) terminal segments, dorsal (**b**) terminal segments, ventral (**c**) tip of tergite 9 + ectoproct, dorsal. **fl.s.** flanged setae **inv** dorsal invagination of T9+ectoproct **s.p.** setose subanal plate **S8+9** fused eighth and ninth sternites **T8** eighth tergite **T9+e** fused ninth tergite and ectoproct (Bolivia, Rio Zongo, holotype, MCZ).

**Figure 15.**
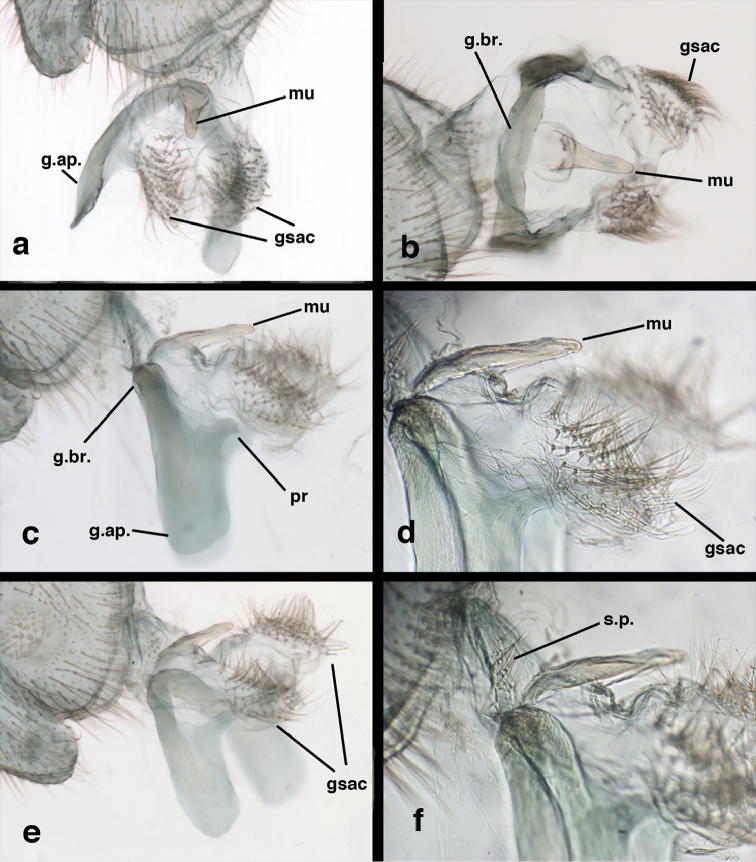
*Ungla banksi* Tauber, new replacement name: Male genitalia, (**a**) gonarcus, frontal (**b**) gonarcus, dorsal (**c**) gonarcus, lateral (**d**) gonarcus (partial), lateral (**e**) gonarcus, anterolateral, showing widely separated, paired lobes of gonosaccus (**f**) tip of tergite 9 + ectoproct, with subanal plate above gonarcal bridge **gsac** gonosaccus **g.ap.** gonarcal apodeme **g.br.** gonarcal bridge **mu** mediuncus **pr** unarticulated process on frontal margin of gonarcal apodeme **s.p.** setose subanal plate (Bolivia, Rio Zongo, holotype, MCZ).

**Figure 16.**
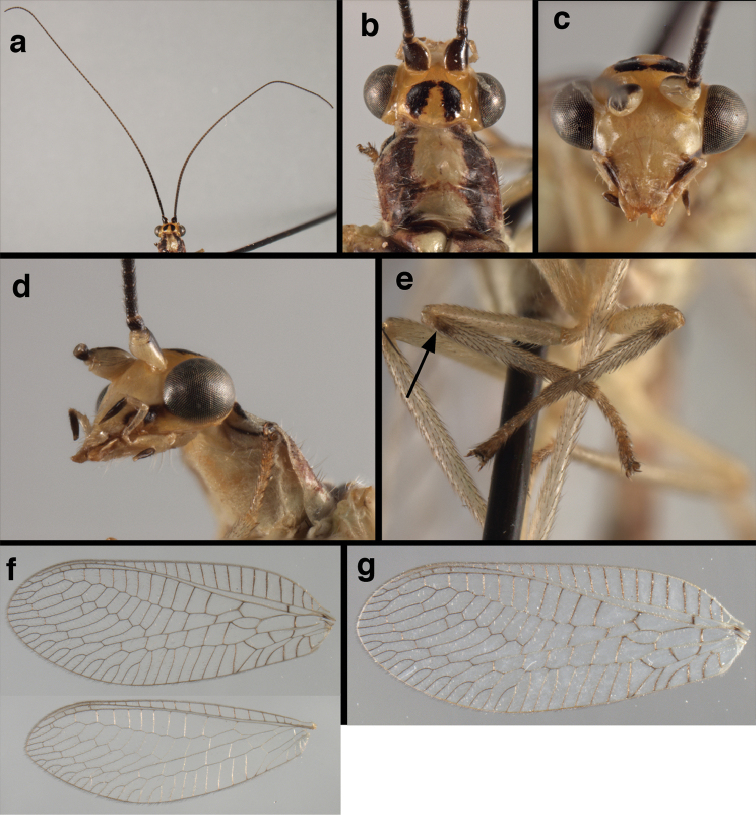
*Ungla banksi* Tauber, new replacement name: External features, (**a**) head, antennae, dorsal (**b**) head, prothorax, dorsal (**c**) head, frontal (**d**) head, prothorax, lateral (**e**) forelegs, frontal [arrow indicates foretibial mark.] (**f**) wings, venation emphasized (**g**) forewing, coloration of veins emphasized (Bolivia, La Paz, female, FSCA).

Figs 11 , 12 , 13 , 14 , 15 , 16

Nothochrysa tibialis Banks, 1914. *Canad. Entomol.* 46: 26-27; “Rio Longo, Bolivia, 750 m. (Fassl.)”. Penny 1977: 28 (list); Oswald 2015 [catalog listing as “Available, invalid, species, unreplaced junior homonym …”]. Chrysopa tibialis (Banks), Brooks and Barnard 1990: 280 [as “ ‘Chrysopa’ incertae sedis (? Leucochrysa)”]. Junior homonym, preoccupied by Nothochrysa tibialisNavás 1913 [now Italochrysa tibialis (Navás, 1913)]. **Holotype** (Figs 11–15). In accordance with Recommendation 60A of the International Code of Zoological Nomenclature, we retain Banks’ type specimen (MCZ Type #12021, Male) as the holotype. Label data: (1) “Rio Zongo 750 m / Bolivia”; (2) “Fassl / coll”; (3) “Collection / N. Banks”; (4) “type” [red]; (5) “Type / 12021” [red]; (6) “Nothochrysa / tibialis Bks / type” [large, white, red border]; (7) “HOLOTYPE / Ungla banksi / Tauber et. al 2017” [red]. We could not find the locality “Rio Longo” in Bolivia. According to Dr. Newton of the Field Museum of Natural History, Chicago (quoted in an email from A. Contreras-Ramos, Universidad Nacional Autónoma de México), the term probably is a misspelling of “Rio Zongo”, a river in La Paz province, 15°43'S, 67°41'W. Thus, we presume that Banks misread Fassl’s handwritten “Z” as an “L”. **Justification for name change.** The species is a valid biological entity within the genus Ungla; its original name (Nothochrysa tibialis Banks, 1914) was preoccupied (Oswald 2015). Because the name is a primary homonym, Articles 57.2 and 60.1 of the International Code of Zoological Nomenclature require the designation of a replacement name. In recognition of Nathan Banks’ discovery and original description of the species, we offer the name Ungla banksi as the replacement.

##### Diagnosis.

This species is distinguished by pronounced, dark brown body markings; dark brown or black marking on frontal base of foretibia; tall wings with rounded apices, forewing with very dark venation, hindwing venation less dark; stark, shiny, dark brown to black markings on dorsum of scape and vertex; moderately enlarged abdominal spiracles; distal setae of S8+9 larger than those on ventral or proximal region, but not exceptionally robust; those on narrow strip of distolateral margin flanged. Forewing 13.0 mm long, 5.5 mm wide; hindwing 12.1 mm long, 4.4 mm wide.

##### Redescription.

Head cream-colored, with vertex smooth, shiny, with inverted U-shaped marking prominent, dark brown, broad, separated anteromesally, not extending anteriorly to area between scapes; dorsal antennal fossa pale; area between eyes and posterior half of vertex cream-colored, unmarked; frons, clypeus cream-colored, without markings; gena with large, brown mark from eye onto clypeus; tentorial pits amber. Antenna with scape cream-colored, unmarked ventrally, laterally, entirely dark brown dorsally; pedicel, flagellum dark brown to black; maxillary palp, labial palp with basal segments pale, ultimate segment dark brown.

Prothorax with pair of wide, brown stripes laterally, extending mesally and becoming reddish along transverse furrow, pale green mesally; transverse furrow in posterior region, not reaching lateral margins of segment, golden to dark brown setae throughout. Mesothorax, metathorax with pair of broad, dark brown stripes laterally, pale green mesally. Legs pale, unmarked except for small dark brown spot near anterior base of foretibia. Measurements: head width: 1.5 mm; ratio head width : eye width: 3.0–3.2 : 1; prothorax width: 1.2 mm, length: 0.9 mm.

Forewing, hindwing broad, with round apices. Forewing with venation heavy, especially at base of R, Rs, M, Cu; stigma lightly opaque, with four dark brown subcostal crossveins below, area surrounding subcostal crossveins marked with dark brown; longitudinal veins mostly green, with brown at intersections; transverse veins, crossveins mostly brown or dark brown, most with dark brown suffusion on surrounding membrane. Forewing 13.4–13.9 mm long, 5.3–5.5 mm wide (ratio, L : W = 2.4–2.6 : 1); height of tallest costal cell 1.2 mm (cell number 8); length of first intramedian cell 1.1–1.2 mm; 11–12 radial cells (closed cells between R and Rs); third gradate cell 2.3 mm long, 0.4 mm wide (ratio, L : W = 5.5 : 1); fourth gradate cell 2.3 mm long, 0.4 mm wide (ratio, L : W = 5.1 : 1); 3–4 Banksian cells (b cells), 4 b’ cells; 7 inner gradates, 6–8 outer gradates, sometimes a middle gradate vein. Hindwing with venation delicate, not swollen, light green, except C, Sc, costal veinlets brown; 12.1–12.4 mm long, 4.4 mm wide (ratio, L : W = 2.7–2.8 : 1), 11–12 radial cells, 2–3 Banksian (b) cells, 4 b’ cells, 5–6 inner gradates, 6–7 outer gradates.

Male: Abdomen with slightly enlarged spiracles (e.g., A7: spiracle diameter ~0.10× length of sternite); T9+ectoproct relatively long (~two thirds times length of T7), with dorsal invagination deep (~0.6× dorsal length of T9+ect), margins of invagination almost straight, base rounded; dorsal margin of T9+ect rounded distally (above anus), often compressed, thus appearing straight; posterior margin of ectoproct short, slightly concave; dorsal apodeme extending along ventral margin of entire segment, lightly sclerotized, posterior corner extending, bending mesally to form small, rounded knob; callus cerci large, ovate, with entire margin lightly sclerotized. S8+9 fused, with line of fusion hardly perceptible; dorsum tapering abruptly to shallow platform at ¾ distance to tip of segment, dorsal margin regular, ventral apodeme well sclerotized; terminus concave to flat, extending distally, slightly beyond tip of T9+ect, flat, distal margin upturned, heavily sclerotized; terminal setae enlarged, with flange-line protrusions basally. Gonarcus arcuate, with bridge slightly angled dorsally, arms robust, elongate, slightly curved inward, rounded distally, mesal section with digitiform process extending posteriorly and inward toward gonosaccus; mediuncus with heavy base, ridged dorsally, tapering to elongate, blunt terminus; gonosaccus bilobed, each lobe with elongate patch of gonosetae; gonosetae arising from enlarged setal bases, dense, large at tip of lobe, becoming smaller, scarcer basally near base of mediuncus; hypandrium internum not found.

##### Variation.

The thickness and the depth of the brown coloration of the veins on the forewing were considerably more robust in the male specimen than in the female.

##### Known distribution.

BOLIVIA: Departments of La Paz, Santa Cruz.

##### Specimens examined

(in addition to type above). Bolivia. Santa Cruz, Florida, 11 km. N.E. Achira, 1800 m, 3/XI/1999, cloud forest, Malaise trap, C. Porter & L. Stange (1F, FSCA).

#### Ungla bolivari

Taxon classificationAnimaliaNeuropteraChrysopidae

(Banks, 1913) comb. n.

Fig. 17

**Figure 17.**
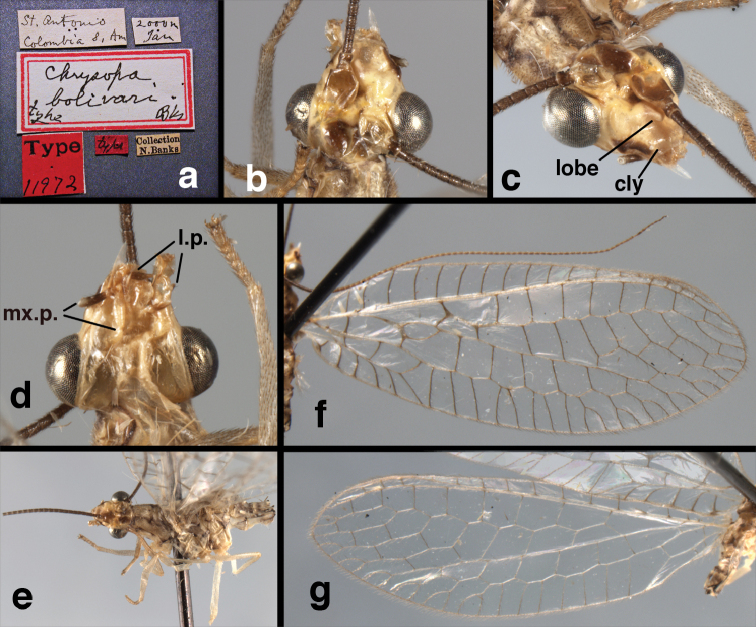
*Chrysopa bolivari* Banks: External features, (**a**) labels (**b**) head, dorsum, damaged (**c**) head, frontolateral, damaged [note: protruding mesal lobe beneath scape and bulbous clypeal area] (**d**) head, ventral (**e**) body, lateral (**f**) forewing (**g**) hindwing. **cly** clypeus **l.p.** labial palp **mx.p.** maxillary palp **lobe** protruding frontal lobe (all: Colombia, San Antonio, holotype, probably male, terminalia missing, MCZ).

Chrysopa bolivari Banks, 1913. *Proc. Entomol. Soc. Wash.* 15: 140; “San Antonio, Colombia, January, 2000 m. (Fassl.)”. Penny 1977: 16 (list); Brooks and Barnard 1990: 279 [list, as “ ‘Chrysopa’ incertae sedis”]; Oswald 2015 (catalog). **Holotype.** (Fig. 17) MCZ (examined). Although the abdomen is missing, the frontal horn and crassate wing venation are typical of a male. The head and body are flattened; thus many structures could not be observed and/or measured accurately. It seems unlikely that Banks had specimens other than this one when he described the species; it is labeled as the type in his hand. Thus, we consider it to be the holotype by original designation. San Antonio, the type locality, is a small city in Tolima Department, Colombia (altitude, ~1500 m). **Support for generic placement.** Currently, this species is known only from the holotype, which is lacking its abdomen. Here, we base our generic identification on several external features that characterize Ungla species: vertex with pair of large, dark marks, distal segments of the maxillary and labial palpi dark brown, and a basal inner gradate that does not meet the Psm. **Diagnosis.** Based on the type specimen, it appears that U. bolivari can be distinguished from other Ungla species by the following suite of external features: small, rounded wings, with tan to light brown longitudinal veins, darker brown transverse veins; several basal veins crassate; large, shiny, dark brown marks on the vertex; frons with single, brown mesal spot and with a small mesal lobe protruding below the scapes (probably males only); clypeal region enlarged (boxy) frontally; antenna with flagellum and pedicel dark brown, scape dark brown dorsally.

##### Redescription

(based on holotype). Head: vertex cream-colored, with large, shiny, dark brown marks; posterolateral region of head probably with brown stripe near margin of eye; frons cream-colored with single brown spot mesally, below scapes, with distinctly protruding, mesal lobe; clypeus cream-colored, anterior margin thickened, raised above labrum; labrum cream-colored, with anterior margin indented mesally; distal edge of gena, lateral margins of clypeus with dark brown stripe. Antenna with dorsal surface of scape dark brown, frontal surface probably cream-colored; pedicel, flagellum dark brown throughout.

Prothorax probably light brown, with narrow, brown, longitudinal stripe laterally, with transverse furrow in central region, ending at two small pale spots interior to lateral margins, with short, dark setae throughout. Mesothorax, metathorax light to dark brown, probably with darker brown markings [as described by Banks, but faded now]. Measurements: head width: 1.2 mm; ratio head width : eye width: 2.7 : 1; prothorax width, length: not measured.

Forewing, hindwing broad, rounded apically, with robust venation, with following veins crassate: R-Cux1, base of Rs, Rs-mx1, base of M near im1; alar membrane clear, without suffusion; stigma clear to very slight suffusion, with three to four light brown crossveins below with very slight tinge of brown suffusion; longitudinal veins tan to light brown, slightly darker at intersections; transverse veins, gradate veins, crossveins brown. Forewing 10.1 mm long, 3.8 mm wide, ratio, L : W = 2.7 : 1; height of tallest costal cell 0.8 mm (cell number 4); length of first intramedian cell 0.8 mm; 8 radial cells (closed cells between R and Rs); 4 Banksian cells (b cells), 4 b’ cells; 3 inner gradates, 3-4 outer gradates. Hindwing 9.4 mm long, 3.1 mm wide (ratio, L : W = 3.0 : 1), 8 radial cells, 3 b (Banksian) cells, 4 b’ cells, 3 inner gradates, 4 outer gradates.

Male and female. Abdominal characteristics unknown.

##### Known distribution.

COLOMBIA: Department of Tolima.

##### Specimens studied.

Holotype only. Also see below.

##### Possible sibling species from Venezuela.

The characteristics of *U. bolivari* are known with certainty only for the type specimen from Colombia. However, one female and two male specimens from Venezuela that resemble the *U. bolivari* holotype were studied. These specimens express many features that characterize *U. bolivari* (e.g., dark basal flagellomeres and dark brown to black markings on the vertex, gena, and lateral margins of clypeus). However, several features of the wings (size, number of gradate veins) lead us to believe that they are not conspecific with *U. bolivari*. Thus, we are not naming it here. However, to facilitate comparison with future specimens, here we include a description and images of the Venezuelan specimens.

**Figure 18.**
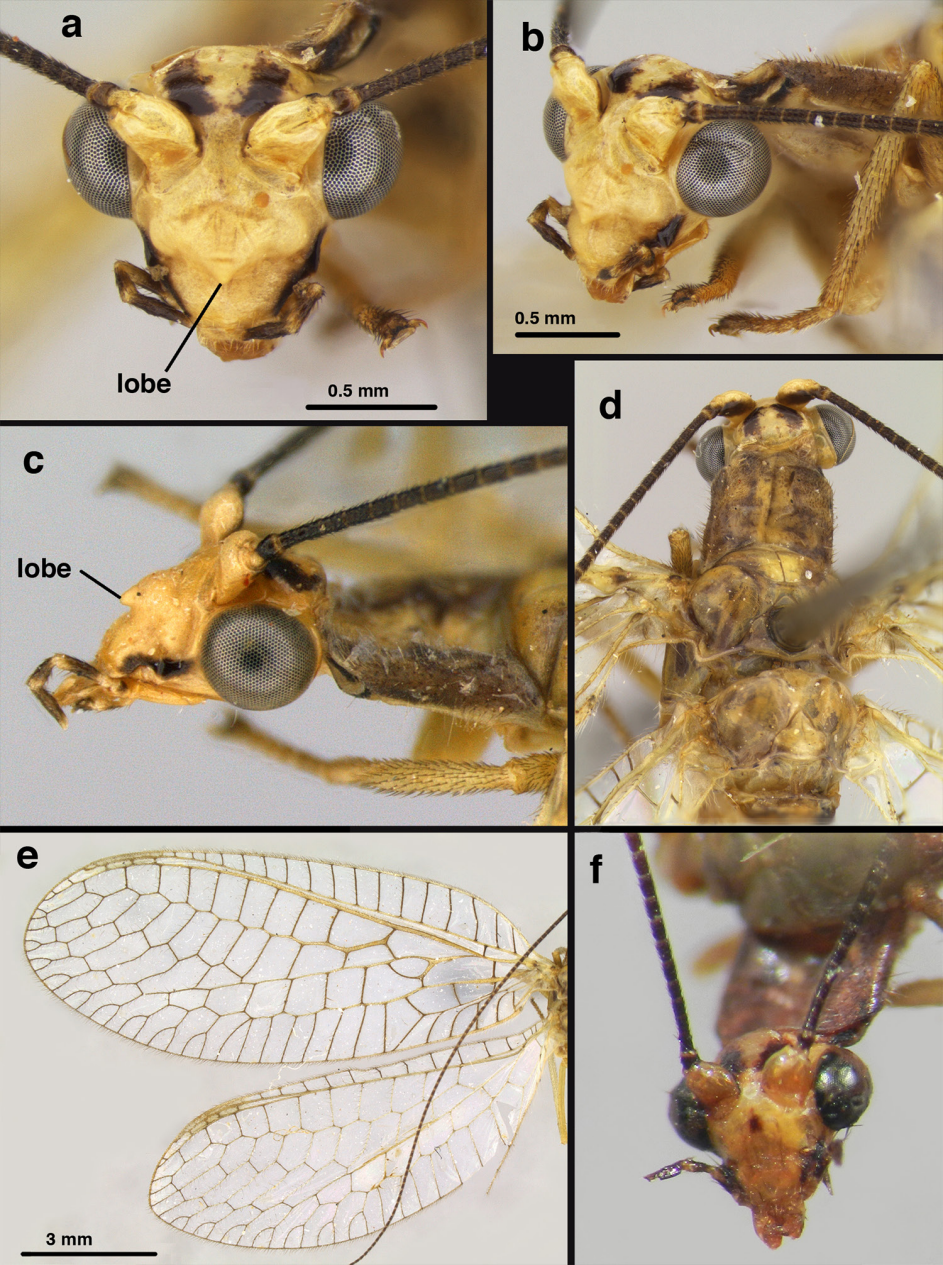
*Ungla bolivari*-like species: External features, (**a**) head, frontal (**b**) head, frontolateral (**c**) head, lateral (**d**) head, thorax, dorsal (**e**) wings, left (**f**) head dorsofrontal. (**a–e** Venezuela, Boconó, male, MIZA; **f** Venezuela, Lara, Yacambu, female, MIZA; all: images, FS).

**Figure 19.**
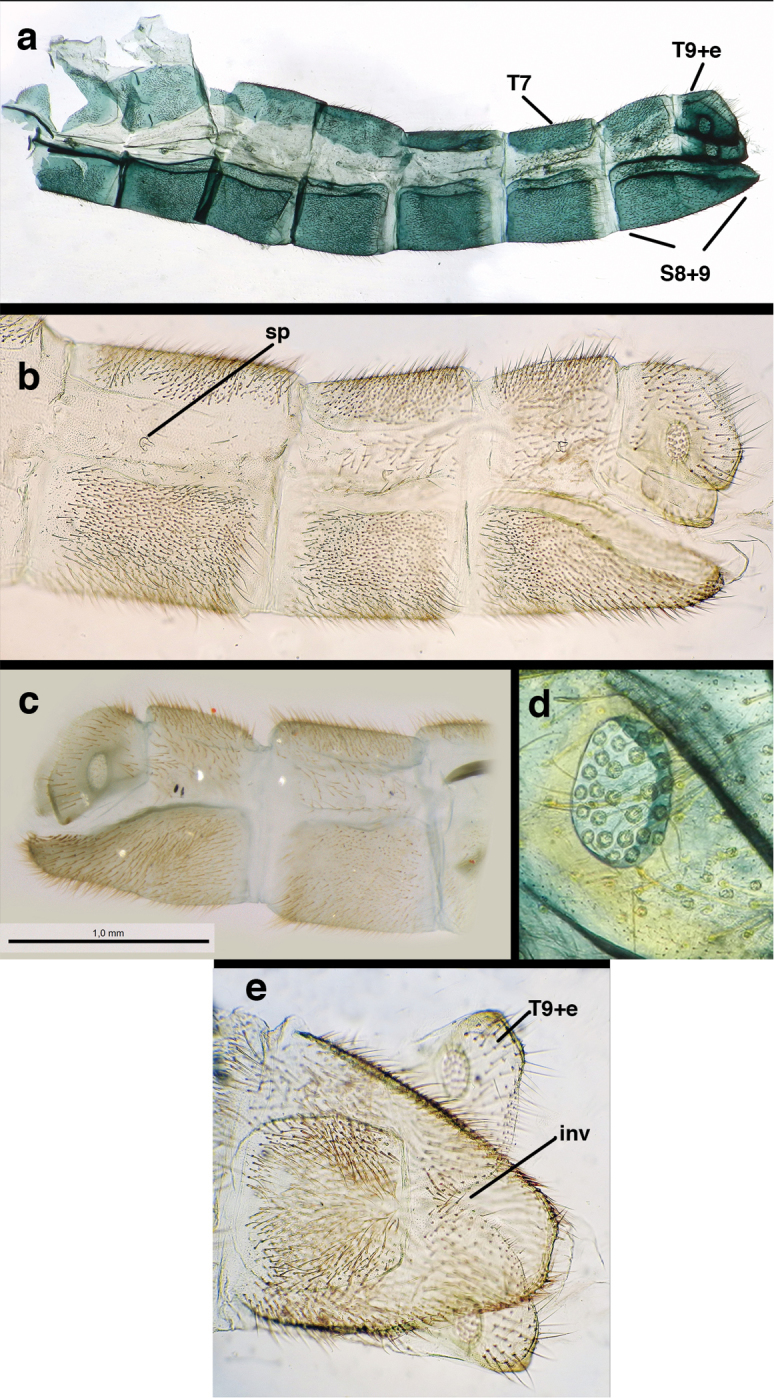
*Ungla bolivari*-like species: Male abdomen, (**a**) segments A3-terminus, lateral (**b**) segments A6-terminus, lateral (**c**) segments A7 – terminus, lateral, without coverslip (**d**) callus cerci (**e**) terminus, dorsal, with coverslip. **inv** invaginated dorsal cleft in T9+ectoproct **sp** spiracle **S8+9** fused eighth and ninth sternites **T7** seventh tergite **T9+e** fused ninth tergite and ectoproct (all: Venezuela, Boconó, MIZA; images, FS).

**Figure 20.**
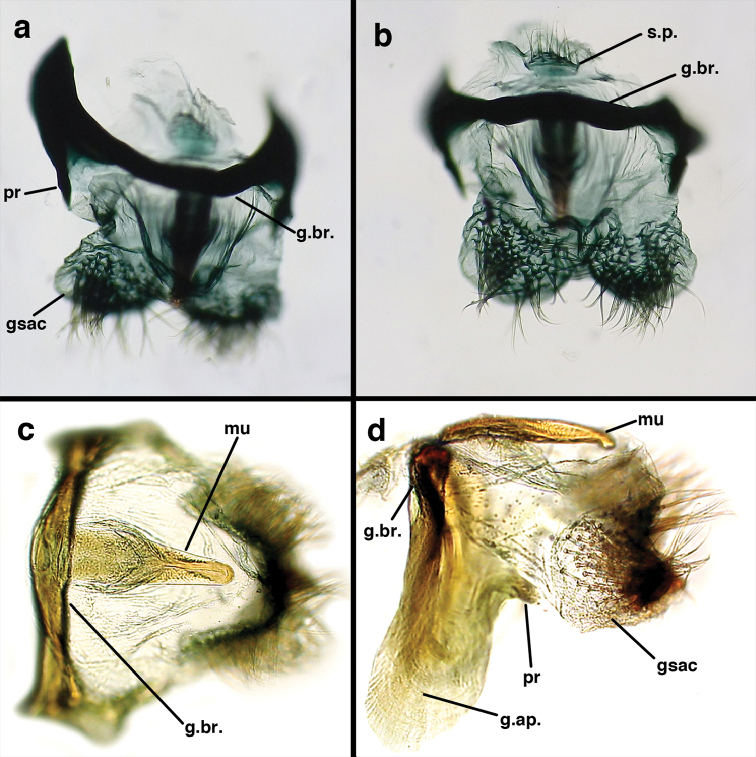
*Ungla bolivari*-like species: Male genitalia, (**a**) gonarcus, dorsal (**b**) gonarcus, frontodorsal (**c**) gonarcus and mediuncus, dorsal (**d**) gonarcus, lateral. **g.ap.** gonarcal apodeme **g.br.** gonarcal bridge **gsac** gonosaccus **mu** mediuncus **pr** unarticulated process on frontal margin of gonarcal apodeme **s.p.** setose subanal plate (all: Venezuela, Boconó, MIZA; images, FS).

*Description of Venezuelan specimens* (Figs 18–20). [For comparison: measurements from the *U. bolivari* holotype are provided in square brackets.]

Head, thorax as in *U. bolivari*, except male with frons lacking mesal marking and clypeus not enlarged or boxy; female with dark mesal spot on frons. Measurements: head width: 1.3 [1.2] mm; ratio head width : eye width: 3.5 : 1 [2.7 : 1]; prothorax length: 0.7 mm, width: 1.0 mm. Forewing, hindwing rounded, with robust venation; alar membrane clear, without suffusion; stigma clear to very slightly opaque, with three [four] light brown crossveins below with very slight tinge of brown suffusion; longitudinal veins cream-colored to tan, slightly darker at intersections; transverse veins, gradate veins, crossveins brown; male with following forewing veins crassate: R-Cux1, base of Rs, Rs-mx1, base of M near im1. Forewing 11.8 mm [10.1 mm] long, 4.6 [3.8] mm wide, ratio, L : W = 2.6 [2.7] : 1; height of tallest costal cell 0.9–1.0 mm (cell number 4) [0.8 mm (cell number 4)]; length of first intramedian cell 0.8 mm [0.8 mm]; 9-10 [8] radial cells (closed cells between R and Rs); 3 [4] Banksian cells (b cells), 4 [4] b’ cells; 6 [3] inner gradates, 6 [3-4] outer gradates; 5 [3] gradate cells. Hindwing 9.9 [9.4] mm long, 3.6 [3.1] mm wide (ratio, L : W = 2.7 [3.0] : 1), 10 [8] radial cells, 3 [3] Banksian cells (b cells), 4 [4] b’ cells, 5 [3] inner gradates, 5 [4] outer gradates.

Male: Abdomen yellowish green. Tergites (T) and sternites (S) densely covered with both elongate, thin and short, thickened setae. Spiracles small. T9+ect cone-shaped, with posteroventral corner acute; dorsal apodeme lightly sclerotized, located on the lower margin of T9+ect; callus cerci ovate with ca. 38 trichobotria. S8+9 fused, in lateral view: tall anteriorly, mesal section tapering abruptly, with distal one third ca. 2.8 times longer than tall, slightly projected beyond apex of T9+ect; ventral apodeme sclerotized, extending along dorsal margin of basal section of S8+9, not reaching apex. Gonarcal bridge narrow, arched; lateral apodemes broad with digitiform projection; mediuncus densely covered with microtrichiae from medial area to apex; gonosaccus with elongate gonosetae on robust chalazae in two lateral fields.

##### Specimens studied.

Venezuela. *Lara*: Parque Nacional Yacambú, El Blanquito, 1463m, 9.70649°N, 69.57608°W, 14-20/IX/2001, R. Briceño, J. Clavijo, A. Chacón, R. Paz. & E. Arcaya Proyecto S1-2000000479 (1F, MIZA). *Trujillo*: Boconó [9°14'N / 70°15'W, 1270 m], 20.vii.1974, F. Fernández. H. & M. Gaiani Legs. (2M, MIZA).

#### Ungla curimaguensis

Taxon classificationAnimaliaNeuropteraChrysopidae

Sosa, 2015

**Figure 21.**
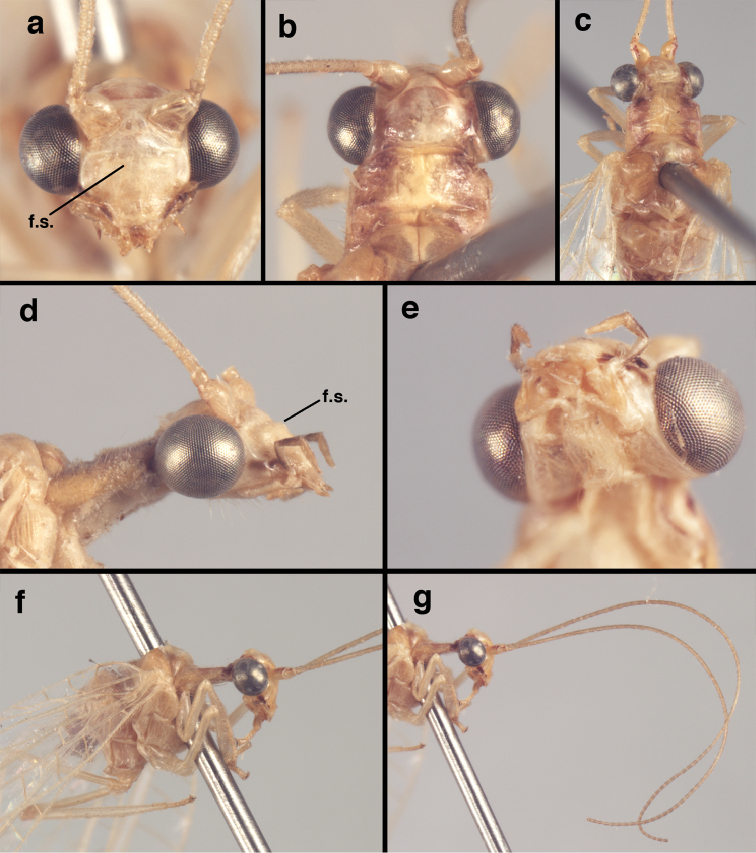
*Ungla curimaguensis* Sosa: External features, (**a**) head, frontal (**b**) head, prothorax, dorsal (**c**) head, thorax, dorsal (**d**) head, prothorax, lateral (**e**) head, ventral (**f**) head, thorax, lateral (**g**) head, antenna, lateral. **f.s.** frontal swelling (all: Venezuela, Aragua, Rancho Grande; **a, b, d, e** male, USNM **c, f, g** female, USNM).

**Figure 22.**
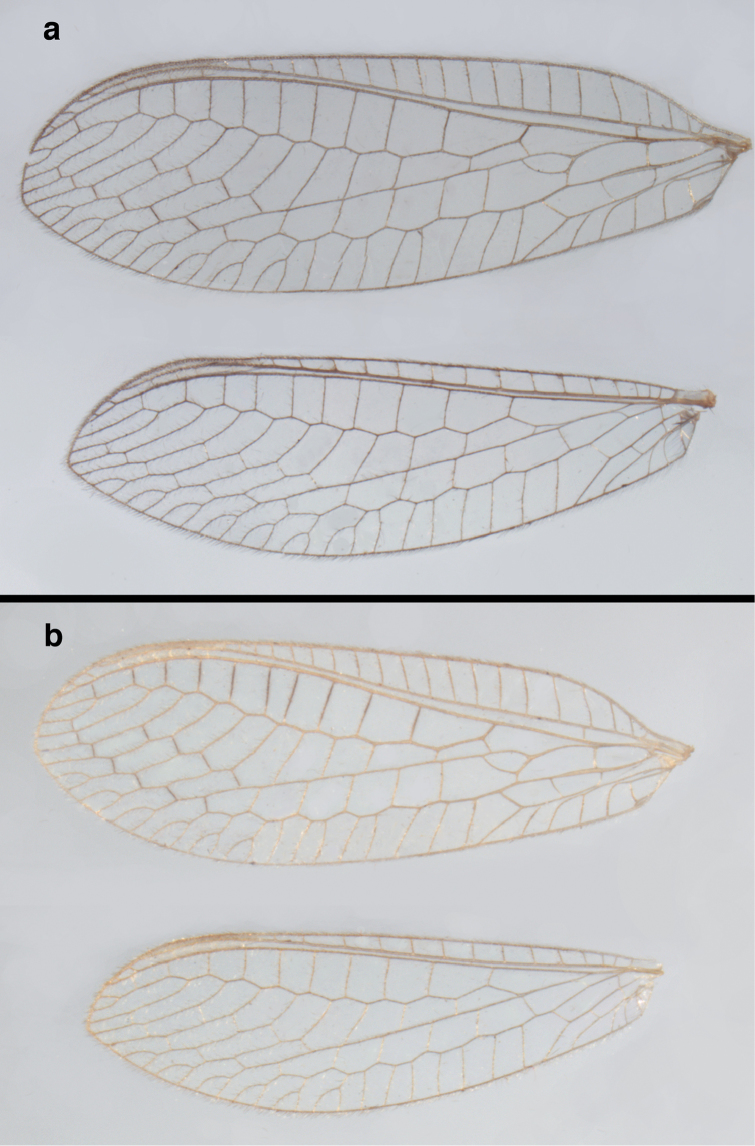
*Ungla curimaguensis* Sosa: Wings, (**a**) female (**b**) male (**a, b** Venezuela, Aragua, Rancho Grande, USNM).

**Figure 23.**
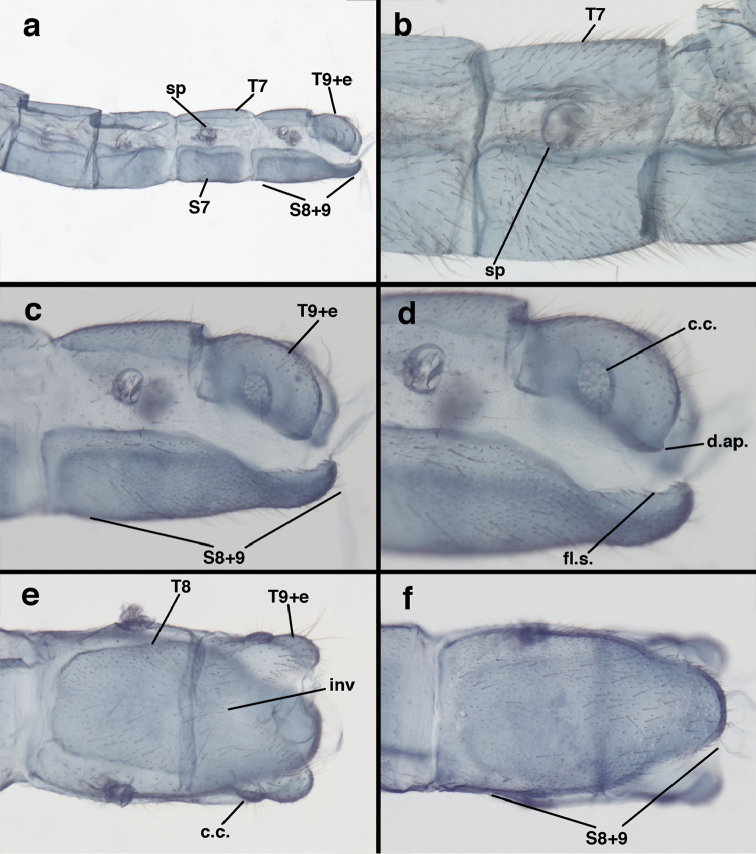
*Ungla curimaguensis* Sosa: Mature male abdomen, (**a**) segments A5-terminus, lateral (**b**) segment A7, lateral (**c**) segments A8 and A9, lateral (**d**) terminus, lateral (**e**) eighth tergite and fused ninth tergite+ectoproct, dorsal (**f**) sternite 8+9, ventral. **c.c.** callus cerci **d.ap.** dorsal apodeme **fl.s.** flanged setae **inv** dorsal invagination of T9+ectoproct **sp** spiracle **T7, T8** seventh, eighth tergites **T9+e** fused ninth tergite and ectoproct **S7** seventh sternite **S8+9** fused eighth and ninth sternites (**a, c, d, e, f** Venezuela, Aragua, Rancho Grande, USNM; **b** same, AMNH).

**Figure 24.**
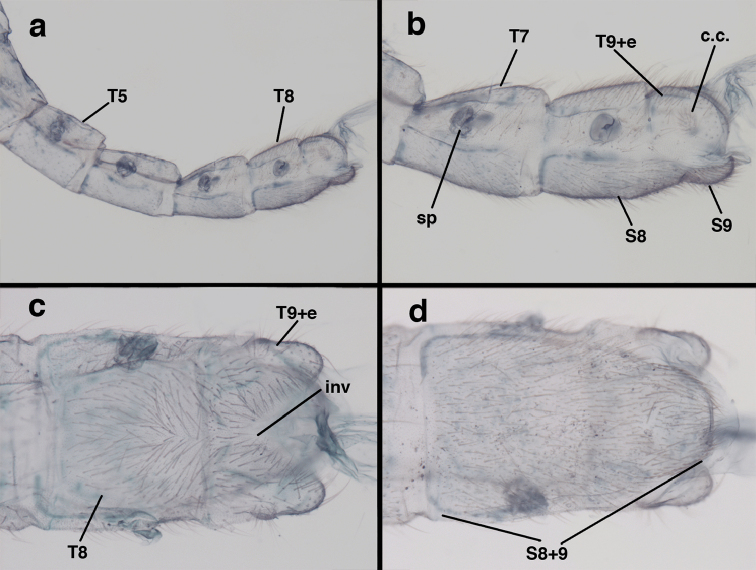
*Ungla curimaguensis* Sosa: Slightly teneral male abdomen, (**a**) segments A5-terminus, lateral (**b**) segments A7-terminus, lateral (**c**) eighth tergite and fused ninth tergite+ectoproct, dorsal (**d**) fused sternites 8+9, ventral. **c.c.** callus cerci, **inv** dorsal invagination of T9+ectoproct **sp** spiracle **T5, T7, T8** fifth, seventh, eighth tergites **T9+e** fused ninth tergite and ectoproct **S8, S9** eighth, ninth sternites (all: Venezuela, Mérida, EMUS).

**Figure 25.**
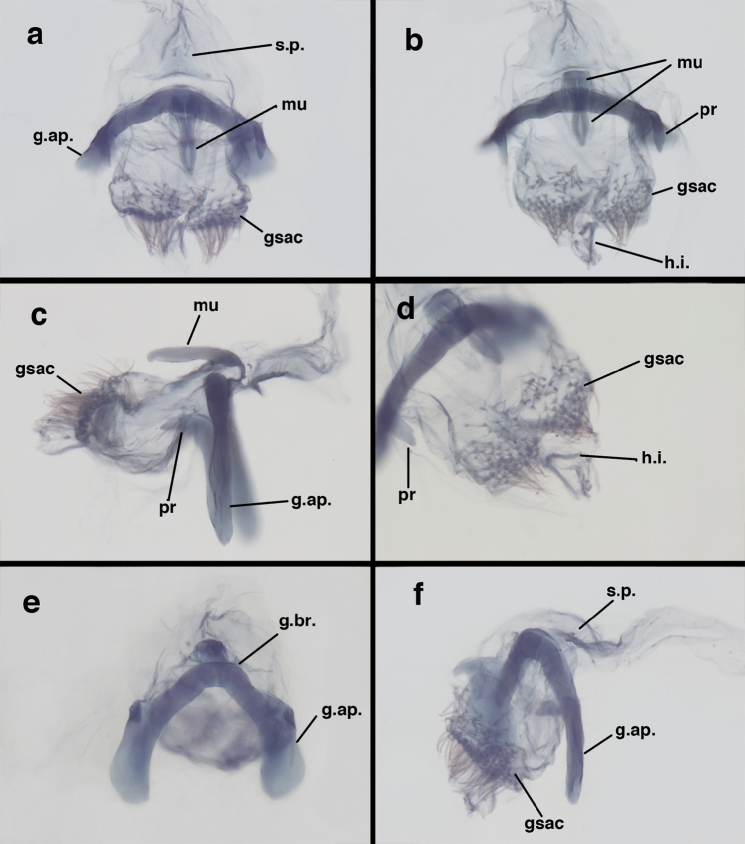
*Ungla curimaguensis* Sosa: Male genitalia, (**a, b**) gonarcal complex, dorsofrontal, with two slightly different positions (**c**) gonarcal complex, lateral (**d**) gonarcus (partial), dorsolateral, with hypandrium internum at base of gonosaccus (**e**) gonarcus, posterior (**f**) gonarcus, posterolateral. **gsac** gonosaccus **g.ap.** gonarcal apodeme **g.br.** gonarcal bridge **h.i.** hypandrium internum **mu** mediuncus **pr** unarticulated process on frontal margin of gonarcal apodeme **s.p.** setose subanal plate (all: Venezuela, Aragua, Rancho Grande, USNM).

Figs 21 , 22 , 23 , 24 , 25 , 143a

Ungla curimaguensis Sosa, 2015. *Zootaxa* 4018 (2): 183–186. “VENEZUELA. *Falcón state*: Curimagua, 11°11'N/69°38'W, 1040 m, 24–26.i.2014, F. Sosa, F. Díaz & R. Paz Legs. Collected with light trap. Deposited in the MJMO.” **Holotype.**MJMO, male. For images of the holotype (a fresh specimen) see Sosa (2015); for labels, see Fig. 143a here.

##### Diagnosis.

Adults of this species are pale green, with a dorsal yellow stripe extending from the head through the abdomen; they have the following distinctive head and wing features. The frons is cream-colored or pale yellow; the genae and lateral margins of the clypeus have a black stripe; the scape is pale green with a red dorsolateral stripe extending from the tip to the base or almost to the base. The inverted U-shaped mark on the vertex that typifies most species of *Ungla* is reduced to small red markings on and around the vertex and fossae. The forewing has pale green veins; however, the gradate veins, which are parallel, are reddish brown to brown, with light suffusion of brown on the surrounding membrane. In males, most of the longitudinal veins of the forewing are crassate, and the abdominal spiracles are enlarged, but the dorsomesal margins of the abdominal sternites are only slightly indented. [Note: On many pinned specimens, the head markings are faded or discolored and the costal and radial crossveins are either partially or completely darkened to light brown (compare Fig. 8 in Sosa 2015 with Figs 21, 22 here.)]

##### Redescription.

Head cream-colored to yellowish, with vertex smooth, shiny; inverted U-shaped marking on vertex faint, reddish, when visible, separated mesally, not extending anteriorly to antennal fossae or scapes, broader posteriorly than anteriorly; area between eyes and vertex with reddish marks or coloration; frons unmarked, flat in female, broad (frontal view) and raised mesally (lateral view) in male; gena with long, brown stripe extending from near base of eyes continuing through basolateral part of clypeal margin; tentorial pits amber to pale. Antenna cream-colored to yellow, dorsum of scape with diffuse reddish, longitudinal stripe laterally, flagellum with pale bristles; maxillary palp with basal two segments pale, distal three segments dark brown to black laterally; labial palp with basal two segments pale, distal one with light brown at tip.

Thorax mostly green (living specimens) to cream-colored (preserved specimens). Prothorax flat, with broad, red to reddish brown stripes laterally, light cream-colored mesally; transverse furrow relatively deep, in middle region of segment, ending laterally in broad, whitish spot on lateral stripe; setae on dorsal, lateral surfaces long, slender, yellow to reddish, dense laterally. Mesothorax, metathorax with broad band of reddish to reddish brown laterally. Legs pale, cream-colored, without marks. Measurements: head width: 1.3 mm; ratio head width : eye width: 2.4–2.6 : 1; prothorax width: 1.0–1.1 mm, length: 0.6–0.7 mm.

Forewing, hindwing slender; forewing rounded apically, hindwing broadly acute; membrane clear, without fumose areas except around gradates. Forewing with veins crassate (male), without thickening (female); stigma transparent to slightly opaque, with 4 (female) to 6 (male) subcostal crossveins below, without marks; longitudinal veins, crossveins mostly light green, pinned specimens (male) with radial crossveins slightly darkened; gradate veins reddish brown, with some suffusion on surrounding membrane. Hindwing venation light green. Forewing 10.4–12.0 mm long, 3.5–3.9 mm wide (ratio, L : W = 2.9–3.1 : 1), height of tallest costal cell 0.8 mm (cell number 6–7); length of first intramedian cell 0.7–0.8 mm; 10 radial cells (closed cells between R and Rs); 4 Banksian cells (b cells), 4 b’ cells; 6 inner gradates, 6 outer gradates. Hindwing 9.5–10.7 mm long, 2.9–3.2 mm wide (ratio, L : W = 3.3 : 1), 10 radial cells, 3 Banksian (b) cells, 4 b’ cells, 3–4 inner gradates, 5–6 outer gradates.

Male: Abdomen with enlarged spiracles (e.g., A7: spiracle diameter ~0.23–0.26× length of sternite); abdominal sternites with small to no dorsal depression; subanal plate large, triangular, with ~twelve midsized setae; T9+ectoproct dome-shaped, with dorsal invagination shallow, extending approximately one half distance to anterior margin of T9, lateral margins of invagination slightly convex, base U-shaped; dorsal margin of ectoproct rounded throughout, with posteroventral margin extended distomesally in husky, rounded knob; ventral margin sclerotized posteriorly to base of callus cerci; callus cerci oblong, with ~ 30 robust microtrichia; circumference sclerotized heavily on posterior, anterior, ventral margins, lightly on dorsal margin; sclerotization contiguous with that on ventral margin of ectoproct, with narrow band of light sclerotization extending dorsally for very short distance from posterodorsal margin of callus cerci. S8+9 fused, with line of fusion not demarcated, but with slight fold on teneral specimen; S9 narrowed, considerably more heavily sclerotized than S8; dorsal margin relatively well sclerotized, including basally; terminus up-turned posteriorly, extending distally well beyond T9+ect, with distal setae slightly enlarged, with row of heavy, flanged setae along dorsodistal margin. Gonarcus flattened, narrow in lateral view, thick in frontal, posterior views; bridge straight for short distance mesally, then arms extending downward at sharp angle; arms very slender in lateral view, broad, with rounded tips in posterior, frontal views, with digitiform process extending forward, not inward from distal margin of apodeme; process with wide base, tapering to acute tip distally; mediuncus long, narrow, straight dorsally (lateral view), rounded, with broad hook distally; bilobed gonosaccus, each lobe with single, large patch of heavy gonosetae facing mesally when unexpanded; dorsal setae smaller than ventral ones, all arising from bulbous setal bases; hypandrium internum attached closely to base of gonosaccus, robust, broadly V-shaped, with lightly sclerotized, hooked comes.

Female: See Sosa (2015).

##### Variation.

The coloration of the body, markings, and wing venation deteriorates quickly and substantially after death.

##### Known distribution.

VENEZUELA: States of Aragua, Falcón, Mérida, Táchira.

##### Specimens examined

[in addition to those listed by Sosa (2015)]. Venezuela. *Aragua*: Rancho Grande, 11/VI/1945 (1M, AMNH); Rancho Grande, 1100m, 11–15/I/1966, S. S. & W. D. Duckworth (1M, 2F, USNM), 16–19/I/1966, S. S. & W. D. Duckworth (2F, USNM), 21-25/I/1966, S. S. & W. D. Duckworth (2M, 2F, USNM), 10–21/II/1969, Duckworth & Dietz (1F, USNM). *Mérida*: Mérida, 1950 m, 13-IX-1973, B. Villegas (1F, UCDC); Mérida, 17-21-V-1996, W. C. Pitt (1M, EMUS).

#### Ungla demarmelsi

Taxon classificationAnimaliaNeuropteraChrysopidae

Sosa, 2015

**Figure 26.**
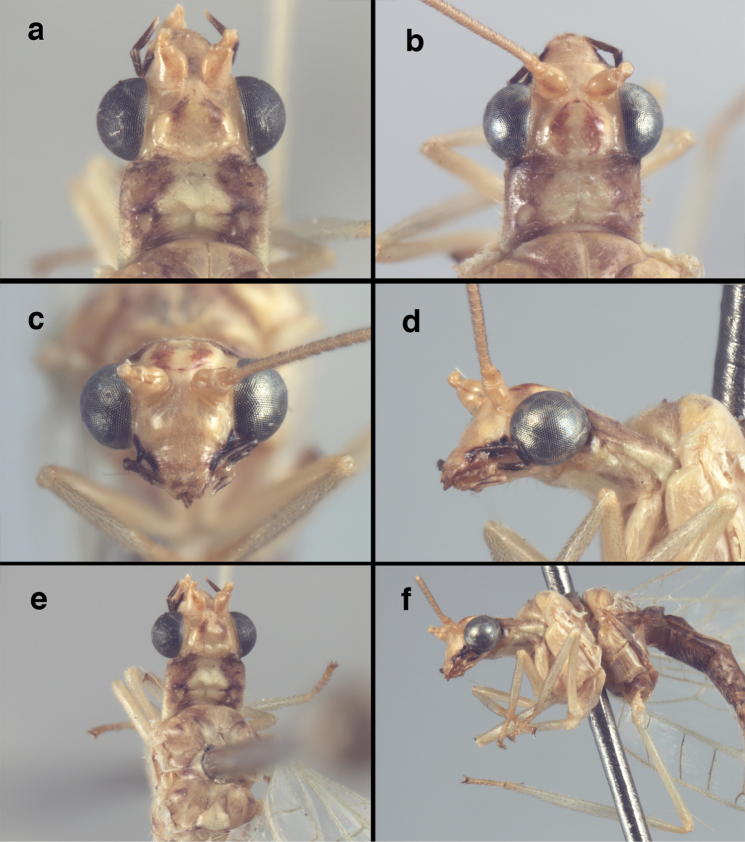
*Ungla demarmelsi* Sosa: External features, (**a, b**) head, prothorax, dorsal (**c**) head, frontal (**d**) head, prothorax, lateral (**e**) head, thorax, dorsal (**f**) body, lateral (all: Venezuela, Distrito Capital, USNM; **a, c–f** male; **b** female).

**Figure 27.**
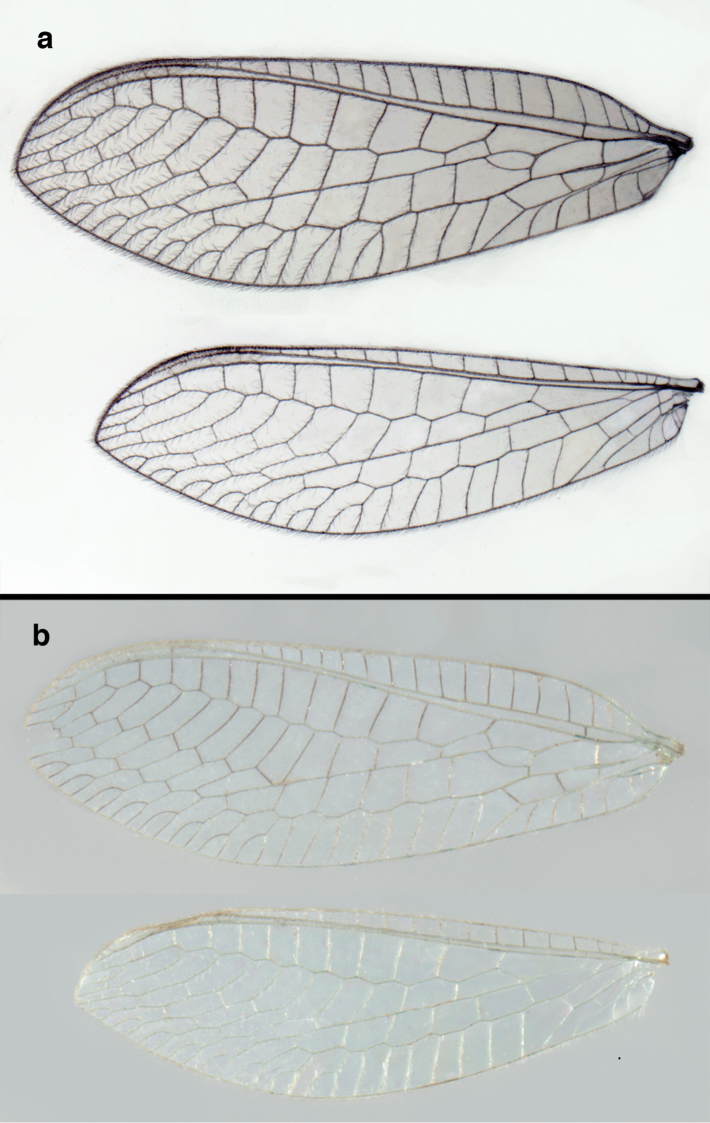
*Ungla demarmelsi* Sosa: Wings, (**a**) venation emphasized (**b**) coloration of veins emphasized (**a** Venezuela, Distrito Capital, male, USNM; **b** Venezuela, Trujillo, female, UCDC).

**Figure 28.**
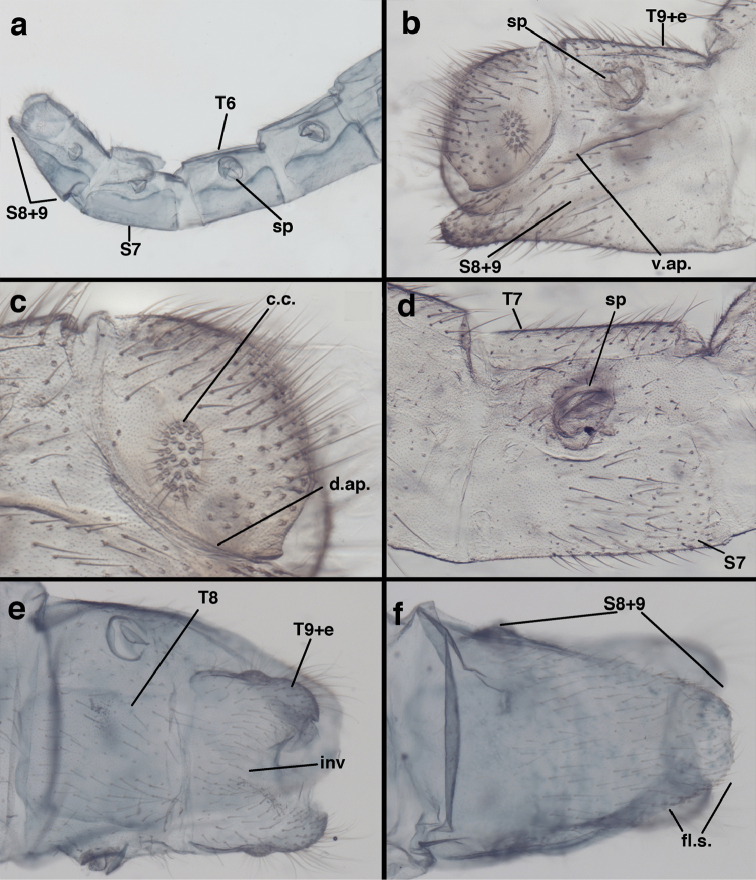
*Ungla demarmelsi* Sosa: Male abdomen, (**a**) segments A5-terminus, lateral (**b**) terminus, lateral (**c**) fused ninth tergite+ectoproct, lateral (**d**) seventh segment, lateral (**e**) eighth tergite and fused ninth tergite+ectoproct, dorsal (**f**) sternite 8+9, ventral. **c.c.** callus cerci **d.ap.** dorsal apodeme **fl.s.** flanged setae **inv** dorsal invagination of T9+ectoproct **sp** spiracle **T6, T7, T8** sixth, seventh, and eighth tergites **T9+e** fused ninth tergite and ectoproct **S7** seventh sternite **S8+9** fused eighth and ninth sternites **v.ap.** ventral apodeme (**a, e, f** Venezuela, Mérida, UCDC; **b, c, d** Venezuela, Distrito Capital, USNM).

**Figure 29.**
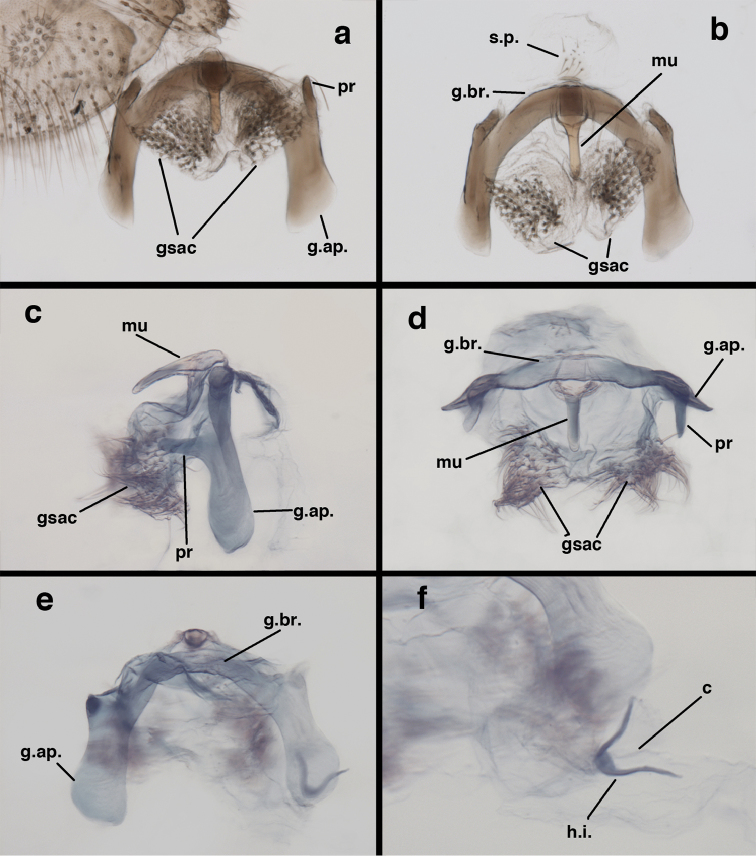
*Ungla demarmelsi* Sosa: Male genitalia, (**a**) gonarcus, frontal, gonosaccus inverted (**b**) gonarcus, frontal, gonosaccus everted (**c**) gonarcus, lateral (**d**) gonarcus, dorsal (**e**) gonarcus, posterior, with hypandrium internum caught beneath apodeme on right (**f**) hypandrium internum. **c** comes **gsac** gonosaccus **g.ap.** gonarcal apodeme **g.br.** gonarcal bridge **h.i.** hypandrium internum **mu** mediuncus **pr** unarticulated process on frontal margin of gonarcal apodeme **s.p.** setose subanal plate (**a, b** Venezuela, Distrito Capital, USNM; **c–f** Venezuela, Mérida, UCDC).

**Figure 30.**
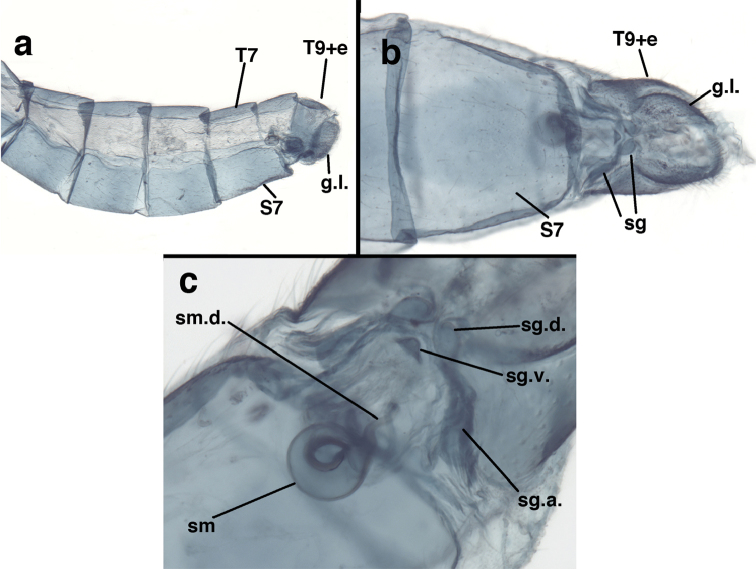
*Ungla demarmelsi* Sosa: Female abdomen and genitalia, (**a**) segments A4-terminus, lateral (**b**) terminus, ventral (**c**) genitalia, ventral. **g.l.** gonapophysis lateralis **sg** subgenitale **sg.a.** arm of subgenitale **sg.d.** dorsal lobe of subgenitale **sg.v**. ventral lobe of subgenitale **sm** spermatheca **sm.d.** spermathecal duct **S7** seventh sternite **T7** seventh tergite **T9+e** fused ninth tergite and ectoproct (all: Venezuela, Trujillo, UCDC).

Figs 26 , 27 , 28 , 29 , 30 , 143b

Ungla demarmelsi Sosa, 2015. *Zootaxa* 4018 (2): 177-180; “VENEZUELA. *Mérida state*. Mérida city. La Hechicera, [8.627491°N/71.162393°W)], 1900 m, 19.i.1988, D. Diez Leg. Deposited in the MIZA.” **Holotype.**MIZA, male. For images of the type see Sosa (2015); for labels, see Fig. 143b here.

##### Diagnosis.

*Ungla demarmelsi* is the only known *Ungla* species that has the following set of features: a yellow head with raised vertex bearing diffuse to dark reddish, inverted U-shaped mark, scape with red mark dorsoapically, pronotum with broad, red, lateral stripes, and mesoscutum marked with red laterally. Males have enlarged spiracles, and the dorsal margins of the sternites are clearly concave in both sexes. The subanal plate is small; it bears about ten rather small setae. The tip of the male S8+9 is concave and extends beyond T9+ect; its terminus bears enlarged setae on robust bases; approximately ten pairs of these setae are also flanged basally above the setal base. The gonarcal bridge is round and smoothly arched throughout.

##### Redescription.

Head cream-colored to yellow, with vertex smooth, shiny; inverted U-shaped marking on vertex, light to dark red, narrow to separated mesally, not extending anteriorly to antennal fossae or scapes, broader posteriorly; area between eyes and vertex unmarked; frons unmarked; gena with long, black stripe extending from base of eyes continuing through lateral margin of clypeus; tentorial pits amber to light brown marginally. Antenna cream-colored to yellow, dorsum of scape with diffuse reddish, longitudinal stripe, flagellum with light brown to amber bristles; maxillary palp with basal two segments pale, distal three segments dark brown to black; labial palp with basal two segments pale, distal one with dark brown.

Thorax mostly green (living specimens) to cream-colored (preserved specimens). Prothorax short, flat, with broad, red to reddish brown, lateral stripes; transverse furrow shallow in middle region, with yellowish spots at lateral margins; setae on dorsal, lateral surfaces long, slender, yellow to reddish, dense laterally. Mesothorax, metathorax with reddish to reddish brown laterally. Legs pale cream-colored, without marks. Measurements: head width: 1.3–1.4 mm; ratio head width : eye width: 2.2 : 1; prothorax width: 1.0–1.1 mm, length: 0.4–0.5 mm.

Forewing rounded apically; hindwing broadly acute; membrane clear, without fumose areas. Forewing with veins uninflated, except base of Cu (male and female); stigma transparent, tinged lightly with brown, with five subcostal crossveins below without marks; longitudinal veins mostly light green; transverse veins green with reddish tinge, crossveins red to light reddish brown. Hindwing venation light green. Forewing 11.7–14.4 mm long, 3.9–4.8 mm wide (ratio, L : W = 2.9–3.0 : 1), height of tallest costal cell 0.7–0.9 mm (cell number 5); length of first intramedian cell 0.8–1.0 mm; 10–11 radial cells (closed cells between R and Rs); 4 Banksian cells (b cells), 4 b’ cells; 5–7 inner gradates, 6–8 outer gradates. Hindwing 10.5–12.9 mm long, 3.1-4.1 mm wide (ratio, L : W = 3.2-3.3 : 1), 10-11 radial cells, 3 Banksian (b) cells, 4 b’ cells, 4-5 inner gradates, 6-7 outer gradates.

Male: Abdomen with large spiracles (e.g., A7: spiracle diameter ~0.24× length of sternite); subanal plate rectangular, with ~twelve robust setae; T9+ectoproct short, with dorsal invagination shallow, extending approximately one half the distance to anterior margin of T9, lateral margins of invagination fairly straight; dorsal margin of segment slightly rounded, with posteroventral margin extended distally in husky, rounded knob; ventral margin heavily sclerotized well beyond callus cerci; callus cerci large, oblong, with ~ 30 robust microtrichia; circumference lightly sclerotized, sclerotization separate from that on ventral margin of ectoproct, without dorsal extension. S8+9 fused, with no line of demarcation; S9 considerably more heavily sclerotized than S8; dorsal margin sclerotized mesally, lightly so basally; terminus rounded, blunt, not up-turned posteriorly, extending distally well beyond T9+ect, with distal setae enlarged, those along dorsodistal margin flanged. Gonarcus broad, smooth, U-shaped (posterior view), bridge thin, apodemes robust (lateral view), turned (thus appearing narrow in posterior view); apodemes rounded to slightly quadrate distally, with process, slender digitiform, rounded distally, extending outward from frontal margin of apodeme, sometimes bending slightly inward; mediuncus elongate, very slightly curved downward, narrow throughout (dorsal view), slightly rounded dorsally (lateral view); bilobed gonosaccus, each lobe with single, large patch of robust gonosetae; gonosetae arising from bulbous setal bases, dorsal setae approximately same size as ventral ones; hypandrium internum robust, broadly U-shaped, with outward-bent arms, very lightly sclerotized, curved comes.

Female. See Fig. 30 here, and Sosa (2015).

##### Variation.

The darkness and size of the head markings on the vertex vary among specimens.

##### Known distribution.

VENEZUELA (northeastern): States of Aragua, Mérida, Trujillo.

##### Specimens examined

[in addition to those listed by Sosa (2015)]. Venezuela. *Aragua*: 14km. N. Colonia Tovar 1750 m, 21-25/I/1983, O. S. Flint, Jr. (1M, 3F, USNM). *Mérida*: Libertador, 3/VII/1979, R. W. Brooks, A. A. Grigarick, J. McLauchlin, R. O. Schuster (2M, 1F, UCDC); Mérida, 17-21/V/1996, W. C. Pitt (1M, EMUS). *Trujillo*: La Mesa, 11/IX/1973, B. Villegas (5F, UCDC).

#### Ungla diazi

Taxon classificationAnimaliaNeuropteraChrysopidae

Sosa, 2015

**Figure 31.**
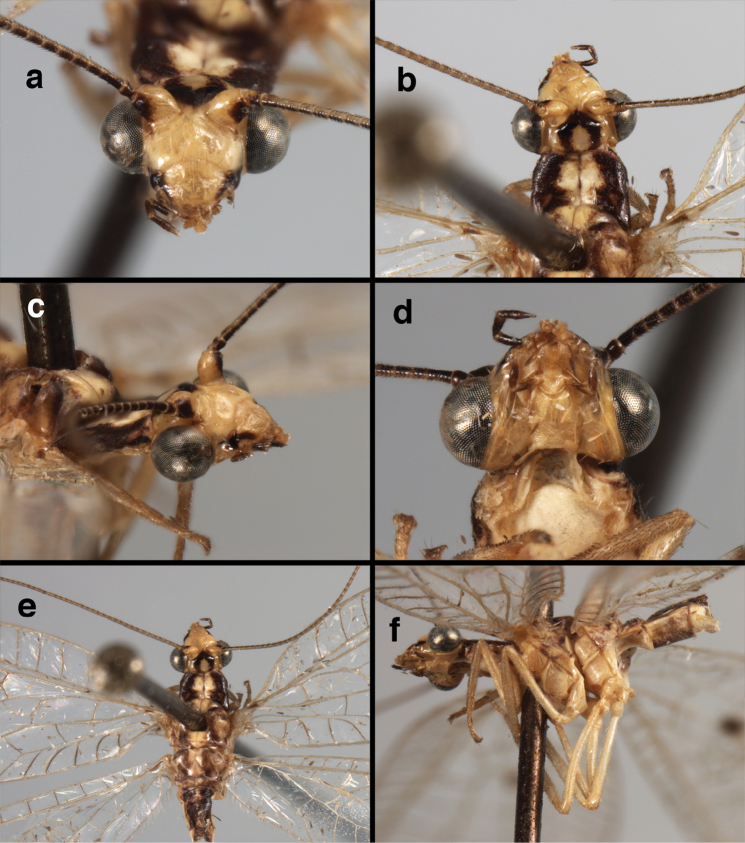
*Ungla diazi* Sosa: External features, (**a**) head, frontal (**b**) head, prothorax, dorsal (**c**) head, frontolateral (**d**) head, ventral (**e**) head, thorax, dorsal (**f**) head, thorax, ventrolateral (all: Bolivia, Cochabamba, male, CAS).

**Figure 32.**
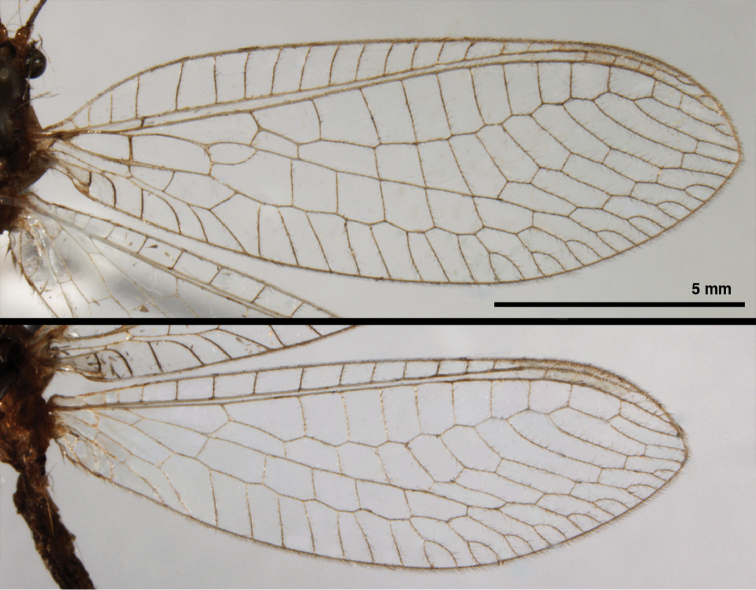
*Ungla diazi* Sosa: Wings (Bolivia, Cochabamba, male, CAS).

**Figure 33.**
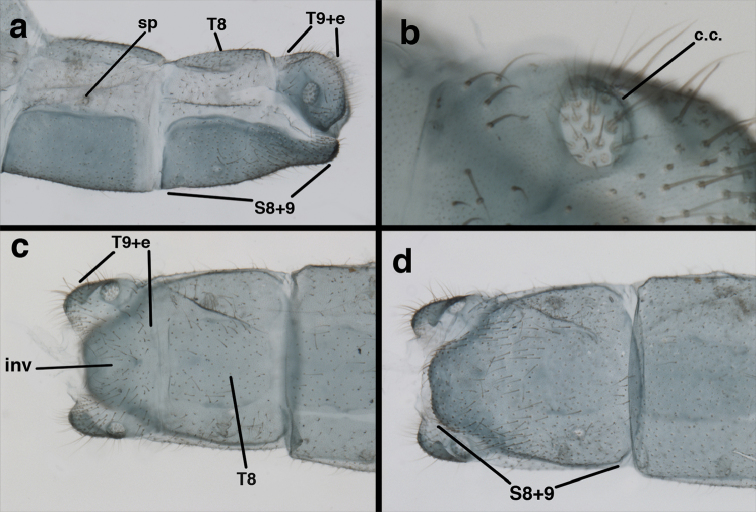
*Ungla diazi* Sosa: Male abdomen, (**a**) segments A7-terminus, lateral (**b**) callus cerci (**c**) eighth tergite and ninth tergite + ectoproct, dorsal (**d**) fused eighth and ninth sternites, ventral. **c.c.** callus cerci **inv** invaginated dorsal cleft in T9+ectoproct **sp** spiracle **S8+9** fused eighth and ninth sternites **T8** eighth tergite **T9+e** fused ninth tergite and ectoproct (all: Bolivia, Cochabamba, CAS).

**Figure 34.**
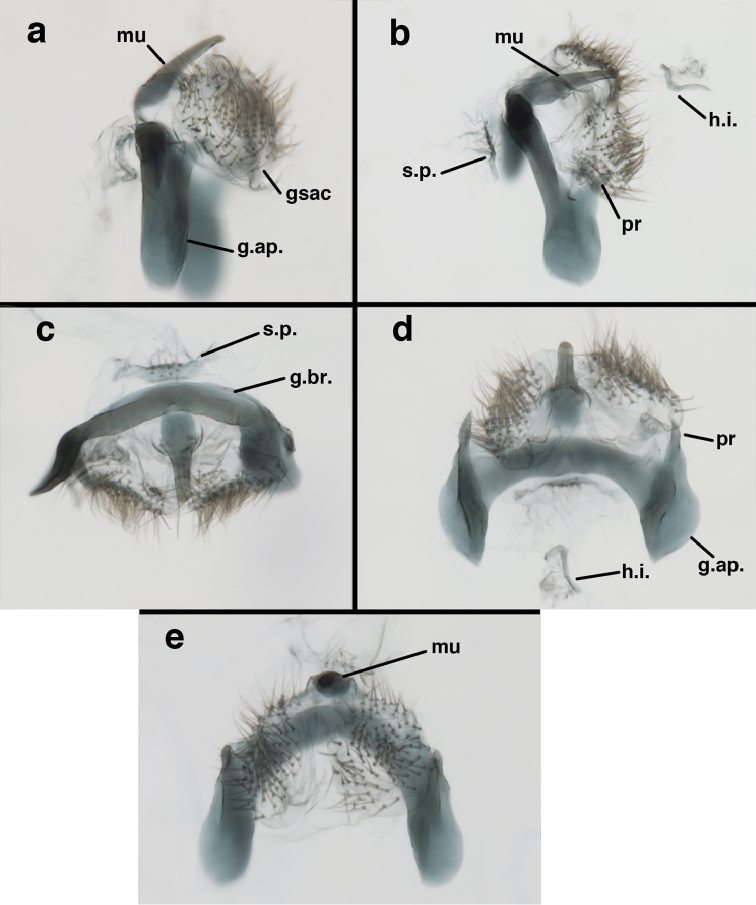
*Ungla diazi* Sosa: Male genitalia, (**a**) gonarcus, lateral (**b**) gonarcus, dorsolateral (**c**) gonarcus, dorsal (**d**) gonarcus, ventral (**e**) gonarcus, frontal. **g.ap.** gonarcal apodeme **g.br.** gonarcal bridge **gsac** gonosaccus **h.i.** hypandrium internum **mu** mediuncus **pr** unarticulated process on frontal margin of gonarcal apodeme **s.p.** setose subanal plate (all: Bolivia, Cochabamba, CAS).

Figs 31 , 32 , 33 , 34 , 143c

Ungla diazi Sosa, 2015. *Zootaxa* 4018 (2): 180–183; “VENEZUELA. *Lara state*: P. N. [Parque Nacional] Yacambú, El Blanquito, 1463 m, 9.70649°N/69.57608°W, 28.iv–4.v.2003, J. Clavijo, R. Briceño, A. Chacón & Q. Árias Leg. [project S1-2000000479]. Deposited in the MIZA.” **Holotype.**MIZA, male. For images of the type see Sosa (2015); for labels, see Fig. 143c here.

##### Diagnosis.

*Ungla diazi* is typified by (i) a yellow flagellum marked with black ventrolaterally, (ii) scape unmarked dorsally, except sometimes with a small to large mark distolaterally on the ventral surface, and (iii) gena cream-colored to golden below the eyes, with a black spot on the pleurostomal margin. The spiracles on the male abdomen are not enlarged, and sternites S4–S6 are densely covered with microsetae.

The marking on the distolateral corner of the ventral surface of the scape varies from absent (some Venezuelan specimens) to large, dark and shiny (Bolivian specimens). Indeed the dorsal head markings of the Bolivian population are so dark and shiny that they appear similar to *U. banksi* and *U. quchapampa*. Hand-written labels on some specimens of *U. diazi* from Bolivia indicate that Adams too thought the species was related to *U. banksi* (then known as *Nothochrysa tibialis*). In addition to the traits above, *U. diazi* adults can be distinguished from *U. banksi* by their more elongate wings and lighter brown veins, largely lacking suffusion. They can be differentiated from *U. quchapampa* by their lack of a large marking on the frons.

##### Redescription.

Head golden to cream-colored, with vertex smooth, shiny; curved marking on vertex dark brown to black, large, prominent, broadly connected mesally, extending anteriorly to antennal fossae, but not between scapes; area between eyes and vertex unmarked; frons unmarked; gena with brown mark distally, not touching eyes, continuing along lateral margin of clypeus, but not reaching tip; tentorial pits with light brown margins. Antenna with scape golden to cream-colored, ventral surface with large, dark brown to black mark distolaterally; pedicel with brown ring; flagellum cream-colored to light tan dorsally, dark brown to black ventrally; maxillary palp with basal two segments pale, distal three segments dark brown; labial palp with basal segment pale, distal two segments with dark brown.

Prothorax cream-colored mesally, with wide, shiny, dark brown, lateral stripes, very thin, brown mesal line; transverse furrow shallow, in posterior region, ending laterally in slightly lightened circular area within dark lateral stripe; dorsal surface apparently with waxy covering, with fine pale setae dorsally, darker, heavier setae laterally. Mesothorax, metathorax cream-colored to tan mesally, marked with light brown to brown laterally. Legs pale, cream-colored, without marks. Measurements: head width: 1.4–1.5 mm; ratio head width : eye width: 2.5–2.7 : 1; prothorax width: 1.1–1.2 mm, length: 0.5–0.7 mm.

Forewing, hindwing rounded apically; membrane clear, largely without fumose areas. Forewing with veins uninflated, except basal section of Psc slightly crassate (male & female); stigma lightly opaque, with four to five, tan to brown subcostal crossveins below stigma, small area surrounding crossveins sometimes marked with brown; longitudinal veins mostly light tan; transverse veins slightly darker, especially costal, distal radial crossveins, anal veins, all usually without suffusion, occasionally light suffusion around anal veins. Hindwing venation pale. Forewing 12.1–14.7 mm long, 4.1–5.1 mm wide (ratio, L : W = 2.9–3.0 : 1), height of tallest costal cell 0.8–1.1 mm (cell number 5–6); length of first intramedian cell 0.8–1.0 mm; 10–11 radial cells (closed cells between R and Rs); 4 Banksian cells (b cells), 4 b’ cells; 4–6 inner gradates, 6–7 outer gradates. Hindwing 10.7–13.1 mm long, 3.3–4.4 mm wide (ratio, L : W = 3.0–3.1 : 1), 10–11 radial cells, 3–4 Banksian (b) cells, 3–4 b’ cells, 3–6 inner gradates, 5–7 outer gradates.

Male. Abdomen with unenlarged spiracles (e.g., A7: spiracle diameter ~0.05x length of sternite); subanal plate large, with ~ten robust setae; T9+ectoproct short, with dorsal invagination shallow, extending approximately one half the distance to anterior margin of T9, lateral margins of invagination slightly convex; dorsal margin of segment rounded distally, with posteroventral margin extended distally in husky, rounded knob; ventral margin sclerotized well beyond callus cerci; callus cerci large, oblong, with ~ 30–36 robust microtrichia; circumference sclerotized, sclerotization contiguous with that on ventral margin of ectoproct, with narrow band of light sclerotization extending dorsally from dorsal margin of callus cerci to top of T9+ect. S8+9 fused, with line of fusion well demarcated; S9 considerably more heavily sclerotized than S8; dorsal margin lightly sclerotized, including basally; terminus up-turned posteriorly, not extending distally far beyond T9+ect, with setae slightly enlarged, but not flanged. Gonarcus with bridge robust, very slightly angled mesally, arms slender in lateral view, flared, with digitiform process slender; mediuncus long, narrow, slightly rounded dorsally (lateral view); bilobed gonosaccus, each lobe with single, large patch of large gonosetae probably facing mesally when unexpanded; gonosetae arising from bulbous setal bases, dorsal ones slightly smaller than ventral ones; hypandrium internum robust, broadly V-shaped, with lightly sclerotized, U-shaped comes.

Female. See Sosa (2015).

##### Variation.

The darkness and sizes of the head and appendage markings on this species show considerable variability. Dorsal head markings range from faint, diffuse to dark, shiny brown or black. The basal flagellomeres range from entirely pale, to light brown on ventral and lateral surfaces only, to shiny dark brown or black on all surfaces.

##### Known distribution.

BOLIVIA: Department of Cochabamba. VENEZUELA: States of Aragua, Lara.

##### Specimens examined

[in addition to those listed by Sosa (2015)]. Bolivia. Cochabamba Carrasco, Siberia, 1850 m, F. Walz, XII/1962 (3F, CAS), XII/1962-I/1963 (11F, 1M, CAS), X/1963 (1M, 9F, CAS). Venezuela. *Aragua*: Rancho Grande, 1100m, 24–31/X/1966, S. S. & W. D. Duckworth (2M, 2F, USNM), 14/VI/1967, 25/VI/1967, 4/VI/1967, 5/VII/1967, 1/VIII/1967, 5/VIII/1967, 15/VIII/1967, R. W. Poole (1M, 5F, CUIC), 10–21/II/1969, Duckworth & Dietz (3F, USNM); Henri Pittier Nat. Park, Rancho Grande Bio. Station, white light, 1100 m, 24/I/1996, J. & A. Skevington (1F, DEBU).

#### Ungla favrei

Taxon classificationAnimaliaNeuropteraChrysopidae

(Navás, 1935)

Figs 35 , 36 , 37 , 38 , 39 , 40 , 41 , 42

**Figure 35.**
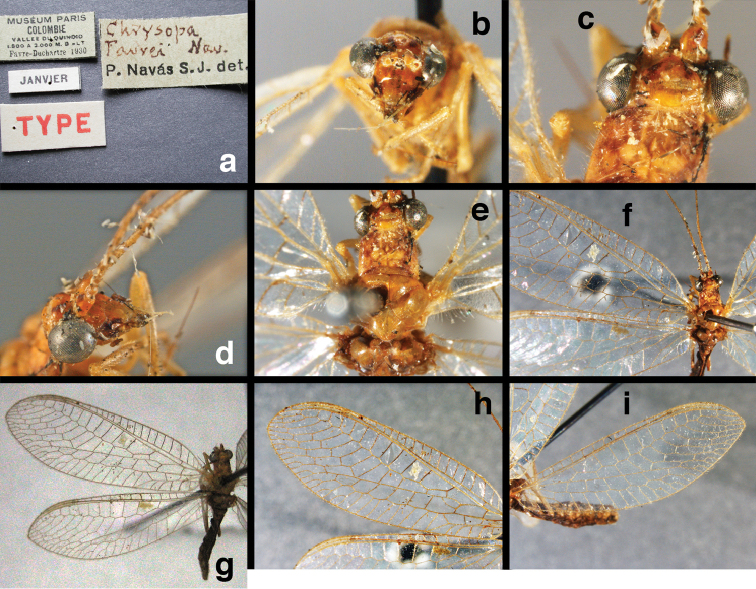
*Chrysopa favrei* Navás: External features, (**a**) labels (**b**) head, frontal (**c**) head, prothorax, dorsal (**d**) head, frontolateral (**e**) head, thorax, dorsal (**f**) body, dorsolateral (**g**) body, left wings (**h**) forewing (**i**) hindwing (all: Colombia, Valle de Quindio, lectotype, female, MNHN).

**Figure 36.**
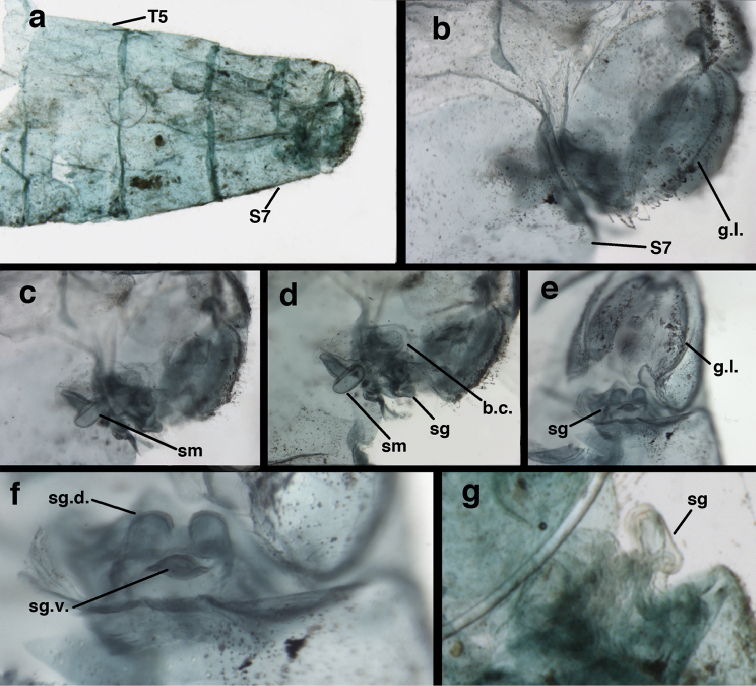
*Chrysopa favrei* Navás: Female abdomen, (**a**) segments A5-A10, lateral (**b**) terminalia, exterior, lateral (**c, d**) genitalia, interior, lateral (**e**) gonapophysis lateralis, subgenitale, posterior (**f**) subgenitale, posterior (**g**) subgenitale, lateral. **b.c.** bursa copulatrix **g.l.** gonapophysis lateralis **S7** seventh sternite **sg** subgenitale **sg.d.** dorsal lobe of subgenitale **sg.v**. ventral lobe of subgenitale **sm** spermatheca **T5** fifth tergite (all: Colombia, Valle de Quindio, lectotype, MNHN).

**Figure 37.**
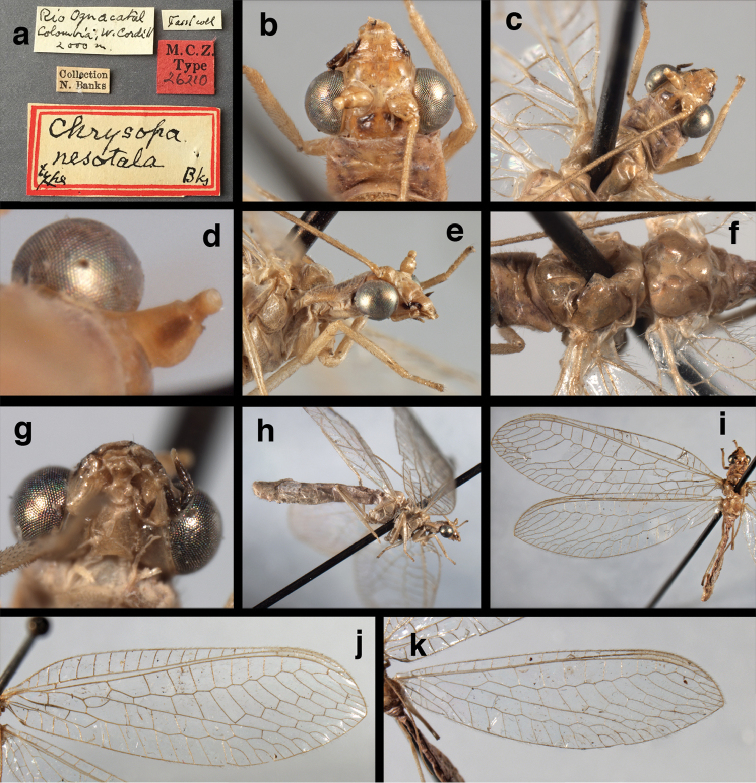
*Chrysopa nesotala* Banks: External features, (**a**) labels (**b**) head, frontodorsal (**c**) head, prothorax, dorsal (**d**) scape, dorsal (**e**) head, prothorax, lateral (**f**) thorax, dorsal (**g**) head, ventral (**h**) body, lateral (**i**) body, left wings (**j**) right forewing (**k**) right hindwing (Colombia, Rio Ognacatal, holotype, female, MCZ).

Chrysopa favrei Navás, 1935. *Rev. R. Acad. Cienc. exactas fis. nat. Madrid* 32: 363–364, fig. 57; “Colombie: Vallée de Quindio, 1.500 à 2.000m. d’alt. Favre-Duchartre, 1930, janvier. Mus. de París”. Penny 1977: 17 (list); Brooks and Barnard 1990: 279 (list, as “ ‘Chrysopa’ incertae sedis”); Oswald 2015 (catalog). Ungla favrei (Navás) by Legrand et al. 2008: 136 (tax); Oswald 2015 (catalog). **Lectotype** (Figs 35, 36). MNHN, female (examined); lectotype designated by Legrand et al. (2008: 136). The type locality is probably in the Cocora Valley (1800–2400 m, Department of Quindio) in the Central Cordillera of the Andean mountains.
Chrysopa nesotala Banks, 1944. *Bol. Entomol. Venezolana* 3: 16; “Rio Ognacatal, Western Cordillera, Colombia, 2.000 meters (Fassl coll.). Type M.C.Z. N° 26.210”. Penny 1977: 19 (list); Oswald 2015 (catalog). Suarius nesotala (Banks), by Adams and Penny 1985: 436 (tax. disc.). Ungla nesotala (Banks), by Brooks and Barnard 1990: 279, Freitas 2007: 415 (key to adults); Oswald 2015 (catalog). **syn. n. Holotype** (Fig. 37). MCZ, female (examined); holotype by original designation. Banks referred to a single specimen; thus it is the holotype. We could not find a “Rio Ognacatal” in South America; we assume that the term refers to Río Aguacatal, a stream in Valle del Cauca Department, Colombia (located between El Centenario and Barrio Terrón Colorado. Rio Aguacatal is in the Western Cordillera of the Andes at an elevation of approximately 2,000 meters. **Support for synonymy.** The types of both species are females and discolored with age. A large series of specimens (CAS, see below) collected in Cochabamba Department, Bolivia, allowed us to associate males and females of the species and to identify diagnostic external and male genitalic characters that confirm the synonymy.

##### Diagnosis.

This species is distinguished from other Andean *Ungla* species by a brown, inverted U-shaped mark that is broken mesally, a white to cream-colored face, frons usually with brown mesal spot of variable size and darkness, antenna cream-colored, with longitudinal brown mark on the distal, upper surface of the scape that extends onto the pedicel, and wings with pale longitudinal veins and numerous brown crossveins. The male abdomen has moderately enlarged spiracles and dense setation; the gonarcal bridge has a ledge that extends forward and receives the base of the mediuncus.

Among all *Ungla* species, *U. favrei* appears most similar to *U. elbergi* sp. n., both externally and in some male abdominal features (e.g., the moderately enlarged spiracles (A7: ~0.15× length of sternite). However, its male abdominal segments are taller in height and shorter in length than those of *U. elbergi*, and its genital structures (gonarcus, mediuncus and gonosaccus) differ in shape. It also resembles *U. grandispiracula* sp. n., which can be differentiated by its larger spiracles (A7: 0.25× length of sternite), more robust gonosetae, gonarcal bridge without an enlarged mesal platform, and scape with brown dorsal mark that does not reach the pedicel.

##### Redescription.

Body color: brown to brownish, sometimes with yellow mesally. Head cream-colored, with vertex smooth, often shiny; inverted U-shaped marking on vertex brown to reddish brown, usually prominent, narrowing and sometimes separated mesally, not extending anteriorly to area between scapes; antennal fossa, area between eyes and posterior half of vertex unmarked; frons often with brown, small to large, triangular marking centrally; gena with broad, brown stripe extending from eye along lateral margin of gena, clypeus; tentorial pits amber-colored. Antenna pale, dorsum of scape with brown longitudinal stripe distally, extending onto dorsal surface of pedicel; maxillary palp, labial palp with basal two segments pale, distal segments dark brown.

Prothorax yellowish mesally, with broad, brown to reddish brown, longitudinal, submesal stripes almost reaching lateral margin; transverse furrow in posterior region, not reaching lateral margins; dorsal surface with thin, pale setae, sparse mesally, denser laterally. Mesothorax, metathorax brown to yellowish brown laterally, yellow mesally. Measurements: head width: 1.2–1.3 mm; ratio head width : eye width: 2.4 : 1; prothorax width, 1.0 mm; length: 0.5–0.6 mm.

Forewing, hindwing narrow, rounded apically; membrane clear, hyaline, without fumose areas, with slender venation; stigma lightly opaque to clear, with four to five light to dark brown subcostal crossveins below stigma, area surrounding crossveins unmarked; longitudinal veins mostly pale, slightly darker at intersections, base of Rs, distal parts of Psc, anal veins darker; transverse veins mostly pale, with costal crossveins slightly dark near subcosta, intracubital crossveins, first gradate veins, distal veinlets slightly darker, without suffusion. First gradate vein meets Psm very near intersection of transverse vein. Forewing 11.3–13.4 mm long, 3.7–4.6 mm wide (ratio, L : W = 2.9–3.1 : 1); height of tallest costal cell 0.9 mm (cell number 4–6); length of first intramedian cell 0.8–0.9 mm; 10–11 radial cells (closed cells between R and Rs); 4 Banksian cells (b cells), 4 b’ cells; 4–6 inner gradates, 5–7 outer gradates. Hindwing with venation pale, 10.1–14.3 mm long, 3.1–4.7 mm wide (ratio, L : W = 3.1–3.2 : 1), 10–11 radial cells, 3 Banksian (b) cells, 4 b’ cells, 4–5 inner gradates, 5–6 outer gradates.

**Figure 38.**
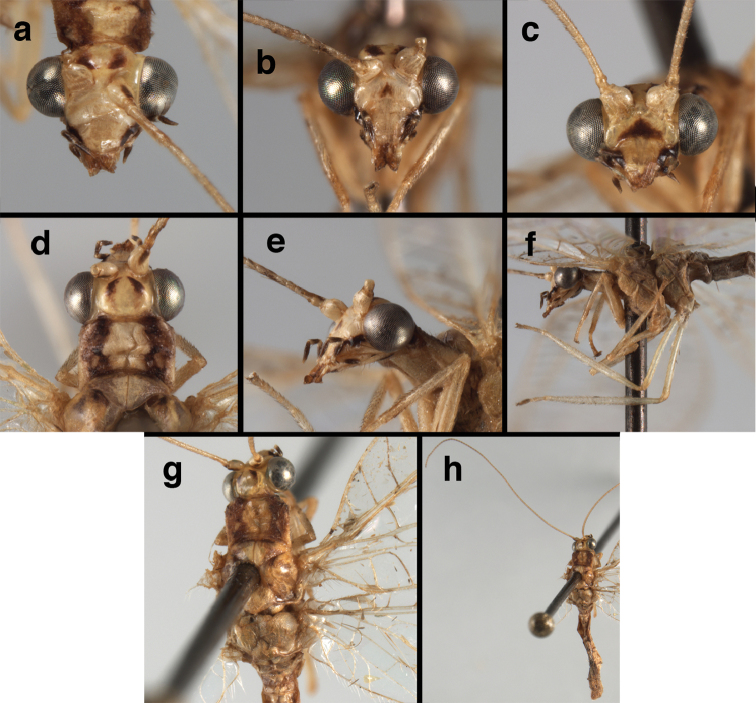
*Ungla favrei* (Navás): External features, (**a, b, c**) head, frontal [Note variation in markings.] (**d**) head, prothorax, anterior mesothorax, dorsal (**e**) head, lateral (**f**) thorax, lateral (**g**) head, thorax, dorsal (**h**) body, dorsal (all: Bolivia, Cochabamba, CAS; **a** male; **b–h** female).

**Figure 39.**
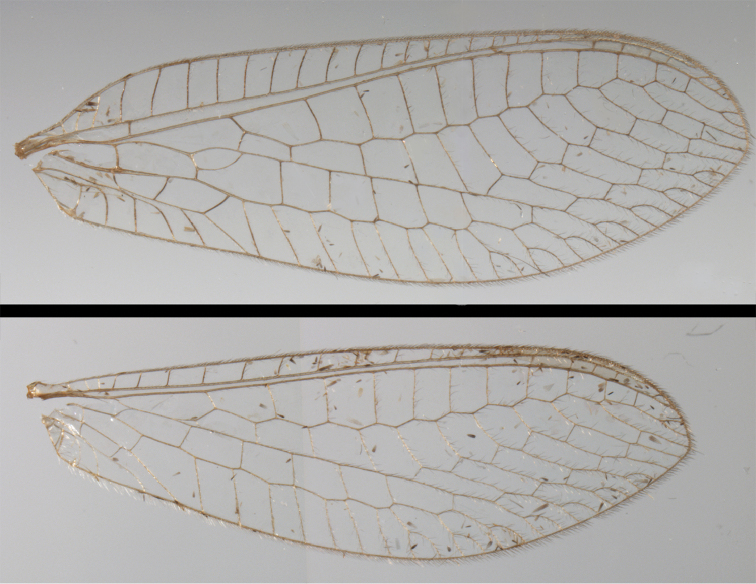
*Ungla favrei* (Navás): Wings (Bolivia, Cochabamba, female, CAS).

Male. Abdomen with long and short setae, especially dense posteriorly, on A7–A9; spiracles moderately enlarged (e.g., A7: spiracle diameter ~0.15x length of sternite). T9+ectoproct rounded, sloping ventrally (lateral view), with dorsal invagination rounded, shallow (extending approximately one half distance to anterior margin of T9), posteroventral knob well defined, heavily sclerotized, bending mesally; ventral margin lightly sclerotized beyond callus cerci; callus cerci large, ovate, circumference lightly sclerotized; subrectal plate bearing field of ~15 short setae. S8+9 fused, with line of fusion not demarcated; dorsal margin sclerotized, especially basal 2/3rds; terminus rounded, extending distally only slightly beyond the tip of T9+ect; terminal setae dense, enlarged, mostly simple, except for series of ~10–16 flanged setae on both sides of upper margin. Gonarcus (frontal view) broad, rounded throughout, flat; dorsal margin extending forward toward base of mediuncus, as short, ledge-like protuberance; lateral margins of the gonarcal apodemes with short, rounded lateral process extending forward. Mediuncus broad, thick basally (at attachment to the gonarcal bridge), becoming narrow distally, length ~1.8× height of gonarcus (measured in lateral view), distal portion arching downward from base, terminus with slightly enlarged knob. Gonosaccus large, robust, with two large pouches each bearing large field of robust, elongate, slightly curved gonosetae arising from large sockets (bases). Hypandrium internum narrow, U-shaped, with irregularly shaped comes.

##### Variation.

Among the specimens we examined, there was considerable variation in the size and degree of separation of the dorsal head markings, in the presence or absence and size of a frontal marking, and the darkness and amount of brown coloration on the wing veins.

##### Known distribution.

BOLIVIA (central): Department of Cochabamba. COLOMBIA (west to central): Departments of Cundinamarca, Quindio, Valle del Cauca.

##### Specimens examined

(in addition to types above). Bolivia. *Cochabamba*: Corrosco Siberia, 1650 m, XII/1962 – I/1963, purchase F. Walz (7M, 14F, CAS), I/1964, F. Walz (1F, CAS), X/1983 (1M, SDMC), 1850 m, X/1963, F. Walz (1M, 5F, CAS). Colombia. *Cundinamarca*: Monterredondo, 1420 m, XII/1958 (1F, CAS), II/1959 (1F, CAS).

**Figure 40.**
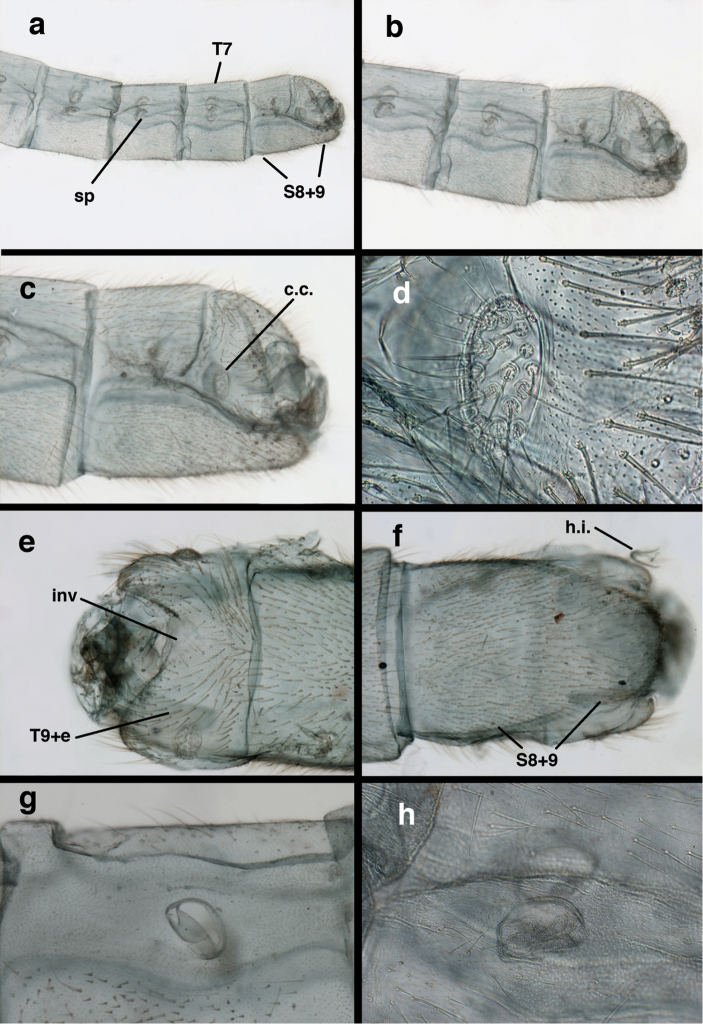
*Ungla favrei* (Navás): Male abdomen, (**a**) segments A5-terminus, lateral (**b**) segments A7-terminus, lateral (**c**) segment A9+ectoproct, lateral (**d**) callus cerci (**e**) tergite 9+ectoproct, dorsal, with gonarcus inside [Note deep mesal invagination of posterior margin.] (**f**) sternite 8+9, ventral, with large, flanged setae on distal margin (**g, h**) enlarged spiracle (A6). **c.c.** callus cerci **inv.** invagination of T9+ectoproct,dorsal surface **h.i.** hypandrium internum **sp** spiracle **S8+9** fused eighth and ninth sternites **T7** seventh tergite **T9+e** ninth tergite + ectoproct (Bolivia, Cochabamba, CAS).

**Figure 41.**
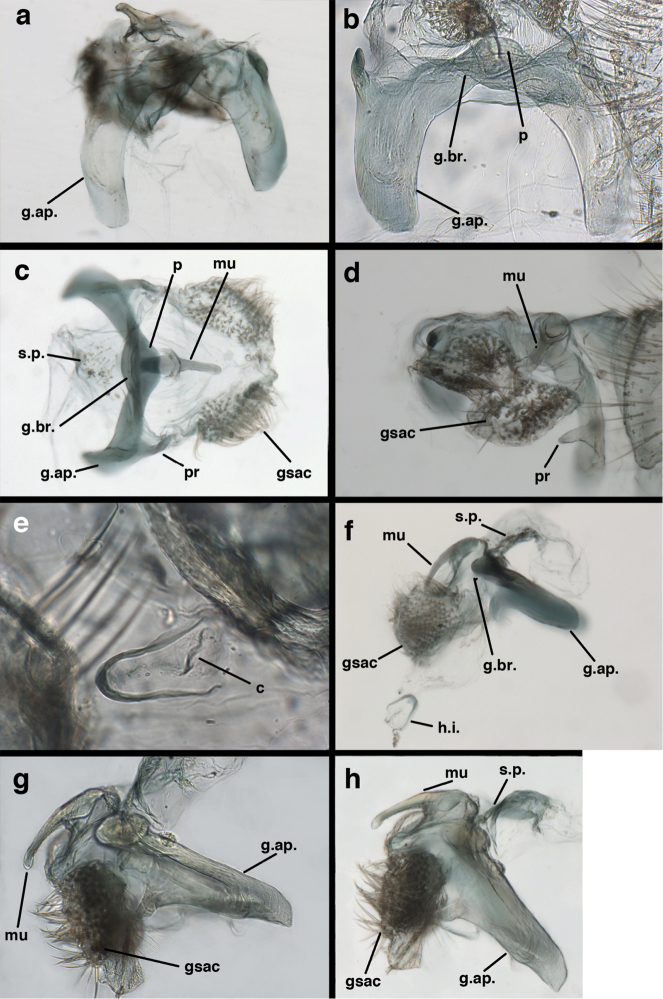
*Ungla favrei* (Navás): Male genitalia, (**a**) gonarcal complex, posterior, somewhat flattened (**b**) gonarcus, posterior (**c**) gonarcal complex, dorsal, gonosaccus fully expanded (**d**) gonarcal complex, frontal, tilted, gonosaccus partially expanded (**e**) hypandrium internum (**f**) gonarcal complex, lateral, gonosaccus fully expanded (**g, h**) gonarcal complex, lateral, gonosaccus partially expanded. **c** comes **gsac** gonosaccus **g.ap.** gonarcal apodeme **g.br.** gonarcal bridge **h.i.** hypandrium internum **mu** mediuncus **p** platform on mesal section of gonarcal bridge **pr** unarticulated process on frontal margin of gonarcal apodeme **s.p.** setose subanal plate (Bolivia, Cochabamba, CAS).

**Figure 42.**
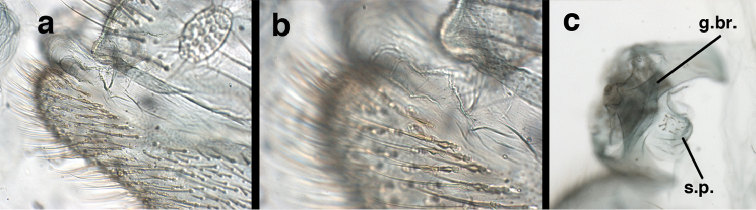
*Ungla favrei* (Navás): Male terminalia, (**a, b**) tip of segment A9, lateral, showing patch of enlarged, flanged setae on lateral margin of S9 and large, but unmodified setae on tip of S9 (**c**) subanal plate above gonarcal bridge. **g.br.** gonarcal bridge **s.p.** setose subanal plate (Bolivia, Cochabamba, CAS).

#### Ungla grandispiracula

Taxon classificationAnimaliaNeuropteraChrysopidae

Tauber sp. n.

http://zoobank.org/C33FBC0C-31C4-4EBA-B599-822EFB4DC851

**Figure 43.**
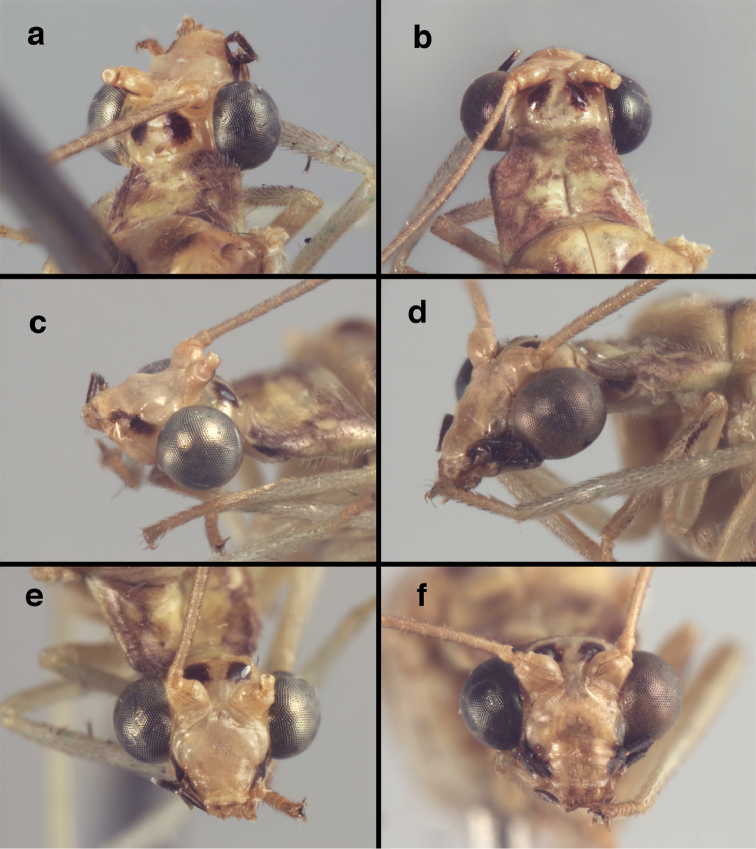
*Ungla grandispiracula* Tauber, sp. n. External features, (**a, b**) head, prothorax, dorsal (**c, d**) head, frontolateral (**e, f**) head, frontal (all: Colombia, Antioquia, USNM; **a, c, e** male, holotype; **b, d, f** female, paratype).

**Figure 44.**
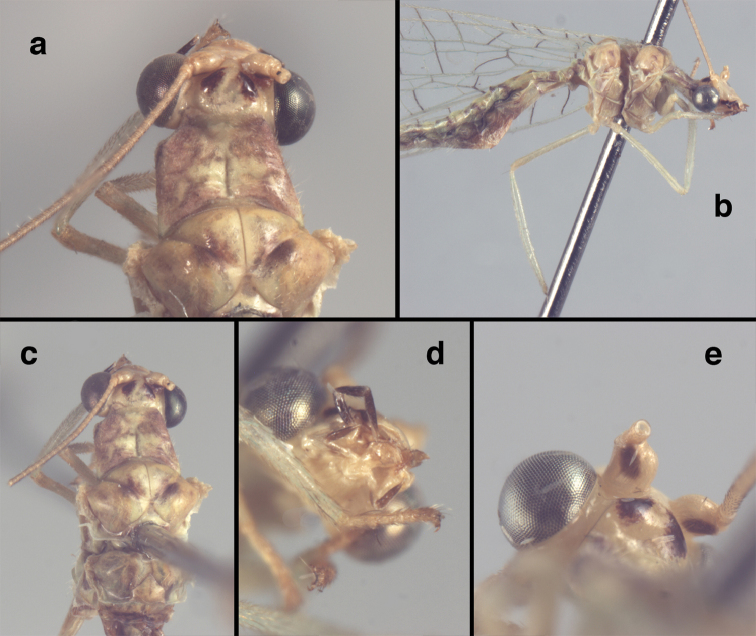
*Ungla grandispiracula* Tauber, sp. n. External features, (**a**) head, prothorax, mesothorax, dorsal (**b**) thorax, lateral (**c**) thorax, dorsal (**d**) head, ventral (**e**) scapes, dorsal (all: Colombia, Antioquia, USNM; **a, c** female, paratype; **b, d, e** male, holotype).

**Figure 45.**
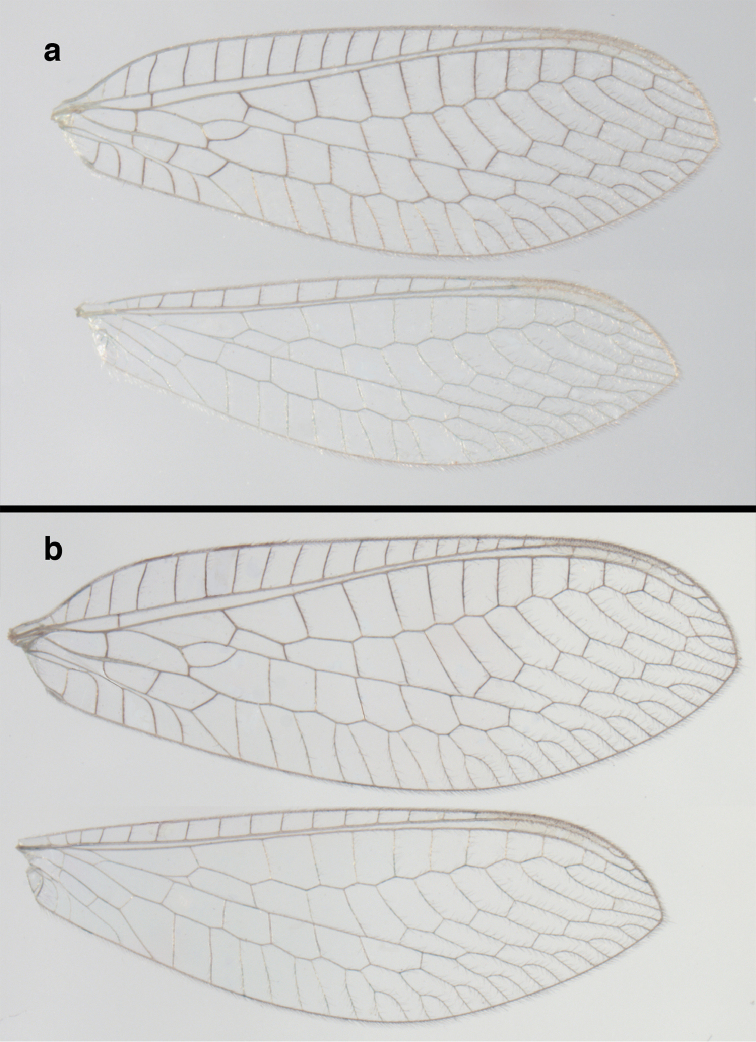
*Ungla grandispiracula* Tauber, sp. n. Wings, (**a**) male, holotype (**b**) female, paratype (all: Colombia, Antioquia, USNM).

**Figure 46.**
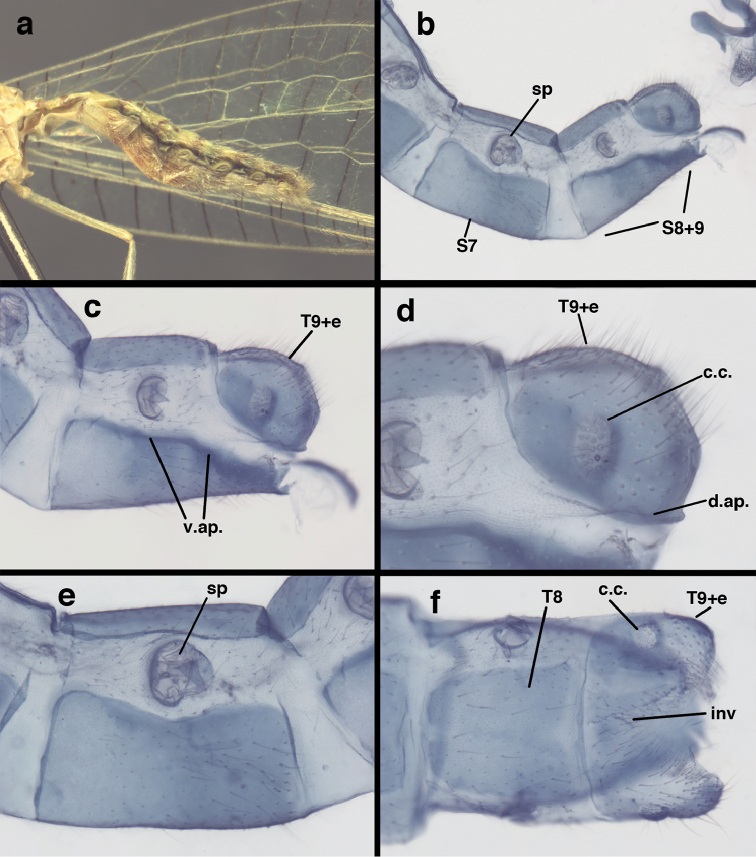
*Ungla grandispiracula* Tauber, sp. n. Male abdomen, (**a**) exterior, lateral (**b**) segments A7-terminus, lateral (**c**) terminal segments, lateral (**d**) tergite 9+ectoproct, lateral (**e**) seventh segment, lateral, with enlarged spiracle (**f**) tergite 9+ectoproct, dorsal. **c.c.** callus cerci **d.ap.** dorsal apodeme **inv** dorsal invagination of T9+ectoproct **sp** spiracle **S7** seventh sternite **S8+9** fused eighth and ninth sternites **T8** eighth tergite **T9+e** ninth tergite + ectoproct **v.ap.** ventral apodeme (**a** Colombia, Antioquia, holotype, USNM; **b–f** Colombia, Valle del Cauca, paratype, FSCA).

**Figure 47.**
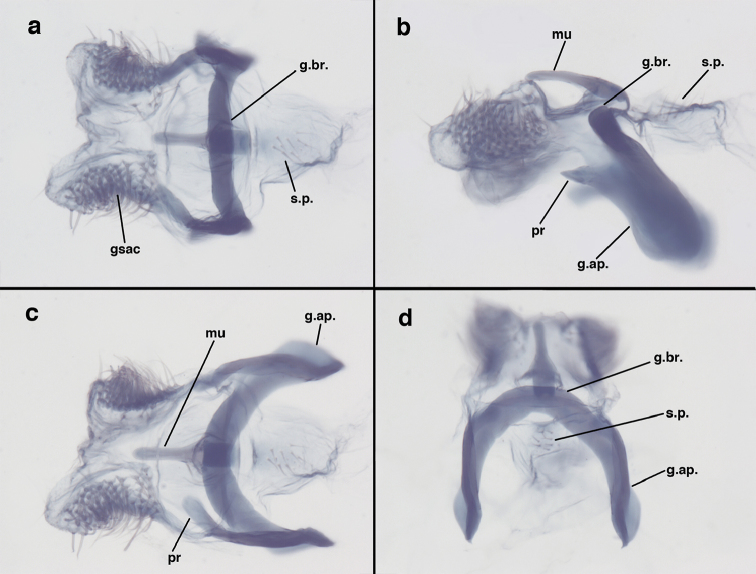
*Ungla grandispiracula* Tauber, sp. n. Male genitalia, (**a**) gonarcal complex, dorsofrontal, (**b**) gonarcus, lateral (**c**) gonarcal complex, dorsal, gonosaccus fully expanded (**d**) gonarcal complex, posterior. **gsac** gonosaccus **g.ap.** gonarcal apodeme **g.br.** gonarcal bridge **mu** mediuncus **pr** unarticulated process on frontal margin of gonarcal apodeme **s.p.** setose subanal plate (all: Colombia, Valle del Cauca, paratype, FSCA).

Figs 43 , 44 , 45 , 46 , 47 , 144b

##### Holotype

(Figs 43a, c, e, 44b, d, e, 45a, 144b). USNM, male. Colombia, Antioquia, 12 km. NW Medellín, rd to San Pedro, 15 Feb. 1983, O. S. Flint, Jr.

##### Etymology.

The species name “*grandispiracula*” (Latin, neuter, plural) refers to the large spiracles that distinguish males of this species from those of *U. favrei*, another Andean species of *Ungla* with which it shares many features. The word is a compound noun in apposition to the genus name (*grandis*, meaning “large”; *spiracula*, meaning “spiracles”).

##### Diagnosis.

The Andean species *U. grandispiracula* and *U. favrei* are very similar externally and in many of their male abdominal features. Both have a brown or red, inverted U-shaped mark that is broken mesally, a white to cream-colored face, cream-colored antenna with longitudinal brown mark on the distal, upper surface of the scape that extends onto the pedicel, and wings with pale longitudinal veins and numerous brown crossveins. They also have enlarged abdominal spiracles and similar genitalia. However, there are subtle differences in the male abdomen that distiguish the two species: the *U. grandispiracula* spiracles are larger (A7: 0.25x length of S7 versus 0.15x in *U. favrei*), its gonosetae are more robust, and its gonarcal bridge is narrow, uniformly rounded, and it lacks the mesal ledge that occurs in *U. favrei*. Females of the two species are difficult to separate; in *U. grandispiracula* the frons is unmarked (variable in *U. favrei*) and the stripe on the dorsal surface of the scape does not extend onto the pedicel as it does in *U. favrei*.

Externally, this species also resembles the Argentinian *U. elbergi*, sp. n. However, the *U. grandispiracula* spiracles are larger than those of *U. elbergi*; and, unlike on *U. elbergi*, the two lobes of the gonosaccus are well separated mesally, and the gonosetae are borne laterally, on somewhat flattened plates.

##### Description.

Head white to cream-colored with dark brown to black markings; vertex smooth, often shiny; inverted U-shaped marking dark brown, prominent but small, dot-like, narrowing and separated mesally, not extending anteriorly to area between scapes; antennal fossa, area between eyes and posterior half of vertex unmarked; frons unmarked, slightly swollen laterally in males; gena with dark brown to black stripe extending from near base of eye along lateral margin of gena, most of clypeus; tentorial pits amber-colored. Antenna pale, dorsum of scape with short, brown longitudinal stripe distally, not extending onto dorsal surface of pedicel; maxillary palp with basal two segments pale, three distal segments dark brown; labial palp with basal segment pale, middle segment light brown, distal segment dark brown.

Prothorax yellowish mesally, with broad, diffuse, reddish brown, longitudinal, lateral stripes, extending to lateral margin; transverse furrow in mesal region, almost reaching lateral margins; dorsal surface with thin, pale setae, sparse mesally, denser laterally. Mesothorax, metathorax marked with reddish brown laterally, yellow mesally; both with pair of brown spots on margin between prescutum and scutum (smaller on metathorax), pair of small brownish spots laterally. Measurements: head width: 1.5 mm; ratio head width : eye width: 2.3 : 1; prothorax width: 1.0 mm; length: 0.5 mm.

Forewing with apex rounded, hindwing acute; membrane clear, hyaline, without fumose areas, with venation slender (female) to very slightly crassate (male); stigma lightly opaque to clear, with three to four light brown subcostal crossveins below stigma, area surrounding crossveins unmarked; longitudinal veins light green, all costal, radial crossveins brown to brownish; transverse veins in posterior sector of wing brown to pale; gradates dark brown without suffusion. First gradate vein meeting Psm. Forewing 12.6–13.7 mm long, 4.3–4.8 mm wide (ratio, L : W = 2.9 : 1); height of tallest costal cell 0.7–0.8 mm (cell number 5–6); length of first intramedian cell 0.9–1.0 mm; 10–11 radial cells (closed cells between R and Rs); 4 Banksian cells (b cells), 4 b’ cells; 4–6 inner gradates, 6 outer gradates. Hindwing 11.4–12.3 mm long, 3.5–4.0 mm wide (ratio, L : W = 3.1–3.2 : 1), 10–11 radial cells, 3 Banksian (b) cells, 4 b’ cells, 3–4 inner gradates, 6 outer gradates.

Male. T9+ect relatively long (~0.5 length of T7), with dorsal invagination moderately deep (~0.5× dorsal length of T9+ect), margins of invagination almost straight, base rounded; dorsal margin of T9+ect straight basally, rounded distally, posterior margin of ectoproct convex, posteriorly with dorsal apodeme prominent, but without knob or extension. Abdomen with setae more or less of a single size (no short setae), relatively sparse on A7-A9; spiracles greatly enlarged (e.g., A7: spiracle diameter ~0.25× length of sternite). T9+ect fairly well rounded throughout, terminating distoventrally at small distal extension of dorsal apodeme, with dorsal invagination rounded, not shallow (deeper than one half distance to anterior margin of T9); area anterior to, below, and around callus cerci diffusely sclerotized, with sclerotization melding with dorsal apodeme along ventral and posteroventral margin of ectoproct; callus cerci large, ovate, circumference sclerotized throughout, but lightly dorsally; subrectal plate narrow longitudinally, bearing field of ~10 medium length setae. S8+9 fused, with line of fusion not demarcated, with distal 2/3rds of segment well sclerotized, terminus rounded, extending distally well beyond the tip of T9+ect; terminal setae dense, not enlarged, only few (~4) on each side with small flanges. Gonarcus (posterior view) rounded, with slight angle mesally; apodemes, bridge slender (all views), without mesal enlargement; mesal process digitiform, bending mesally (dorsal view). Mediuncus elongate, narrow, slightly bent dorsally (lateral view), with quadrate base, rounded distally, terminus without knob. Gonosaccus large, robust, with two large pouches each bearing a lateral plate with large field of robust, elongate, slightly curved gonosetae arising from large sockets (bases). Hypandrium internum not found.

##### Known distribution.

COLOMBIA: States of Antioquia, Valle del Cauca.

##### Specimens examined

(in addition to holotype). 1F, same data as holotype (paratype, USNM). Colombia, Dept. of Valle [Valle del Cauca], Carretera a Biventura, Km 18, 5–10/IX/1978, M. D. Tidwell (1M, paratype, FSCA).

#### Ungla laufferi

Taxon classificationAnimaliaNeuropteraChrysopidae

(Navás, 1922)

Figs 48 , 49 , 50 , 51 , 52 , 53 , 54 , 55 , 56

**Figure 48.**
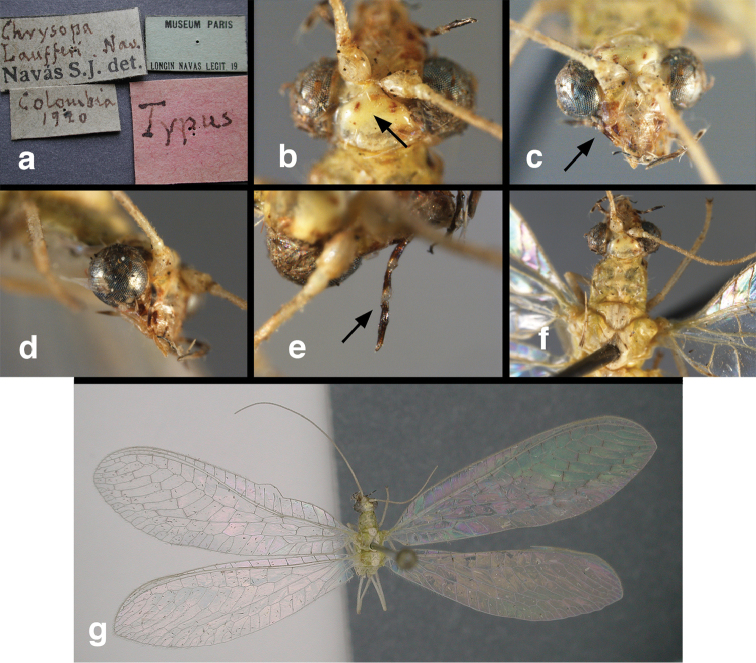
*Chrysopa laufferi* Navás: External features, (**a**) labels (**b**) head, dorsal [Arrow indicates red dorsal marks on vertex.] (**c**) head, frontal [Arrow indicates red subocular and clypeal marks.] (**d**) head, lateral (**e**) head, dorsal [Arrow indicates labial palp.] (**f**) head, prothorax, mesothorax, dorsal (**g**) habitus with wings (Colombia, no locality, holotype, sex unknown, MNHN).

**Figure 49.**
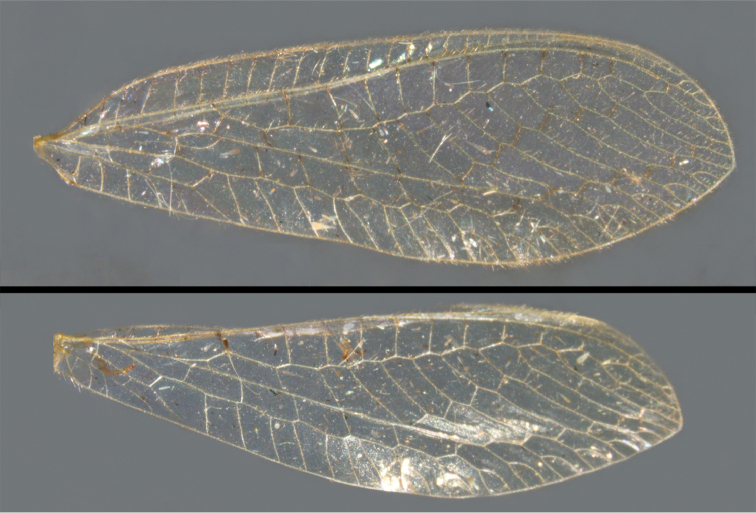
*Chrysopa laufferi* Navás: Right wings (Colombia, no locality, holotype, sex unknown, MNHN).

**Figure 50.**
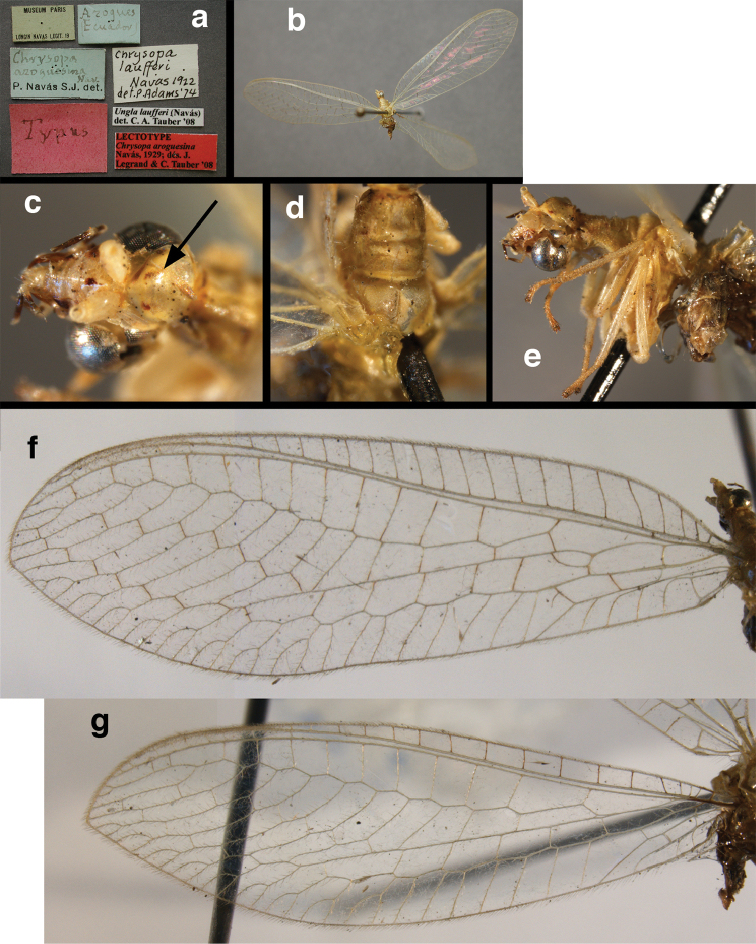
*Chrysopa aroguesina* Navás: External features, (**a**) labels (**b**) habitus, dorsal (**c**) head, dorsal [Arrow indicates red marks on vertex.] (**d**) prothorax, mesothorax, dorsal (**e**) head, thorax, dorsolateral (**f**) forewing (**g**) hindwing (Ecuador, Azogues, lectotype, female, MNHN).

**Figure 51.**
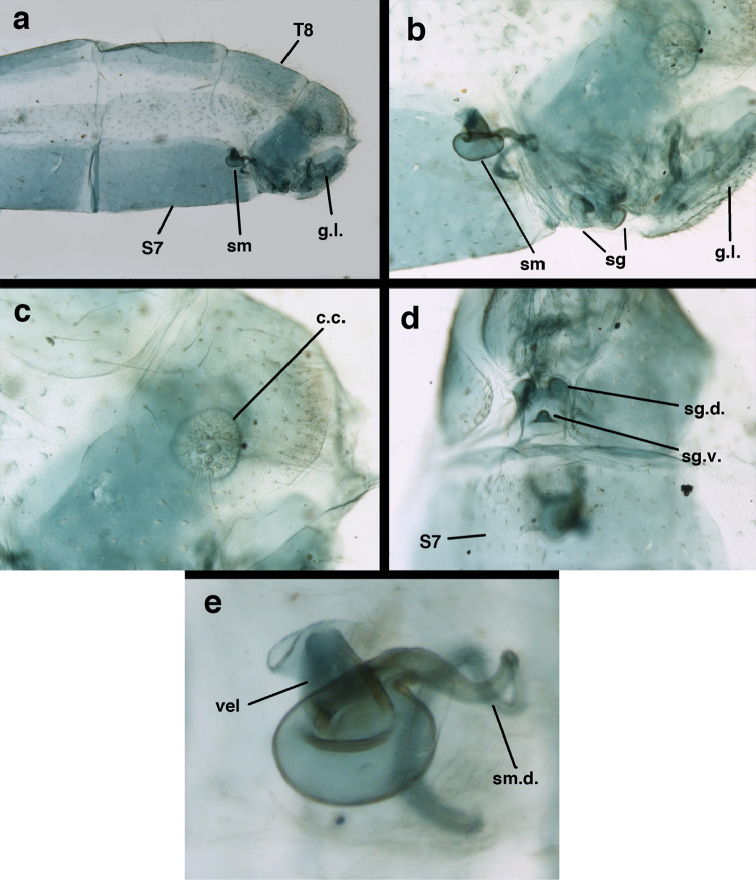
*Chrysopa aroguesina* Navás: Female abdomen, (**a**) segments A6-terminus, lateral (**b**) terminalia, lateral (**c**) callus cerci (**d**) terminalia, ventral (**e**) spermatheca. **c.c.** callus cerci **g.l.** gonapophysis lateralis **S7** seventh sternite **sg** subgenitale **sg.d.** dorsal lobe of subgenitale **sg.v.** ventral lobe of subgenitale **sm** spermatheca **sm.d.** spermathecal duct **T8** eighth tergite **vel** spermathecal velum (Ecuador, Azogues, lectotype, MNHN).

**Figure 52.**
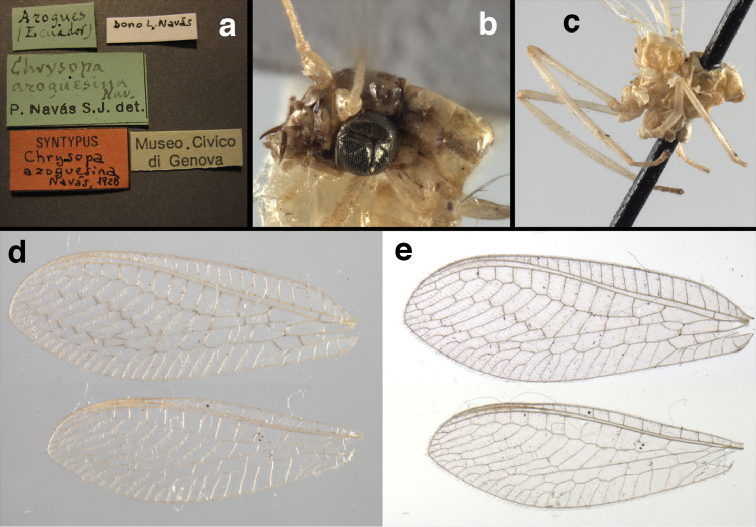
*Chrysopa aroguesina* Navás: External features, (**a**) labels (**b**) head (damaged), dorsolateral (**c**) thorax (damaged), dorsolateral (**d**) wings showing markings on veins (**e**) wings showing venation (Ecuador, Azogues, paralectotype, MSNG).

Chrysopa laufferi Navás, 1922. *Rev. R. Acad. Cienc. exactas fis. nat. Madrid* (“1921”) 19: 260; “Colombia. Un ejemplar en mi colección, donativo de Don Jorge Lauffer, a quien me complazco en dedicar esta especie”. Penny 1977: 19 (list); Oswald 2015 (catalog). Ceraeochrysa laufferi (Navás), by Brooks and Barnard 1990: 269. Ungla laufferi (Navás), by Legrand et al. 2008: 149; Freitas et al. 2009: 556 (removal from Ungla, return to Ceraeochrysa, redesc, tax); Tauber and Flint 2010: 57-58 (return to Ungla); Oswald 2015 (catalog). **Holotype** (Figs 48, 49). MNHN, sex unknown (examined, abdomen missing); holotype by original designation (see Legrand et al. 2008: 149). The type locality is an unspecified location in Colombia.
Chrysopa aroguesina Navás, 1929. *Rev. chil. Hist. nat.* (“1928”) 32: 110–111; “Ecuador: Azogues. Campos leg. Col. m”. Penny 1977: 16 (list, as “*azoguesina*”, incorrect subsequent spelling); Poggi 1993: 424 (type, as C. “*azoguesina*”, incorrect subsequent spelling); Oswald 2015 (catalog). Ceraeochrysa aroguesina (Navás), by Brooks and Barnard 1990: 268 (as C. “*azoguesina*”, incorrect subsequent spelling); Legrand et al. 2008: 115-116 (synonymy with C. laufferi); Freitas et al. 2009: 556 (synonymy reversed); Tauber and Flint 2010: 57–58 (synonymy reinstated); Oswald 2015 (catalog). **Lectotype** (Figs 50, 51). MNHN, female (examined); lectotype designated by Legrand et al. (2008: 115–116). A paralectotype (examined, sex unknown, MSNG) is badly damaged (Fig. 52), but the features that are visible are similar to those of the lectotype. Both specimens were collected in Azogues, the capital city of Cañar Province (~2,500 m) in south-central Ecuador. **Support for synonymy.** See Tauber and Flint 2010: 57–58.

##### Diagnosis.

This species and *U. stangei* (see below) are the only two *Ungla* species known to have light green body color, a dorsal yellow stripe, and a pair of red spots or small, crescent-shaped marks on the vertex (in place of the inverted U-shaped mark that typifies most *Ungla* species). The wings of both species have slightly acute tips; the longitudinal and transverse veins are largely yellow to light green; most transverse veins are pale mesally with small brown marks at both ends where they intersect with longitudinal veins. The gradate veins, the first two r-m crossveins, and the last crossvein of the distal b’ cell are entirely brown and are bordered with brown suffusion.

Adults of *U. laufferi* can be distinguished from *U. stangei* by their smaller wing size (forewings = 13.5–15.9 mm, 11–13 radial cells, 6–7 inner gradate veins, 6–8 outer gradates), and 6–7 gradate cells that are long and narrow. Also, in the *U. laufferi* male, the S8+9 is elongate and its dorsal surface tapers evenly throughout the entire segment; the gonarcus is arcuate and has elongate gonarcal arms that extend downward from the gonarcal bridge (smoothly or slightly angled); they do not recurve markedly below the gonarcal bridge and gonosaccus.

##### Redescription.

Pale green, with dorsal surface mostly yellowish from vertex to tip of abdomen. Head with vertex raised, smooth, shiny, with pair of red to reddish brown spots or crescent shaped marks laterally; frons smooth, flat, with or without small mesal mark anteromesal to base of antennae; gena, lateral edge of clypeus with reddish to brownish stripe; maxillary palp with distal three segments dark brown, pale at articulations, basal segments unmarked; labial palp unmarked except tip of distal palpomere dark brown; antenna light yellow basally, becoming slightly brownish distally; scape, pedicel without marks. Prothorax length and width about equal, narrowing anteriorly, unmarked or slightly tinged with red. Legs yellow-green, with similarly colored setae; tarsus paler; base of claws strongly dilated. Measurements: head width: 1.2–1.4 mm; ratio head width : eye width: 2.2–2.4 : 1; prothorax width: 0.9–1.0 mm, length: 0.6–0.7 mm.

Forewing, hindwing elongate, with broadly acute tips; membrane mostly clear, hyaline, with fumose areas around brown sections of transverse veins, gradate veins; stigma clear to slightly opaque; longitudinal veins mostly green; transverse veins with brown at most intersections; gradate veins, first two r-m crossveins, last crossvein of distal b’ cell entirely brown, with surrounding membrane suffused with brown. Forewing 13.5–15.9 mm long, 4.6–5.3 mm wide (ratio, L : W = 2.9–5.0: 1); height of tallest costal cell 0.8–1.2 mm (cell number 8–9); width of first intramedian cell 0.8–1.0 mm; 11–13 radial cells (closed cells between R and Rs); third gradate cell 1.9–2.2 mm long, 0.4–0.5 mm wide (ratio, L : W = 3.8–5.0 : 1); fourth gradate cell 1.9–2.4 mm long, 0.4–0.5 mm wide (ratio, L : W = 4.6–5.5 : 1); 4–5 Banksian cells (b cells), 4–5 b’ cells; 6–7 inner gradates, 6–8 outer gradates. Hindwing 12.0–14.4 mm long, 3.8–4.5 mm wide (ratio, L : W = 3.1–3.3 : 1), 11–14 radial cells, 3–4 Banksian (b) cells, 4–5 b’ cells, 5–7 inner gradates, 3–8 outer gradates.

Male. Abdomen with small spiracles (e.g., A7: spiracle diameter ~0.02x length of sternite); T9+ectoproct relatively long (~2/3× length of T7), with dorsal invagination shallow (~1/3 dorsal length of T9+ect), margins of invagination rounded throughout; dorsal margin of T9+ect rounded distally (above anus), often compressed, thus appearing straight (especially in teneral specimens); posterior margin of ectoproct relatively straight, posteroventral corner slightly extended distally, without knob; dorsal apodeme along ventral margin sinuous, with arms extending dorsally on each side of callus cerci; callus cerci large, ovate, margin unsclerotized at top. S8+9 fused, with line of fusion perceptible; dorsum tapering gently throughout, with margin slightly irregular; ventral apodeme on margin of S8 lightly, irregularly sclerotized, ventral apodeme on margin of S9 more densely sclerotized; terminus extended distally, but not beyond T9+ect, flat, distal margin upturned, plate-like, well sclerotized (setae mostly missing). Gonarcus U-shaped or slightly acute (probably teneral), with bridge slender, apodemes elongate, extending below bridge then bending posteriorly, with quadrate unarticulated process mesally; mediuncus relatively broad-based, with pair of straight, sclerotized rods, extending from base along sides of mediuncus, coalescing distally in rounded, down-turned beak; gonosaccus bilobed, each lobe with single, relatively sparse patch of gonosetae arising from enlarged setal bases, lobes evertible (setae extended) or withdrawn (patches of setae opposite each other); hypandrium internum V-shaped, with flat base, mature specimens: arms expanded, scalloped, comes hook-shaped.

##### Variation.

Among the specimens we examined, there was considerable variation in the size and depth of the reddish markings on the frons mesal to the eyes and below the base of the antenna. The specimens from the unknown type locality in Colombia, Azogues in south-central Ecuador, and Cajamarca in Peru had distinct markings, whereas the specimens from Cuenca, Huancavelica, and Matucana, Lima, in Peru, had no mark or only a speck of red on the frons mesal to the eyes.

##### Known distribution.

COLOMBIA (no locality). ECUADOR (south central): Provinces of Cañar, Huancavelica. PERU (central coast and north): Provinces of Lima, Cajamarca, Cuzco.

##### Specimens examined

(in addition to types above). ECUADOR. *Huancavelica*: Cuenca, 10-III-1965, L. E. Peña (2M, CAS). PERU. *Cajamarca*: shrubs on hillside, 1 mile S.W. of town, 26-VIII-1971, P. S & H. L. Broomfield, B. M.1971-486, Fertile valley in Andes, 8000 ft. (2M, BMNH); *Cuzco*, 23/XI/1965, H. & M. Townes (FSCA); *Lima*: Matucana, 2389 n., 28-30/VI/1974, C. Porter & L. Stange (5M, 1F, IFML; 5M, 2F, FSCA).

**Figure 53.**
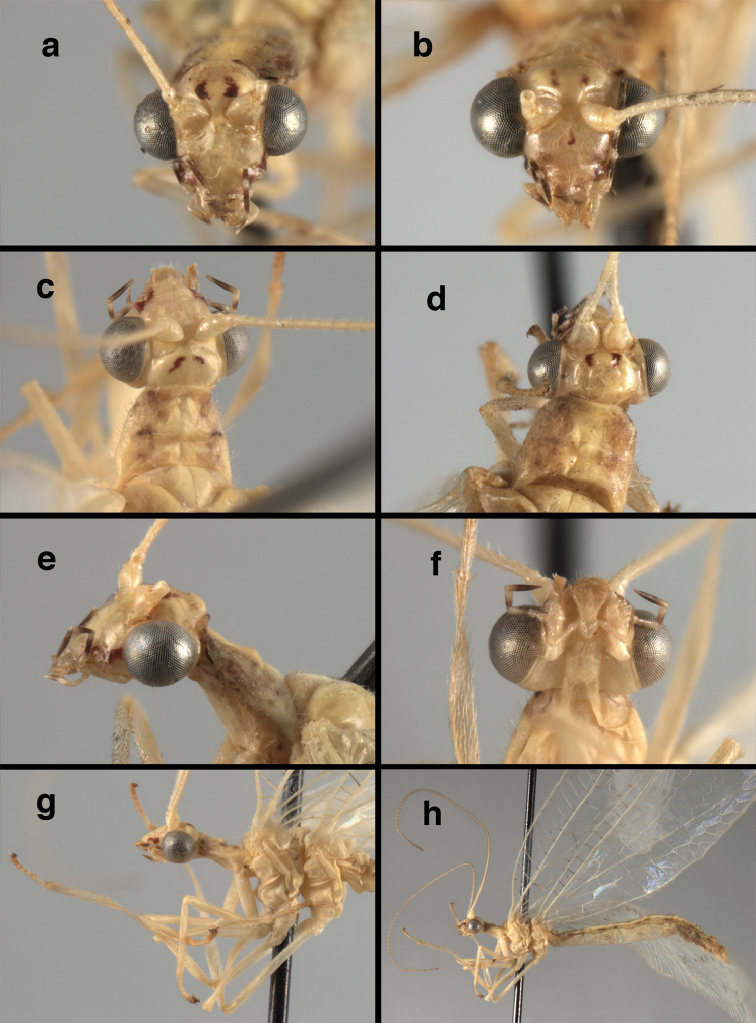
*Ungla laufferi* (Navás): External features, (**a, b**) head, frontal [Note variation in marks on vertex and frons.] (**c, d**) head, prothorax, dorsal (**e**) head, lateral (**f**) head, ventral (**g**) head, thorax, lateral (**h**) body, lateral (**a, e** Peru, Lima, male, FSCA; **b** Ecuador, Huancavelica, male, CAS; **c, f, g, h** Peru, Cuzco, male, FSCA; **d** Peru, Lima, female, FSCA).

**Figure 54.**
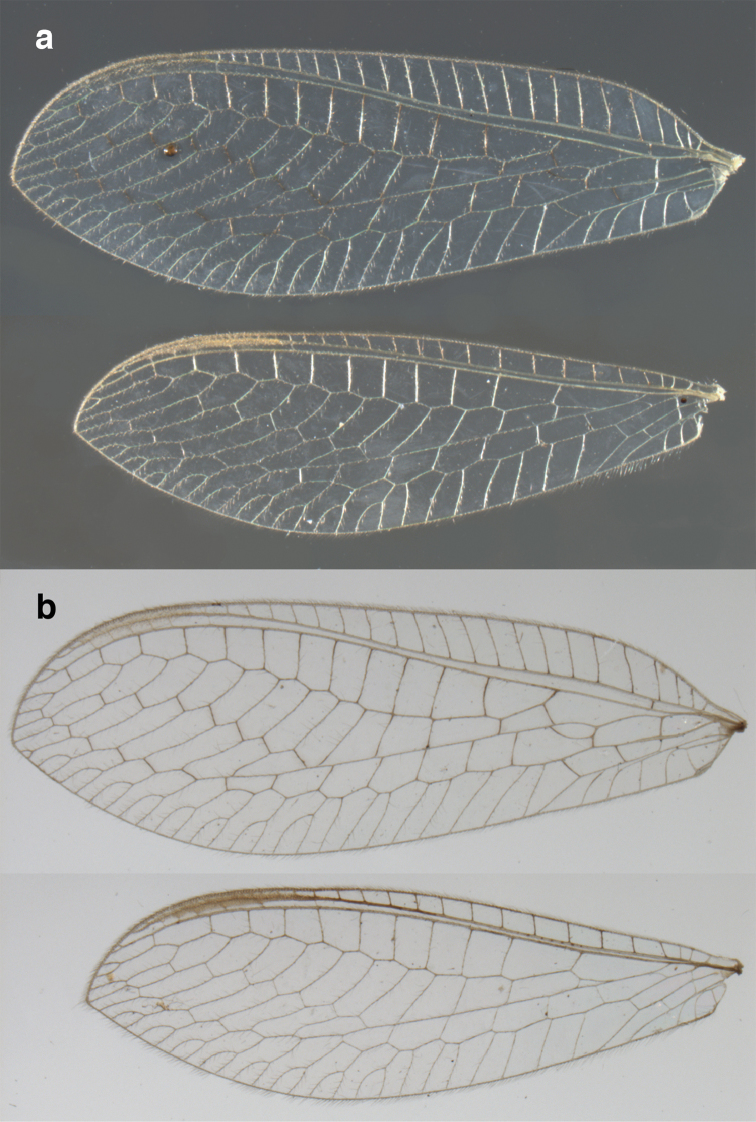
*Ungla laufferi* (Navás): Wings (**a** Peru, Lima, male, FSCA **b** Peru, Cajamarca, male, BMNH).

**Figure 55.**
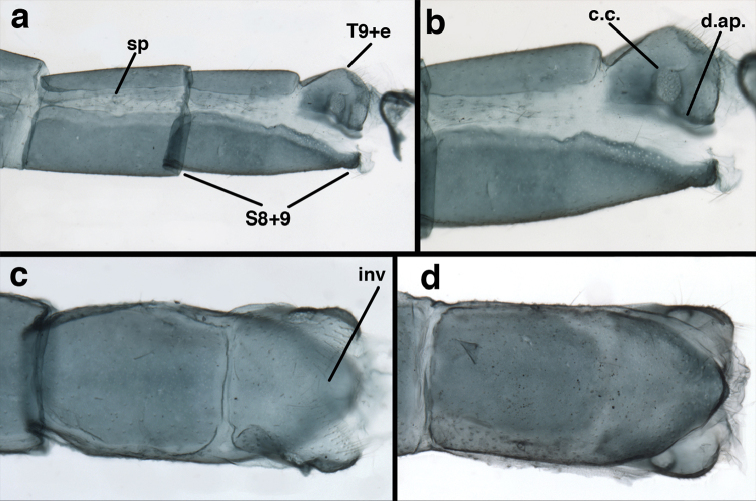
*Ungla laufferi* (Navás): Male abdomen, (**a**) segments A7-terminus, lateral (**b**) segments A8-terminus, lateral (**c**) tergite 8, tergite 9+ectoproct, dorsal (**d**) sternite 8+9, ventral. **c.c.** callus cerci **d.ap.** dorsal apodeme **inv** dorsal invagination of T9+ectoproct **sp** spiracle **S8+9** fused eighth and ninth sternites **T9+e** ninth tergite + ectoproct (**a–c** Peru, Lima, FSCA; **d** same, IFML).

**Figure 56.**
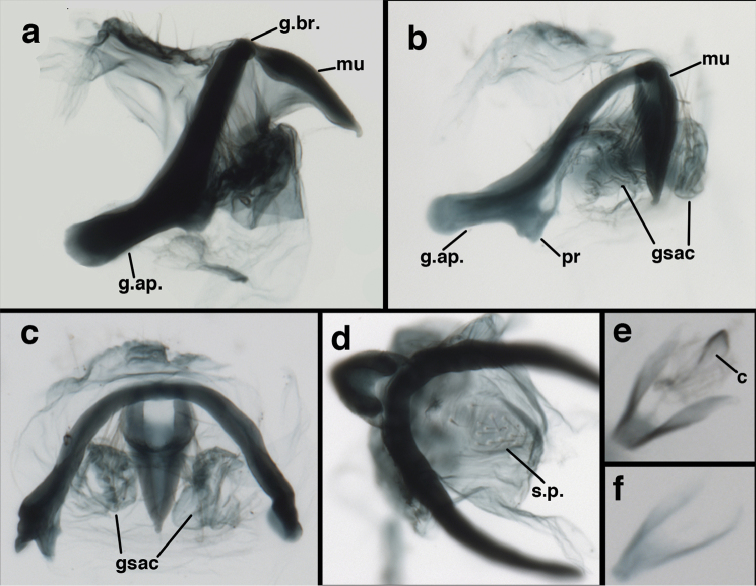
*Ungla laufferi* (Navás): Male genitalia, (**a**) gonarcal complex, lateral with gonosaccus collapsed (**b**) gonarcus, lateral with gonosaccus partially expanded (**c**) gonarcus frontal, gonosaccus partially expanded (**d**) gonarcus, posterior with subanal membrane and plate folded below (**e**) hypandrium internum, well sclerotized (**f**) hypandrium internum, slightly teneral. **c** comes **gsac** gonosaccus **g.ap.** gonarcal apodeme **g.br.** gonarcal bridge **mu** mediuncus **pr** unarticulated process on frontal margin of gonarcal apodeme **s.p.** setose subanal plate (all: Peru, Lima; **a, d, e** IFML; **b, c, f** FSCA).

#### Ungla martinsi

Taxon classificationAnimaliaNeuropteraChrysopidae

Sosa, 2015

**Figure 57.**
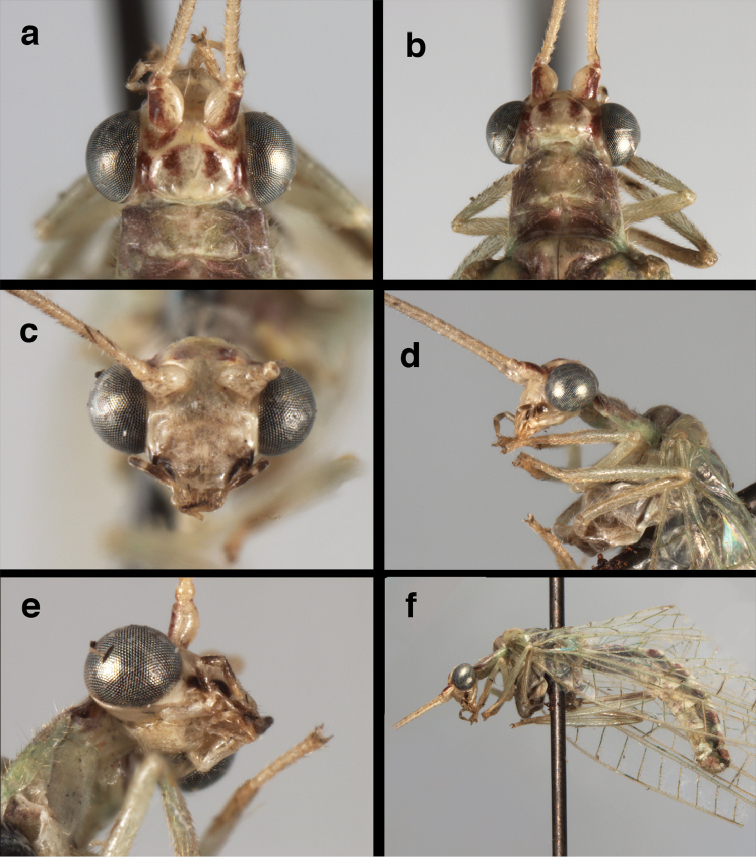
*Ungla martinsi* Sosa: External features, (**a**) head, dorsal (**b**) head, prothorax, dorsal (**c**) head, frontal (**d**) head, lateral (**e**) head, ventrolateral (**f**) body, lateral (all: Venezuela, Aragua, DEBU; **a, b, d, f** female; **c, e** male).

**Figure 58.**
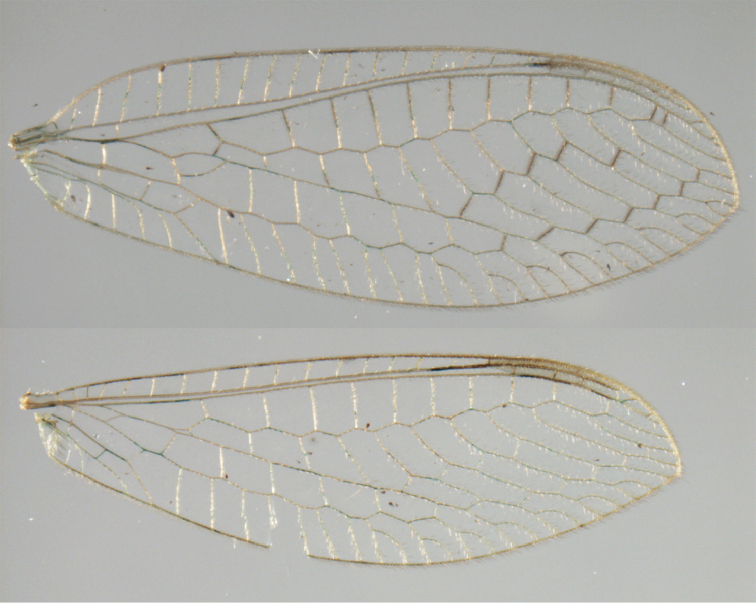
*Ungla martinsi* Sosa: Wings (Venezuela, Aragua, female, DEBU).

**Figure 59.**
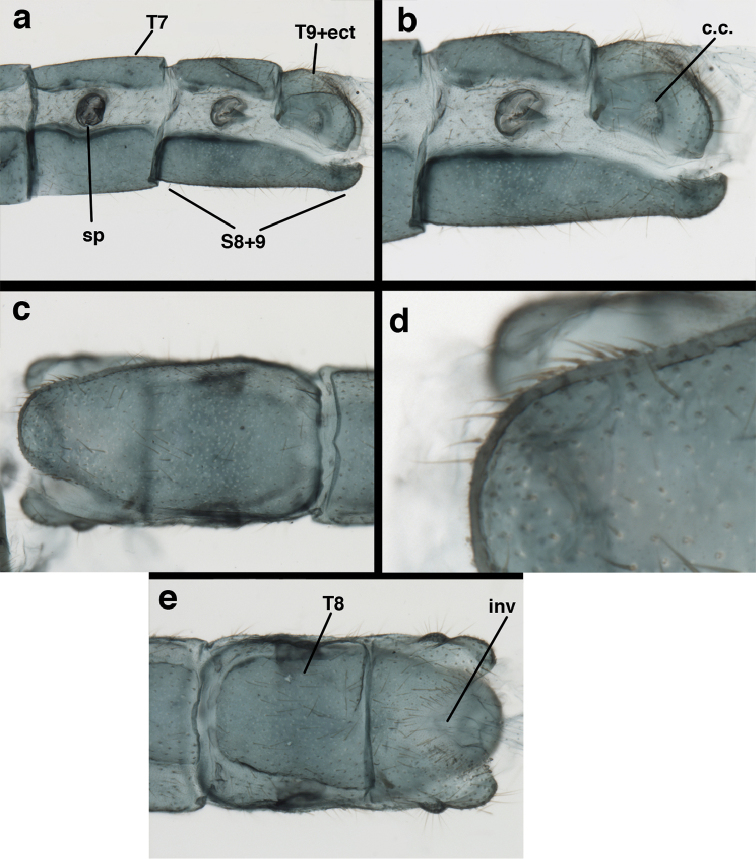
*Ungla martinsi* Sosa: Male abdomen, (**a**) segments A7-terminus, lateral (**b**) segments A8-terminus, lateral (**c**) fused sternites S8+9, ventral (**d**) tip of sternite S9, ventral, with enlarged, flanged setae (**e**) tergites T8 and T9, dorsal. **c.c.** callus cerci **inv** invaginated dorsal cleft in T9+ectoproct **sp** spiracle **S8+9** fused eighth and ninth sternites **T7, T8** seventh and eighth tergites **T9+e** ninth tergite + ectoproct (all: Venezuela, Aragua, DEBU).

**Figure 60.**
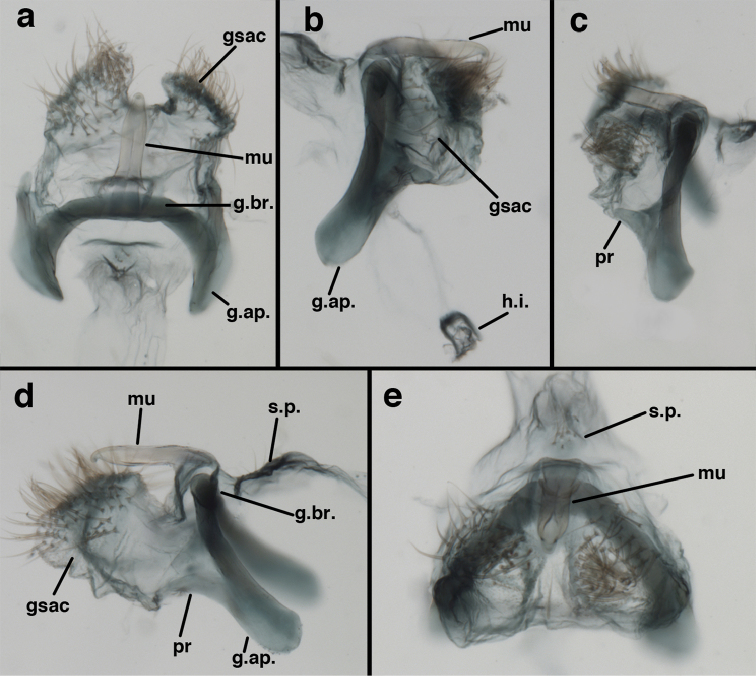
*Ungla martinsi* Sosa: Male genitalia, (**a**) gonarcal complex, dorsal, with gonosaccus expanded (**b**) gonarcus, lateral, with gonosaccus partially expanded (**c**) gonarcus, lateral, gonosaccus partially expanded (**d**) gonarcus, lateral, gonosaccus expanded (**e**) gonarcus, frontal, gonosaccus partially expanded. **gsac** gonosaccus **g.ap.** gonarcal apodeme **g.br.** gonarcal bridge **h.i.** hypandrium internum **mu** mediuncus **pr** unarticulated process on frontal margin of gonarcal apodeme **s.p.** setose subanal plate (all: Venezuela, Aragua, DEBU).

Figs 57 , 58 , 59 , 60 , 143d , 143e

Ungla martinsi Sosa, 2015. *Zootaxa* 4018 (2): 187-191; “VENEZUELA. *Portuguesa state*. Ospino, Santa Barbara, [9°28.695'N/69°33.111W], 1340 m, 10–11.v.2013, F. Sosa & C. Martins Leg. Collected with light trap. Deposited in the MJMO.” **Holotype.**MJMO, male. For images of the type see Sosa (2015); for labels, see Fig. 143d here.
Ungla rubricosa Sosa, 2015. *Zootaxa* 4018 (2): 194-196; “VENEZUELA. *Aragua*: P. N. Henri Pittier, 1140 m, 20.ii.2008, F. Sosa, F. Díaz & R. Zuñiga Leg. Collected with trap light. Deposited in the MJMO.”. **syn. n. Holotype.**MJMO, male (teneral). For images of the type see Sosa (2015); for labels, see Fig. 143e here. **Support for synonymy.** The original description of U. rubricosa was based on a single teneral male and two female specimens from Venezuela (Sosa 2015). Additional Venezuelan specimens used in the study here, allowed us to reexamine the pattern of variation among a larger group of specimens and to establish that the two species indeed are the same.

##### Diagnosis

(also see Sosa 2015). This phenotypically variable species is recognized by its green body, marked with deep red; light yellow to cream-colored head; U-shaped marking on vertex either broken mesally or connected via thin mesal line; margins of head adjacent to eyes marked with red band; scape and pedicel with red dorsal stripe; flagellum cream-colored; frons unmarked; palpi marked with black. The wing venation, with dark brown transverse veins and fumose R-Rs crossveins and gradates, and the male genitalia are also distinctive.

*Ungla martinsi* resembles another Venezuelan species *U. curimaguensis*, in size, male abdominal and genital characteristics, and perhaps head markings. However, the two species differ notably in that *U. curimaguensis* has largely green forewing venation and it lacks the dark genal marks and marked maxillary and labial palpi of *U. martinsi*.

##### Redescription.

Head cream-colored to yellow with red markings; vertex smooth, shiny, with U-shaped marking reduced to two arms or two prominent spots separated at center; area between antennae unmarked; red stripe between eye and sides of vertex, extending posteriorly; dorsal antennal fossa entirely red or with thin reddish stripe mesally, not extending between antennae. Frons cream-colored, without markings; clypeus cream-colored, with black stripe on lateral margin, contiguous with black genal mark, which reaches almost to eye. Antenna mostly cream-colored, scape, pedicel with broad dorsolateral red stripe; flagellum yellow with black bristles; maxillary palp with two basal segments pale, distal three segments black dorsally, with intersections, tip of distal palpomere pale; labial palp with basal two segments pale, ultimate segment light brown to black shading laterally.

Prothorax green with cream-colored stripe mesally, pair of broad, dark red stripes sublaterally; transverse furrow in posterior region, not deep, reaching lateral margins of segment, long, cream-colored to golden setae throughout. Mesothorax reddish brown centrally, green, marked with red laterally; metathorax green with reddish brown markings. Legs light green, unmarked. Measurements: head width: 1.4–1.5 mm; ratio head width : eye width: 2.7: 1; prothorax width: 0.9–1.2 mm, length: 0.7–0.8 mm.

Forewing, hindwing with moderate width. Forewing with rounded apex, robust, but without heavy venation, with very slight swelling at furcation of Cu; stigma lightly marked with brown or pale, with four to five light brown to pale subcostal crossveins below; longitudinal veins light green, costal, radial crossveins light to dark brown anteriorly, pale or brown posteriorly; gradate veins, distal Psm-Psc crossvein black, surrounded by dense dark brown shading; gradates in parallel series or sometimes with distal one or two inner gradates closer to outer gradates; basal inner gradate meeting Psm. Forewing 11.2–13.7 mm long, 3.6–4.8 mm wide, (ratio, L : W = 2.8–3.1 : 1); height of tallest costal cell 0.7–0.9 mm (cell number 6); length of first intramedian cell 0.8–0.9 mm; 10–11 radial cells (closed cells between R and Rs); third gradate cell 1.5–2.0 mm long, 0.4–0.5 mm wide (ratio, L : W = 4.0–4.5 : 1); fourth gradate cell often missing, when present 1.3–2.0 mm long, 0.3–0.4 mm wide (ratio, L : W = 3.8–4.7 : 1); 4 Banksian cells (b cells), 4 b’ cells; 4–6 inner gradates, 5–7 outer gradates. Hindwing with apex subacute, venation light green; 9.9–12.2 mm long, 3.0–3.8 mm wide (ratio, L : W = 3.0–3.3: 1), 10–11 radial cells, 3 Banksian (b) cells, 4 b’ cells, 3–5 inner gradates, 4–6 outer gradates.

Male. Abdomen with large spiracles (e.g., A7: spiracle diameter ~0.2× length of sternite); A7-A9 with numerous setae extending from robust setal bases; T9+ectoproct relatively long (~0.6× length of T7), with dorsal invagination deep (~0.5× dorsal length of T9+ect), margins of invagination almost straight, base rounded; dorsal margin of T9+ect sloped distally (above anus); posterior margin of ectoproct rounded throughout; ventral margin of T9+ect straight, with lightly sclerotized, straight apodeme, slightly below sclerotization around callus cerci, posterior corner of apodeme bent mesally in small, rounded knob; posteroventral corner of T9+ect appearing angular (lateral view); callus cerci large, ovate, with entire margin lightly sclerotized. S8+9 fused, with line of fusion not readily perceptible; dorsal margin with apodeme extending along basal ~1/2 length of segment; segment extending further without apodeme, then gradually sloping ventrally to tip of segment; terminus extending distally, well beyond tip of T9+ect, heavily sclerotized, upturned distally, concave in posterior view; terminal setae on posterodorsal margin of S8+9 enlarged, with small, flange-like protrusions basally. Gonarcus arcuate (dorsal, ventral views), V-shaped (frontal, caudal views); bridge robust, moderately wide throughout; arms elongate, rounded distally, dorsal section with triangular process extending posteriorly toward gonosaccus; mediuncus with narrow base, paired internal rods adjacent to each other for ~3/4 distance from base, fusing near terminus; dorsal surface of mediuncus fairly straight, with short, rounded (blunt) beak distally; gonosaccus bilobed, each lobe large, bearing dense patch of gonosetae; gonosetae robust, arising from enlarged setal bases; hypandrium internum quadrate, with narrow arms, rounded comes.

Female. See Sosa (2015).

##### Biology.

Oviposition of stalked, clustered eggs (probably infertile) (see images, Sosa 2015). None underwent embryonic development.

##### Known distribution.

VENEZUELA: States of Aragua, Portuguesa.

##### Specimens examined

[in addition to those listed by Sosa (2015)]. Venezuela. *Aragua*: Rancho Grande, 10–21/II/1969, Duckworth & Dietz (1F, USNM); Rancho Grande, IX-20-1973, B. Villegas (1F, UCDC); Rancho Grande, 18 km NW Maracay, at light, 22.iii.1992, C. Michalski (1F, AMNH); Henri Pittier Nat. Park: Nr Rancho Grande, 1100–1200m, 19–20.i.1996, J. & A. Skevington (1F, DEBU), Rancho Grande Bio Station, white light, 24.i.1996, 1100 m, J. & A. Skevington (1M, DEBU).

#### Ungla mexicana

Taxon classificationAnimaliaNeuropteraChrysopidae

Tauber sp. n.

http://zoobank.org/D9E28126-29C6-4D56-8B96-2C96BF056B43

**Figure 61.**
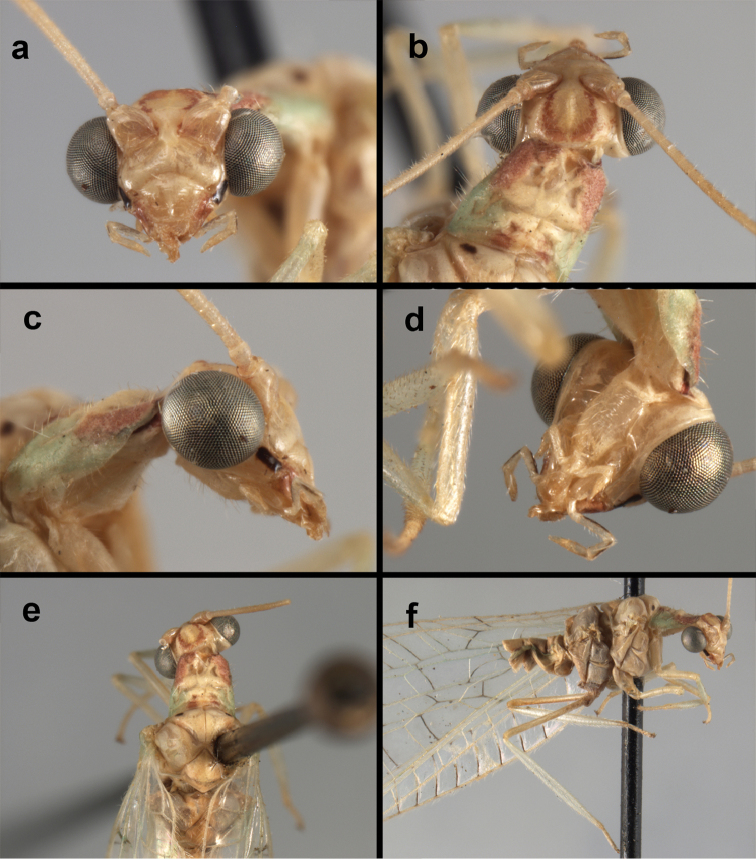
*Ungla mexicana* Tauber, sp. n. External features, (**a**) head, frontal (**b**) head, prothorax, dorsal (**c**) head, prothorax, lateral (**d**) head, ventrolateral (**e**) head, thorax, dorsal (**f**) head, thorax, lateral (Mexico, Chiapas, holotype, female, CAS).

**Figure 62.**
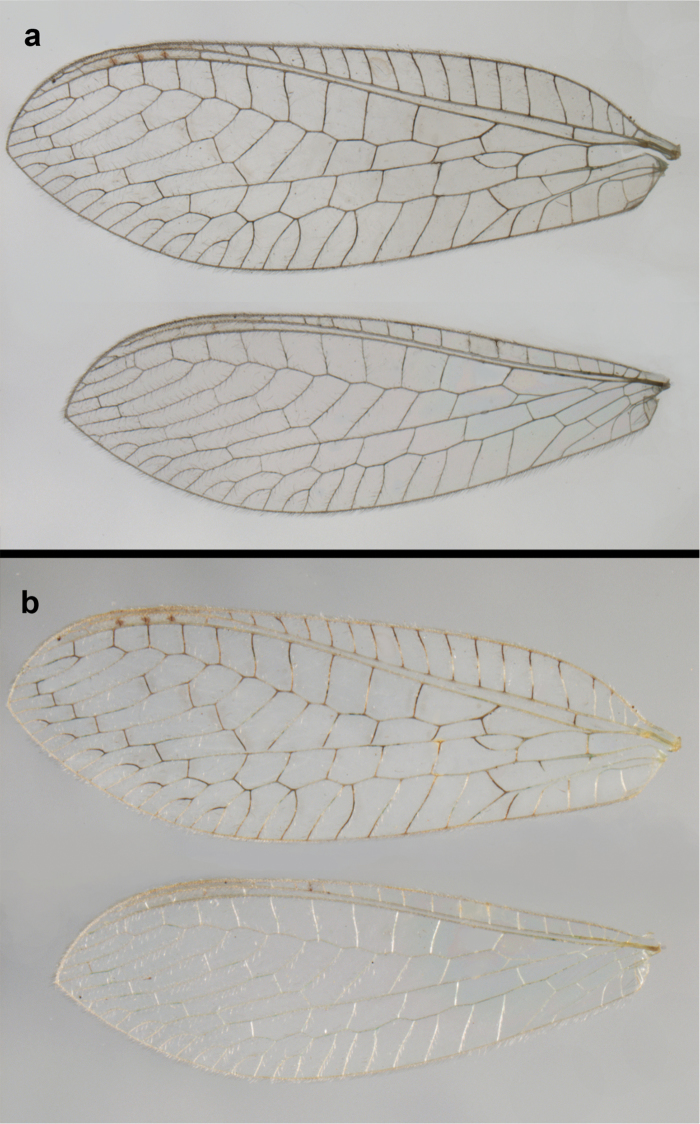
*Ungla mexicana* Tauber, sp. n. Wings, (**a**) venation emphasized (**b**) coloration of veins emphasized (Mexico, Chiapas, holotype, female, CAS).

**Figure 63.**
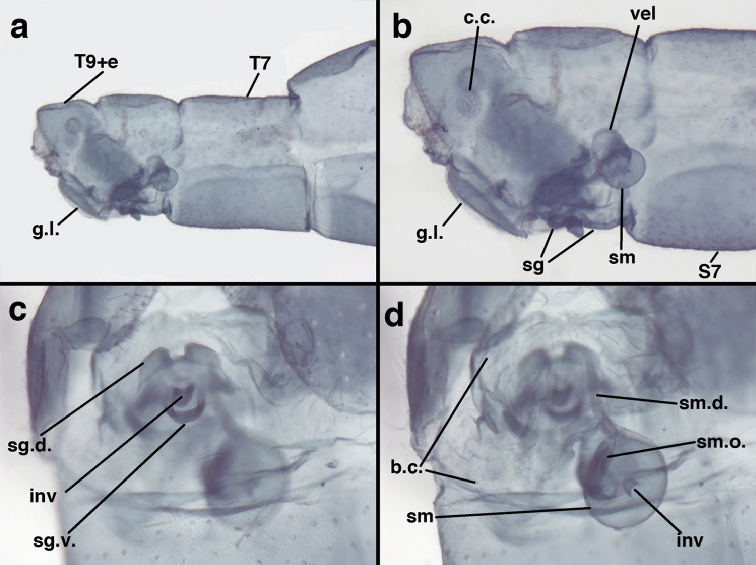
*Ungla mexicana* Tauber, sp. n. Female abdomen, (**a**) segments A7-terminus, lateral (**b**) terminalia, lateral (**c, d**) genitalia, ventral. **b.c.** bursa copulatrix **c.c.** callus cerci **g.l.** gonapophysis lateralis **inv** spermathecal invagination **sg** subgenitale **sg.d**. dorsal lobe of subgenitale **sg.v.** ventral lobe of subgenitale **sm** spermatheca **sm.d.** spermathecal duct **sm.o**. spermathecal opening to bursa copulatrix **S7** seventh sternite **T7** seventh tergite **T9+e** fused ninth tergite and ectoproct **vel** spermathecal velum (Mexico, Chiapas, holotype, CAS).

Figs 61 , 62 , 63 , 144c

##### Holotype

(Figs 61–63, 144c). FSCA, female. Mexico, Chiapas, 21 mi. SE San Cristóbal de las Casas, 2.iv.1962, F. D. Parker, L. A. Stange.

The holotype (a female) is the only specimen of this species that we have seen, and we were reluctant to describe it as new on the basis of such limited material. However, the specimen is very well preserved, and its external features (head and body coloration and markings, wings) are notable. The abdomen is cleared, stained, and in a vial attached to the specimen. Because of the specimen’s importance as the northernmost record for the genus, we describe it to facilitate future identifications.

##### Etymology.

The type locality of this species is in the highlands of southern Mexico (State of Chiapas). Its species name, *mexicana* (Latin, adj., fem.), recognizes that it is the first, and at this time the only, species of *Ungla* reported from Mexico.

##### Diagnosis.

The female holotype expresses many of the characteristics that typify most *Ungla* species: a U-shaped marking around the margins of the vertex, dark genal markings, dark [albeit, thin] markings on the palpi, and a typical doughnut shaped spermatheca.

*Ungla mexicana* is recognized by its green body with reddish markings; prothorax with a distinctive red lateral band that extends only about one half the length of the segment (from the anterior margin to the midlength of the segment), and mesothorax with a pair of bold black markings anteriorly. The *U. mexicana* forewing venation is green, with most longitudinal and transverse veins marked with dark brown at intersections; crossveins are dark brown, with the gradate veins having brown suffusion on the adjacent membrane. This species most closely resembles *U. martinsi*; however, the two differ as follows. In addition to the above features, the U-shaped marking on the *U. mexicana* vertex is much larger, the scape is without a distinct dorsal stripe, and the distal three maxillary palpomeres have a thin lateral stripe rather than being black throughout.

##### Description.

Head cream-colored with red markings; vertex smooth anteriorly, shiny, with U-shaped marking large, robust, lateral arms separated anteriorly, meeting posteriorly, with lateral margins marked with red adjacent to eyes, posterior section unmarked; dorsal fossae probably with small, red mesal mark; area between antennae unmarked; frons cream-colored, unmarked; clypeus with lateral margin red; tentorial pits amber-colored; gena with bold, black stripe throughout, contiguous with red clypeal mark. Antenna: cream-colored, dorsal surface of scape possibly with tinge of red, but no stripe, flagellar setae pale; maxillary palp pale, distal segments with thin black lateral stripe; labial palp pale throughout.

Thorax mostly cream-colored; prothorax with pair of broad lateral bands – red anteriorly, green posteriorly; transverse furrow slightly posterior to middle of segment, not extending to lateral margins; setae pale. Legs cream-colored, unmarked. Measurements: head width: 1.4 mm; ratio head width : eye width: 2.3: 1; prothorax width: 1.0 mm, length: 0.8 mm.

Forewing, hindwing somewhat broad, apex slightly acute; membrane clear, hyaline. Forewing with veins robust, not crassate; Rs slightly sinuous; first intramedian cell ovate; basal inner gradate meeting Psm; stigma slightly opaque; longitudinal veins green, marked with brown at intersections, most transverse veins green, marked either basally (most R-Rs crossveins) or on both ends (most veins leaving Rs, most Psm-Psc crossveins, bases of distal forks on posterior margin); gradates, three of four crossveins below stigma, dark brown with brown suffusion on adjacent membrane; basal Rs-M crossveins, second icu crossvein dark brown without suffusion. Hindwing with venation green, unmarked. Forewing 14.1 mm long, 4.7 mm wide (ratio, L : W = 3.0 : 1); height of tallest costal cell 1.1 mm (cell number 5); width of first intramedian cell 1.0 mm; 10 radial cells (closed cells between R and Rs); third gradate cell 2.0 mm long, 0.5 mm wide (ratio, L : W = 4.3 : 1); fourth gradate cell 1.6 mm long, 0.7 mm wide (ratio, L : W = 3.5 : 1); 4 Banksian cells (b cells, sometimes distinct), 4 b’ cells; 4 inner gradates (basal one sometimes not clear), 6 outer gradates. Hindwing 12.7 mm long, 4.2 mm wide (ratio, L : W = 3.0 : 1), 10 radial cells, 3 Banksian (b) cells, 4 b’ cells, 3 inner gradates, 5-6 outer gradates.

Male. Unknown.

##### Known distribution.

MEXICO: Central Highlands region in state of Chiapas.

##### Specimens examined

. Holotype only.

#### Ungla nigromaculifrons

Taxon classificationAnimaliaNeuropteraChrysopidae

Sosa, 2015

**Figure 64.**
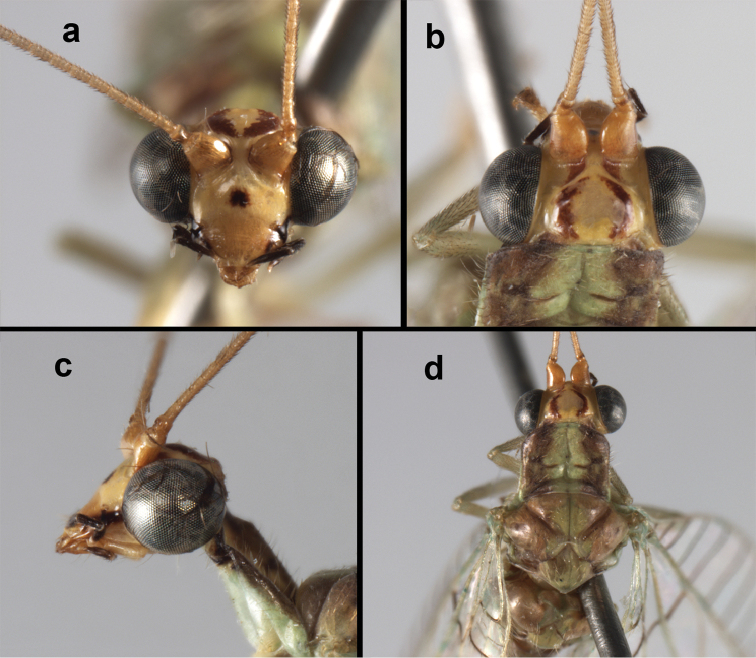
*Ungla nigromaculifrons* Sosa: External features, (**a**) head, frontal (**b**) head, dorsal (**c**) head, prothorax, lateral (**d**) head, thorax, dorsal (all: Venezuela, Táchira, UMSP; **a, c** male; **b, d** female).

**Figure 65.**
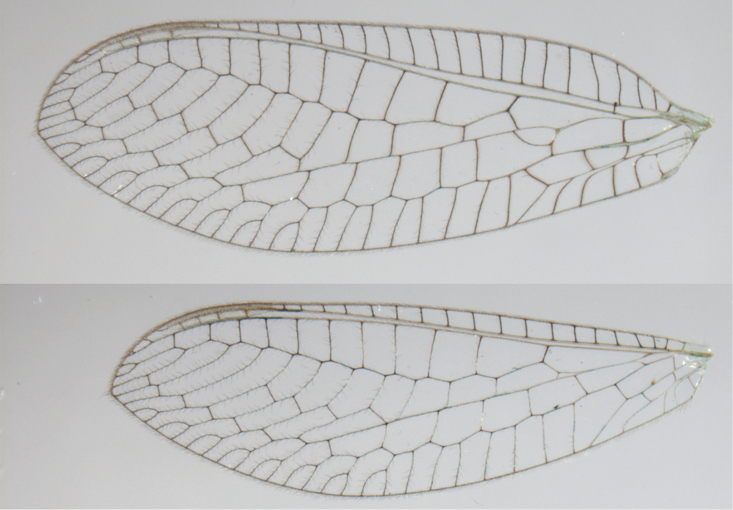
*Ungla nigromaculifrons* Sosa: Wings (Venezuela, Táchira, female, UMSP).

**Figure 66.**
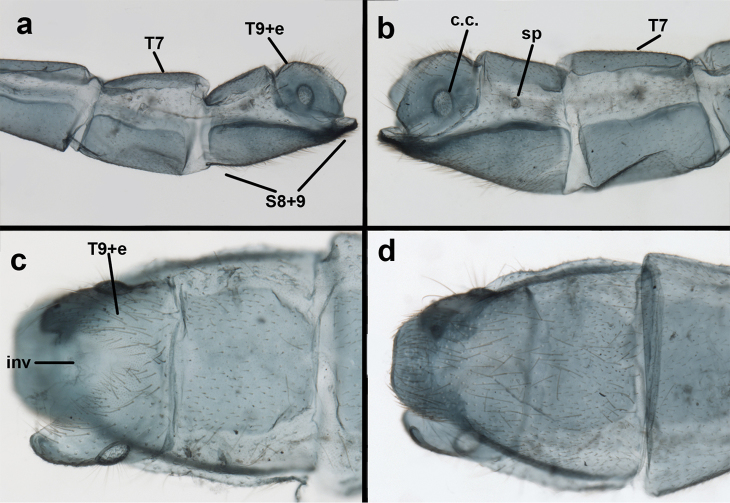
*Ungla nigromaculifrons* Sosa: Male abdomen, (**a**) segments A6-terminus, lateral (**b**) segments A7-terminus, lateral (**c**) T8, T9+ectoproct, dorsal (**d**) sternite 8+9, ventral. **c.c.** callus cerci **inv** invaginated dorsal cleft in T9+ectoproct **sp** spiracle **S8+9** fused eighth and ninth sternites **T7** seventh tergite **T9+e** ninth tergite + ectoproct (Venezuela, Táchira, UMSP).

**Figure 67.**
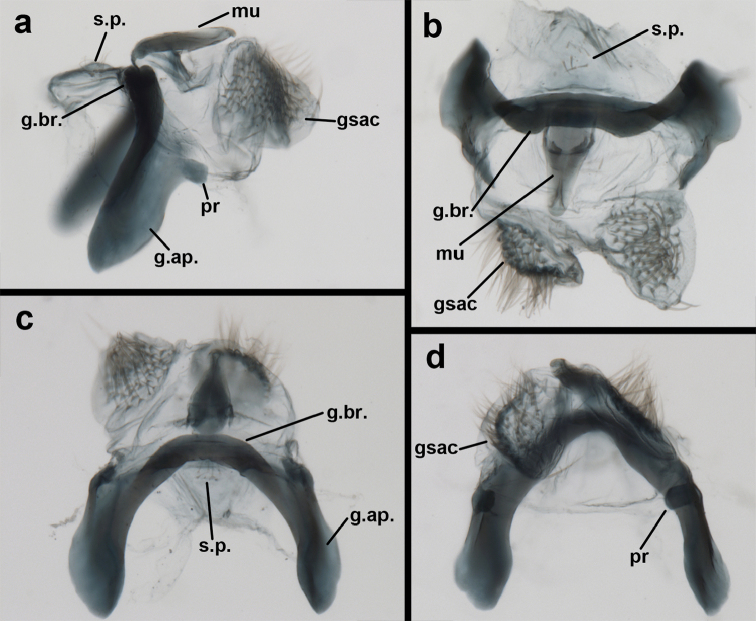
*Ungla nigromaculifrons* Sosa: Male genitalia, (**a**) gonarcal complex, lateral, with gonosaccus expanded (**b**) gonarcus, dorsal [Note internal structure of mediuncus.] (**c**) gonarcus, posterior and slightly dorsal [Note shape of gonarcus.] (**d**) gonarcus, frontal. **gsac** gonosaccus **g.ap.** gonarcal apodeme **g.br.** gonarcal bridge **mu** mediuncus **pr** unarticulated process on frontal margin of gonarcal apodeme **s.p.** setose subanal plate (Venezuela, Táchira, UMSP).

Figs 64 , 65 , 66 , 67 , 143f

Ungla nigromaculifrons Sosa, 2015. *Zootaxa* 4018 (2): 191–193; “VENEZUELA. *Trujillo state*: Boconó [9°14'N/70°15'W, 1270 m], 20.vii.1974, F. Fernández. H [Hijo] & M. Gaiani Leg. Deposited in the MIZA.” **Holotype.**MIZA, male. For images of the type see Sosa (2015); for labels, see Fig. 143f here. Much of the abdomen of the holotype is lost; the original description illustrates the structures that are present: T9+ect (lateral), S8+9 (ventral) and parts of the gonarcus.

##### Diagnosis.

This species is distinguished by a golden yellow head and a round, black spot on the frons, and dark wing venation. It differs from other species that have a prominent black frontal spot and cream-colored antennae (*U. stangei*, *U. favrei*, and *U. adamsi*) in that the scape is tinged with red, but otherwise unmarked (distinct stripe in *U. favrei*), the wings are smaller and with at most 4 to 8 gradate veins per series (8 to 11 for *U. stangei*), the clypeus and frons do not exhibit sexual dimorphism (male clypeus swollen and female frons with transverse marking in *U. adamsi*), and finally the hindwing has dark veins (unlike all the other species).

##### Redescription.

Head: As described by Sosa (2015). Maxillary palp with basal two segments cream-colored, distal three segments entirely black, intersections cream-colored; labial palp with basal segment pale, distal two entirely black. Thorax: Prothorax with transverse furrow in the posterior section of segment, marked with narrow, dark red transverse stripe extending almost to center of segment; setae long, golden. Mesonotum, metanotum green mesally, with pair of broad, dark red stripes laterally. Measurements: head width: 1.5 mm; ratio head width : eye width: 2.1–2.2: 1; prothorax width: 0.8–1.1 mm, length: 0.6–1.0 mm.

Forewing, hindwing with moderate width. Forewing with rounded apex, hindwing with apex slightly acute; venation not heavy, very slight swelling at furcation of Cu; stigma lightly marked with brown or pale, with four to five brown subcostal crossveins below; longitudinal veins light green, marked with brown at attachments with transverse veins, crossveins; gradates dark brown to black; narrow covering of dark pigmentation on membrane adjacent to dark veins; gradates in parallel series; basal inner gradate sometimes meeting Psm. Forewing 12.8–15.5 mm long, 4.5–5.3 mm wide, (ratio, L : W = 2.9 : 1); height of tallest costal cell 0.9–1.1 mm (cell number 6–7); length of first intramedian cell 0.8–1.1 mm; 11 radial cells (closed cells between R and Rs); third gradate cell 1.4–1.6 mm long, 0.6 mm wide (ratio, L : W = 2.6–3.0 : 1); fourth gradate cell 1.5 mm long, 0.5 mm wide (ratio, L : W = 2.8 : 1); 3–4 Banksian cells (b cells), 4 b’ cells; 5–6 inner gradates, 7–8 outer gradates. Hindwing 11.2–13.9 mm long, 4.3–4.4 mm wide (ratio, L : W = 3.0–3.2: 1), 10–11 radial cells, 3 Banksian (b) cells, 4 b’ cells, 4–5 inner gradates, 6–7 outer gradates.

Male. Abdomen with small spiracles (e.g., A7: spiracle diameter ~0.05× length of sternite); A7-A9 with numerous setae, those extending from pleural region robust; without microtholi; T9+ectoproct relatively long (~0.5× length of T7), with dorsal invagination deep (~0.5× dorsal length of T9+ect), margins of invagination almost straight, base rounded; dorsal margin of T9+ect (above anus) straight with acute angle at tip; posterior margin of ectoproct straight throughout; ventral margin of T9+ect straight, with sclerotized, curved apodeme, contiguous with sclerotization around callus cerci, posterior corner of apodeme slightly bent mesally in small, rounded corner; posteroventral corner of T9+ect appearing angular (lateral view); callus cerci large, ovate, with entire margin well sclerotized. S8+9 fused, with line of fusion faintly perceptible; dorsal margin with heavy apodeme extending along entire length of segment; dorsal margin gradually sloping ventrally from base of segment approximately to level of callus cerci, then bending to flat terminal plate; terminus extending distally, well beyond tip of T9+ect, heavily sclerotized, distal edge of plate turned up slightly, flat in posterior view; terminal setae on extreme upper layer of posterodorsal margin of S8+9 enlarged, with small, flange-like protrusions basally. Gonarcus arcuate, with slight V-shape (frontal, caudal views); bridge robust, moderately wide, rounded throughout; arms elongate, extending backwards away from mediuncus, rounded distally, mesal section with digitiform process extending posteriorly toward gonosaccus; mediuncus with moderately wide base, paired internal rods flared basally, distally, adjacent mesally; dorsal surface of mediuncus smooth, slightly depressed distally, with short, rounded (blunt) beak distally; gonosaccus bilobed, each lobe large, with large, dense patch of gonosetae; gonosetae robust, arising from enlarged setal bases; hypandrium internum not found.

Female. See Sosa (2015).

##### Known distribution.

COLOMBIA (central): Department of Colombia. VENEZUELA (northeastern): States of Trujillo, Táchira, Mérida, Lara.

##### Specimens examined

[in addition to those listed by Sosa (2015)]. Colombia. *Cundinamarca*: Chía, 30.vi.1998, M. Pérez (1M, USNM). Venezuela. *Táchira*: Quebrada Los Mirtos, 8 km S El Cobre, 7°58.593'N, 72°04.515'W, el. 2400 m, 22.iv.1995, Holzenthal, Cressa, Gutic (1M, 1F, UMSP); *Mérida*: 9 km NE Mérida, Valle Grande, 15.vii.1991, C. Porter & L. Stange (1F, FSCA); Tabay, 2,200 m, 31.iv.1981, H. K. Townes (1?, FSCA); *Lara*: Cubiro, 6.v.1981, H. K. Townes (1F, FSCA).

In the original description of *U. nigromaculifrons*, a specimen from Lara State in Venezuela was reported as a paratype (Sosa: 2015); we now believe that this specimen was misidentified. It is a species that resembles *U. bolivari* (see above).

#### Ungla pallescens

Taxon classificationAnimaliaNeuropteraChrysopidae

Penny, 1998

**Figure 68.**
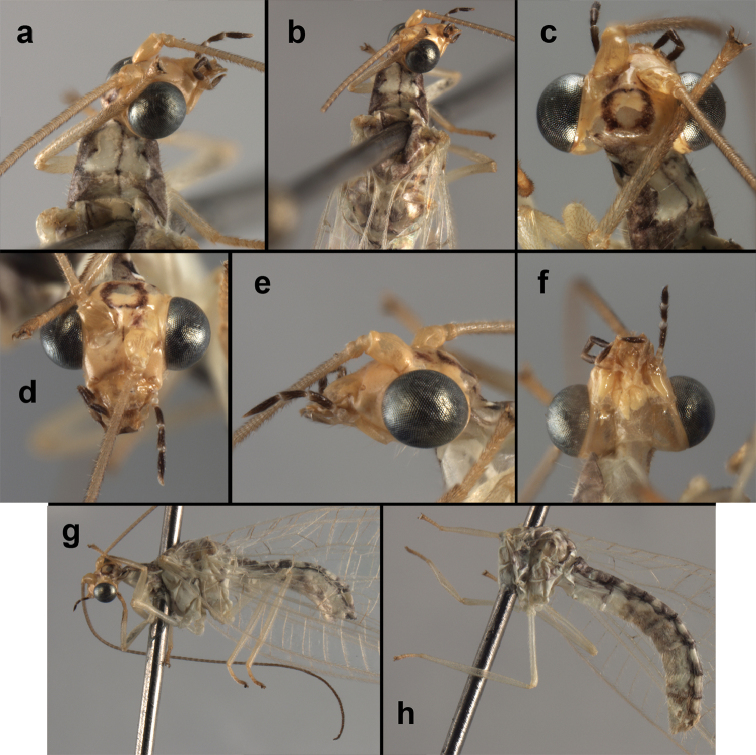
*Ungla pallescens* Penny: External features, (**a**) head, prothorax, dorsolateral, dorsal (**b**) head, thorax, dorsal (**c**) head, dorsal (**d**) head, frontal (**e**) head, lateral (**f**) head, ventral (**g, h**) body, lateral (all: Costa Rica, Monteverde, paratypes, CAS; **a–g** female; **h** male, slightly teneral).

**Figure 69.**
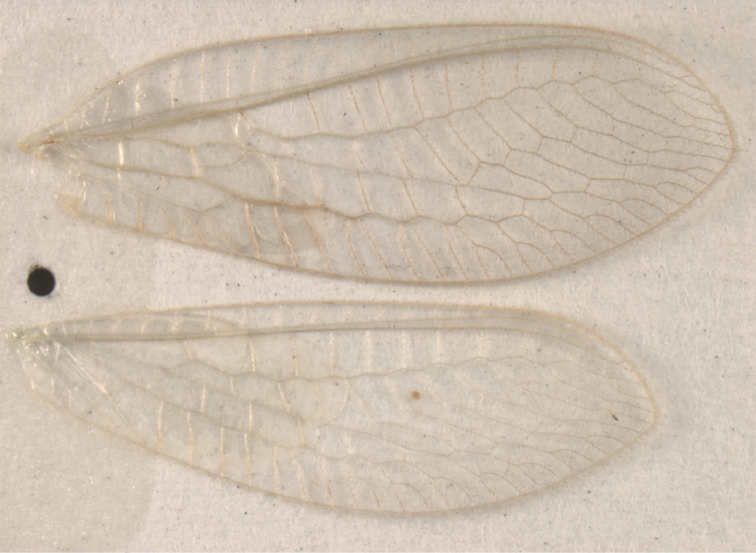
*Ungla pallescens* Penny: Wings (Costa Rica, Monteverde, paratype, male, CAS).

**Figure 70.**
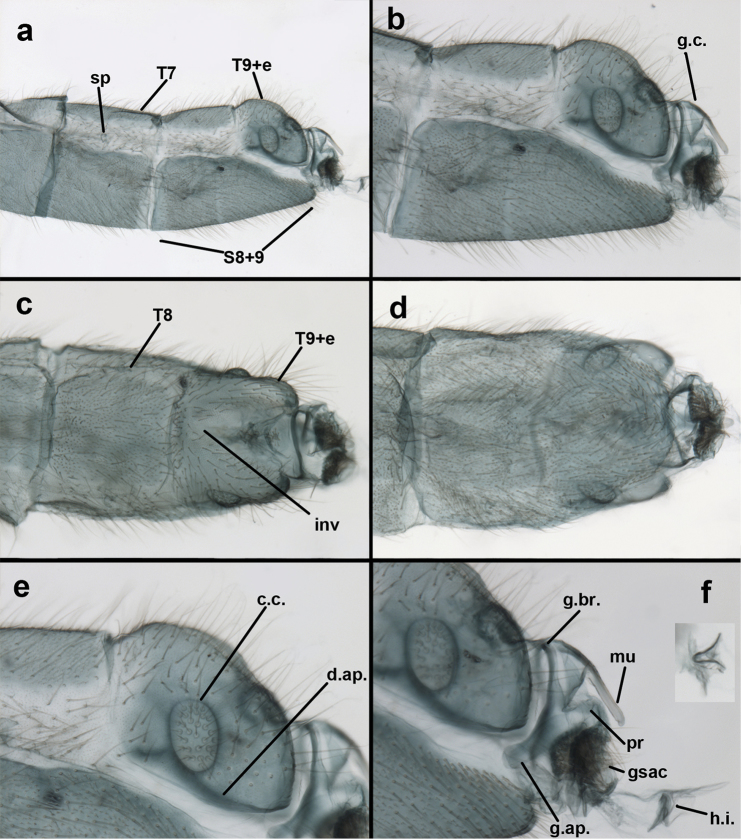
*Ungla pallescens* Penny: Male abdomen and genitalia (slightly teneral), (**a**) segments A7-terminus, lateral (**b**) segments A8-terminus, lateral (**c**) segments A8, T9+ectoproct, dorsal (**d**) sternite 8+9, ventral (**e**) T9+ectoproct, lateral (**f**) gonarcal complex, lateral [insert: hypandrium internum, dorsal; same scale as gonarcus]. **c.c.** callus cerci **d.ap.** apodeme on ventral margin of T9+ectoproct **gsac** gonosaccus **g.ap.** gonarcal apodeme **g.br.** gonarcal bridge **g.c.** gonarcal complex **h.i.** hypandrium internum **inv** invaginated dorsal cleft in T9+ectoproct **mu** mediuncus **pr** unarticulated process on frontal margin of gonarcal apodeme **sp** spiracle **S8+9** fused eighth and ninth sternites **T7, T8** seventh and eighth tergites **T9+e** ninth tergite + ectoproct (Costa Rica, Monteverde, paratype, CAS).

Figs 68 , 69 , 70 , 143h , 143i

Ungla pallescens Penny, 1998. Journal of Neuropterology 1: 69–72; “COSTA RICA: Puntarenas Province, Monteverde, 18.IX.1990, N. D. Penny”. Penny 2002: 227 (tax); Oswald 2015 (catalog). **Holotype.**INBio, male (not examined); holotype by original designation. Allotype and male paratype in CAS (examined) (Figs 68–70; labels: Fig. 143h, i). Originally, this species was described from a series of specimens collected in Costa Rica by N. D. Penny; these remain the only specimens thus far reported for this species.

##### Diagnosis.

At this time, *U. pallescens* is the only *Ungla* species known to lack a genal mark; all other described *Ungla* species have a mark (either large or small) on the gena. The *U. pallescens* adult body is pale green, with large white areas and subtle, pale brown markings; the flagella are cream-colored basally and brownish distally; and the wing venation, including the gradate veins, is entirely light green (Fig. 69; also see Penny 1998, 2002).

##### Description.

See Penny (1998). Head. Measurements: head width: 1.5 mm; ratio head width : eye width: 0.6 : 1; prothorax width: 0.75 mm, length: 1.0 mm.

Forewing, hindwing delicate; forewing rounded apically; hindwing tapered, but rounded apically; membrane entirely clear, without markings or fumose areas; stigma clear; all veins light green, unmarked. Forewing with Rs straight; im1 ovate; first gradate vein meeting Psm; longitudinal veins (especially base of Rs, Psc of the male) slightly thicker than other veins. Forewing 13.1–13.2 mm long, 4.8 mm wide (ratio, L : W = 2.8 : 1); height of tallest costal cell 1.0 mm (cell number 8); width of first intramedian cell 1.0 mm; 11–12 radial cells (closed cells between R and Rs); third gradate cell 1.6 mm long, 0.4 mm wide (ratio, L : W = 4.1 : 1); fourth gradate cell 1.4 mm long, 0.4 mm wide (ratio, L : W = 3.5 : 1); 4 Banksian cells (b cells), 4 b’ cells; 5–6 inner gradates, 7–8 outer gradates. Hindwing 11.9–12.1 mm long, 3.9 mm wide (ratio, L : W = 3.1 : 1), 11 radial cells, 3 Banksian (b) cells, 4 b’ cells, 5 inner gradates, 7 outer gradates.

Male (paratype, slightly teneral). Abdomen with unenlarged spiracles (e.g., A7: spiracle diameter ~0.06× length of sternite); subanal plate obscured; T9+ectoproct moderately long (~0.5× length of T7), with dorsal invagination deep (~0.8× dorsal length of T9+ect), margins of invagination almost straight, base rounded; dorsal margin of T9+ect convex, curving downward, melding into descending distal margin; posterior margin of ectoproct slightly rounded, tip subacute, without protruding knob; ventral margin of T9+ect convex, with distinct, curved apodeme extending along entire length; posterior margin of T9+ect straight; callus cerci large, ovate, margin sclerotized throughout; sclerotization contiguous with that on ventral margin of ectoproct. S8+9 fused, with line of fusion apparent; dorsal margin (lateral view) without apodeme, with very slight taper, small hump, then stronger taper distally; terminus rounded (lateral view), slightly convex (ventral view), not extending much beyond T9+ect; terminal setae long, some along upper lateral edge of S8+9 with small flanges, otherwise simple. Gonarcus (teneral) arcuate, U-shaped, with bridge slender, curved throughout, arms elongate, extending ventrobasally from gonarcal bridge, rounded distally, mesal section with elongate digitiform process extending posteromesally; mediuncus broad basally, tapering to straight, rounded projection, with pair of internal rods extending length of projection; tip of mediuncus rounded, with slight knob; gonosaccus bilobed (mostly unexpanded in the paratype), each lobe with large maleable patch of elongate gonosetae arising from enlarged setal bases, facing medially (not as in Fig. 52 of Penny 1998: 71, but see his description); hypandrium internum V-shaped with rounded apex, arms curved distally.

##### Known distribution.

COSTA RICA: Province of Puntarenas. PERU: Province of Lambayeque [tentative].

##### Specimens examined.

Two paratypes with same data as holotype (1M, 1F, CAS). Peru. Lambayeque: 28 km E. Olmos, Marañón Hwy. Rest. El Salvador, Alt. 1150m, 4/I/1964, P. J. Hutchison & J. K. Wright (M, teneral, CAS).

Note 1: The above male specimen from Peru expresses almost all of the characteristics that distinguish *U. pallescens* (gena unmarked, distal palpomeres dark, abdominal spiracles small, venation pale and genital characteristics). The one exception is that the markings on the vertex are small and spot-like. Unfortunately, the wings are broken at about midlength and the genitalia are teneral (as they are on the male paratype of *U. pallescens* that we studied). Thus, at this time we consider our identification of this specimen (and the range extension for the species) as tentative.

**Figure 71.**
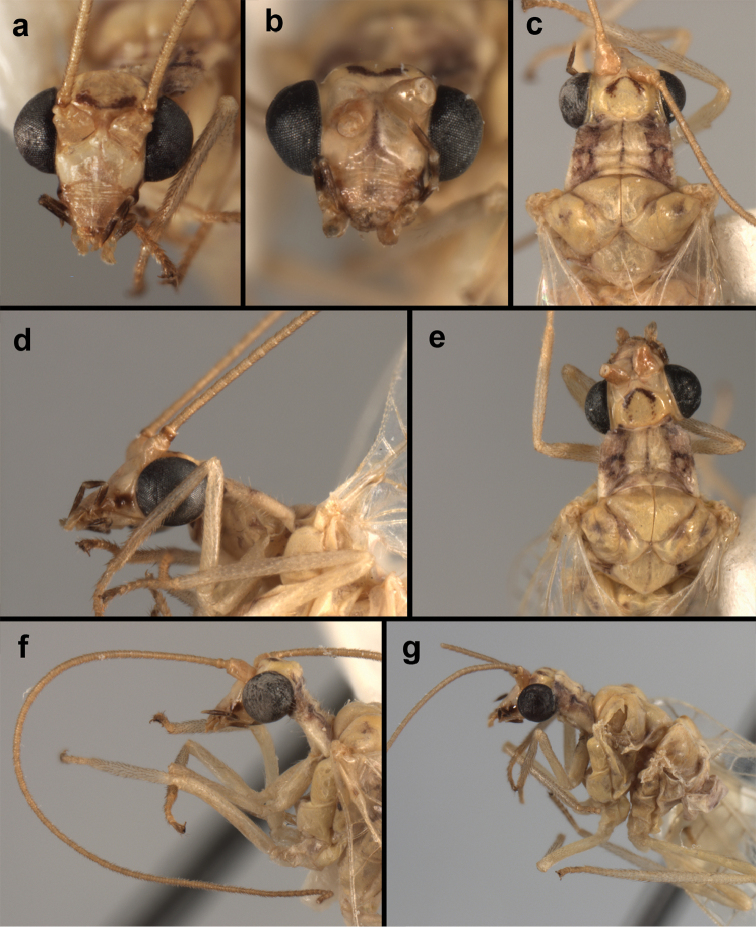
Ungla species near pallescens Penny: External features, (**a, b**) head, frontal (**c**) head, prothorax, dorsal (**d**) head, prothorax, lateral (**e**) head, partial thorax, dorsal (**f**) head, partial thorax, lateral (**g**) head, thorax, lateral (Bolivia, Cochabamba, female, FSCA).

**Figure 72.**
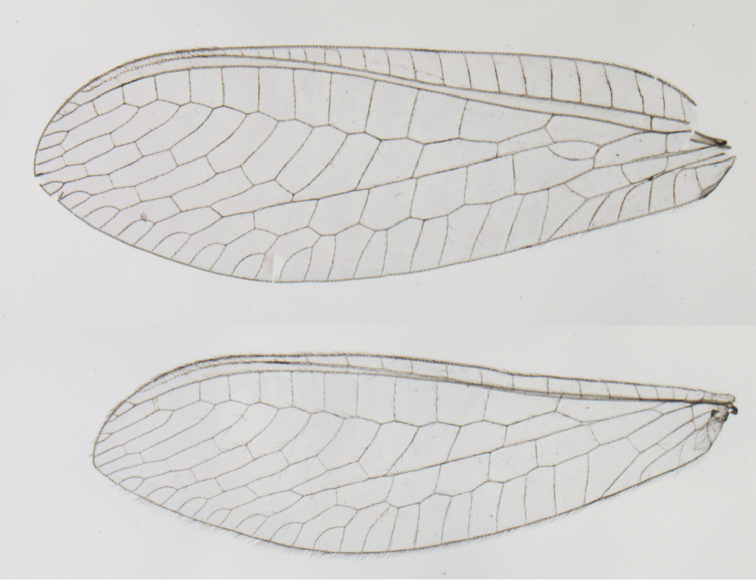
Ungla species near pallescens Penny: Wings (Bolivia, Cochabamba, female, FSCA).

Note 2: We have seen female specimens from Bolivia that resemble *U. pallescens* or perhaps *U. chacranella* in their pale, lightly colored bodies, green venation, size, etc. (see Figs 71, 72). However, the gena has a very distinct dark brown/black stripe, and the pedicel has a brown ring around the circumference – both characteristics lacking from *U. pallescens*; the genal mark is present on *U. chacranella*, but not the ring on the pedicel. Unfortunately, there were no male specimens in the series from Bolivia, and we were unable to identify them. The locality data and depository for these specimens are: Bolivia, Cochabamba, 5.iii.1981, D. Foster, blacklight (11F, FSCA).

#### Ungla pennyi

Taxon classificationAnimaliaNeuropteraChrysopidae

Tauber sp. n.

http://zoobank.org/7B1D6459-1AF5-44C5-9C97-80A3B8DB3DDB

**Figure 73.**
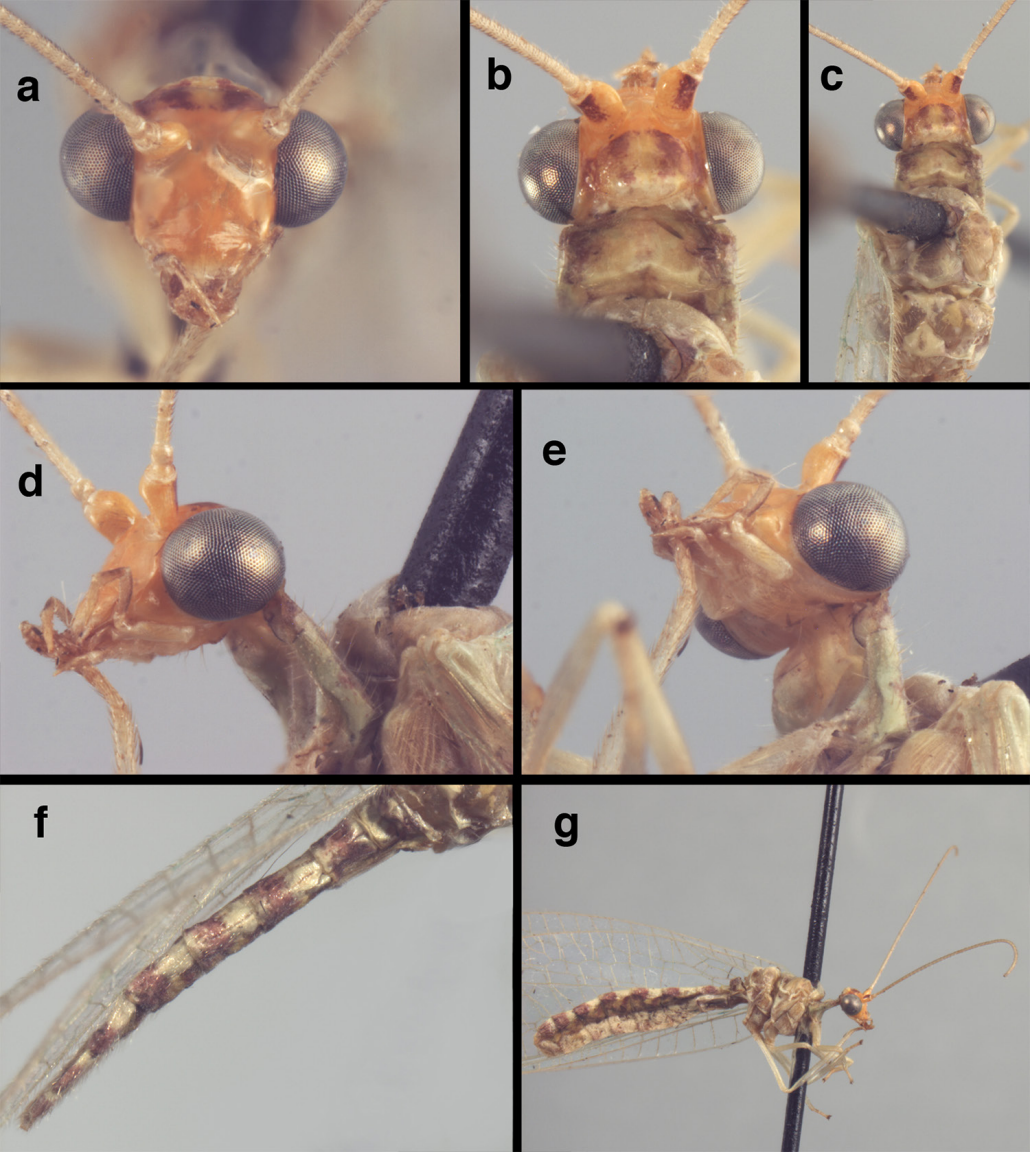
*Ungla pennyi* Tauber, sp. n. External features, (**a**) head, frontal (**b**) head, prothorax, dorsal (**c**) head, thorax, dorsal (**d**) head, lateral (**e**) head, ventral (**f**) abdomen, dorsal (**g**) body, lateral (Costa Rica, Puntarenas, paratype, female, CAS).

**Figure 74.**
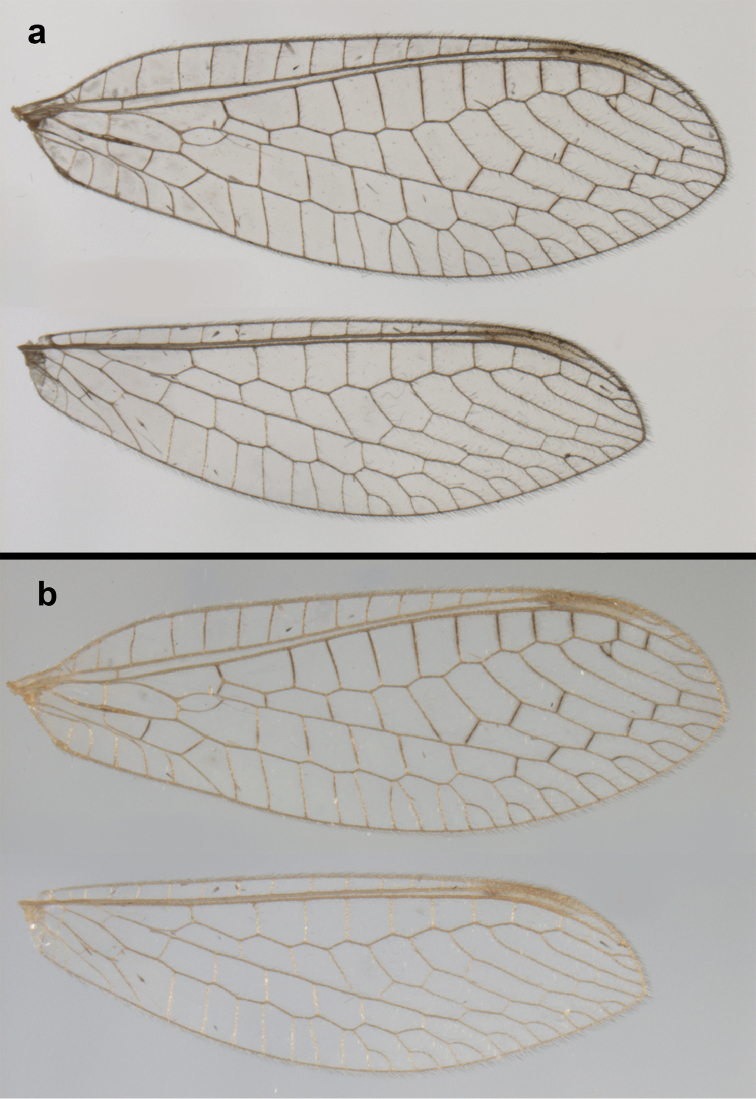
*Ungla pennyi* Tauber, sp. n. Wings, (**a**) venation emphasized (**b**) coloration of veins emphasized (Costa Rica, Cartago, holotype, male, FSCA).

**Figure 75.**
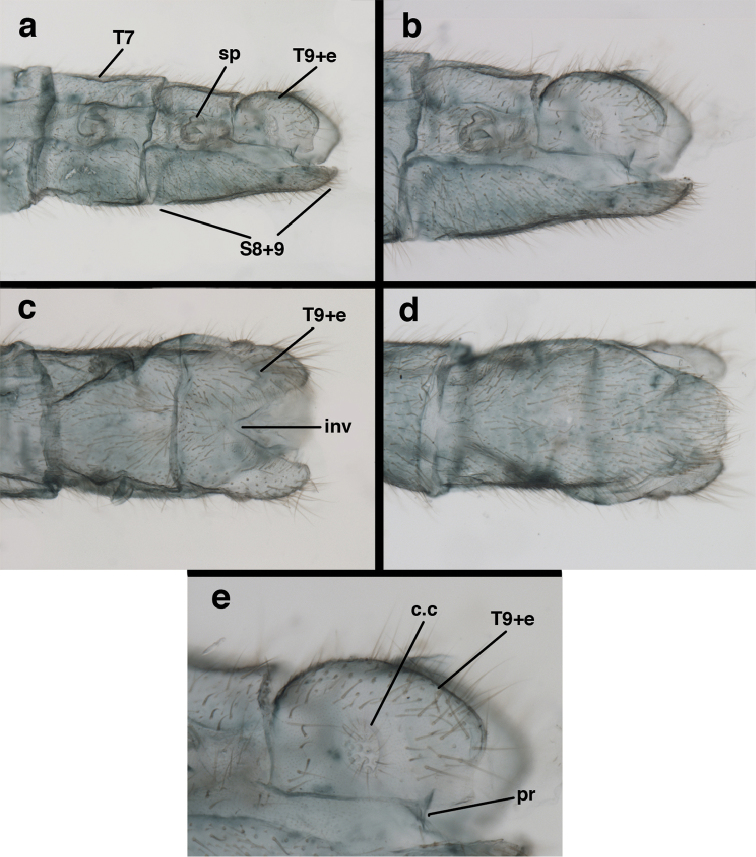
*Ungla pennyi* Tauber, sp. n. Male abdomen, (**a**) segments A7-terminus, lateral (**b**) segments A8-terminus, lateral (**c**) segments A8, T9+ectoproct, dorsal (**d**) sternite 8+9, ventral (**e**) T9+ectoproct, lateral. **c.c.** callus cerci **inv** invaginated dorsal cleft in T9+ectoproct **pr** triangular process **sp** spiracle **S8+9** fused eighth and ninth sternites **T7** seventh tergite **T9+e** ninth tergite + ectoproct (Costa Rica, Cartago, holotype, FSCA).

**Figure 76.**
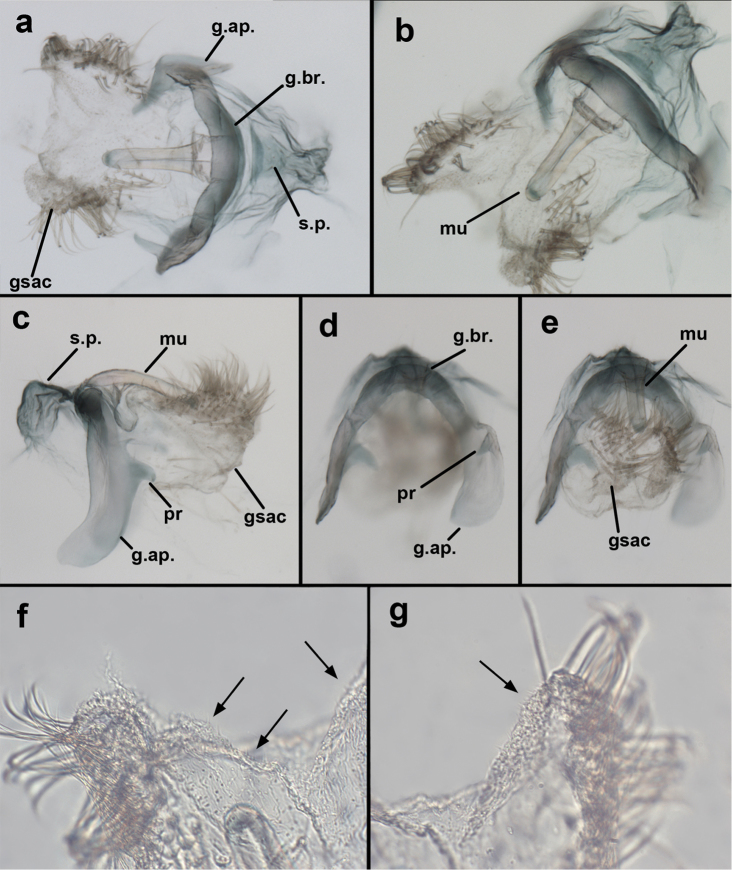
*Ungla pennyi* Tauber, sp. n. Male genitalia, (**a**) gonarcal complex, dorsal, with gonosaccus fully expanded (**b**) gonarcus, dorsal [Note internal structure of mediuncus.] (**c**) gonarcus, lateral (**d**) gonarcus, frontal, with mediuncus, gonosaccus obscured, position of left gonarcal apodeme slighltly distorted (**e**) gonarcus, frontal, with mediuncus, gonosaccus visible (**f, g**) inner, opposing (spiny) surfaces of gonosaccus lobes [Arrows indicate spines on surface of lobes.]. **gsac** gonosaccus **g.ap.** gonarcal apodeme **g.br.** gonarcal bridge **mu** mediuncus **pr** unarticulated process on frontal margin of gonarcal apodeme **s.p.** setose subanal plate (Costa Rica, Cartago, holotype, FSCA).

Figs 73 , 74 , 75 , 76 , 144d

##### Holotype

(Figs 74–76, 144d). FSCA, male. Costa Rica, Cartago Province, Tuis, 16-29.vii.1987, H. L. Dozier.

##### Etymology.

The species is named for Norman D. Penny of the California Academy of Sciences, who died during the preparation of this revision. Norm was a world-renown Neuropterist and valued colleague. We (CAT) had some disagreements with Norm, but our shared attachment to the Neuroptera stimulated us to overcome our differences. His work, especially on the Costa Rican and Amazonian chrysopids, has been very useful in this and our other studies.

##### Diagnosis.

*Ungla pennyi* is one of three *Ungla* species reported from an area north of South America; the other two species are *U. pallescens* from Puntarenas, Costa Rica, and *U. mexicana*, sp. n., described above, from Chiapas, Mexico. *Ungla pennyi* differs from these two species in its smaller size (forewing length = 10.9 mm, vs 13.1–14.1 mm for *U. mexicana* and *U. pallescens*), and cream-colored, unmarked palpi. Also, in contrast to *U. pallescens*, the male *U. pennyi* has enlarged abdominal spiracles. [The male of *U. mexicana* is unknown.].

The *U. pennyi* holotype closely resembles specimens of the Venezuelan species *U. curimaguensis* and *U. martinsi*, in size and especially in the male abdominal and genital characteristics. Thus, it is possible that our specimen represents a differentiated population of one of these two species. However, it lacks the dark genal marks and marked maxillary and labial palpi of both species, and the largely green forewing venation of *U. curimaguensis*. In *U. curimaguensis*, the C-Sc crossveins, R-Rs crossveins and the transverse branches from the Rs are entirely green, whereas in *U. pennyi* and in *U. martinsi* they are dark brown with surrounding membrane suffused with brown coloration. At this time, we consider that the set of features expressed by the *U. pennyi* specimen indicate species-level differentiation; we await confirmation or correction of our opinion when additional specimens from Central America become available.

##### Description.

Head badly discolored, probably cream-colored with red markings; vertex smooth anteriorly, shiny, with a pair of red spots in place of U-shaped marking, pair of larger red marks around posterolateral margin of raised vertex; dorsal fossae either pale or reddish; no apparent markings between antennae; frons with hint of mesal red spot below each antenna; reddish marking(s) near front of frons; gena without visible coloration; clypeus, labrum without marks. Antenna: dorsal surface of scape with broad red longitudinal stripe laterally, frontal surface with indistinct dark markings mesally; pedicel, flagellum pale, with pale setae basally, darker setae distally; maxillary, labial palpi pale, without discernible marks.

Thorax without distinct coloration or marks; prothorax short, with pale setae; mesothorax perhaps with pair of dark spots on frontal surface of scutum. Legs pale, unmarked, with pale setae. Measurements: head width: 1.3 mm; ratio head width : eye width: 2.4 : 1; prothorax width: 0.9 mm, length: 0.5 mm.

Forewing rounded apically; hindwing slightly acute, both with venation robust; membrane mostly clear, dull; stigma opaque, brownish, with three faint subcostal crossveins, basal one with brown suffusion. Forewing: 10.9 mm long, 3.7 mm wide (ratio, L : W = 3.0 : 1); Rs fairly straight; first intramedian cell ovate; basal inner gradate meeting Psm; height of tallest costal cell 0.6 mm (cell number 5); width of first intramedian cell 0.7 mm; 10 radial cells (closed cells between R and Rs); third gradate cell 1.1 mm long, 0.4 mm wide (ratio, L : W = 2.9 : 1); fourth gradate cell absent; 4 Banksian cells (b cells), 4 b’ cells; 3 inner gradates, 5 outer gradates. Forewing veins discolored with age, but probably green (pale) and brown; dark brown veins usually with surrounding membrane suffused with brown; longitudinal veins pale; distal, basal C-Sc crossveins pale, mesal C-Sc crossveins either dark brown or brown near Sc; R-Rs crossveins dark brown to brown; transverse branches from Rs surrounding b cells entirely brown or brown only near Psm; distal transverse branches light brown near Rs, paler distally; veins between Psm-Psc brown or marked with brown; base of Rs, gradate veins, basal intracubital crossvein dark. Hindwing 9.7 mm long, 2.9 mm wide (ratio, L : W = 3.3 : 1), 10 radial cells, 3 Banksian (b) cells, 4 b’ cells, 3 inner gradates, 5 outer gradates; venation entirely pale (probably light green).

Male. Abdomen with very large spiracles (e.g., A7: spiracle diameter ~0.25× length of sternite); subanal plate small, with ~4 setae; T9+ectoproct long (~0.7 length of T7), with dorsal invagination deep (~0.6x dorsal length of T9+ect), margins of invagination almost straight, base acute; dorsal margin of T9+ect rounded, convex, curving downward, melding into rounded distal margin; posterior margin of ectoproct rounded, protruding, basal knob blunt, curving inward; ventral margin of T9+ect straight, with narrow apodeme extending along entire length, apodeme forming triangular process distally, extending posteriorly as short rod; posterior margin of T9+ect straight; callus cerci large, ovate, margin sclerotized throughout, but not darkened; sclerotization appearing contiguous with that on ventral margin of ectoproct. S8+9 fused, with line of fusion not perceptible; dorsum tapering slightly anteriorly, forming concave depression at about 3/4^th^ distance to tip; distal one forth rounded, forming concave platform; terminus blunt (lateral view), extending distally well beyond T9+ect; most terminal setae long, simple, a few along upper lateral edge of S8+9 with small flanges. Gonarcus arcuate, U-shaped, with bridge robust, curved throughout, arms elongate, extending ventrobasally from gonarcal bridge, rounded distally, mesal section with short digitiform process extending posteromesally; mediuncus partially rounded, long, curving downward from top of bridge, ending in blunt knob, internally with pair of adjacent sclerotized rods, broad basally, tapering mesally, then fusing distally; gonosaccus bilobed, each lobe with large maleable patch of large gonosetae arising from enlarged setal bases, facing outward (everted), more flexible inner membrane with large, dense patch of spines, facing inward; hypandrium internum V-shaped, with rounded apex, small comes.

##### Known distribution.

COSTA RICA: Provinces of Cartago, Puntarenas.

##### Specimens examined

(in addition to holotype above). Puntarenas, Costa Rica: Finca Las Cruses near San Vito, Puntarenas Province, Costa Rica 1800m elev. 13.III.69 at black light, J. Sheldon #1969-140 (paratype, F, CAS).

#### Ungla quchapampa

Taxon classificationAnimaliaNeuropteraChrysopidae

Tauber sp. n.

http://zoobank.org/B8D8847A-9D88-4128-9BA4-9CC1FDC4131A

**Figure 77.**
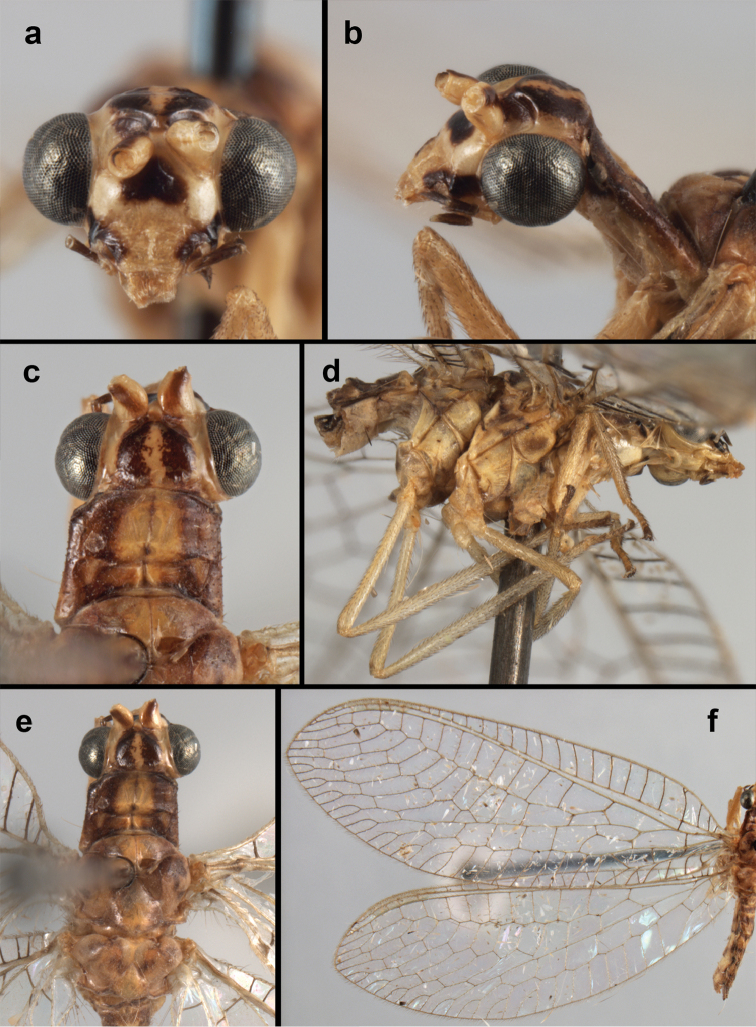
*Ungla quchapampa* Tauber, sp. n. External features, (**a**) head, frontal (**b**) head, prothorax, lateral (**c**) head, prothorax, dorsal (**d**) thorax, lateral (**e**) head, thorax, dorsal (**f**) left wings (Bolivia, Cochabamba, holotype, male, MCZ).

**Figure 78.**
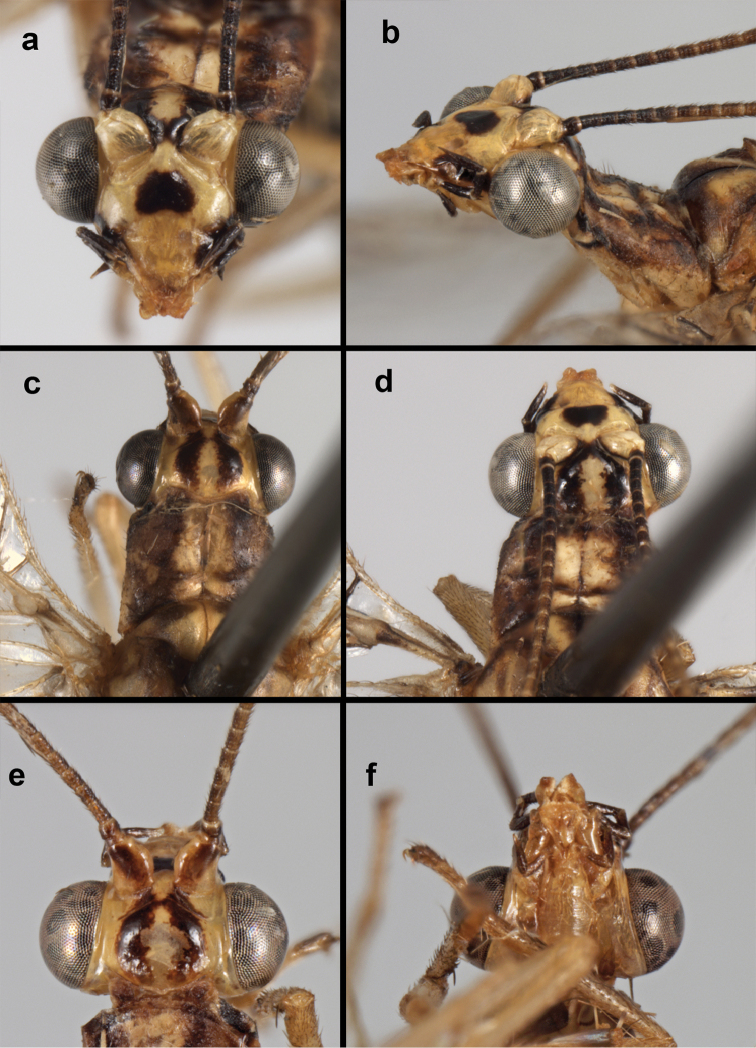
*Ungla quchapampa* Tauber, sp. n. External features, (**a**) head, frontal (**b**) head, prothorax, lateral (**c**) head, prothorax, dorsal (**d**) head, thorax, dorsal (**e**) head, dorsal (**f**) head, ventral (Bolivia, Cochabamba, paratypes, male, CAS).

**Figure 79.**
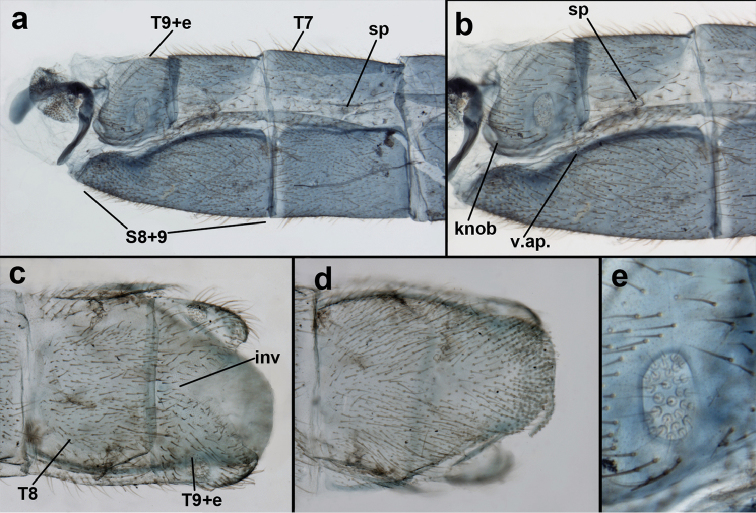
*Ungla quchapampa* Tauber, sp. n. Male abdomen, (**a**) segments A7-terminus, lateral (**b**) segments A8-terminus, lateral (**c**) tergite 8, tergite 9+ectoproct, dorsal (**d**) sternite 8+9, ventral, with heavy setae (**e**) callus cerci. **inv** invaginated dorsal cleft in T9+ectoproct **sp** spiracle **S8+9** fused eighth and ninth sternites **T7, T8** seventh and eighth tergites **T9+e** ninth tergite + ectoproct **v.ap.** apodeme on dorsal margin of S8+9 (Bolivia, Cochabamba, holotype, MCZ).

**Figure 80.**
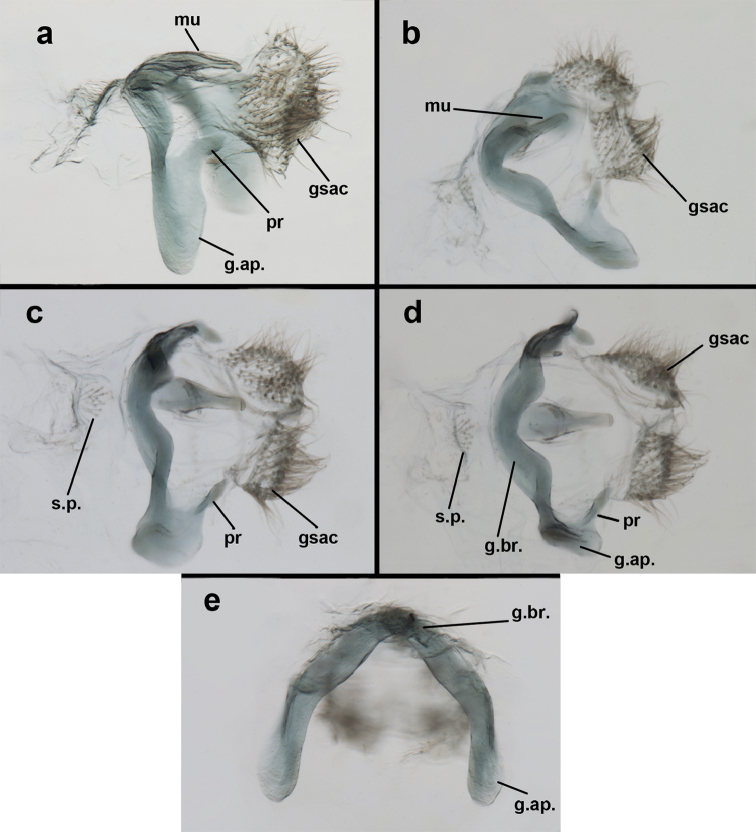
*Ungla quchapampa* Tauber, sp. n. Male genitalia, (**a**) gonarcal complex, frontolateral, with gonosaccus expanded (**b**) gonarcus, dorsolateral, with gonosaccus fully expanded [Note close connection between mediuncus and gonarcal bridge.] (**c, d**) gonarcus dorsal, gonosaccus expanded (**e**) gonarcus, posterior, gonosaccus in distance. **gsac** gonosaccus **g.ap.** gonarcal apodeme **g.br.** gonarcal bridge **mu** mediuncus **pr** unarticulated process on frontal margin of gonarcal apodeme **s.p.** setose subanal plate (Bolivia, Cochabamba, holotype, MCZ).

Figs 77 , 78 , 79 , 80 , 144e

##### Holotype

(Figs 77, 79, 80, 144e). Male (MCZ). Bolivia, Cochabamba, Chapare, Steinbach.

##### Etymology.

The type locality of this species is the Andean valley of Cochabamba in central Bolivia. The locality is also known by its Quechua name – Quchapampa. Both the Spanish and the Quechua names are derived from the Quechua words “qhucha” (meaning “small lake”) and “pampa” (meaning “open plain”) (Ajacopa 2007). We use the name “quchapampa” as a compound, proper noun (Latinized, fem.), in apposition to the genus name.

##### Diagnosis.

*Ungla quchapampa* is one of several species that has a prominent dark mark in the center of the frons, but it is the only one that has a dark flagellum and also lacks a prominent protuberance on the frons. The size and shape of the marks on the vertex are also distinctive: they are very large, dark brown, contiguous with each other, and extend anteriorly to the mesal margins of the antennal base. In more than half of the specimens we examined, the first intramedian cell (im1) of the forewing was quadrate (unlike the commonly triangular im1 cells of other *Ungla* species).

*Ungla quchapampa* closely resembles *U. diazi* in many features – notably size, wing features, and male abdominal characters (external and internal). In addition, the two species are known to occur sympatrically; however, *U. quchapampa* can be differentiated by its prominent frontal marking, sometimes quadrate im1 cell in the forewing, and the absence of markings on the ventral surface of the scape.

##### Description.

Head cream-colored with dark brown to black markings; vertex with inverted U-shaped marking large, robust, with lateral arms meeting anteriorly, extending forward into dorsal fossa, between antennae; mesal side, posterior margin of dorsal fossa marked with dark brown; posterior parts of vertex unmarked; frons with large, prominent mesal mark; clypeus with lateral margin black; tentorial pits surrounded by dark brown; gena with black stripe from eye, contiguous with black mark on clypeal margin. Antenna: scape cream-colored, dorsum with large, brown longitudinal mark; pedicel, flagellum dark brown to black basally, becoming lighter distally, with cream-colored intersegmental membrane; maxillary palp with basal two segments pale, three distal segments black, articulations pale; labial palp with basal segment pale, distal two segments marked with black.

Thorax with distinct longitudinal cream-colored stripe mesally; prothorax with pair of broad, dark reddish brown stripes laterally; transverse furrow in posterior region of segment, extending to lateral margins; setae mostly elongate, pale. Measurements: head width: 1.3–1.5 mm; ratio head width : eye width: 2.1–2.6 : 1; prothorax width: 1.1–1.2 mm, length: 0.6–0.7 mm.

Forewing, hindwing somewhat broad, apex round; membrane clear, hyaline, without fumose area; stigma slightly opaque to light brown; longitudinal veins green, marked with brown at intersections with transverse veins; transverse veins mostly brown or marked with brown; forewing with veins robust, not crassate; Rs straight; first intramedian cell ovate or quadrate; basal inner gradate either meeting Psm or not; gradate veins, subcostal crossveins below stigma dark brown, costal crossveins, R-Rs crossveins, intracubital crossveins entirely brown; hindwing with costal crossveins, subcostal crossveins, gradate veins brown. Forewing 13.0–14.3 mm long, 4.6–5.1 mm wide [ratio L : W = 2.7–2.8 : 1]; height of tallest costal cell 1.0–1.1 mm (cell number 5–6); width of first intramedian cell 1.2 mm (whether quadrate or ovate); 11–12 radial cells (closed cells between R and Rs); third gradate cell 1.5–1.6 mm long, 0.5 mm wide (ratio, L : W = 3.0–3.4 : 1); fourth gradate cell 1.5–1.6 mm long, 0.4–0.5 mm wide (ratio, L : W = 3.1–3.8 : 1); 3–4 Banksian cells (b cells, sometimes not distinct), 4 b’ cells; 5–7 inner gradates (basal one sometimes not distinct), 7 outer gradates. Hindwing 11.6–13.0 mm long, 3.9–4.3 mm wide (ratio, L : W = 2.9–3.0 : 1), 10–11 radial cells, 3 Banksian (b) cells, 4 b’ cells, 5–6 inner gradates, 6–7 outer gradates.

Male. Abdomen with small spiracles (e.g., A7: spiracle diameter ~0.04× length of sternite); subanal plate substantial, with ~20 setae in a well defined group; T9+ect relatively long (~0.6× length of T7), with dorsal invagination deep (~0.7× dorsal length of T9+ect), margins of invagination almost straight, base acute rounded; dorsal margin of T9+ect short, straight, angled downward to round distal tips; posterior margin of ectoproct rounded, protruding somewhat, posteroventral corner rounded but with small distal projection of apodeme, small knob; ventral margin bent, with moderately heavy apodeme except anteriorly; callus cerci large, ovate, margin sclerotized throughout; sclerotization probably contiguous with that on ventral margin of ectoproct. S8+9 fused, with line of fusion not perceptible; basal three-fourths of dorsal margin with heavy apodeme; dorsum tapering slightly anteriorly, forming concave depression at about 3/4^th^ distance to tip; distal one-fourth forming concave terminus (lateral view), extending distally beyond T9+ect; setae slender, mostly long, simple, probably without flanges (We could not find any flanged setae; however, a number of the distolateral setae were missing). Gonarcus arcuate, V-shaped (frontal view), with bridge robust, bent mesally, arms elongate, extending ventrodistally from gonarcal bridge, rounded distally, mesal section with digitiform process extending posteromesally; mediuncus flat, closely attached to gonarcal bridge, not particularly long, extending distally or downward, ending in blunt, bent knob; bilobed gonosaccus, with lobes well separated, each lobe cylindrical, with rigid patch of large gonosetae facing mesally when uneverted, outwardly when everted; gonosetae arising from enlarged setal bases; hypandrium internum not found.

##### Known distribution.

BOLIVIA (central): Department of Cochabamba (Provinces of Carrasco and Chapare).

##### Specimens examined

(in addition to holotype). Same data as holotype (1F, paratype, MCZ); Bolivia, Cochabamba, Carrasco, Siberia 1650 m, xxii.1962–i.1963, i.1964, F. H. Walz (5F, all paratypes, CAS). Four of the specimens in the CAS have attached notes handwritten by P. A. Adams.

#### Ungla siderocephala

Taxon classificationAnimaliaNeuropteraChrysopidae

(Navás, 1933) comb. n.

**Figure 81.**
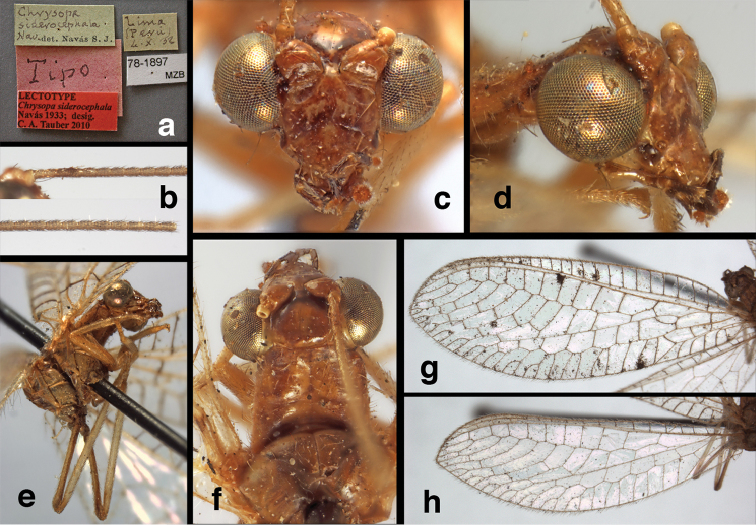
*Chrysopa siderocephala* Navás: External features, (**a**) labels (**b**) antenna, basal above, distal below (**c**) head, frontal (**d**) head, lateral (**e**) thorax, lateral (**f**) head, prothorax, mesothorax (partial), dorsal (**g**) forewing (**h**) hindwing (Peru, Lima, lectotype, male, MZBS).

**Figure 82.**
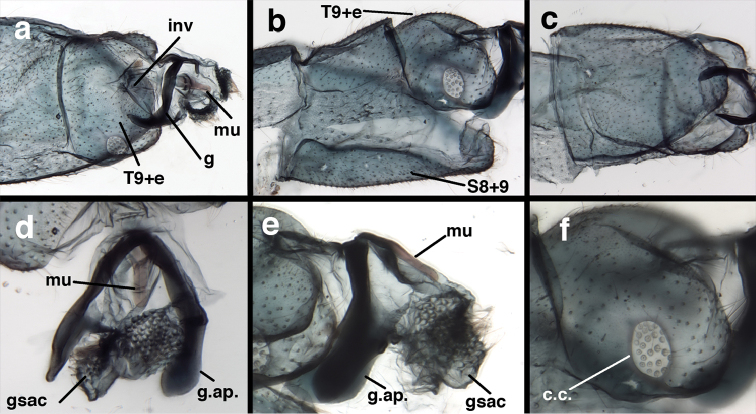
*Chrysopa siderocephala* Navás: Male terminalia, (**a**) terminalia, dorsal (**b**) terminalia, lateral (**c**) fused sternites 8+9, ventral (**d**) gonarcus, frontal (**e**) gonarcus, lateral (**f**) tergite 9 + ectoproct, lateral. **c.c.** callus cerci **g** gonarcus **gsac** gonosaccus **g.ap.** gonarcal apodeme **inv** dorsal invagination of T9+ectoproct **mu** mediuncus **S8+9** fused eighth and ninth sternites **T9+e** fused ninth tergite and ectoproct (Peru, Lima, lectotype, MZBS).

**Figure 83.**
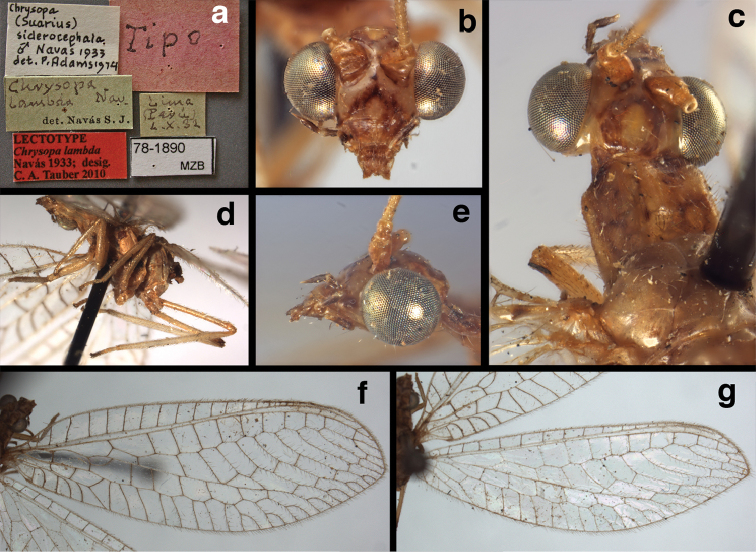
*Chrysopa lambda* Navás: External features, (**a**) labels (**b**) head, frontal (**c**) head, prothorax, dorsal (**d**) thorax, lateral (**e**) head, lateral (**f**) forewing (**g**) hindwing (Peru, Lima, lectotype, male, MZBS).

**Figure 84.**
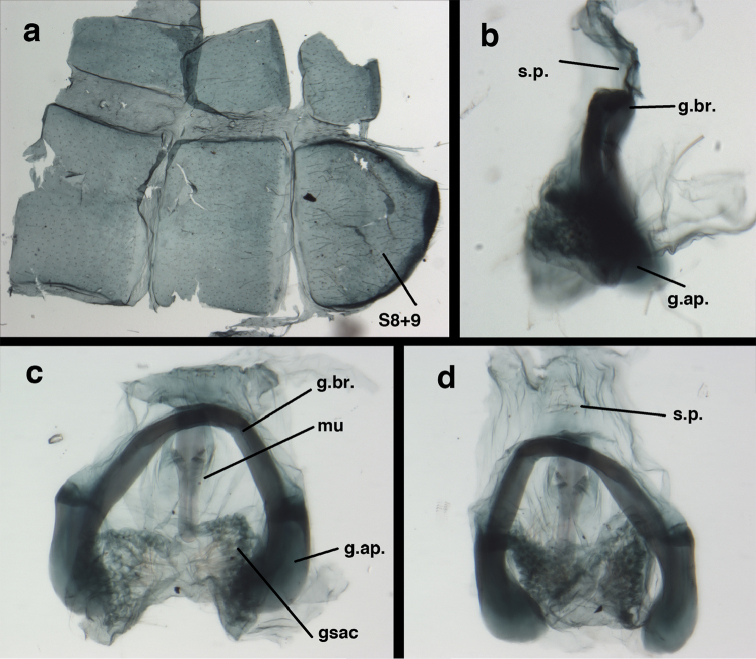
*Chrysopa lambda* Navás: Male terminalia, (**a**) integument of abdominal segments A6-terminus, dissected and flat (**b**) gonarcus, lateral (**c**) gonarcus, frontal (**d**) gonarcus, frontal, showing subanal plate above gonarcal bridge. **gsac** gonosaccus **g.ap.** gonarcal apodeme **g.br.** gonarcal bridge **mu** mediuncus **S8+9** fused eighth and ninth sternites **s.p.** setose subanal plate (Peru, Lima, lectotype, MZBS).

**Figure 85.**
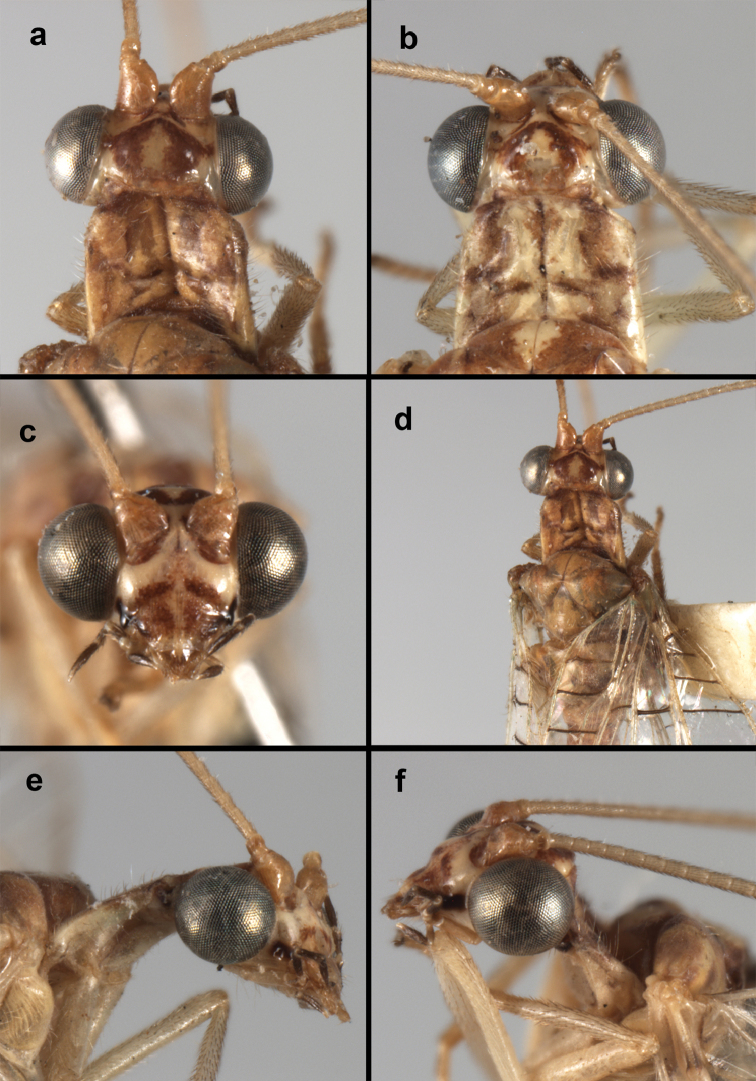
*Ungla siderocephala* (Navás): External features, (**a, b**) head, prothorax, dorsal (**c**) head, frontal (**d**) head, thorax, dorsal (**e, f**) head, prothorax, lateral (all: Peru; **a, d** Huaral, female, CAS; **b** La Samme, female, CAS; **c, e, f** La Samme, male, CAS).

**Figure 86.**
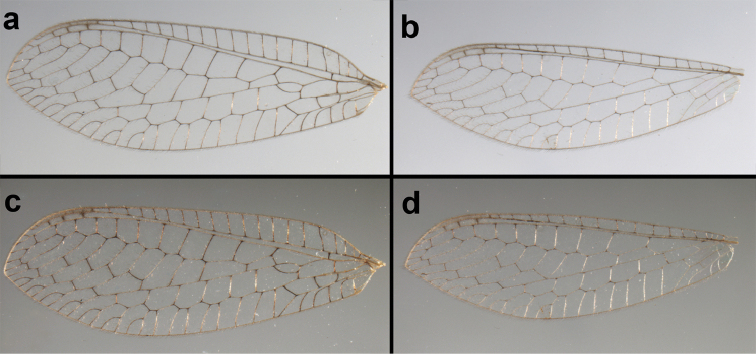
*Ungla siderocephala* (Navás): Wings, (**a, b**) venation emphasized (**c, d**) coloration of veins emphasized (all: Peru, La Samme, female, CAS).

**Figure 87.**
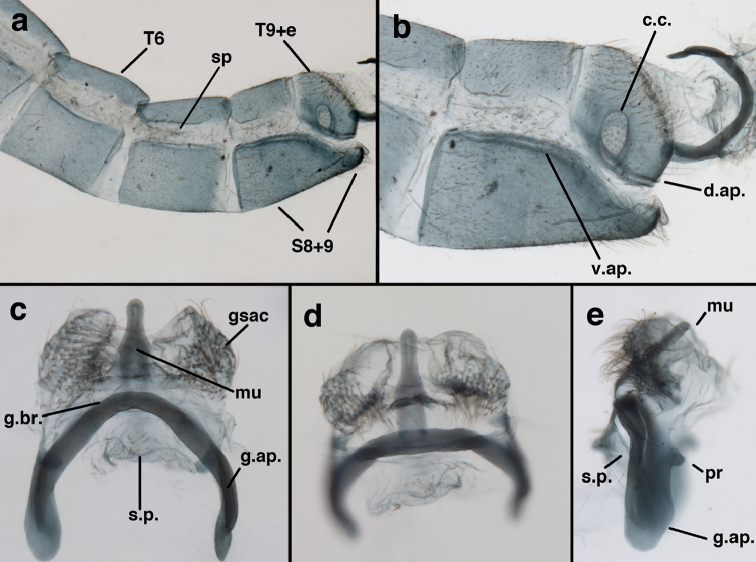
*Ungla siderocephala* (Navás): Male abdomen and terminalia, (**a**) segments A6–terminus, lateral (**b**) terminalia, lateral (**c**) gonarcus, posterodorsal (**d**) gonarcus, dorsal (**e**) gonarcus, lateral. **c.c.** callus cerci **d.ap.** apodeme on ventral margin of T9+ectoproct **gsac** gonosaccus **g.ap.** gonarcal apodeme **g.br.** gonarcal bridge **mu** mediuncus **pr** unarticulated process on frontal margin of gonarcal apodeme **sp** spiracle **S8+9** fused eighth and ninth sternites **s.p.** setose subanal plate **T6** sixth tergite **T9+e** fused ninth tergite and ectoproct **v.ap.** apodeme on dorsal margin of S8+9 (all: Lima, Peru, USNM).

Figs 81 , 82 , 83 , 84 , 85 , 86 , 87

Chrysopa siderocephala Navás, 1933. *Rev. R. Acad. Ciencias exactas fis. Nat. Madrid* (1933a) 30: 306–307; “Perú: Lima, 4.X.1932”. Penny 1977: 20 (list); Monserrat 1985: 238 (type); Brooks and Barnard 1990: 280 (list, as “ ‘Chrysopa’ incertae sedis”); Oswald 2015 (catalog). **Lectotype** (Figs 81, 82). MZBS, male. Specimen somewhat dirty, color faded. Confusion concerning the type is unlikely; however, to assure stability, here the specimen in the MZBS is designated as the lectotype (des. CAT).
Chrysopa lambda Navás, 1933. *Rev. R. Acad. Ciencias exactas fis. Nat. Madrid* (1933a) 30: 307; “Perú: Lima, 4.X.1932”. Penny 1977: 19 (list); Monserrat 1985: 238 (type); Brooks and Barnard 1990: 280 (list, as “ ‘Chrysopa’ incertae sedis”); Oswald 2015 (catalog). **syn. n. Lectotype** (Figs 83, 84). MZBS, male. Specimen discolored, with abdomen in poor condition. Confusion concerning the type is unlikely; however, to assure stability, here the specimen in the MZBS is designated as the lectotype (des. CAT). **Support for synonymy.** The types of both C. siderocephala and C. lambda were collected at the same locality and on the same day, and in his original description, Navás (1933a) noted the similarity between the two. However, the notable variation in the shape of the frontal markings (from an inverted V to an inverted Y) caused Navás to believe that he had more than one species. Additional specimens are now available. Phillip Adams recognized the synonymy and labeled the C. lambda specimen as the junior synonym, but he did not publish his conclusion. Here, we formalize the synonymy. The main structural difference that we noticed between the two specimens is in the intersection of the basal inner gradate with the Psm: on both wings of the C. lambda type the basal inner gradate veins meet the Psm, whereas on the C. siderocephala type they do not. We attribute this difference to intraspecific variation. Otherwise, the venation of the two specimens is almost identical, and the size of the wings is very similar: C. lambda, forewing 12.2–12.3 mm long, 4.2 mm wide, hindwing 11.0–11.1 mm long, 3.3–3.4 mm wide; C. siderocephala, forewing 12.3–12.4 mm long, 4.3–4.4 mm wide, hindwing 11.1–11.3 mm long, 3.3–3.6 mm wide.

##### Diagnosis.

This species is recognized by the following set of features: body mostly cream-colored to green; head cream-colored, vertex with robust reddish brown, inverted U-shaped mark with lateral bar extending to mesal margin of eyes; frons with broad, reddish brown, transverse band above clypeus; antenna with basal two segments reddish, flagellum pale; thorax probably with yellowish mesal stripe dorsally, reddish brown stripe sublaterally, legs pale, unmarked, with pale setae; wings hyaline, lightly fumose around gradate veins, with veins green, except costal crossveins, gradate veins black. In the male, the spiracles are not enlarged; the setae on the pleural membrane of A7, A8 have slightly enlarged bases; the terminus of S8+9 is without flanged setae; and the gonarcus, mediuncus, and gonosaccus are as in Figs 82, 84, 87.

##### Redescription.

Head cream-colored with reddish brown to dark brown markings; vertex smooth, shiny; inverted U-shaped marking prominent, reddish brown, broad, connected anteromesally above antennae, with lateral bar extending to midregion of eye; posterior half of vertex unmarked; dorsal antennal fossa with thin reddish brown stripe mesally, extending between antennae to reddish brown anterior fossa. Frons with narrow, elongate, brown stripe between antennal fossae (“Y-shaped” mark of Navás), anterior margin of frons with pair of heavy reddish brown transverse bands from midregion to genal mark; clypeus with pair of large reddish brown marks, separated mesally, extending to margin laterally; gena with large, dark brown to black mark extending from eyes, contiguous with dark mark on lateral margin of clypeus; tentorial pits surrounded by reddish brown. Antenna with scape, pedicel reddish, unmarked; flagellum cream-colored; maxillary palp, labial palp with two basal segments pale, other segments dark brown.

Prothorax cream-colored mesally, with pair of wide, reddish brown stripes sublaterally, reddish brown extending mesally along transverse furrow; transverse furrow in posterior region, not reaching lateral margins of segment, long, cream-colored to golden setae throughout. Mesothorax, metathorax probably cream-colored mesally, with pair of broad, reddish brown stripes laterally. Legs entirely cream-colored, unmarked. Measurements: head width: 1.3–1.6 mm; ratio head width : eye width: 2.0–2.3 : 1; prothorax width: 0.8–1.1 mm, length: 0.6–0.7 mm.

Forewing, hindwing of moderate width, with apex rounded or slightly subacute. Forewing with robust, but not heavy venation, some swelling at furcation of Cu; stigma lightly opaque, with three dark brown subcostal crossveins below, area surrounding subcostal crossveins marked with dark brown; longitudinal, most transverse veins (costal, radial, cubital crossveins) tan, marked extensively with brown at intersections; gradate veins, base of Rs, icu crossveins dark brown, most with dark brown suffusion on surrounding membrane. Forewing 12.3–13.5 mm long, 4.2–4.6 mm wide, (ratio, L : W = 2.8–3.0 : 1); height of tallest costal cell 0.8 mm (cell number 5–6); length of first intramedian cell 0.8–0.9 mm; 10 radial cells (closed cells between R and Rs); third gradate cell 1.3–1.4 mm long, 0.4 mm wide (ratio, L : W = 2.5–2.6 : 1); fourth gradate cell often missing, when present 0.7–1.1 mm long, 0.4 mm wide (ratio, L : W = 1.6–2.4 : 1); 3–4 Banksian cells (b cells), 4 b’ cells; 3–4 inner gradates, 6 outer gradates. Hindwing venation robust, not heavy, marked as on forewing; 11.0–12.0 mm long, 3.3–3.7 mm wide (ratio, L : W = 3.1–3.3 : 1), 10 radial cells, 3 Banksian (b) cells, 4 b’ cells, 2–4 inner gradates, 5–6 outer gradates.

Male. Abdomen with small spiracles (e.g., A7: spiracle diameter ~0.03× length of sternite); A6–A9 with numerous robust, elongate setae extending from large setal bases, especially notable on pleural regions; T9+ectoproct relatively long (~0.6× length of T7), with dorsal invagination deep (~0.7× dorsal length of T9+ect), margins of invagination almost straight, base rounded; dorsal margin of T9+ect rounded distally (above anus); posterior margin of ectoproct gently curved throughout; ventral margin of T9+ect with well sclerotized, elongate, curved apodeme contiguous with sclerotization around callus cerci, posterior corner of apodeme extending posteriorly, bending mesally to form small, rounded knob; posteroventral corner of T9+ect appearing angular (lateral view); callus cerci large, ovate, with entire margin lightly sclerotized. S8+9 fused, with line of fusion not readily perceptible; dorsal margin with apodeme extending along basal ~¾ length of segment, then tapering abruptly without apodeme to tip of segment; terminus extending distally, slightly beyond tip of T9+ect, heavily sclerotized, upturned distally, concave in posterior view; terminal setae on S8+9 enlarged, without flange-like protrusions basally. Gonarcus arcuate (dorsal, ventral views), V-shaped (frontal, caudal views); bridge robust, moderately wide throughout; arms elongate, rounded distally, dorsal section with digitiform process extending posteriorly, then inward toward gonosaccus; mediuncus with narrow base, paired internal rods visible basally, extending distally, fusing at terminus; dorsal surface of mediuncus ridged, not smooth, with short, rounded (blunt) beak distally; gonosaccus bilobed, each lobe large, with large, dense patch of gonosetae; gonosetae robust, arising from enlarged setal bases; hypandrium internum not found.

##### Variation.

Among the specimens we examined, there was variation in the depth and size of the reddish brown markings on the vertex and frons.

##### Known distribution.

PERU: Region of La Libertad, Province of Lima (Districts of Chancay, San Isidro).

##### Specimens examined

(in addition to types above). Peru. *Huaral*: Chancay, river valley, 25/III/1951, Ross & Michelbacher (1F, CAS); Chancay, shrubs nr. River, 40 mi. N. Lima, 29/VII/1971 (1M, BMNH). *La Libertad*: Samme, 1500 m, 40 km. w. Trujillo, 12-17/VII/1975, C. Porter & L. Stange (1M, 1F, CAS; 1M, FSCA); Samme, 15/VII/1982, R. B. Miller & L. Stange (4F, FSCA); Samme, ca. Trujillo, 1,500 m, 12–19/VII/1975, C. Porter & L. Stange (1M, 2F, IFML); Simbal, 4/VII/1974, L. Stanner & C. Porter (?, IFML). *Lima*: nr. Castillo (1M, USNM); San Isidro, at light, 6/II/1977, C. P. Kimball (1F, USNM); Chosica, 2800 ft.,9/VI/??, Parish Coll., N. Banks (sex unknown, abdomen missing, MCZ; identified as “*confraterna*” by Banks, probably in error; our ID as *U. siderocephala* is tentative).

#### Ungla stangei

Taxon classificationAnimaliaNeuropteraChrysopidae

Tauber sp. n.

http://zoobank.org/20764E07-491E-445C-85DB-2DF49322BB53

**Figure 88.**
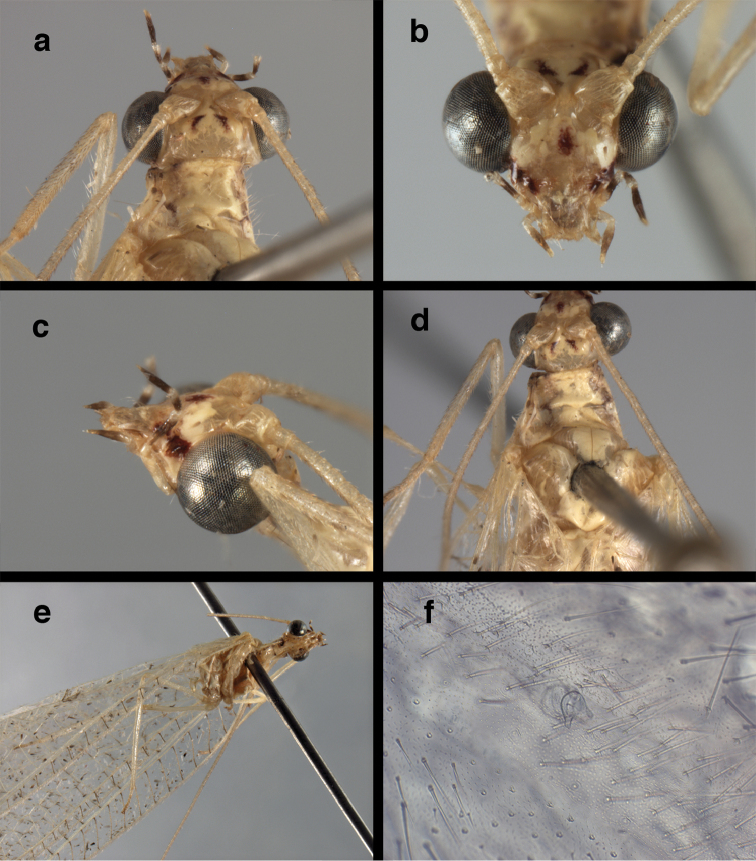
*Ungla stangei* Tauber, sp. n. External features, (**a**) head, dorsal (**b**) head, frontal (**c**) head, lateral (**d**) head, thorax, dorsal (**e**) head, thorax, ventral (**f**) male abdominal spiracle (all: Bolivia, La Paz, male; **a–e** holotype, CAS; **f** paratype, CAS).

**Figure 89.**
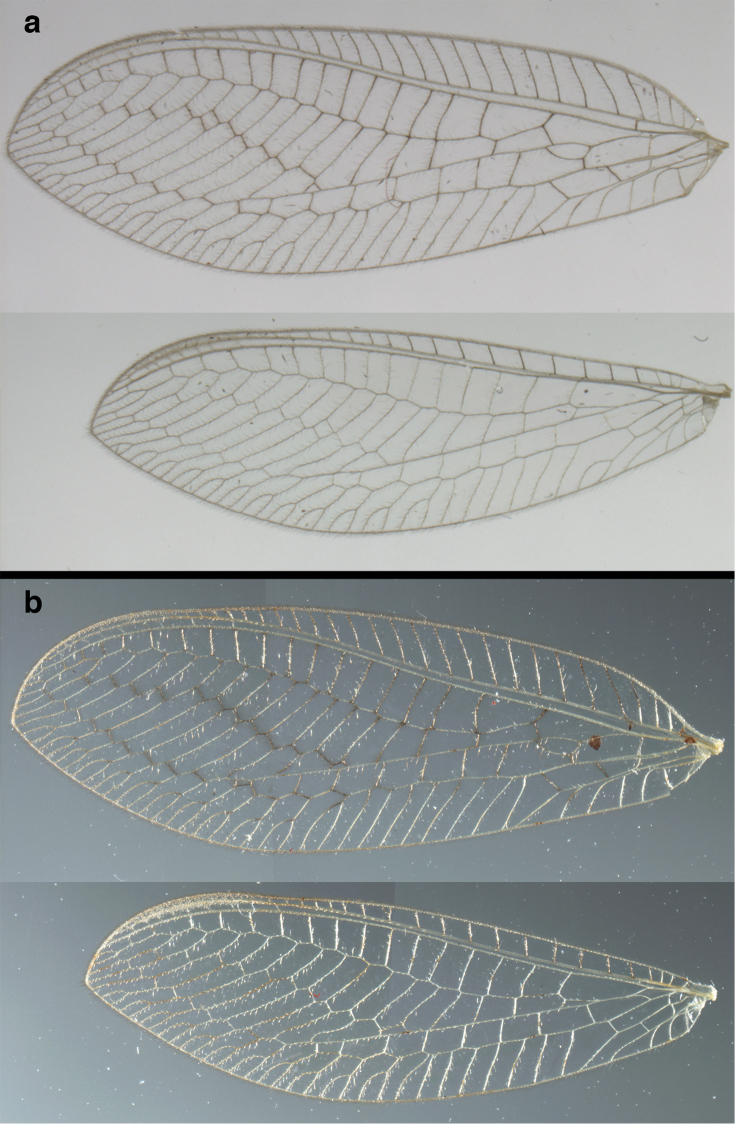
*Ungla stangei* Tauber, sp. n. Wings, (**a**) venation emphasized (**b**) coloration of veins emphasized (Bolivia, La Paz, paratypes, male, CAS).

**Figure 90.**
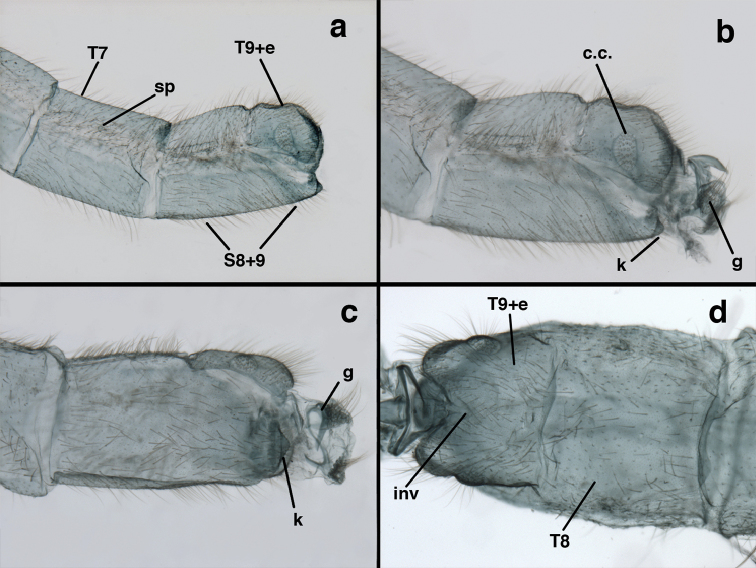
*Ungla stangei* Tauber, sp. n. Male abdomen (slightly teneral), (**a**) abdominal segments A7-terminus, lateral (**b**) terminalia, lateral (**c**) S8+9, ventral (**d**) T8, T9+ectoproct, dorsal [Note invagination.]. **c.c.** callus cerci **g** gonarcal complex **inv** dorsal invagination of T9+ectoproct **k** sclerotized knob at tip of S9 **sp** spiracle **S8+9** fused eighth and ninth sternites **T7, T8** seventh, eighth tergites **T9+e** fused ninth tergite and ectoproct (all: Bolivia, La Paz; **a–c** paratype, CAS; **d** holotype, CAS).

**Figure 91.**
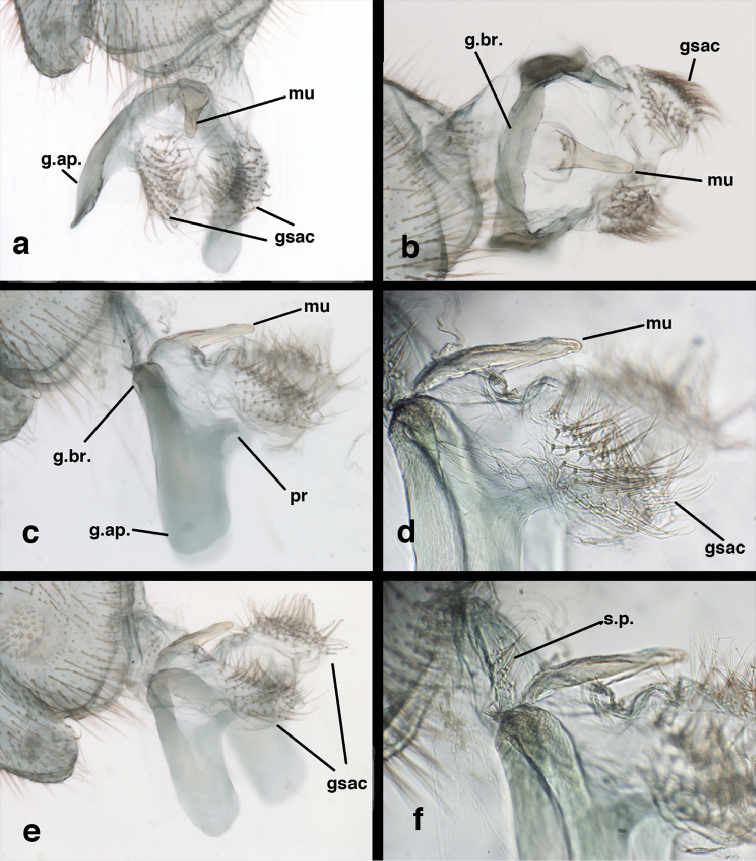
*Ungla stangei* Tauber, sp. n. Male genitalia (mature), (**a**) gonarcus, dorsal (**b**) gonarcus, frontal (**c–f**) gonarcus, lateral. **gsac** gonosaccus **g.ap.** gonarcal apodeme **g.br.** gonarcal bridge **mu** mediuncus **pr** unarticulated process on frontal margin of gonarcal apodeme **s.p.** setose subanal plate (all: Peru, Andahuaylas, paratype, CAS).

**Figure 92.**
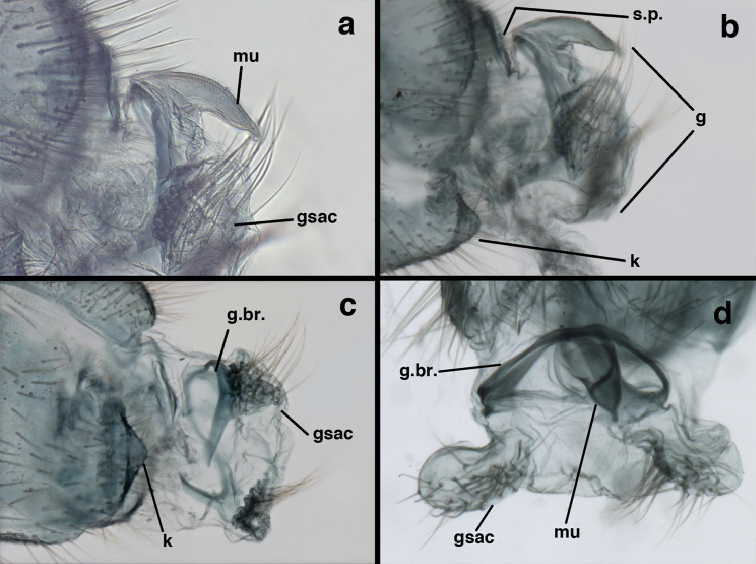
*Ungla stangei* Tauber, sp. n. Male genitalia (teneral), (**a**) gonarcus, posterolateral (**b**) gonarcus and tip of S9, lateral (**c**) S8+9 ventral [Note sclerotized knob at tip of S9.] (**d**) gonarcal complex, dorsal **g** gonarcal complex **gsac** gonosaccus **g.br.** gonarcal bridge **k** sclerotized knob at tip of S9 **mu** mediuncus **s.p.** setose subanal plate (all: Bolivia, La Paz; **a–c** paratype, CAS; **d** holotype, CAS).

Figs 88 , 89 , 90 , 91 , 92 , 144f

##### Holotype

(Figs 88a–e, 90d, 92d, 144f). CAS, male. Bolivia, La Paz, Rio Zongo, 3200 m, 24–30/XI/1984, L. E. Pena.

##### Etymology.

The species is named for Lionel A. Stange, a life-long student of the systematics and natural history of myrmeleontoid Neuroptera, an enthusiastic, generous collector of Neuropteran specimens, colleague, and friend.

##### Diagnosis.

Like *U. laufferi* (above), adults of this species are recognized by their light green body color, yellow longitudinal stripe dorsally, and distinctive pair of red spots on the vertex in place of the inverted U-shaped mark that typifies most *Ungla* species. Despite the similarities of the two species, *U. stangei* is distinguished by several notable external features: (1) larger wings (forewing length = 17.1–17.6 mm, versus 13.5–15.9 mm in *U. laufferi*), (2) wings with a greater number of gradate cells, all of which are long and narrow (n = 9–10 gradate cells; third gradate cell 2.23–2.74 mm long; fourth gradate cell 2.39–2.74 mm long), versus a smaller number of gradate cells, all of which are shorter and wider in *U. laufferi* (n = 6–7 gradate cells; third gradate cell 1.88–2.22 mm long; fourth gradate cell 1.86–2.36 mm long), (3) heavy setation on the pleural region of the terminal abdominal segments (A7, A8+9) (*U. stangei*: dense covering of elongate setae arising from enlarged setal bases; *U. laufferi*: a sparse covering of long setae arising from small setal bases).

The three male specimens of *U. stangei* that we examined appeared somewhat teneral. In all three, the abdomen and genitalia were soft, flexible, and very lightly sclerotized; the arms of the gonarcus were elongate and flexible – extending fully around and below the gonosaccus; and the mediuncus was broad, rounded, and somewhat soft at the base. We are hesitant to ascribe taxonomic significance to these features.

##### Description.

Head yellow to whitish cream-colored with red to reddish black markings; vertex smooth, often shiny, with inverted U-shaped marking reduced to two anterolateral red spots; fossae, posterior parts of vertex, area between antennae unmarked; frons cream-colored, with dark red markings laterally between base of antenna and margin of eyes, small to large red mesal spot between base of antennae and clypeus; clypeus with dark red mark on lateral margin; tentorial pits amber-colored; gena with reddish black to black stripe. Antenna with scape, pedicel, flagellum cream-colored, unmarked; maxillary palp with basal two segments pale, three distal segments black with articulations pale; labial palp with basal segment pale, distal two segments with black.

Thorax green, with distinct longitudinal yellow stripe mesally; prothorax with transverse furrow in central region of segment, extending almost to lateral margins; setae mostly elongate, pale. Measurements: head width: 1.5–1.6 mm; ratio head width : eye width: 2.3–2.8 : 1; prothorax width: 1.0–1.1 mm, length: 0.8 mm.

Forewing, hindwing elongate, with acute tips; membrane mostly clear, hyaline, with brown, fumose areas around brown veins or brown markings on veins; stigma clear to slightly opaque; longitudinal veins mostly green; transverse veins mostly green, with brown at intersections; gradate veins, r-m crossveins, distal crossveins of most b’ cells entirely brown. Forewing 16.0–17.6 mm long, 5.0–6.0 mm wide (ratio, L : W = 2.9–3.0 : 1); height of tallest costal cell 1.3–1.5 mm (cell number 5–6); width of first intramedian cell 0.9–1.0 mm; 14–18 radial cells (closed cells between R and Rs); third gradate cell 2.2–2.7 mm long, 0.4–0.5 mm wide (ratio, L : W = 4.8–5.5 : 1); fourth gradate cell 2.4–2.7 mm long, 0.4–0.5 mm wide (ratio, L : W = 5.1–6.0 : 1); 3–4 Banksian cells (b cells), 5–6 b’ cells; 9–12 inner gradates, 9–11 outer gradates. Hindwing 15.1–15.7 mm long, 4.8–5.0 mm wide (ratio, L : W = 3.1 : 1), 8–16 radial cells, 3–4 Banksian (b) cells, 5–6 b’ cells, 10–11 inner gradates, 8–10 outer gradates.

Male. Abdomen with small spiracles (e.g., A7: spiracle diameter ~0.03× length of sternite); T9+ectoproct relatively long (~half length of T7), with dorsal invagination moderately deep (~0.4x dorsal length of T9+ect), margins of invagination almost straight, base rounded; dorsal margin of T9+ect rounded distally (above anus), often compressed, thus appearing straight; posterior margin of ectoproct relatively straight, posteroventral corner slightly extended distally, with small knob; ventral margin lightly sclerotized except anteriorly; callus cerci large, ovate, margin sclerotized on sides and bottom, not top; sclerotization contiguous with that on ventral margin of ectoproct. S8+9 fused, with line of fusion perceptible; dorsum tapering abruptly to shallow platform at ¾ distance to tip of segment, dorsal margin fairly regular; terminus concave to flat, extended distally, but not beyond T9+ect, flat, distal margin upturned, heavily sclerotized, with distal projection ventrally; setae simple (without flanges), slender, mostly long. Gonarcus (teneral) circular, with bridge slender, arms elongate, curving in complete circle below bridge, mesal section probably with process extending posteriorly; mediuncus rounded, hollow basally, extending distally or downward to blunt, curved hook; bilobed gonosaccus, each lobe with single patch of large gonosetae probably facing mesally; gonosetae arising from enlarged setal bases; hypandrium internum not found.

##### Known distribution.

BOLIVIA: State of La Paz. ECUADOR: Province of Pichincha. PERU: Province of Andahuaylas. All localities at ~3000 meters elevation.

##### Specimens examined

(in addition to holotype). 4M, 1F, one without abdomen, all with same data as holotype (all paratypes, CAS). Ecuador. *Pichincha*: near Quito, 20-VII-1989, L. Stange & R. Miller (1F, paratype, FSCA). Peru. *Andahuaylas*: 5 mi. N. of Andahuaylas, 7-III-1951, Ross & Michelbacher (1M, paratype, CAS).

##### Variation.

The specimens from Bolivia and Ecuador vary somewhat in the depth of coloration in their markings and in the size of their wings, but all exhibit all of the markings mentioned in the description. In comparison, the marks on the sole specimen from Peru are light, and its wings are slightly shorter; most notably, it appears to lack a marking on the frons. We suspect that the natural coloration of this specimen has faded; but, we cannot exclude the possibility of individual variation or a pale colored population. This specimen is the only male that is not teneral.

#### Ungla yutajensis

Taxon classificationAnimaliaNeuropteraChrysopidae

Sosa, 2015

Figs 93 , 94 , 143g

**Figure 93.**
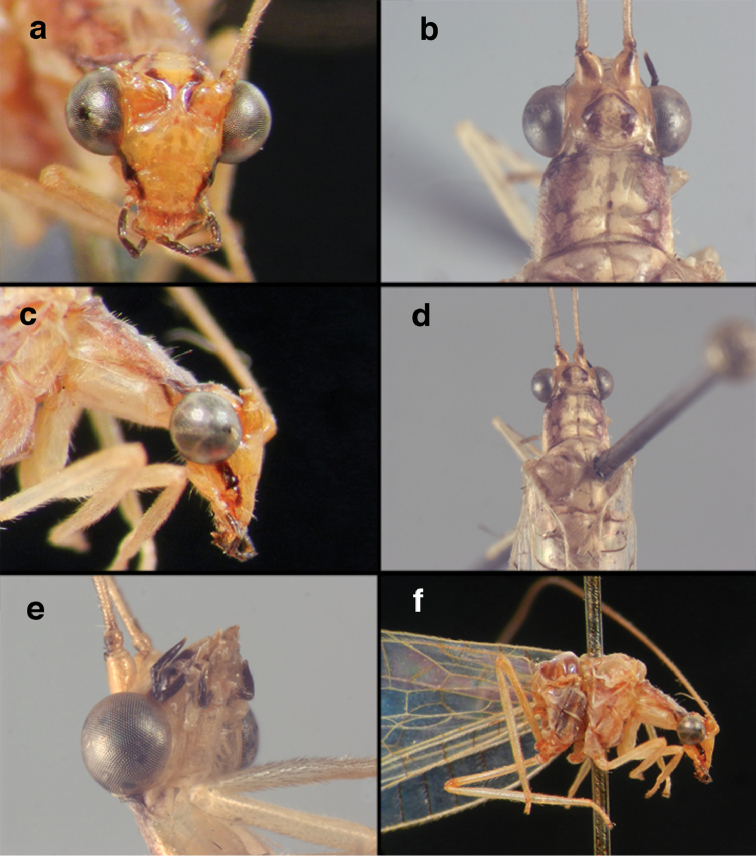
*Ungla yutajensis* Sosa: External features, (**a**) head, frontal (**b**) head, prothorax, dorsal (**c**) head, prothorax, lateral (**d**) head, prothorax, dorsal (**e**) head, ventral (**f**) head, thorax, lateral (all: Venezuela, Amazonas, female: **a, c, f** holotype, MIZA, images FS; **b, d, e** USNM).

**Figure 94.**
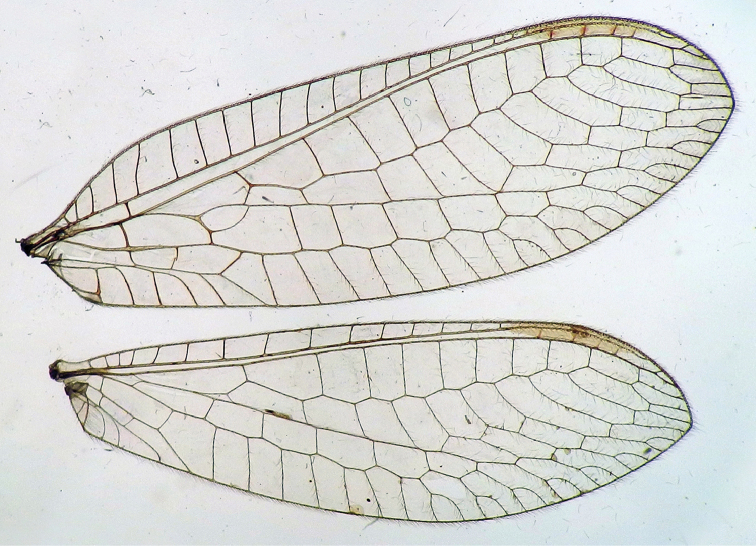
*Ungla yutajensis* Sosa: Wings (Venezuela, Amazonas, holotype, female, MIZA, images FS).

Ungla yutajensis Sosa, 2015. *Zootaxa* 4018 (2): 196-198; “VENEZUELA. *Amazonas state*: Cerro Yutajé 5°45'N / 66°08'W, 1750 m, 17-24.ii.1995, J. L. Garcia Leg. Deposited in the MIZA.” **Holotype** (Figs 93a, c, f, 94, 143g). MIZA, female. For additional images, see Sosa (2015).

##### Diagnosis.

Previously, this species was known from the single (slightly teneral) female holotype (Sosa 2015). Subsequently, additional female specimens became available for comparison; unfortunately, the male remains unknown. This species resembles both *U. favrei* and *U. diazi* in that adults have a golden head, black stripe on the gena and basolateral section of the clypeus, a pair of broad, curved, brown marks on the vertex, and broad dark brown or reddish brown lateral stripes on the prothorax. However, a pale flagellum distinguishes *U. yutajensis* from *U. diazi*, and the dark crossveins and the four dark marks in the pterostigma differentiate it from *U. favrei*.

##### Redescription.

Head yellow dorsally; vertex smooth, shiny; inverted U-shaped marking prominent, dark red, broad posteriorly, separated or lightly connected anteromesally, not extending anteriorly to area between scapes; dorsal antennal fossa pale, with dark reddish stripe mesally, reaching mesal base of scape; area between eyes and posterior half of vertex cream-colored, unmarked; frons, clypeus yellow, without markings; gena with large, black mark from eyes onto clypeus; tentorial pits amber. Antenna with scape cream-colored, with broad, diffuse dark red stripe extending full length, onto dorsal surface of pedicel; pedicel with frontal surface pale, dorsal surface dark red; flagellum yellow, except basal flagellomere brownish; maxillary palp with basal two segments pale, distal three segments black; labial palp with basal segment pale, distal two segments black.

Prothorax green, with pair of broad, dark red stripes along entire lateral length, with pair of pale, circular spots at level of transverse furrow; transverse furrow in posterior region of prothorax extending laterally only to pale spots. Mesothorax, metathorax green, with ivory mesally; mesonotum with pair of reddish spots on anterior margin of scutellum; metanotum unmarked. Legs pale, unmarked. Measurements: head width: 1.4–1.6 mm; ratio head width : eye width: 2.3 : 1; prothorax width: 1.1–1.4 mm, length: 0.7–0.8 mm.

Forewing, hindwing elongate; forewing with round apex; hindwing with slightly acute apex. Forewing with venation not inflated, except at base of Cu; stigma slightly opaque, with four to five crossveins below, basal three to four crossveins dark brown, surrounding area marked with dark brown; basal subcostal crossvein, costal, cubital crossveins black; longitudinal veins mostly green; base of Rs dark brown; transverse veins, crossveins mostly brown or reddish brown, without suffusion on surrounding membrane; gradate veins dark brown to black, with some suffusion on surrounding membrane. Forewing 15.8–15.9 mm long, 5.0–5.2 mm wide (ratio, L : W = 3.0–3.1 : 1); height of tallest costal cell 1.0–1.1 mm (cell number 5–6); length of first intramedian cell 1.0 mm; 11–12 radial cells (closed cells between R and Rs); third gradate cell 1.3–1.6 mm long, 0.5 mm wide (ratio, L : W = 2.6–3.1 : 1); fourth gradate cell 1.3 mm long, 0.5 mm wide (ratio, L : W = 2.7 : 1); 4 Banksian cells (b cells), 4 b’ cells; 5–7 inner gradates, 7–8 outer gradates. Hindwing with venation green; 14.2–14.5 mm long, 4.3–4.5 mm wide (ratio, L : W = 3.0–3.2 : 1), 11 radial cells, 3 Banksian (b) cells, 4 b’ cells, 4–6 inner gradates, 6–7 outer gradates.

Male. Unknown.

Female. See Sosa (2015: 196).

##### Variation.

No variation of note.

##### Known distribution.

VENEZUELA: State of Amazonas.

##### Specimens examined

(in addition to holotype). Venezuela, *Amazonas*: Cerro de la Neblina, Camp III. 0°56'10"N 66°3'53"W, 1820 m, 15-17 Feb, 1984, D. Davis (F, USNM); Cerro de la Neblina, Camp II. 0°49'N 65°59'W, 2100 m, 16-18 March, 1984, J. A. Louton (3F, USNM); Cerro de la Neblina, Camp XI. 0°52'N 65°58'W, 1450 m, 25–28 Feb, 1985, black light, P. J. Spangler, P. M. Spangler, R. A. Faitoute (7F, USNM).

### Part 2. *Ungla* species from southern South America (Argentina, southern Brazil)

The *Ungla* species of southern South America (Argentina, Brazil) comprise a grouping of seven valid species, none of which is known to also occur outside of the region. Six of the species (*U. annulata, U. argentina, U. chacranella, U. confraterna, U. elbergi, U. ivancruzi*) are small bodied (forewing length < 13.5 mm), and they resemble each other in a number of features. We suspect that at least some of them may be closely related to each other. The other species, *U. steinbachi*, is larger bodied (forewing length > 14.5 mm); it is known only from the type (a female). Possible relationships of *U. steinbachi* with other species are not apparent.

**Key to adults**

**Table:** 

1	Longitudinal veins of forewing with conspicuously alternating sections of white and dark brown or black; mark on vertex V-shaped (not rounded) anteriorly, extending forward toward scapes	**2**
1'	Longitudinal veins of forewing mostly light green to tan, sometimes with brown at junctions with transverse veins, or brown for long stretches; mark on vertex in shape of inverted U (rounded anteriorly), not extending forward toward scape	**3**
2 (1)	Mark on vertex extending anteriorly between scapes; frons usually with pair of light brown spots mesally (Fig. 103a–c); forewings with relatively dull membrane, with prominent fumose markings surrounding gradate veins and other crossveins (Fig. 104); veins extending from radial sector to apex of wing usually marked with black and white; male abdominal spiracles not enlarged (Fig. 105a, b)	***U. argentina* (Navás)**
2'	Mark on vertex extending towards scapes, but usually not between them; frons without mesal mark (Fig. 98b, c, d); forewing membrane shiny, hyaline, with fumose markings surrounding gradate veins and other crossveins often masked by reflection (Fig. 95b, e, f); veins extending from radial sector to apex of wing usually entirely black (Fig. 99c); male abdominal spiracles enlarged (Fig. 100a)	***U. annulata* Navás**
3 (1)	Palpomeres marked with black or dark brown laterally; forewing with most transverse veins green, sometimes brown; male abdominal spiracles enlarged or not	**4**
3'	Palpomeres cream-colored, without marks (Fig. 134b); forewing with most transverse veins and crossveins brown or black (Fig. 134d); male abdominal spiracles not enlarged (Fig. 135a)	***U. ivancruzi* Freitas**
4 (3)	Forewing with longitudinal veins regularly marked with brown at intersections, costal crossveins and sections of other crossveins marked with brown; male abdominal spiracles slightly to moderately enlarged	**5**
4'	Forewing with longitudinal veins (except sometimes the anal veins) and all crossveins entirely green or with very slight darkening at intersections with transverse veins; male abdominal spiracles may be greatly enlarged	**6**
5 (4)	Head with prominent, black stripe on gena and clypeus, from base of eyes at least part way along lateral clypeal margin (Fig. 130d, f); male abdominal spiracles moderately enlarged (width = ~0.13x length of segment) (Fig. 132a–c)	***U. elbergi* Tauber, sp. n.**
5'	Head with small genal mark, not in contact with base of eye or margin of clypeus (Fig. 126b); male abdominal spiracles slightly enlarged (width = ~0.10x length of segment) (Fig. 128a)	***U. confraterna* (Banks)**
6 (4’)	Forewing small (no more than 13.5 mm long, 4.8 mm wide); 10-12 radial cells; 5-6 inner gradates, 5-6 outer gradates (Figs 107i, 117); male abdominal spiracles greatly enlarged (Fig. 118a–d)	***U. chacranella* (Banks)**
6'	Forewing large (over 14.5 mm long, 5.3 mm wide); 13 radial cells; 7 inner gradates, 9 outer gradates; male unknown (Fig. 138a)	***U. steinbachi* (Navás)**

#### Ungla annulata

Taxon classificationAnimaliaNeuropteraChrysopidae

Navás, 1914

**Figure 95.**
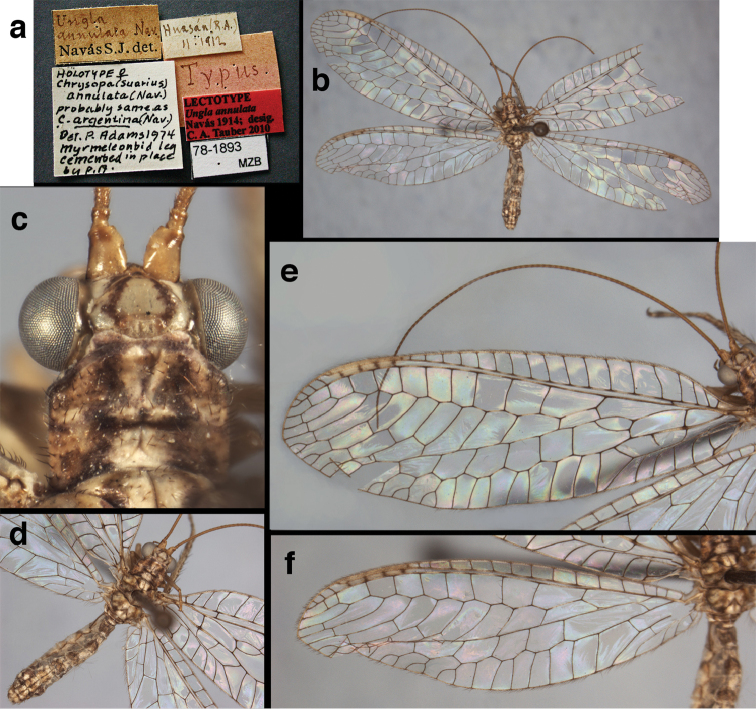
*Ungla annulata* Navás: External features, (**a**) labels (**b**) habitus, dorsal (**c**) head, dorsal (**d**) body, dorsal (**e**) forewing (**f**) hindwing (Argentina, Catamarca, lectotype, female, MZBS).

**Figure 96.**
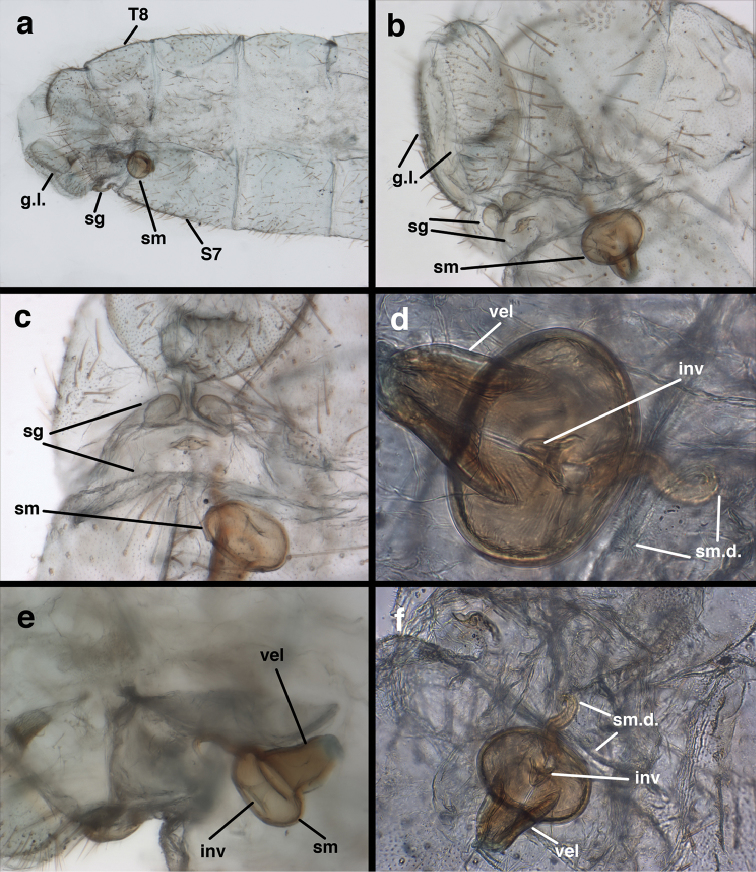
*Ungla annulata* Navás: Female abdomen and genitalia, (**a**) segments A6-terminus, lateral (**b**) terminus, ventrolateral (**c**) genital structures, ventral (**d**) spermatheca (**e, f**) spermathecal complex, *in situ*. **g.l.** gonapophysis lateralis **inv** spermathecal invagination **sg** subgenitale **sm** spermatheca **sm.d.** spermathecal duct **S7** seventh abdominal sternite **T8** eighth abdominal tergite **vel** velum (Argentina, Catamarca, lectotype, MZBS).

**Figure 97.**
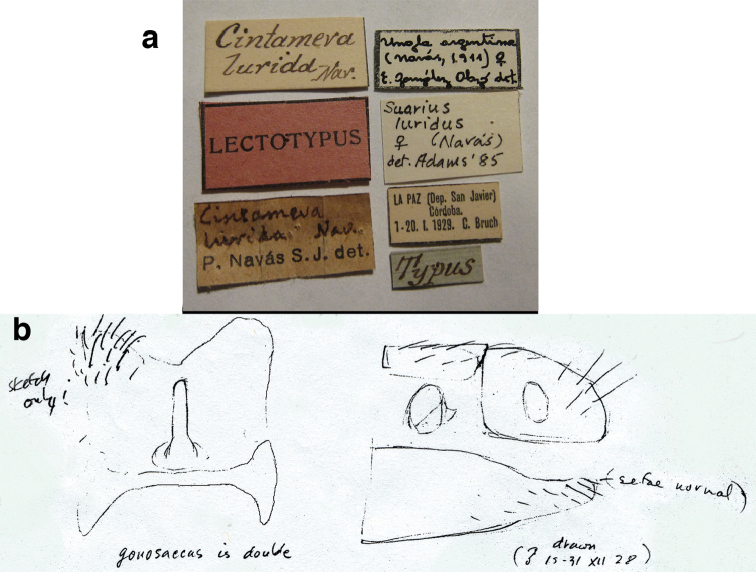
*Cintameva lurida* Navás: Syntypes, (**a**) labels on female syntype (**b**) sketches of male syntype from P. A. Adams notes, prob. 1985 (all: Argentina; Córdoba, MACN).

**Figure 98.**
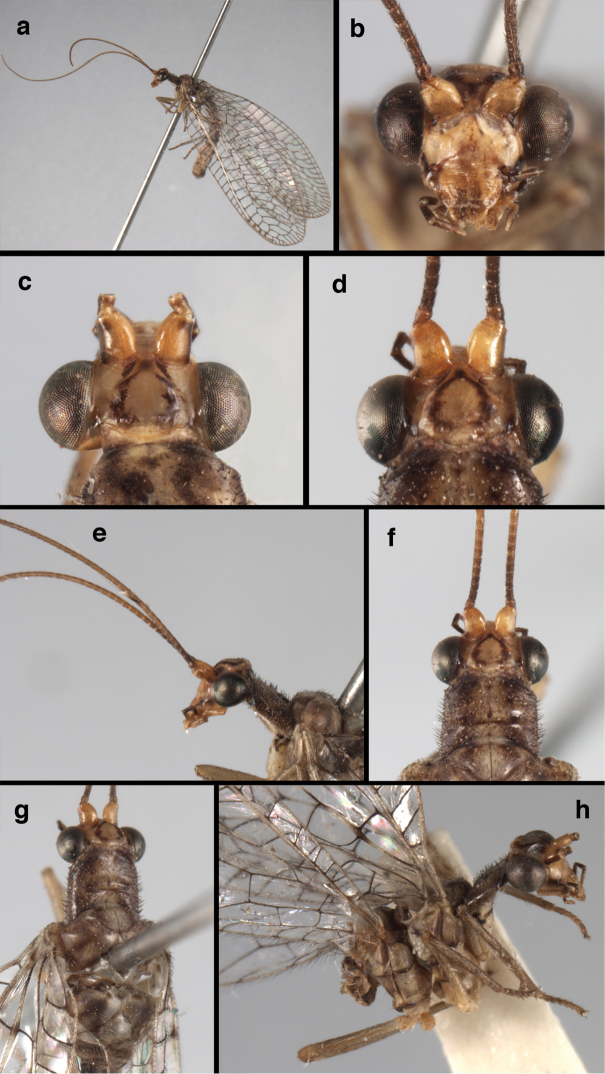
*Ungla annulata* Navás: External features, (**a**) habitus, lateral (**b**) head, frontal (**c, d**) head, dorsal [Note variation in markings on scape and vertex.] (**e**) head, prothorax, lateral (**f**) head, prothorax, dorsal (**g**) head, thorax, dorsal (**h**) head, thorax, lateral (all: Argentina, Salta, female; **a, d–g** USNM; **b, c, h** SEMC).

**Figure 99.**
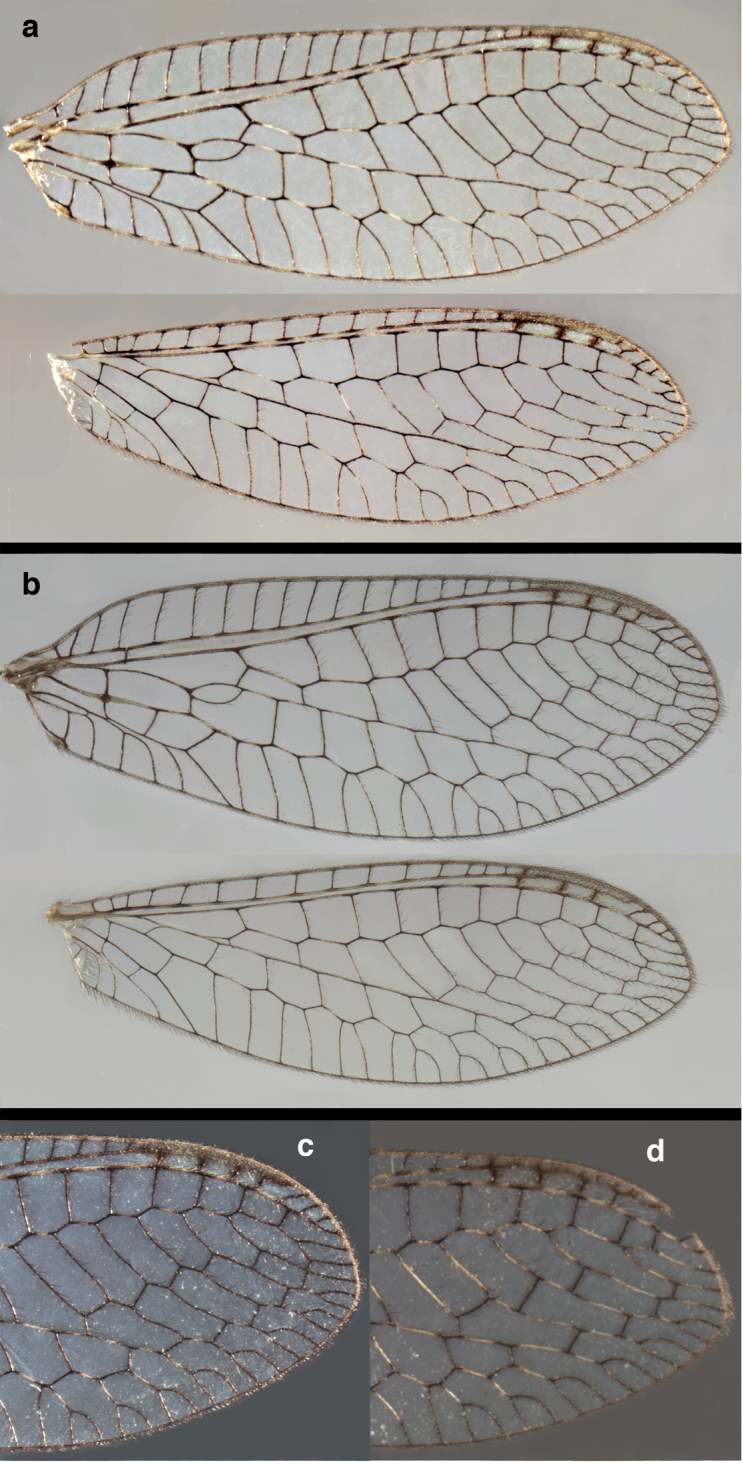
*Ungla annulata* Navás: Intra and interspecific variation in wings, (**a, b**) forewings (upper); hindwings (lower) (**c**) tip of forewing (**d**) for comparison, tip of forewing of *U. argentina* (Navás) (Argentina; **a, b, c** Salta, female, USNM; **d** Santiago del Estero, female, CAS).

**Figure 100.**
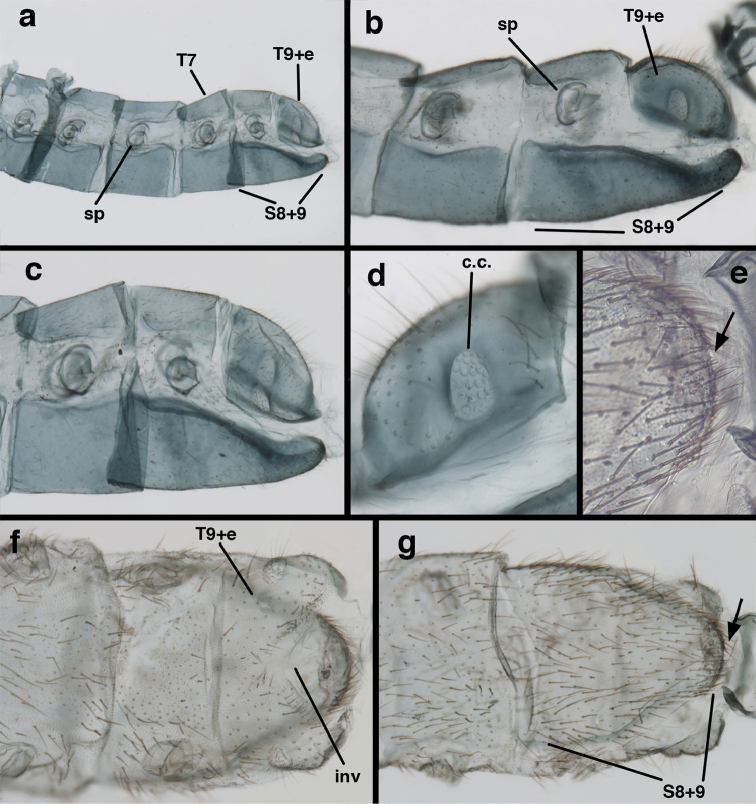
*Ungla annulata* Navás: Male abdomen, (**a**) segments A4-terminus, lateral (**b, c**) segments A7-terminus, lateral (**d**) callus cerci (**e**) tip of S9 with large, unmodified terminal setae (**f**) terminal tergites, dorsal [Note deep mesal invagination of T9+e.] (**g**) fused terminal sternites 8+9, ventral. **c.c.** callus cerci **inv** invaginated dorsal cleft in T9+ectoproct **sp** spiracle **S8+9** fused eighth and ninth sternites **T7** seventh tergite **T9+e** ninth tergite + ectoproct. Arrows indicate tip of S9 (Argentina; **a, c–g**: Salta, SEMC; **b**: Buenos Aires, USNM).

**Figure 101.**
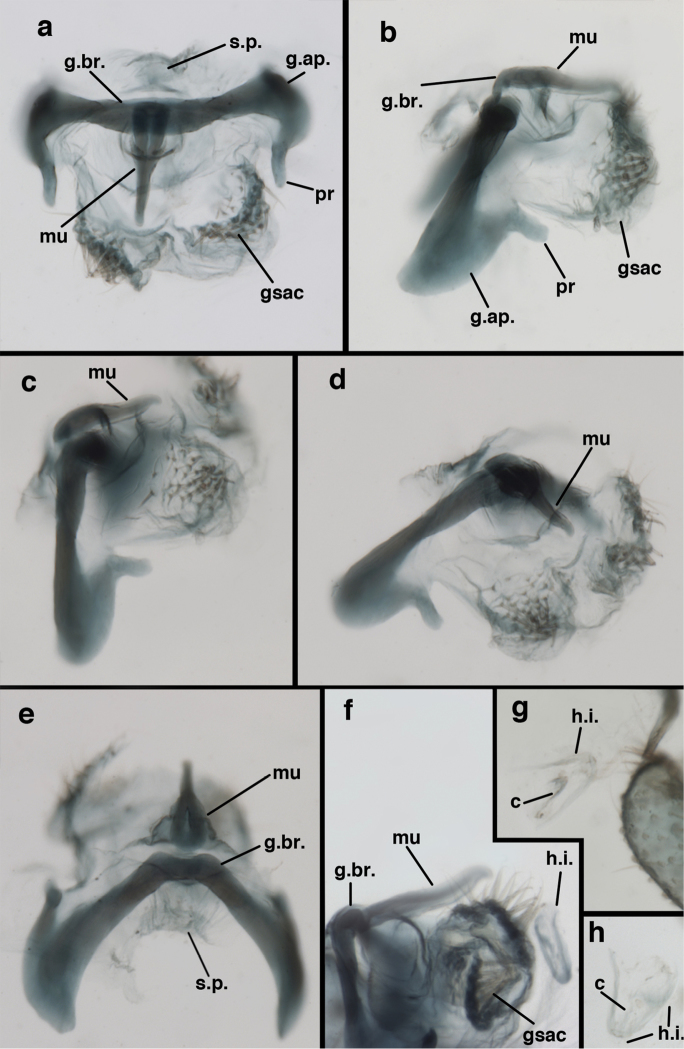
*Ungla annulata* Navás: Male genitalia, (**a**) gonarcus, dorsal (**b**) gonarcus, lateral (**c**) gonarcus, posterolateral (**d**) gonarcus, frontolateral (**e**) gonarcus, posterior (**f**) gonarcal complex, partial, lateral (**g, h**) hypandrium internum. **c** comes **g.ap.** lateral apodeme of gonarcus **g.br.** gonarcal bridge **gsac** gonosaccus **h.i.** hypandrium internum **mu** mediuncus **pr** unarticulated process on frontal margin of gonarcal apodeme **s.p.** setose subanal plate (Argentina, Salta; **a–e** SEMC; **f** FSCA; **g** CAS).

Figs 95 , 96 , 97 , 98 , 99 , 100 , 101

Ungla annulata Navás, 1914. *Broteria (Zool.)* 12: 224-225; “República Argentina: Huasán, Febrero de 1912 (Bruch.)”. Stange 1967: 41 (catalog); Adams 1975: 169 (synonym of Hypochrysa argentina Navás); Penny 1977: 16 (list, as synonym of H. argentina); Brooks and Barnard 1990: 276 (tax, as synonym of U. argentina); Monserrat 1985: 240 (type); Monserrat and Freitas 2005: 165 (tax, as synonym of U. argentina); Oswald 2015 (catalog, as synonym of U. argentina). **Lectotype** (Figs 95, 96): MZBS, female (examined). Monserrat (1985: 240) reported a type from the MZBS; it is a female, studied by Adams (1975: 169). We (CAT) examined the specimen in 2010; to help stabilize the nomenclature of Ungla, here this specimen is designated as the lectotype (des. CAT). *Type locality*: The type locality “Huasán” is located in northwestern Argentina – Andalgalá, Catamarca (27°34'S, 66°19'W, ~1100 m).
Cintameva lurida Navás, 1930. *Rev. Chil. Hist. Nat.* 34: 65-66; “República Argentina: La Paz (Dep. San Javier), Córdoba 1-20 de Enero de 1929. Col Bruch”. Stange 1967: 35 (catalog); Penny 1977: 19 (list, as Chrysopa); Brooks and Barnard 1990: 280 (list, as “ ‘Chrysopa’ incertae sedis”); González Olazo 1996: 378 (catalog, as synonym of U. argentina); Oswald 2015 (catalog, as synonym of U. argentina). **syn. n. Syntypes** (Fig. 97). MACN. Several specimens from the type locality and the approximate collection dates (Dec. 15-31, 1928, Jan 20, 1929) are in the MACN. Any of those specimens that carry dates from Jan 1 to Jan 20 could be considered syntypes. Currently, one of the specimens carries lectotype and type labels, but none of these labels are in Navás’ hand, and it is not clear which of the specimens in the MACN Navás may have used. Stange (1967: 35) noted a male and González Olazo (1996: 378) noted a female that they each considered as the holotype. Given that Navás clearly had more than one specimen, we suggest that a lectotype be designated after the full series of syntypes has been studied carefully. **Support for synonymy.**González Olazo (1996: 378) recognized that this species belongs in the genus Ungla, and he listed it as synonymous with U. argentina. However, it is not clear what specimens or information he considered to support his synonymy. Here, in the absence of a confirmed type specimen for C. lurida, we used Navás’ description of the external features and Adams’ unpublished notes and drawings from specimens in the MACN. These specimens had been collected at the type locality during the period mentioned in the original description and during the month earlier. Adams apparently considered these specimens as representative of the C. lurida type series.

##### Diagnosis.

This species, along with *U. argentina*, can be differentiated from most other *Ungla* species by a number of features: brownish coloration, longitudinal wing veins with alternating white or cream-colored and dark brown or black, often with dark patches at intersections. It differs from *U. argentina* in usually expressing the following external features: vertex with distinct, inverted U-shaped marking, usually divided mesally, with anterior markings running forward, but not extending beyond scapes to the frons; frons pale (sometimes whitish), unmarked; wing membrane irridescent, wings with longitudinal and some transverse veins alternately white and black, distal ones, dark (see Figs 98, 99c, d, for comparison of distal venation with *U. argentina*). In the males of both species, the terminus of S8+9 has large, but simple (unflanged) setae, and the gonosaccus is bilobed, with lobes broadly connected. In *U. annulata*, the abdominal spiracles are greatly enlarged – a trait that clearly separates it from *U. argentina*. Also, the ventral apodeme of the *U. annulata* ectoproct extends distally beyond the terminus of the segment, a feature that does not occur in *U. argentina*.

##### Redescription.

Head: vertex cream-colored to tan, with dark reddish brown “U-shaped marking” prominent, usually broken mesally, with pigmented coloration extending forward from tips of marking to area between scapes, not extending to frons; anteromesal margin of antennal fossa also with elongated, reddish brown to brown marking extending to mesal margin of scapes; gena, lateral margins of the clypeus with dark brown stripe. Antenna with scape cream-colored to tan with diffuse, brown, longitudinal mark or marks laterally, pedicel mostly brown or light brown, flagellum tinged with brown basally, tan distally; basal palpomeres pale, mesal palpomeres brown, terminal segments pale with brown basally.

Prothorax brown to dark brown laterally, tan to light brown mesally, with transverse furrow in posterior region, not reaching lateral margins, with short, dark setae throughout. Mesothorax, metathorax brown to dark brown, with darker brown markings. Measurements: head width: 1.3–1.4 mm; ratio head width : eye width: 2.3–2.5 : 1; prothorax width: 1.0–1.2 mm; prothorax length: 0.7 mm.

Forewing, hindwing broad to very moderately narrow, with apices rounded (forewing) to rounded or slightly subacute (hindwing), venation slender to slightly robust; alar membrane hyaline, with suffusion light, often obscured by reflection; stigma light brown, opaque, with four to five black crossveins below surrounded by dark brown marks; longitudinal veins with alternate creamy and dark markings (usually dark brown at intersections with dark veins and cream-colored between intersections); transverse veins, gradate veins, crossveins dark. Forewing 10.2–11.6 mm long, 3.7–4.3 mm wide (ratio, L : W = 2.7–3.0 : 1); height of tallest costal cell 0.7–0.9 mm (cell number 3–6); first intramedian cell ovate, 0.8–0.9 mm long; 9–10 radial cells (closed cells between R and Rs); third gradate cell 1.0–1.2 mm long, 0.4–0.5 mm wide (ratio, L : W = 2.6–3.2 : 1); fourth gradate cell 0.9–1.1 mm long, 0.4–0.5 mm wide (ratio, L : W = 2.2–3.2 : 1); 3–4 Banksian cells (b cells), 4 b’ cells; 3–5 inner gradates, 5–6 outer gradates. Hindwing 9.1–10.4 mm long, 3.0–3.4 mm wide (ratio, L : W = 3.0–3.1 : 1), 9–10 radial cells, 3 b (Banksian) cells, 4 b’ cells, 3–5 inner gradates, 4–6 outer gradates.

Male. Abdomen with greatly enlarged spiracles, with large flaps on opening (e.g., A7: spiracle diameter ~0.30× length of sternite); T9+ectoproct short, rounded posterodorsally, with dorsal invagination extending ~3/4 distance to anterior margin of T9, lateral margins of invagination straight; ventral margin of T9+ect sclerotized throughout, terminus extending distomesally as distinct knob; callus cerci large, ovate, entire margin lightly sclerotized, sclerotization contiguous with sclerotization along posteroventral margin of segment; subanal plate small, delicate, with patch of one to eight irregularly placed setae. S8, S9 fused, fusion demarcated; dorsal margin with light sclerotization, heavier throughout terminal ~1/5^th^ of S8+9, terminal setae robust, not flanged or otherwise modified. Gonarcus with bridge slender, lateral apodemes slightly expanded, rounded distally, process on side of lateral apodeme bent perpendicularly (lateral view) and slightly inward (frontal view) from gonarcal arm; mediuncus long, narrow, with dorsal margin slightly bowed; gonosaccus distinctly bilobed, with lobes broadly connected mesally; each lobe with large patch of gonosetae on enlarged bases – anterobasal gonosetae smaller, shorter, on smaller bases than posterodistal gonosetae; hypandrium internum U-shaped with arms straight (frontal view), bending upward (lateral view), comes hook-shaped.

##### Variation.

The *U. annulata* specimens that we examined varied in size and the depth of the dark markings on the head and body and the degree of white and black on the wing veins. The male abdominal and genital characteristics were consistent among specimens.

##### Larvae and biology.

Unknown.

##### Known distribution.

ARGENTINA (northwest to central east): Provinces of Buenos Aires, Catamarca, Cordoba, Entre Rios, Salta, Tucumán.

##### Specimens studied

(in addition to types above). Argentina. *Buenos Aires*: Sa. [Sierra] de la Ventana, R. Sauce Grande, 23–25/II/1968, O. S. Flint, Jr. (3M, 16F, USNM). *Cordoba*: Alta Gracia, 9/I/1968, J. & L. Stange (1M, 4F, FSCA); Embalse, 10/I/1977, L. Stange (1F, IFML). *Entre Rios*: Pronunciamiento, 1/X/1963, F. H. Walz (1M, CAS). *La Rioja*: no locality, E. Giacamelli (1F, MSNG). *Salta*: Cafayate, 2/XII/1970, C. Porter & L. Stange (1F, IFML), 1/I/1972. D. J. Brothers, at light (2M, 5F, SEMC); Cafayate, Yacochuya, 1500 m, 1–15/IV/1969, Willick, Terán, Stange (2M, 2F, FSCA); no locality (probably Salta city), 14/II/1951, Ross & Michelbacher (1M, 4F, CAS). *Tucumán*: San Pedro de Colalan, L. A. Stange (2M, FSCA); Amaicha, 19-XI-1966, L. Stange (1F, FSCA).

The above specimen from La Rioja (MSNG) bears a red type label with the name “*Cintameva giacamellina* Nav.” handwritten by Navás. Apparenly, the name was never published; it is not listed by Oswald (2015). We identified the specimen as a female of *U. annulata*.

#### Ungla argentina

Taxon classificationAnimaliaNeuropteraChrysopidae

(Navás, 1911)

**Figure 102.**
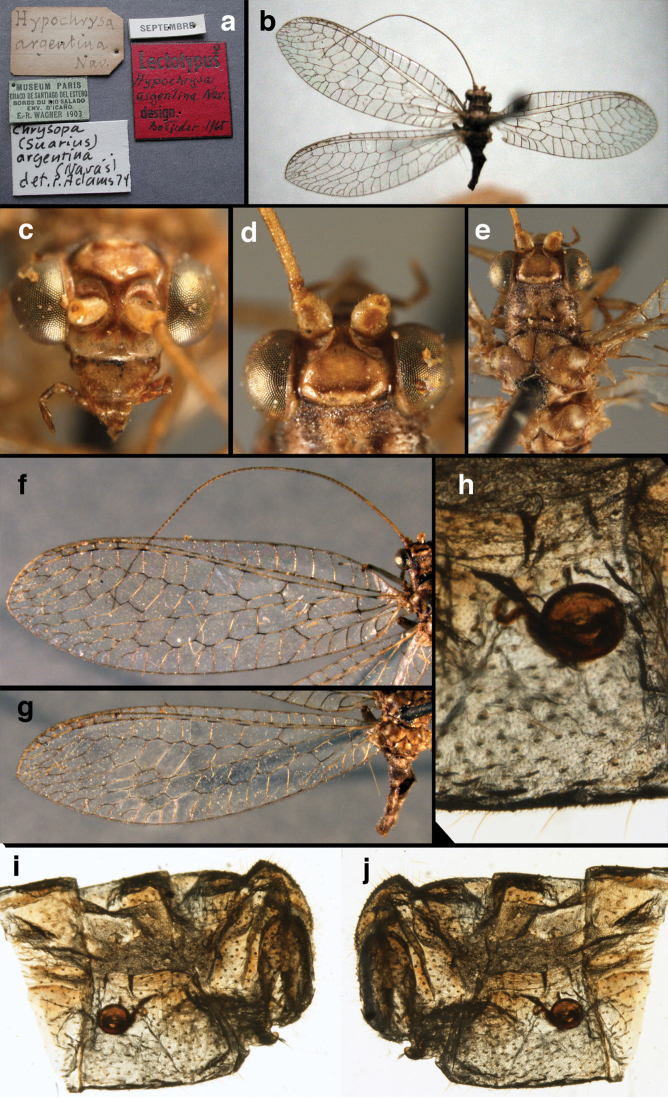
*Ungla argentina* (Navás): Features, (**a**) labels (**b**) habitus, dorsum (**c**) head, frontal (**d**) head, dorsal (**e**) head, thorax, dorsal (**f**) forewing (**g**) hindwing (**h**) spermatheca (**i, j**) terminalia, left, right (Argentina, Santiago del Estero, lectotype, female, MNHN).

**Figure 103.**
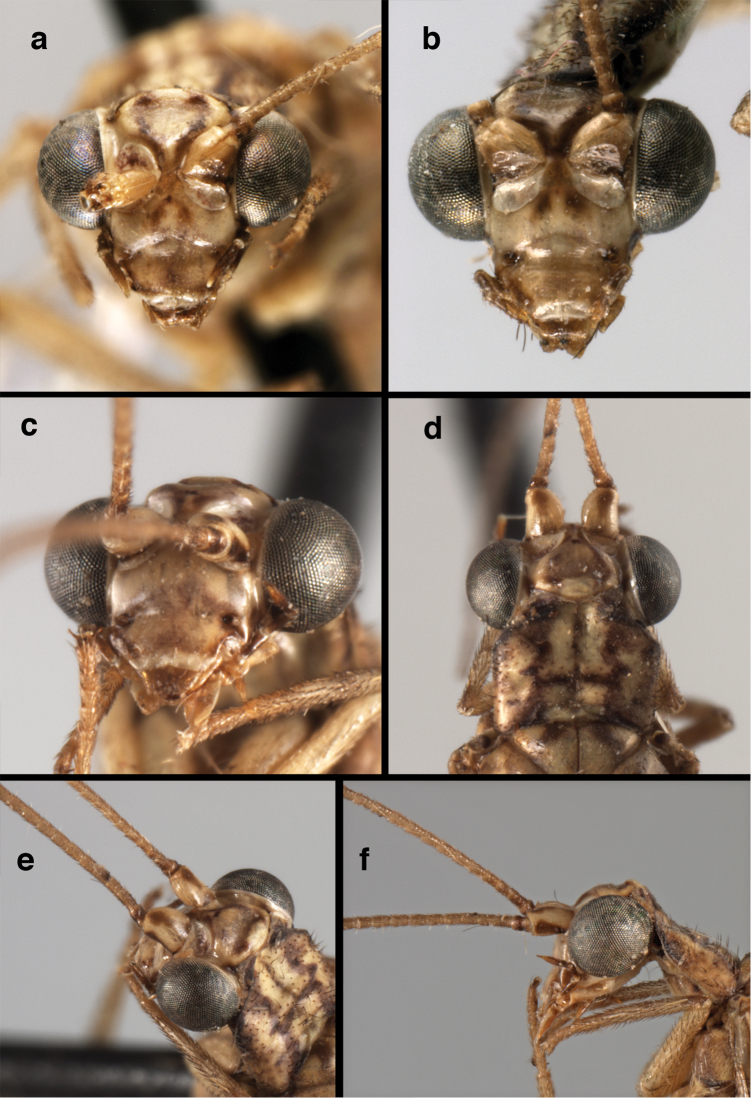
*Ungla argentina* (Navás): External features, (**a, b, c**) head, frontal [Note variation in frontal markings.] (**d**) head, prothorax, dorsal (**e**) head, prothorax, dorsolateral (**f**) head, prothorax, lateral (Argentina, Santiago del Estero, CAS; **a** male; **b–f** female).

**Figure 104.**
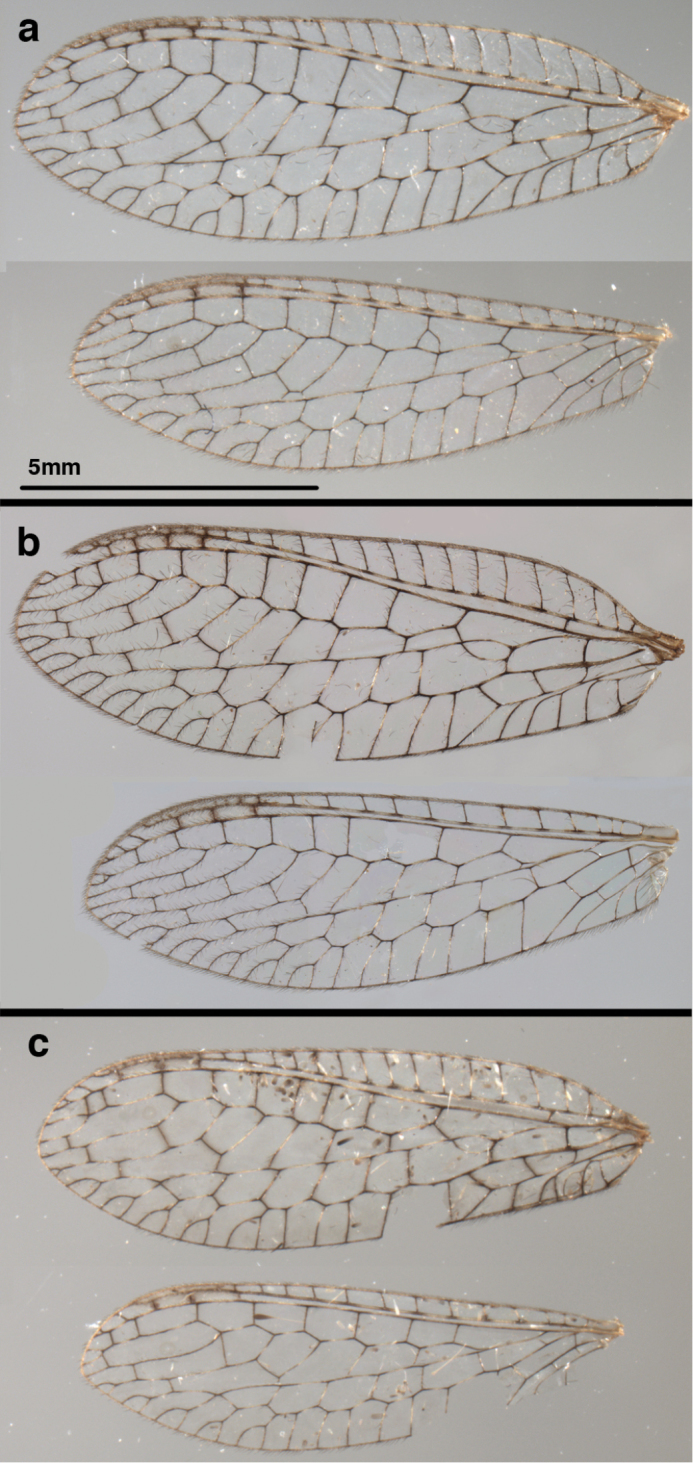
*Ungla argentina* (Navás): Variation in size, shape, coloration, venation of forewings and hindwings (Argentina; **a** Catamarca, male, CAS; **b** Santiago del Rio, female, CAS; **c** La Rioja, male, CAS). Scale applies to all images.

**Figure 105.**
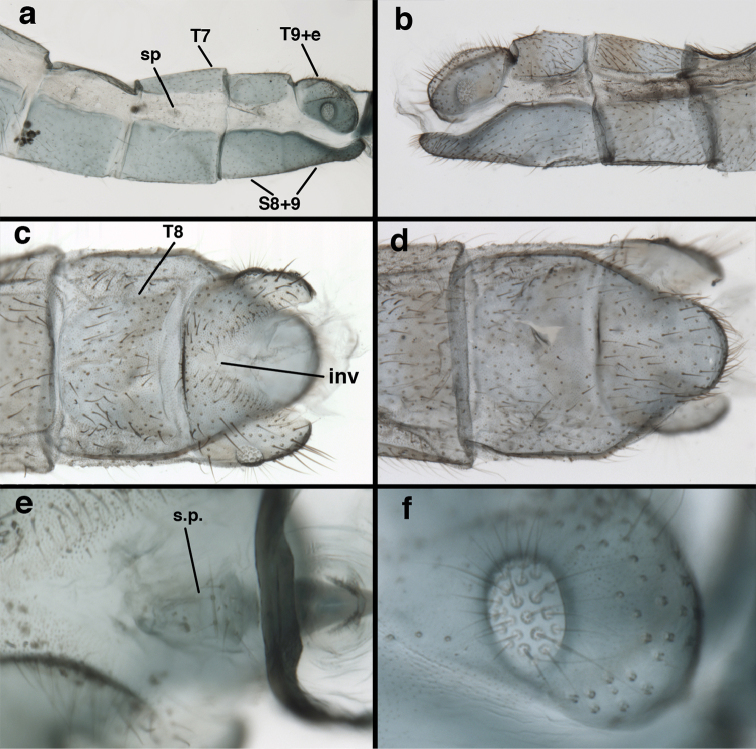
*Ungla argentina* (Navás): Male abdomen, (**a, b**) segments A6-terminus, lateral (**c**) tergites T8 and T9+ectoproct, dorsal (**d**) fused sternites S8+9, ventral (**e**) terminus, dorsal (**f**) callus cerci. **inv** invaginated dorsal cleft in T9+ectoproct **sp** spiracle **s.p.** setose subanal plate **S8+9** fused eighth and ninth sternites **T7** seventh tergite **T8** eight tergite **T9+e** fused ninth tergite and ectoproct (Argentina; **a, e, f** Tucumán, CAS; **b, c, d** Santiago del Estero, CAS).

**Figure 106.**
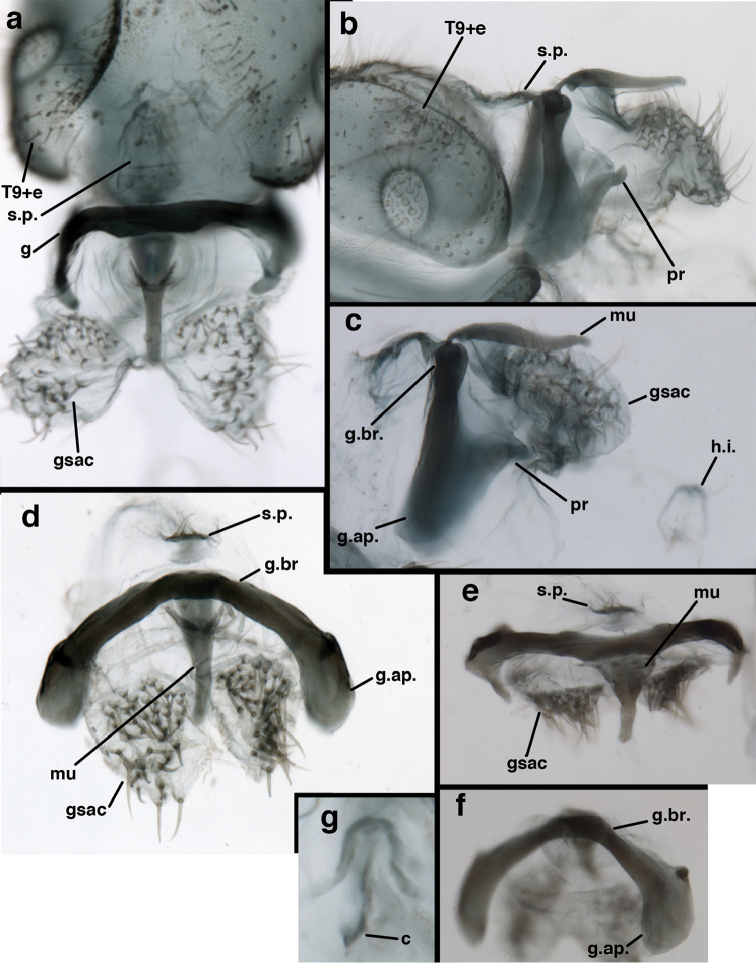
*Ungla argentina* (Navás): Male genitalia, (**a**) gonarcus, frontodorsal, attached dorsally to abdominal terminus via very short membrane (**b**) gonarcus, membranous attachment to T9+ectoproct, lateral (**c**) gonarcus, lateral, with hypandrium internum, frontal (**d**) gonarcus, frontodorsal (**e**) gonarcus, dorsal (**f**) gonarcus, posterior (**g**) hypandrium internum, frontal. **c** comes **g** gonarcus **g.ap.** gonarcal apodeme **g.br.** gonarcal bridge **gsac** gonosaccus **h.i.** hypandrium internum **mu** mediuncus **pr** unarticulated process on frontal margin of apodeme **s.p.** setose subanal plate **T9+e** fused ninth tergite and ectoproct (Argentina; **a, b, g** Tucumán, CAS; **c** Catamarca, CAS; **d, e, f** Santiago del Estero, CAS).

Figs 102 , 103 , 104 , 105 , 106

Hypochrysa argentina Navás, 1911. *Ann. Soc. Sci. Bruxelles* 35 (pt. 2): 275; “Chaco de Santiago del Estero. Bords da Rio Salado, env. d’Icaño. E.-R. Wagner, septembre, 1903”. Navás 1913: 93 (tax). Chrysopa argentina (Navás), by Tjeder 1966: 247. Adams 1967: 221 (tax); Stange 1967: 31 (catalog); Tjeder 1971: 113 (redescr, tax); Penny 1977: 16 (list). Suarius argentinus (Navás), by Adams 1975: 169 (redesc). Adams and Penny 1985: 436 (discussion); Adams and Penny 1986: 121 (tax); Oswald 2015 (catalog). Ungla argentina (Navás), by Brooks and Barnard 1990: 241, 276. González Olazo 1996: 378, 381 (tax), Monserrat and Freitas 2005: 165-168 (redesc, larval desc, biol); Freitas 2007: 415 (key to adults); Legrand et al. 2008: 115 (type); Tauber et al. 2014: supplementary material (list); Oswald 2015 (catalog). **Lectotype** (Fig. 102). MNHN, female (examined), designated by Tjeder (1971: 112–113; also see Legrand et al. 2008: 115), Oswald (2015). *Type locality*: The type was collected on the banks of Rio Salado, near Icaño, on the eastern edge of the northern Argentinian province of Santiago del Estero. Rio Salado is a tributary of Rio Paraná. The term “Chaco” in “Chaco de Santiago del Estero” has several possible meanings; here it probably refers to the lowland part of the Chaco region that was acquired by the province of Santiago del Estero. The specimen has its right antenna and posterior right wing missing; the abdomen is permanently mounted between two small discs, and the internal features are difficult to see clearly. **Synonyms.** At one time or another, seven species names were synonymyzed with U. argentina. However, at this time, none of the synonymies are confirmed. One (Ungla annulata Navás) is a valid biological entity and is treated above. Two of the names [Cintameva lurida Navás and Chrysopa plesia Navás] are junior synonyms of species other than U. argentina; they are treated under U. annulata and U. chacranella, respectively. The final four (Chrysopa venulosa Navás, C. graciana Navás, C. nervulosa Navás, and C. coronata Navás) are considered *species inquirenda*, and are discussed at the end of the article.

##### Diagnosis.

The lectotype of *Hypochrysa argentina* expresses a set of external characters that we concluded are typical for the species: wings with membrane somewhat shiny to slightly dull, veins robust; both longitudinal veins and transverse veins marked alternately with white (or cream-color) and black (or dark brown); gradate veins dark with prominent fumose marks on surrounding membrane; dorsum of head (vertex) brown, with relatively weak, inverted U-shaped marking, with paired darker brown markings extending anteriorly from the vertex, between the scapes, onto the frons and around the lower margins of the antennal fossae. The *U. argentina* male abdomen has small, unmodified spiracles; the mediuncus is relatively short and slightly sinuous dorsally; gonosetae are present in a single patch on the upper and outer surfaces of each lobe of the bilobed gonosaccus; and setae at the tip of S8+9 are simple, i.e., they are not flanged.

##### Redescription.

Head: vertex light brown to brown, with U-shaped marking prominent to somewhat obscured, brown coloration extending anteriorly between the scapes, usually to the frons, forming an X-shaped frontal marking below antennal fossae; antennal fossa with large, dark brown mark extending around dorsal margin, between antennae, and around frontal margin; gena, lateral margin of clypeus with dark brown stripe. Antenna cream-colored; scape with diffuse, brown, longitudinal mark or marks; pedicel mostly brown; flagellum tinged with brown. Basal two labial palpomeres pale, distal three brown; membrane between palpomeres cream-colored; distal maxillary palpomere light brown, basal two cream-colored to tan.

Prothorax with brown, longitudinal stripes sublaterally, laterally; transverse furrow in posterior region, not reaching lateral margins; short, dark setae throughout. Mesothorax, metathorax with lighter, more diffuse brown markings. Measurements: head width: 1.3–1.4 mm; ratio head width : eye width: 2.4–2.6 : 1; prothorax width: 1.0 mm; prothorax length: 0.5–0.7 mm.

Forewing, hindwing moderately narrow, with well-rounded apices, robust venation; alar membrane clear, with light to dark brown suffusion adjacent to dark veins; stigma usually light brown, cloudy to opaque, with three to four black crossveins below, surrounded by dark brown fumose to solid marks; longitudinal and transverse veins with alternate creamy and dark markings (usually dark brown at intersections with crossveins and cream-colored between intersections); gradate veins, crossveins dark. Forewing 8.6–11.1 mm long, 2.8–3.8 mm wide (ratio, L : W = 2.9–3.1 : 1); height of tallest costal cell 0.6–0.8 mm (cell number 5–6); first intramedian cell ovate, 0.6–0.8 mm long; 8–9 radial cells (closed cells between R and Rs); third gradate cell 0.6–1.2 mm long, 0.3–0.4 mm wide (ratio, L : W = 1.8–3.5 : 1); fourth gradate cell 0.8–0.9 mm long, 0.3 mm wide (ratio, L : W = 2.6–2.9 : 1); 3–4 Banksian cells (b cells), 4 b’ cells; 2–4 inner gradates, 3–5 outer gradates. Hindwing 7.7–9.7 mm long, 2.3–3.1 mm wide (ratio, L : W = 3.1–3.3 : 1), 8–9 radial cells, 3 b (Banksian) cells, 4 b’ cells, 2–4 inner gradates, 3–5 outer gradates.

Male. Abdomen with spiracles small (e.g., A7: spiracle diameter ~0.04x length of sternite); T9+ectoproct short, rounded posteriorly, with dorsal invagination deep, almost reaching anterior margin of T9, lateral margins of invagination straight to convex; callus cerci large, ovate, entire margin lightly sclerotized, sclerotization extending distally along posteroventral margin of segment; subanal plate small, delicate, with patch of one to eight irregularly placed setae. S8, S9 fused, fusion demarcated; dorsal margin with light sclerotization anteriorly, heavier throughout terminal ~1/5^th^ of S8+9; terminal setae slightly robust, otherwise unmodified. Gonarcus with bridge slender, lateral apodemes slightly expanded, blunt, process on sides of lateral apodemes curved smoothly, perpendicularly (lateral view) and slightly inward (frontal view) from gonarcal arm; mediuncus long, narrow, with dorsal margin slightly bowed; gonosaccus distinctly bilobed, each lobe with large patch of robust gonosetae on enlarged bases – anterobasal gonosetae smaller, shorter, on smaller bases than posterodistal gonosetae; hypandrium internum U-shaped with arms bending outward distally, comes elongate, irregular.

Female. Spermatheca round, pillbox-shaped, velum obscured, invagination present but size not clear, spermathecal duct with U-shaped bend and curve; subgenitale with short knob-like protrusion.

##### Variation.

The clarity of the alternate white and dark markings on the wing veins is quite variable; white areas are often dull. Similarly, the size, shape, and prominence of the head and facial marks can also vary; occasionally the paired brown frontal marks are absent, but the mark on the vertex usually extends anteriorly between the scapes and onto the top of the frons.

We examined one particularly small male specimen (Argentina: La Rioja, Guadacol, 1–3 December 1983, Luis F. Pena, CAS) that, based on abdominal features, we believe is *U. argentina*. However, it expresses very unusual features: extensive regions of white on the transverse veins, many R-Rs crossveins missing (especially on the hindwing), and the presence of only one series of gradate veins (the distal series) on the hindwing. This specimen remains an enigma.

##### Larvae and biology.

Monserrat and Freitas (2005) reported on some aspects of the developmental cycle and the third instar; most notably, they stated that the third instar carries debris, and eggs are laid in clusters. Unfortunately, voucher specimens for their study are not available. Thus, we cannot confirm the species identification for the report. Interestingly, the authors did not mention enlarged spiracles on the male abdomen (thus, this character could be consistent with *U. argentina*). However, the report does not indicate that there were markings on the forewing longitudinal veins that are typical of *U. argentina*. Thus, the identification of their specimens remains unconfirmed.

##### Known distribution.

ARGENTINA (northwest to central east): Provinces of Buenos Aires (Monserrat and Freitas 2005), Catamarca, La Rioja, Salta, Santiago del Estero, Tucumán.

##### Specimens studied

(in addition to type). Argentina. *Catamarca*: Andalgalá, 24-XII-1971, D. J. Brothers, at light (1M, 1F, SEMC); Ruta 46, 12 Km. O de Andalgalá, 28-III-2005, J. Torréns y P. Fidalgo, at light (1F, FSCA). *La Rioja*: Famatina, 23-XI-1975, L. Stange (1F, IFML); Guadacol, 1-3-XII-1983, L. E. Pena (1M, 6F, CAS); Patquia, 600 m, XII-1957 (1F, CAS). *Salta*: Cafayate, 1.I.1972, D. J. Brothers, at light (1F, SEMC). *Santiago del Estero*: Choya, 9 – XI-1961 (1F, CAS); El Pinto, XII-1956 (2F, CAS); Fernandes, II-1957 (5F, 1M, CAS). *Tucumán*: Amaicha, L. Stange, 20-XI-1966 (1M, FSCA); 11 km. W. Las Cejas, 15-VIII – 22-IX-1963, L. Stange (1M, FSCA).

#### Ungla chacranella

Taxon classificationAnimaliaNeuropteraChrysopidae

(Banks, 1915) comb. n.

Figs 107 , 108 , 109 , 110 , 111 , 112 , 113 , 114 , 115 , 116 , 117 , 118 , 119

**Figure 107.**
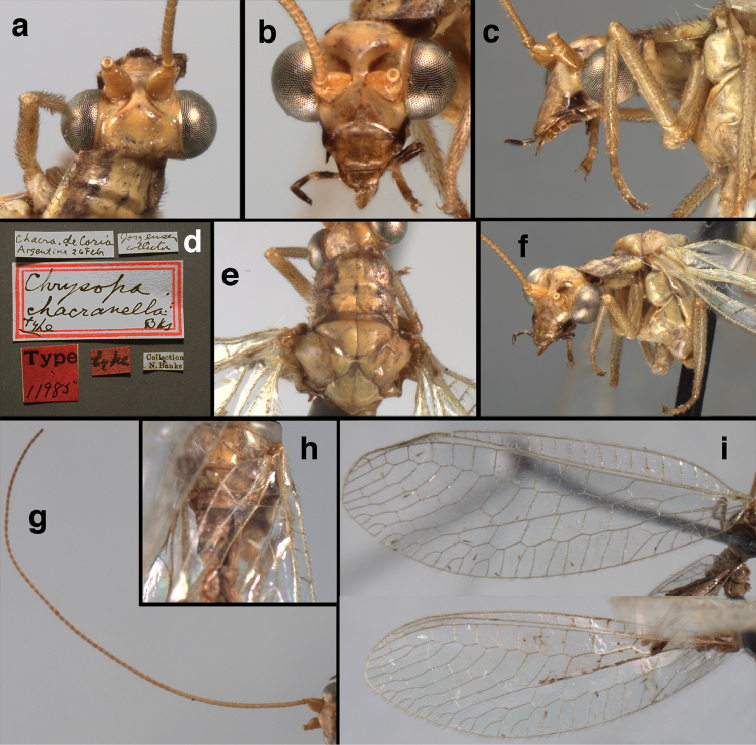
*Chrysopa chacranella* Banks: Features, (**a**) head, dorsal (**b**) head, frontal (**c**) head, prothorax, lateral (**d**) labels (**e**) prothorax, mesothorax, dorsal (**f**) head, thorax, frontolateral (**g**) antenna (**h**) metathorax, dorsal (**i**) wings (Mendoza, Chacras de Coria, holotype, female, MCZ).

**Figure 108.**
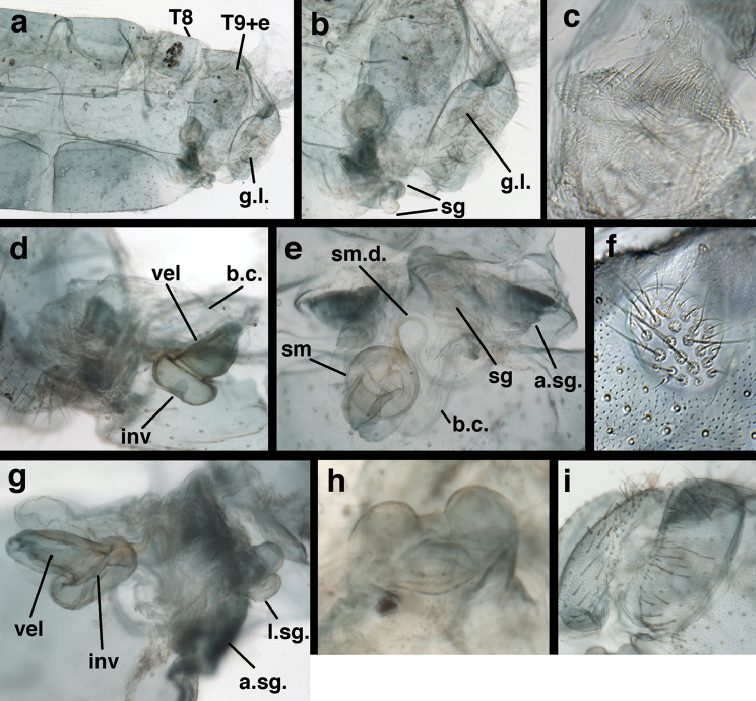
*Chrysopa chacranella* Banks: Female abdomen and genitalia, (**a**) segments A7-terminus, lateral (**b**) terminalia, lateral (**c**) transverse sclerite with ridged striations (internal to gonapophyses laterales) (**d**) spermatheca, lateral (**e**) genitalia, ventral (**f**) callus cerci (**g**) genitalia, dorsolateral (**h**) subgenitale, ventral (**i**) gonaphophyses laterales, posteroventral. **a.sg.** lateral arm of subgenitale **b.c.** bursa copulatrix **cr** crumena **g.l.** gonapophysis lateralis **inv** spermathecal invagination **l.sg.** lobe of subgenitale **sg** subgenitale **sm** spermatheca **sm.d.** spermathecal duct **T8** eighth tergite **T9+e** ninth tergite and ectoproct **vel** velum (Argentina; **a–c, e–i** holotype, Mendoza, Chacras de Coria, MCZ; **d** Salta, FSCA) .

**Figure 109.**
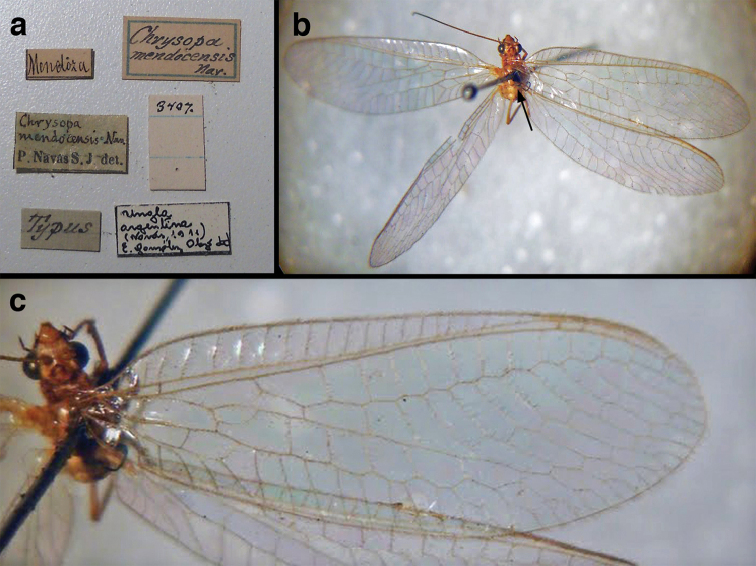
*Chrysopa mendocensis* Navás: Features, (**a**) labels (**b**) habitus, dorsal, abdomen missing (**c**) forewing (Argentina, Mendoza, lectotype, sex unknown, MLPA).

**Figure 110.**
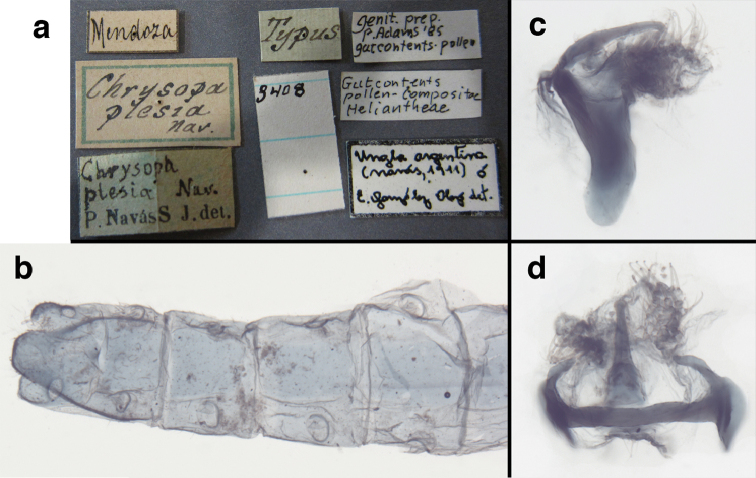
*Chrysopa plesia* Navás: Features, (**a**) labels (**b**) abdomen, dorsal, (**c**) gonarcus, lateral (**d**) gonarcus, dorsal (Argentina, Mendoza, lectotype, male, MLPA).

**Figure 111.**
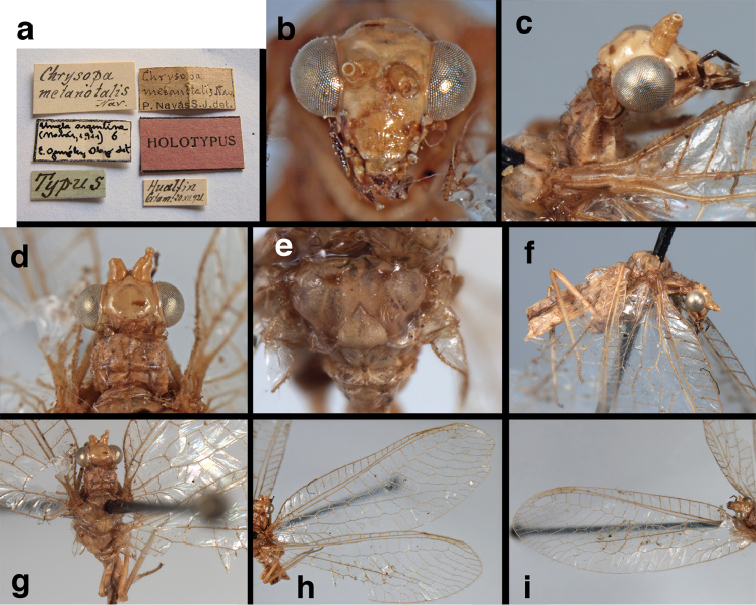
*Chrysopa metanotalis* Navás: External features, (**a**) labels (**b**) head, frontal (**c**) head, prothorax, lateral (**d**) head, prothorax, dorsal (**e**) metathorax, dorsal (**f**) body, lateral (**g**) body, dorsal (**h**) forewing (**i**) hindwing (Argentina, Catamarca, lectotype, male, MACN).

**Figure 112.**
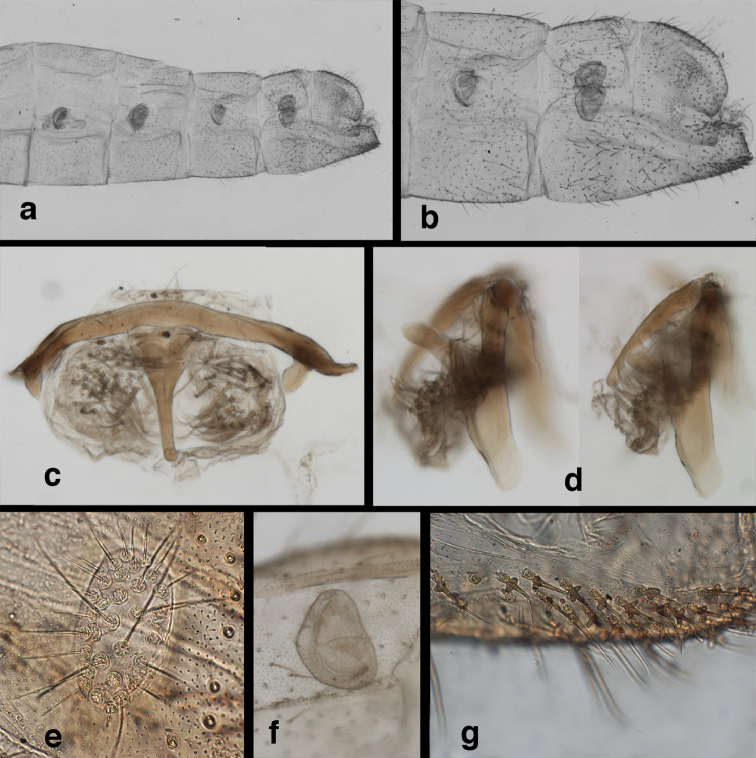
*Chrysopa metanotalis* Navás: Male abdomen and genitalia, (**a**) segments A5-terminus, lateral (**b**) segments A7-terminus, lateral (**c**) gonarcal complex, dorsal (**d**) gonarcal complex, left: posterolateral, right: lateral (**e**) callus cerci (**f**) spiracle, A8 (**g**) enlarged, flanged setae on distolateral margin of S9 (Argentina, Catamarca, lectotype, MACN).

**Figure 113.**
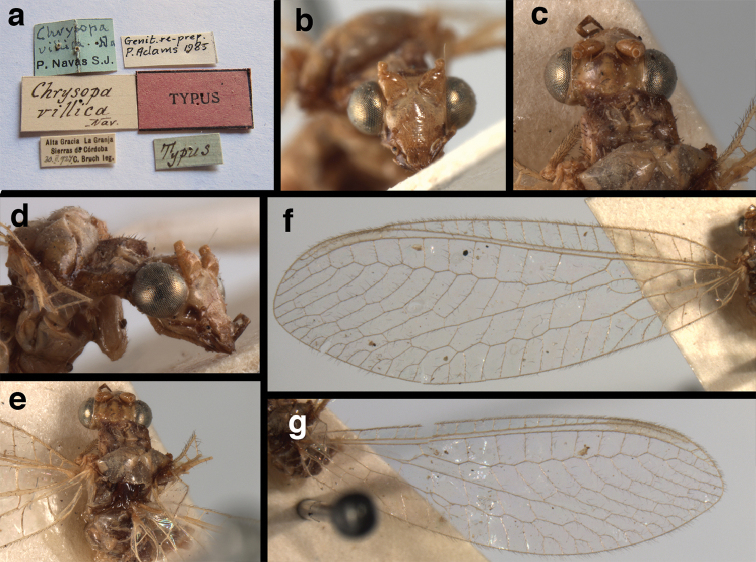
*Chrysopa villica* Navás: External features, (**a**) labels (**b**) head, frontal (**c**) head, prothorax, dorsal (**d**) head, prothorax, frontolateral (**e**) thorax, dorsal (**f**) forewing (**g**) hindwing (Argentina, Córdoba, lectotype, male, MACN).

**Figure 114.**
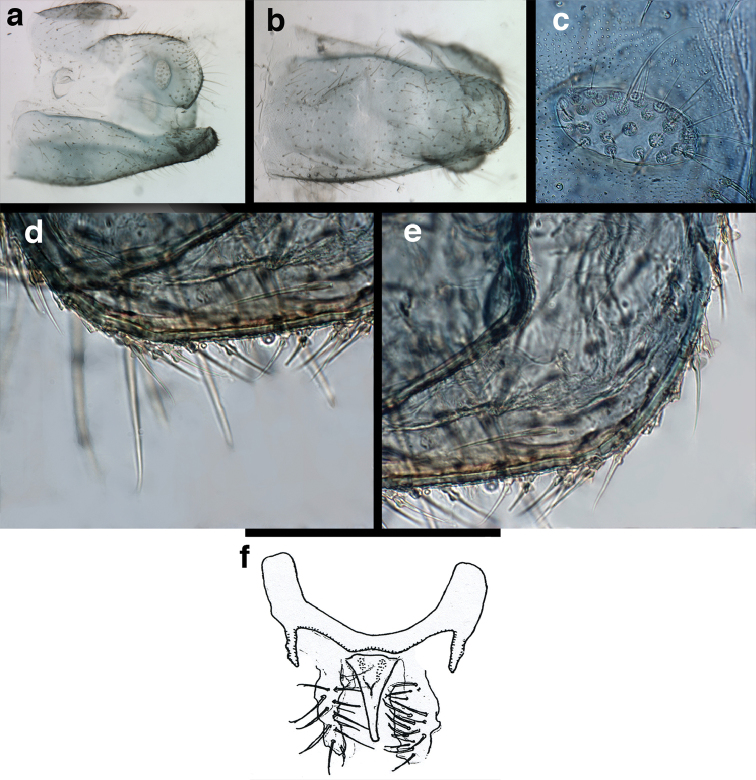
*Chrysopa villica* Navás: Male abdomen and genitalia, (**a**) segments A8-terminus, lateral, dissected and partially disarticulated (**b**) segments A8-terminus, ventral (**c**) callus cerci (**d, e**) enlarged, flanged setae on distal margin of S9 (**f**) sketch of gonarcal complex (dorsal), from unpublished notes by P. A. Adams (Argentina, Córdoba, lectotype, MACN).

Chrysopa chacranella Banks, 1915. *Proc. Acad. Nat. Sci. Philadelphia* (“1914-1915”) 66: 626; “Chacra di Coria, Argentine, 26 February (Jensen-Haarup)”. Penny 1977: 17 (list); Brooks and Barnard 1990: 279 [list, as “ ‘Chrysopa’ incertae sedis (? Ungla”)]; Oswald 2015 (catalog). **Holotype** (Figs 107, 108). MCZ, female (examined). Banks did not mention more than one specimen, and the single specimen in the collection is labeled as the type in Banks’ hand. We consider it to be the holotype by original designation. The type locality, Chacras de Coria, is in the northeastern region of Argentina: Province of Mendoza, Luján de Cuyo (~900 m). **Support for generic placement.** The C. chacranella type specimen expresses the external and internal generic level features that characterize Ungla.
Chrysopa mendocensis Navás, 1918. *Physis, Rev. Soc. Argentina Cienc. nat*. 4: 85–86; “Mendoza, BRUCH (Mus. de La Plata)”. Stange 1967: 36 (catalog); Penny 1977: 19 (list); Brooks and Barnard (1990: 280, list, as “ ‘Chrysopa’ incertae sedis”); González Olazo 1996: 380 (catalog, as Ungla sp.); Oswald (2015, as Chrysopa). **syn. n. Lectotype** (Fig. 109). MLPA, sex unknown (examined). Navás did not mention how many specimens he used to make the original description. Both Stange (1967: 36) and González Olazo 1996: 380) reported that the type was in the MLPA, and González Olazo noted that the abdomen was missing. Also, González Olazo (1996: 380) identified the specimen as a species of Ungla. To help stabilize the nomenclature of the genus, this specimen, with labels as in Fig. 109 and abdomen missing, is designated as the lectotype (des. CAT). **Support for synonymy.** We base our synonymy of C. mendocensis with U. chacranella on the shape and venation of the wings and the overall external features of the type. First, although Navás (1918) in his description of C. mendocensis stated that three veins in the subcostal area are black, we and Adams (unpublished notes) found that all the veins, including the subcostals, are green. Totally green venation is typical of U. chacranella. Second, the head and facial coloration, markings, size, etc. of the C. mendocensis type are indistinguishable from those of U. chacranella. Similarly, because of the above features, we disregarded González Olazo’s label of “Ungla argentina (Navás)” on the type specimen.
Chrysopa plesia Navás, 1918. *Rev. Soc. Arg. Cienc. nat.*, 4: 86-87; “Mendoza, BRUCH (Mus. de La Plata)”. Stange 1967: 36 (catalog); Penny 1977: 20 (list); Brooks and Barnard 1990: 280 (list, as “ ‘Chrysopa’ incertae sedis”); González Olazo 1996: 380 (catalog, synonymy with U. argentina); Oswald 2015 (catalog). **syn n. Lectotype** (Fig. 110). MLPA, male (examined). Head missing, genitalia in a vial with glycerine. Stange (1967: 36) reported a male, and González Olazo (1996: 380) reported a single specimen (sex unspecified). We examined the external features briefly and the abdomen in detail. The locality data are as reported in the original description, and the identification label is in Navás’ hand. The specimen carries an additional identification label (Ungla argentina) by González Olazo (Fig. 110a). Here, this specimen is designated as the lectotype of C. plesia (des. CAT). **Support for synonymy.** Adams’ (unpublished notes) stated that this specimen resembles “C. binaria” (now a synonym of U. confraterna). However, he noted several differences; for example, he considered the venation to be paler than that of “C. binaria”, and he noted that the vertex of the C. plesia type has a pair of reddish marks posterolaterally, which are not present on the C. binaria type. It is surprising that he made no mention of the enlarged abdominal spiracles, which are prominent on the C. plesia type. All of the above features [pale wing venation, reddish marks on vertex, and large abdominal spiracles (males)] are typical of U. chacranella.
Chrysopa metanotalis Navás, 1924. *Rev. Chil. Hist. nat.* 27: 114 (“1923”); “República Argentina: Hualfín (Catamarca), 20 de Diciembre de 1921 (col. Bruch)”. Stange 1967: 36 (catalog); Penny 1977: 19 (list); Brooks and Barnard 1990: 280 (list, as “ ‘Chrysopa’ incertae sedis”); González Olazo 1996: 380 (catalog, as Ungla sp.); Oswald (2015, as Chrysopa). **syn n. Lectotype** (Figs 111, 112). MACN, male (examined). González Olazo (1996: 380) recognized the type as a species of Ungla, and he attached an “Ungla argentina” identification label to the specimen, but he did not formalize the synonymy. To help stabilize the nomenclature of the genus, here this specimen is designated as the lectotype of C. metanotalis (des. CAT). The type locality, Hualfín, is in northeastern Argentina: Belén, Catamarca (~1850 m). **Support for synonymy.** Unlike Navás (1923) in his original description, we did not observe dark cross veins on the wings; they were pale as in U. chacranella. We base the synonymy on the shared head and metanotal markings, similarities in the wings (size, shape, venation, and pale veins), the enlarged abdominal spiracles, and the male genitalia.
Chrysopa villica Navás, 1929. *Rev. Soc. Entomol. Arg.* 2: 222; “República Argentina; La Granja, Alta Gracia, 30 de febrero de 1924. Col. Bruch”. Stange 1967: 38 (catalog); Penny 1977: 21 (list); Brooks and Barnard 1990: 280 (list, as “ ‘Chrysopa’ incertae sedis”); Oswald (2015, as Chrysopa). **syn n. Lectotype** (Figs 113, 114). MACN, male (examined). Stange (1967: 38) and Adams (unpublished notes) reported that a type, from the province of Córdoba, is in the MACN; to stabilize the nomenclature of the genus, here this specimen is designated as the lectotype of C. villica (des. CAT). When we examined the specimen (a male), parts of the abdomen were cleared and in a vial of glycerine; unfortunately, the genitalia had been lost sometime after Adams examined the specimen. [Apparently Stange’s (1967: 38) note of the type as a female is in error.] The original description mentions both La Granja and Alta Gracia as the type locality, and the type bears a label with these same two localities. Both places are in the Province of Córdoba, within ~73 km of each other: La Granja is a town in the Department of Columbus (~730 m), and Alta Gracia is a city in the Department of Santa Maria (~550 m). **Support for synonymy.** We based the synonymy on our examination of the external features of the type and the structures that are present on what remains of the abdomen, supplemented with Adams’ unpublished notes on the genitalia.

##### Diagnosis.

Adults of *U. chacranella* are distinguished by the following external features: dark brown to black genal marks; antenna, including pedicel, pale, without dark ring; wing veins, including crossveins and gradates, light green (an occasional anal vein may appear light brown to brown); wings relatively wide, with rounded to subacute tips.

Several series of specimens in the CAS and FSCA allowed us to associate males and females of this species. Males have abdominal spiracles greatly enlarged; S8+9 elongate, broad (ventral view), shallow, protruding well beyond T9+ect (lateral view); tip of S8+9 with flanged terminal setae; gonarcus with narrow bridge (dorsal view), slender apodemes with long, digitiform mesal process, elongate, narrow mediuncus, gonosaccus bilobed, each lobe with field of robust, widely spaced, terminally bent, gonosetae with large chalazae. The female has spermatheca with a large velum, small invagination; spermathecal duct elongate, curved without tight coils; and subgenitale (ventral view) short, stubby, and with well rounded, separated lobes.

This species resembles *U. confraterna*. However, *U. confraterna* has at least some dark crossveins and slightly acute wing tips. Males of *U. confraterna* also lack the enlarged spiracles, and the dorsal (mesal) portion of the gonarcal bridge is robust and relatively straight, whereas in *U. chacranella* males the spiracles are greatly enlarged and the gonarcal bridge is slender and more rounded dorsally.

##### Redescription.

Head: vertex smooth, slightly depressed mesally, inverted U-shaped marking on vertex reddish brown, usually prominent, often separated mesally but with pigmented marking joining mesally, extending anteriorly toward but not reaching area between scapes; anteromesal margin of dorsal antennal fossa also with dark reddish brown mark; area between eyes and posterior half of vertex cream-colored, unmarked; frons cream-colored with elongate, narrow, linear marking from between antennae to center of frons, anterior margin of frons with or without reddish brown, transverse stripe; clypeus cream-colored to tan; gena, posterolateral margin of the clypeus with dark brown stripe; tentorial pits with brown margins. Antenna with scape cream-colored to light tan, unmarked; pedicel cream-colored to tan, with distal ring of brown; flagellum cream-colored to light tan basally, becoming darker distally; maxillary palp with basal two segments pale, distal segments brown; labial palp with basal segment pale, distal two segments brown.

Prothorax green mesally, with wide, brown, lateral stripes, thin, brown mesal stripe; transverse furrow in posterior region, not reaching lateral margins; short, dark setae throughout. Mesothorax, metathorax yellowish brown with light brown markings. Measurements: head width, 1.2–1.4 mm; ratio head width : eye width, 2.4–2.6 : 1; prothoracic length, 0.5–0.8 mm; prothoracic width, 0.8–1.0 mm.

Forewing, hindwing clear, hyaline, without fumose areas, with slender venation; stigma lightly opaque, with three to six light to dark brown subcostal crossveins below stigma surrounded by dark brown marks; all veins, except anal veins, cream-colored to light green, without suffusion. Forewing 9.8–13.4 mm long, 3.3–4.8 mm wide (ratio, L : W = 2.8–3.1 : 1); height of tallest costal cell 0.6–1.0 mm (cell number 6–8); width of first intramedian cell 0.7–0.9 mm; 10–12 radial cells (closed cells between R and Rs); third gradate cell 1.07–1.48 mm long, 0.35–0.52 mm wide (ratio, L : W =2.86–3.50); fourth gradate cell 0.88–1.72 mm long, 0.32–0.48 mm wide (ratio, L : W = 2.55–3.27); 4–5 Banksian cells (b cells), 4–5 b’ cells; 5–6 inner gradates, 5–6 outer gradates. Hindwing 8.9–12.0 mm long, 2.8–4.1 mm wide (ratio, L : W = 2.9–3.3 : 1), 9–12 radial cells, 3–4 Banksian (b) cells, 4–5 b’ cells, 4–6 inner gradates, 5–7 outer gradates.

Male. Abdomen with greatly enlarged spiracles having large flaps on opening (e.g., A7: maximum spiracle diameter ~0.23–0.30× length of sternite); T9+ectoproct short, with dorsal invagination extending almost to anterior margin of T9, lateral margins of invagination straight to convex; dorsal margin rounded distally; posterior half of ventral margin lightly sclerotized – sclerotization, contiguous with and extending distally from sclerotization around circumference of callus cerci, extending posteriorly in small, rounded knob; callus cerci oblong; subanal plate small, with ~ eight setae. Sternite S8, S9 fused, with line of fusion distinct, terminating in sclerotized, elongate, plate-like extension; dorsal margin sclerotized for entire length, heavier throughout terminal ~one third; terminal setae robust, flanged. Gonarcus with bridge slender, lateral apodemes narrow (lateral view), slightly expanded, rounded distally; process on side of lateral apodeme extending dorsally (lateral view), turning inward distally (frontal view) from gonarcal arm; mediuncus long, narrow, with dorsal margin straight, terminus with small knob; bilobed gonosaccus with large patch of well-spaced gonosetae on enlarged bases – anterobasal gonosetae slightly smaller, shorter, on smaller bases than posterodistal gonosetae; hypandrium internum U-shaped with arms straight (frontal view), bending upward (lateral view), comes delicate.

**Figure 115.**
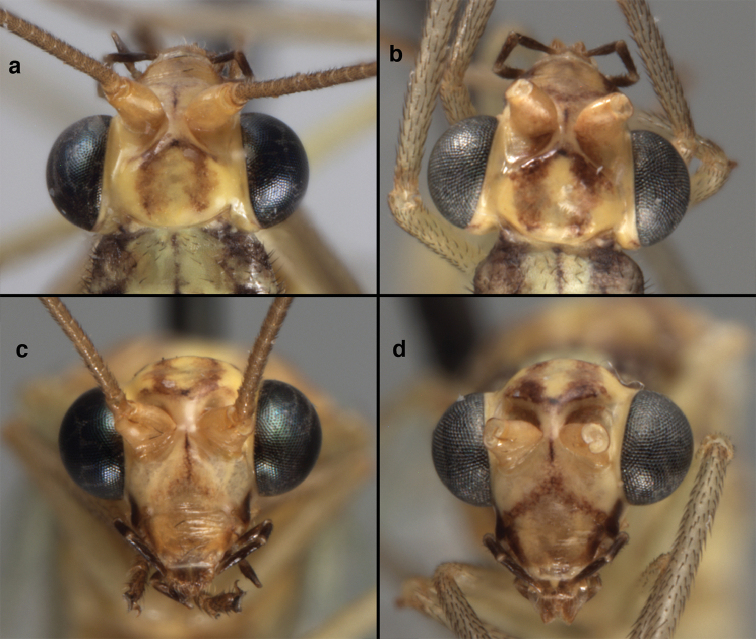
*Ungla chacranella* (Banks): Variation in head markings, (**a, b**) head, dorsal (**c, d**) head, frontal (Argentina, La Rioja, male, FSCA).

##### Variation.

The species shows large variation in both size and coloration. For example, forewing length ranges from 9.8 to 13.4 mm, and the number of radial cells from 10-12 (forewing) and 9-12 (hindwing). In addition, the enlarged spiracles on the abdomen vary in size from ~0.23 to 0.30. Some of the variation in dorsal and frontal markings on the head is illustrated in Fig. 115.

##### Known distribution.

ARGENTINA (northwest): Provinces of Catamarca, Córdoba, La Rioja, Mendoza, Salta, Tucumán.

##### Specimens studied

(in addition to the types above). Argentina. *La Rioja*: Santa Cruz, 1600 m, 1/XII/2002, L. A. Stange (2M, FSCA). Cuesta de Miranda, “25-11-939 (Sic!)”, Biraben-Scott (1M, APTA). *Mendoza*: Rio Mendoza, 1600 m, 5-6-XII-1983, L. E. Pena (8M, 3F, CAS). *Salta*: Yacochuya cerca de Cafayate, 15-XII-1973, L. Stange (13M, 6F, 1?, FSCA); N. Cafayate, Yacochuya, 2-XII-1970, C. Porter-L. Stange (1F, FSCA); Cachi, 22-I-1966, L. Stange (1?, FSCA). *Tucumán*: Tafi de Valle, 7-I-1967 (1M, 1F, USNM).

##### Note.

A series of 11 unidentified female specimens (Figs 71, 72) from Bolivia that resemble both *U. chacranella* and *U. pallescens* remain unidentified. See note under *U. pallescens*.

**Figure 116.**
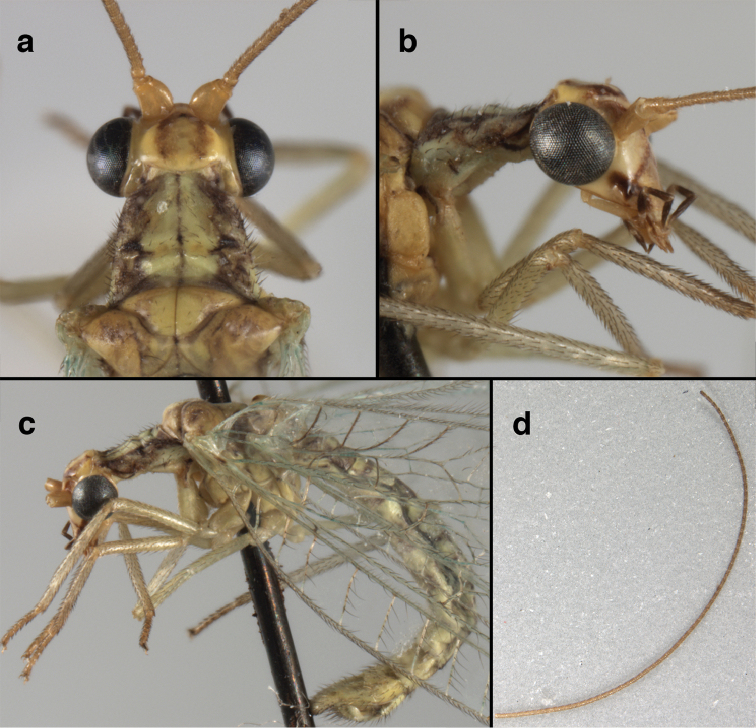
*Ungla chacranella* (Banks): External features, (**a**) head, prothorax, mesothorax (part), dorsal (**b**) head, prothorax, lateral (**c**) body, lateral (**d**) antenna (all: Argentina, La Rioja, male, FSCA).

**Figure 117.**
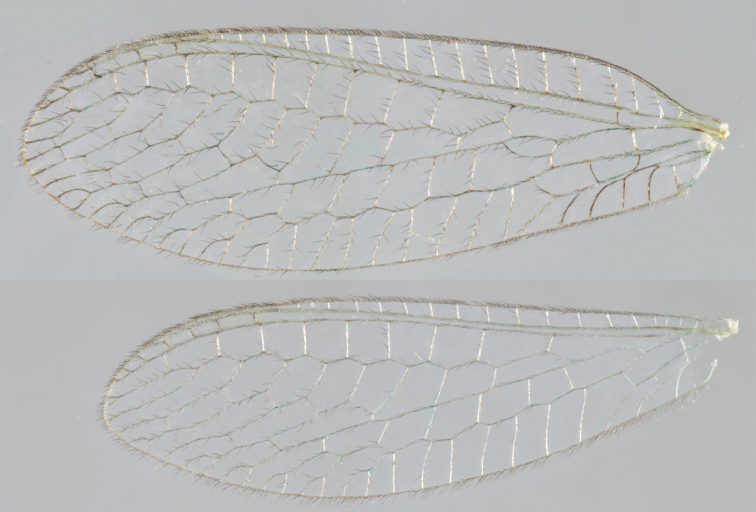
*Ungla chacranella* (Banks): Wings (Argentina, La Rioja, male, FSCA).

**Figure 118.**
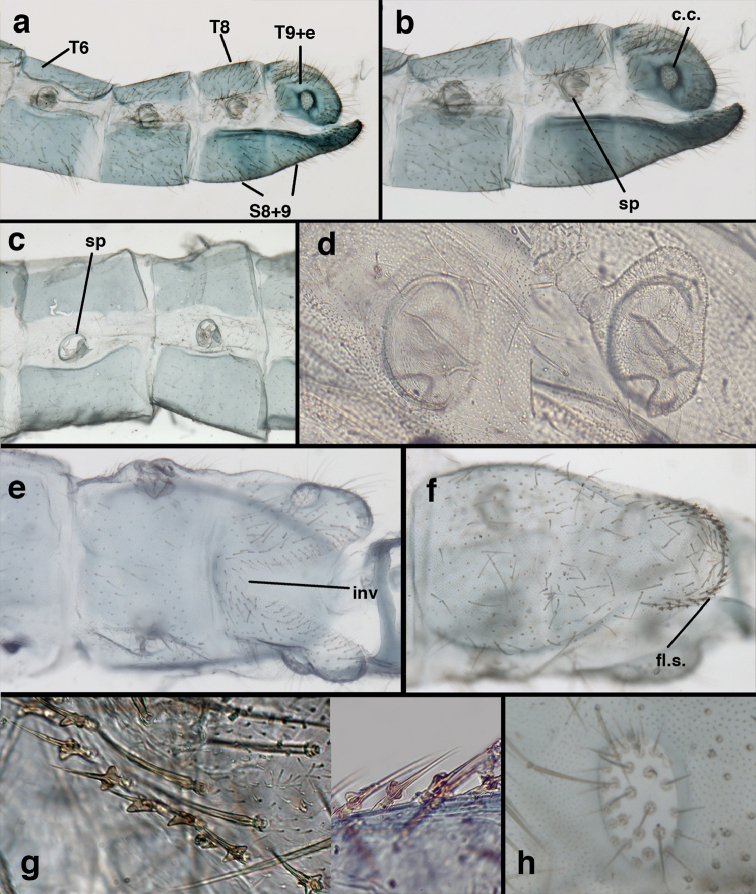
*Ungla chacranella* (Banks): Male abdomen, (**a**) segments A6-terminus, lateral (**b**) segments A7-terminus, lateral (**c**) segments A6 and A7, lateral (**d**) spiracle, left: opening on integumental surface, right: internal, immediately below integumental opening [Note enlarged flap.] (**e**) tergite T9 + ectoproct, dorsal (**f**) fused sternites 8+9, ventral, with robust, flanged setae along margin of S9 (**g**) enlarged, flanged setae on margin of S9, left, right (**h**) callus cerci. **c.c.** callus cerci **fl.s.** flanged setae **inv** distal invagination of dorsal T9+ectoproct **sp** spiracle **S8+9** fused eighth and ninth sternites **T6, T8** sixth and eighth tergites **T9+e** ninth tergite + ectoproct (Argentina; **a, b, g-right** Mendoza, CAS; **c, d** La Rioja, FSCA; **e** Tucumán, USNM; **f, g-left, h** Salta, FSCA).

**Figure 119.**
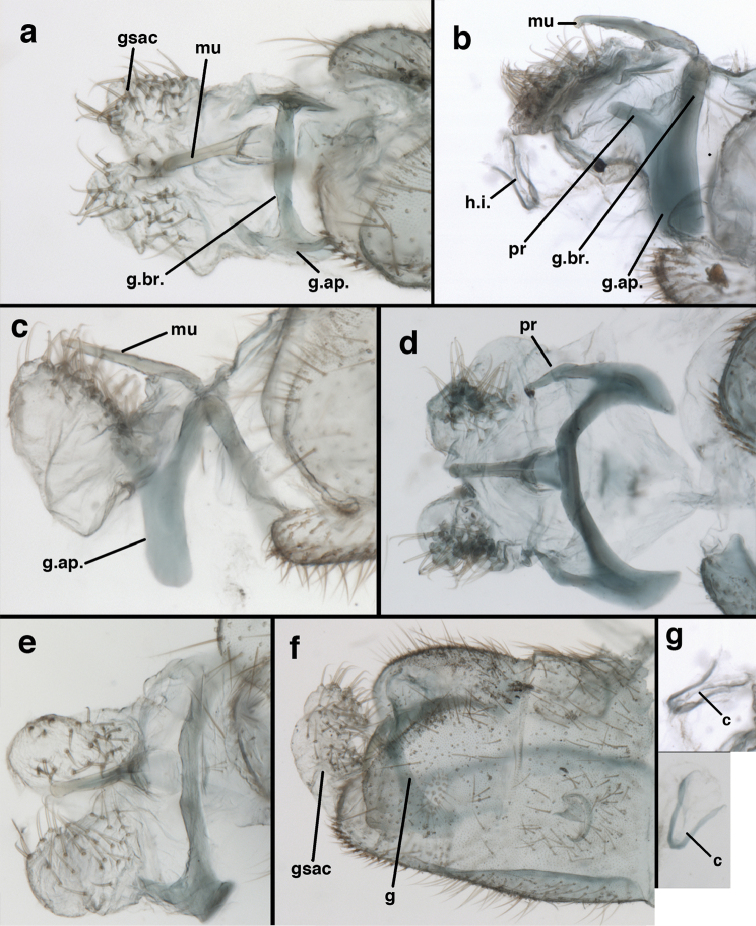
*Ungla chacranella* (Banks): Male genitalia, (**a**) gonarcal complex, dorsal (**b**) gonarcal complex, lateral, gonosaccus not fully expanded (**c**) gonarcal complex, posterolateral (**d**) gonarcal complex, posterodorsal (**e**) gonarcal complex, dorsal, gonosaccus fully expanded (**f**) terminal abdominal segments, dorsolateral, gonosaccus expanded, everted, gonarcus not everted (**g**) hypandrium internum (upper: slightly teneral, lower: mature). **c** comes **g** gonarcus **gsac** gonosaccus **g.ap.** gonarcal apodeme **g.br.** gonarcal bridge **h.i.** hypandrium internum **mu** mediuncus **pr** unarticulated process on frontal margin of gonarcal apodeme (Argentina; **a, c, e, f, g-upper** Salta, FSCA; **b, d** La Rioja, FSCA; **g-lower** Mendoza, CAS).

#### Ungla confraterna

Taxon classificationAnimaliaNeuropteraChrysopidae

(Banks, 1913)

Figs 120 , 121 , 122 , 123 , 124 , 125 , 126 , 127 , 128 , 129

**Figure 120.**
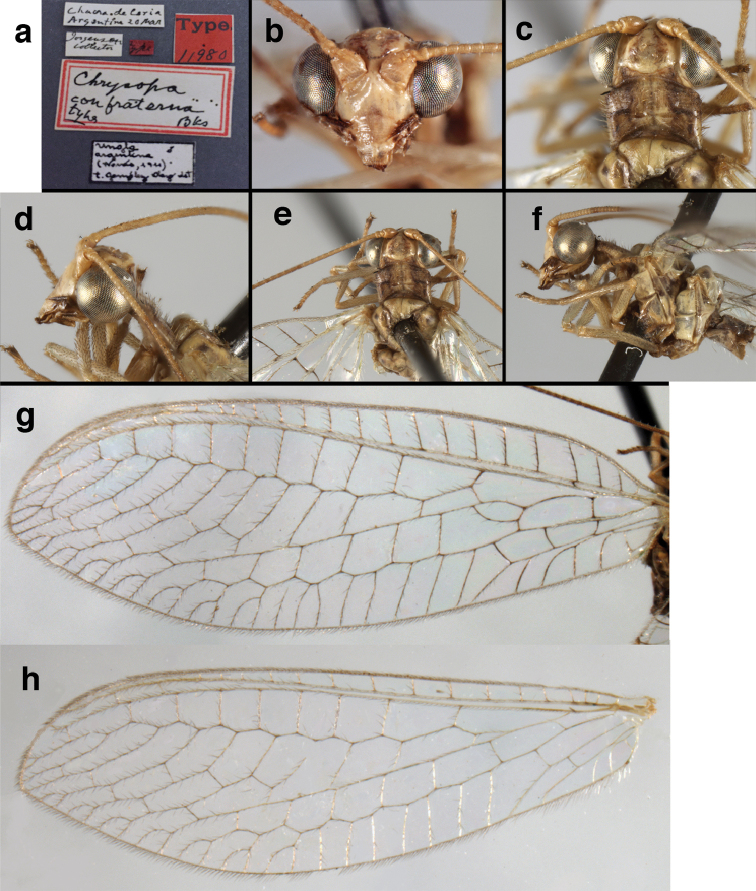
*Chrysopa confraterna* Banks: External features, (**a**) labels (**b**) head, frontal (**c**) head, prothorax, mesothorax (partial), dorsal (**d**) head, lateral (**e**) head, thorax, dorsal (**f**) head, thorax, lateral (**g**) forewing (**h**) hindwing (Argentina, Mendoza, Chacras de Coria, holotype, male, MCZ).

**Figure 121.**
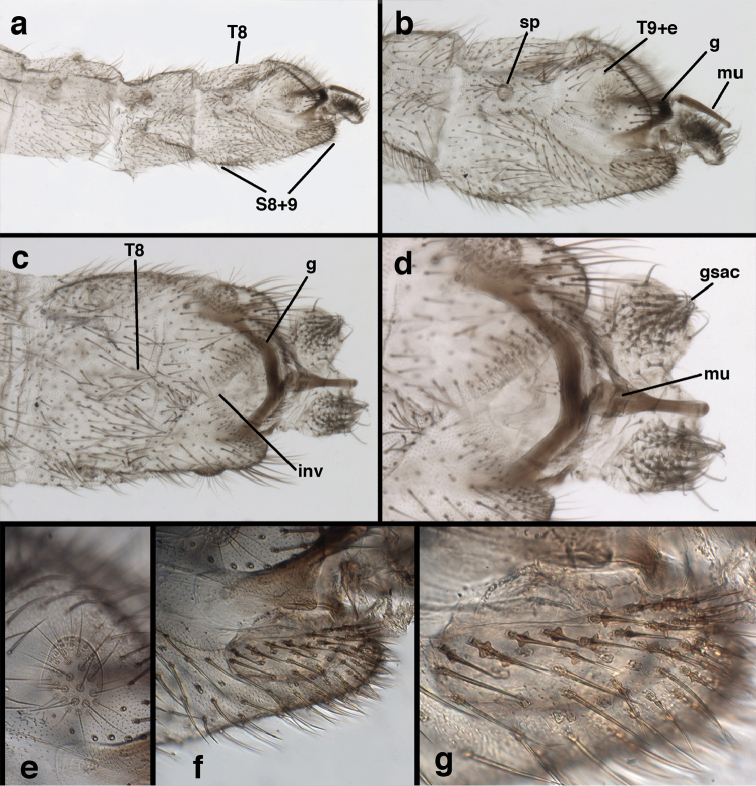
*Chrysopa confraterna* Banks: Male abdomen and genitalia, (**a**) segments A6-terminus, lateral (**b**) terminalia, lateral (**c**) terminalia, dorsal, gonarcus partially extruded posteriorly (**d**) terminus of T9+ectoproct, gonarcal complex (**e**) callus cerci (**f**) sternite 9 [Note demarcation between S8 and S9.] (**g**) sternite 9, lateral, with large, flanged setae. **g** gonarcus **gsac** gonosaccus **inv** distal invagination of dorsal T9+e **mu** mediuncus **sp** spiracle **S8+9** fused eighth and ninth sternites **T8** eighth tergite **T9+e** fused ninth tergite + ectoproct (Argentina, Mendoza, Chacras de Coria, holotype, MCZ).

**Figure 122.**
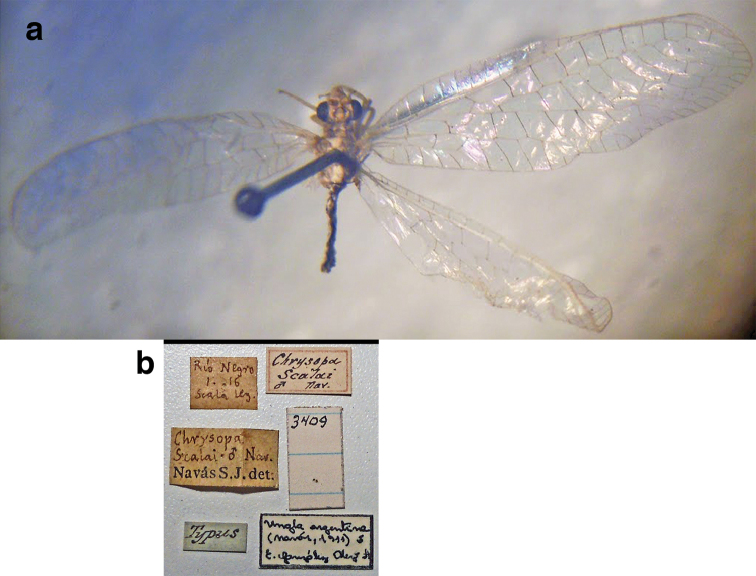
*Chrysopa scalai* Navás: Features, (**a**) habitus, dorsal (**b**) labels (Argentina, Rio Negro, lectotype, male, MLPA).

**Figure 123.**
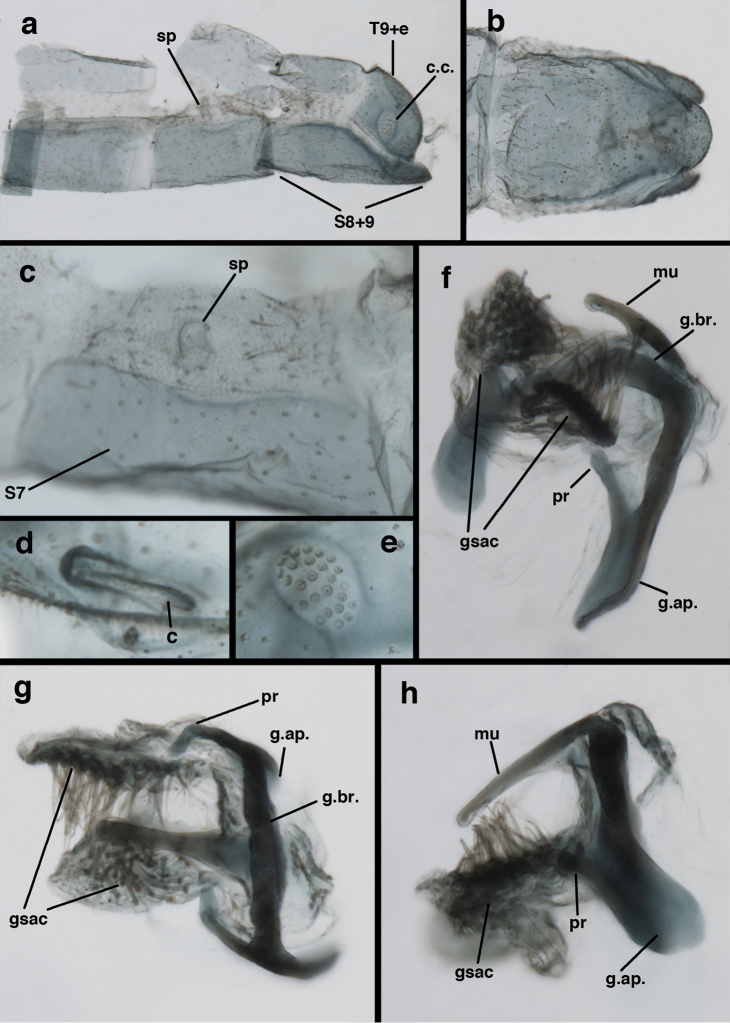
*Chrysopa scalai* Navás: Male abdomen and genitalia, (**a**) segments A6-terminus, lateral (**b**) fused sternites 8+9, ventral (**c**) segment A7, lateral (**d**) hypandrium internum (**e**) callus cerci (**f**) gonarcal complex, frontolateral (**g**) gonarcal complex, dorsal (**h**) gonarcal complex, lateral. **c** comes **c.c.** callus cerci **gsac** gonosaccus **g.ap.** gonarcal apodeme **g.br.** gonarcal bridge **mu** mediuncus **pr** unarticulated process on frontal margin of gonarcal apodeme **sp** spiracle **S7** seventh sternite **S8+9** fused eighth and ninth sternites **T9+e** ninth tergite + ectoproct (Argentina, Rio Negro, lectotype, MLPA).

**Figure 124.**
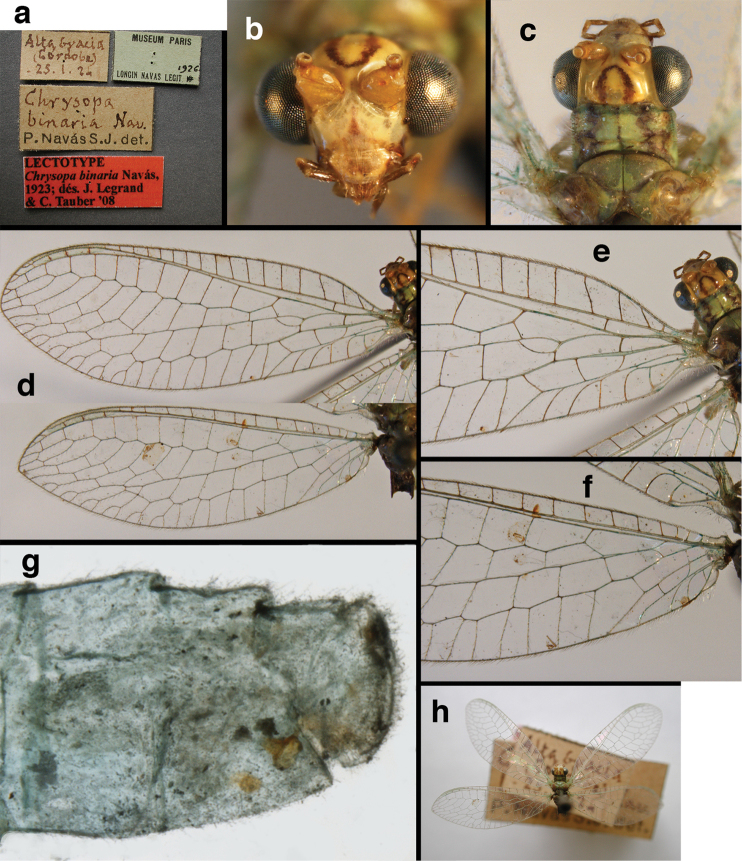
*Chrysopa binaria* Navás: External features, (**a**) labels (**b**) head, frontal (**c**) head, prothorax, dorsal (**d**) wings, left (**e**) base of forewing (**f**) base of hindwing (**g**) abdominal segments A6-terminus, lateral (**h**) habitus, dorsal (Argentina, Córdoba, lectotype, female, MNHN).

**Figure 125.**
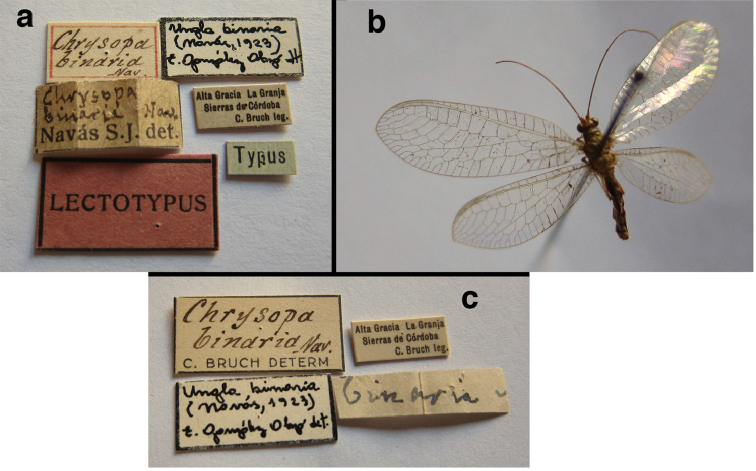
*Chrysopa binaria* Navás: (**a**) labels from paralectotype (**b**) habitus of female paralectotype, dorsal (**c**) labels of nontype specimen, from type locality, and determined by C. Bruch, collector of the lectotype (all: Argentina, Córdoba, MACN).

Chrysopa confraterna Banks, 1913. *Proc. Entomol. Soc. Wash.* 15: 140-141. “Chacra di Coris, Argentine, 26 February, 20 March”. Penny 1977: 17 (list). Suarius confraternus (Banks), by Adams 1975: 171. Ungla confraterna (Banks), by Brooks and Barnard 1990: 276 (catalog); Freitas 2007: 415 (key to adults); Oswald 2015. **Holotype** (Figs 120, 121). MCZ, male (examined); holoytpe by monotypy. Body discolored but otherwise in good condition, bearing Banks’ type label. The type locality, Chacras de Coria, is in the northeastern region of Argentina: Province of Mendoza, Department of Luján de Cuyo, Mendoza (~900 m). The specimen is teneral and thus the abdomen still has most of the setae; however, the integument is torn in several places. The MCZ has a second specimen, from Peru, identified as this species by Banks, but it was not mentioned in the description. Unfortunately, its abdomen is missing. We suspect that it is a pale, somewhat faded specimen of U. siderocephala; i.e., the U-shaped mark on its vertex is heavy, and each arm of the mark has a (thin) lateral extension to the eye – both characteristics of U. siderocephala.
Chrysopa scalai Navás, 1917. *Physis, Rev. Soc. Arg. Cienc. Nat.* 3 :195-196; “Río Negro, Enero de 1916, Prof. A. SCALA leg. (Mus. de La Plata)”. Stange 1967: 37 (catalog); Penny 1977: 20 (list); Brooks and Barnard 1990: 280 (list, as “ ‘Chrysopa’ incertae sedis”); González Olazo 1996: 381 (catalog); Oswald 2015 (catalog). **syn n. Lectotype** (Figs 122, 123). MLPA, male (examined). The type locality “Rio Negro” probably refers to the “Territorio del Rio Negro” which at the time of the collection was included within the Province of Patagonia. Today, Rio Negro is a separate province in the northern part of the Patagonian Region. Because Navás did not state how many specimens he had, and to preclude any confusion, the specimen that we photographed and that bears the number “3409” is hereby designated as the Chrysopa scalai lectotype (des. CAT). A red lectotype label has been applied to the specimen. Note: The specimen number (#3407) that González Olazo (1996: 381) reported for C. scalai is in error; it should read “3409”. We examined the specimen briefly during a visit to the MLPA, and subsequently examined the cleared abdomen in detail. The abdomen is in fairly good condition except that most of the setae are absent and the gonarcus is distorted. **Support for synonymy.** Although González Olazo placed an U. argentina identification label on the C. scalai type, he did not publish a synonymy (see González Olazo (1996: 381). Our examination of the lectotype showed that the specimen has significant differences that preclude a synonymy with U. argentina. Notably, the posterovental corner of the ectoproct has a small knob that protrudes posteriorly, the gonarcus is thin and bears a long process, and the mediuncus is long, narrow, and straight (without a sinuous dorsal margin). All of these features are characteristic of U. confraterna. The specimen also differs from C. chacranella in that the spiracles are not enlarged, and the wing venation has notable brown areas.
Chrysopa binaria Navás, 1923. *Arxius Inst. Ciènc., Inst. Est. Catalans, Sec. Ciènc.* (“1919”b) 7: 191; “República Argentina: Alta Gracia (Córdoba). 24–28 de gener, 17 de febrer de 1922. Atreta per la llum. Bruch (Col. m.)”. Navás 1926: 108 (dist); Stange 1967: 31 (catalog); Penny 1977: 16 (list). Ungla binaria (Navás), by Brooks and Barnard 1990: 240, 276 (tax, list); González Olazo 1996: 378 (catalog); Monserrat and Freitas 2005: 168-171 (redesc, larval desc, biol); Freitas 2007: 415 (key to adults); Reguilón 2010: 78-86 (larval desc, biol.); Tauber et al. 2014: supplementary material (list); Oswald 2015 (catalog). **syn n. Lectotype** (Fig. 124). MNHN, female (examined); lectotype designated by Legrand et al. (2008: 119). Note: Navás reported collection dates in 1922. However, the year on the lectotype label is not clear; Legrand et al. (2008) interpreted it as 1924 (probably an error). The type locality is in northern central Argentina: Province of Cordoba, Department of Santa Maria. In addition to the lectotype, there is at least one other type specimen; Stange (1967: 31) and González Olazo (1996: 378) reported a female type in the MACN. We have seen this specimen and we consider it to be a paralectotype (Fig. 125a, b); it is not missing its head as reported by Legrand et al. (2008: 119). Its locality label has been changed from the original, and it does not include a collection date. However, it bears an original determination label in Navás’ hand. Also, in the MACN, there is another specimen from the type locality, determined by the collector of the type specimen, C. Bruch (labels, Fig. 125c). It bears the same collection label (without date) as the paralectotype; we found no evidence that this specimen was seen by Navás, and we do not consider it to be part of the type series. **Support for synonymy.** The C. binaria type shares external and internal features, especially patterns of wing venation and coloration, head and body markings, with those of U. confraterna females. Also, we found nothing notable in the female abdomen or genitalia that would differentiate the species.

##### Diagnosis.

Externally, the dorsal head markings and largely green longitudinal veins marked with brown at intersections distinguish *U. confraterna* from most of the other small Argentinian species (*U. annulata*, *U. argentina*, and *U. chacranella*). Its lack of a prominent stripe on the gena and clypeal margin distinguishes it from *U. elbergi*. However, the most reliable characters for identifying any of these species are in the male abdomen and genitalia. For example, *U. confraterna* is notable because of the unique size of its abdominal spiracles (diameter ~ 0.08 mm, ~0.1× length of segment) – slightly larger than those of most chrysopine adults, including *U. argentina* (diameter ~0.03 mm, ~0.03× length of segment), and considerably smaller than those of the other small Argentinian species, all of which have spiracles with a diameter of > 0.15 mm, > 0.2× length of segment. The shapes of its terminal segments and gonarcal complex are also distinctive.

##### Redescription.

Head cream-colored, with vertex smooth, often shiny; inverted U-shaped marking on vertex reddish brown, usually prominent, narrowing and sometimes separated mesally, not extending anteriorly to area between scapes; anteromesal margin of dorsal antennal fossa with reddish brown mark (sometimes pale); area between eyes and posterior half of vertex cream-colored, unmarked; frons cream-colored, with markings absent or light reddish brown stripe mesally, or reddish tinge along anterior margin; clypeus cream-colored to tan, with dark brown spot laterally; gena with brown mark not touching eyes, approaching clypeus; tentorial pits with light brown margins. Antenna with scape cream-colored to light tan, unmarked; pedicel cream-colored to tan, with distal ring of brown; flagellum cream-colored to light tan basally, becoming darker distally; maxillary palp with basal two segments pale, mesal two segments brown dorsally, laterally, distal segment brown; labial palp with basal segment pale, distal two segments with brown.

Prothorax relatively short, green mesally, with wide, brown, lateral stripes, thin, brown mesal stripe; transverse furrow in posterior region, not reaching lateral margins; with short, dark setae throughout. Mesothorax, metathorax yellowish brown with light brown markings. Measurements: head width: 1.2–1.3 mm; ratio head width : eye width: 1.7–2.3 : 1; prothorax length: 0.4–0.6 mm; prothorax width: 0.9–1.1 mm.

Forewing, hindwing clear, hyaline, without fumose areas, with slender venation; stigma lightly opaque to clear, with four to five light to dark brown subcostal crossveins below stigma, area surrounding crossveins unmarked; longitudinal veins usually mostly green, with brown at intersections, sometimes with extensive brownish sections; transverse veins, crossveins mostly brown to golden brown. Forewing 10.3–12.4 mm long, 3.6–4.4 mm wide (ratio, L : W = 2.8–2.9 :1); height of tallest costal cell 0.6–0.8 mm (cell number 6–8); length of first intramedian cell 0.7–1.0 mm; 9–12 radial cells (closed cells between R and Rs); 4 Banksian cells (b cells), 4 b’ cells; 3–6 inner gradates, 5–7 outer gradates. Hindwing 9.1–11.1 mm long, 3.0–3.7 mm wide (ratio, L : W = 3.0–3.2 : 1), 9–11 radial cells, 3 Banksian (b) cells, 4 b’ cells, 2–5 inner gradates, 4–5 outer gradates.

Male. Abdomen with slightly enlarged spiracles (e.g., A7: spiracle diameter ~0.10× length of sternite); T9+ectoproct short, with dorsal invagination shallow, extending approximately one half the distance to anterior margin of T9, lateral margins of invagination straight to slightly convex; dorsal margin rounded distally, with posteroventral margin extended distally in well defined knob; ventral margin lightly sclerotized beyond callus cerci; callus cerci large, ovate, circumference lightly sclerotized, sclerotization contiguous with that on ventral margin of ectoproct. S8+9 fused, with line of fusion not demarcated; setae on ventral surface relatively long, not dense; dorsal margin lightly sclerotized, except near base; terminus extended, upturned distally, flat, plate-like, well sclerotized, bearing numerous, large, flanged setae along dorsal margins and heavy, unflanged setae distomesally. Gonarcus with broad, flat bridge (posterior view), arms broad, rounded throughout, with digitiform process extending forward and mesally almost perpendicularly from arm (lateral view); mediuncus long, narrow, straight, with base containing two elongate rods; gonosaccus bilobed, with each lobe bearing single, discrete patch of large gonosetae well separated from opposite lobe, with gonosetae probably facing mesally when unexpanded; gonosetae arising from enlarged setal bases, interior ones slightly smaller than distal ones; hypandrium internum narrow, U-shaped, with hooked comes (Fig. 123d).

**Figure 126.**
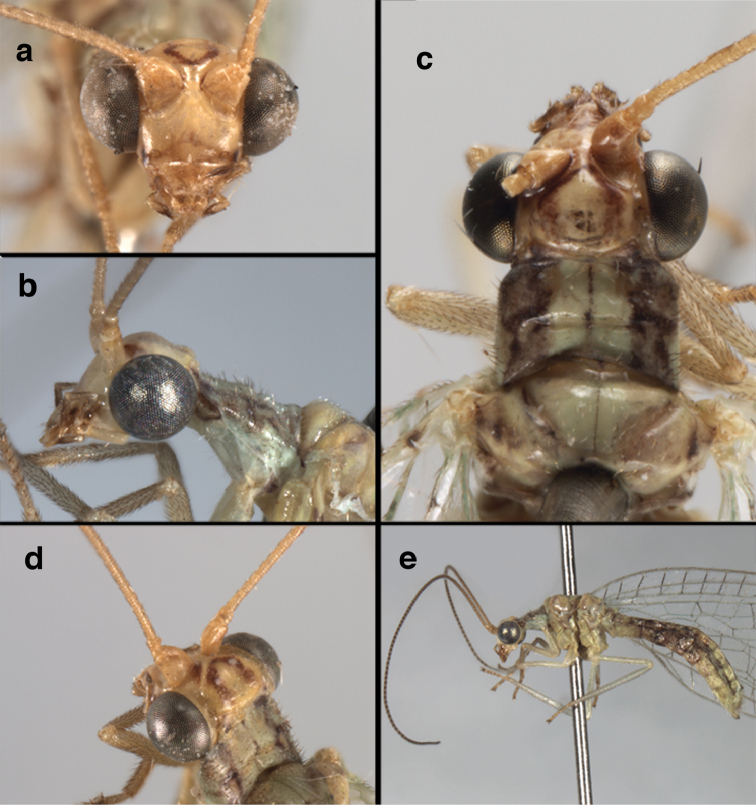
*Ungla confraterna* (Banks): External features, (**a**) head, frontal (**b**) head, prothorax, lateral (**c**) head, prothorax, mesothorax (partial), dorsal (**d**) head, prothorax, dorsolateral, antennal fossa (**e**) body, lateral (Argentina; **a, c, d** Mendoza, female, USNM; **b, e** La Rioja, male, FSCA).

**Figure 127.**
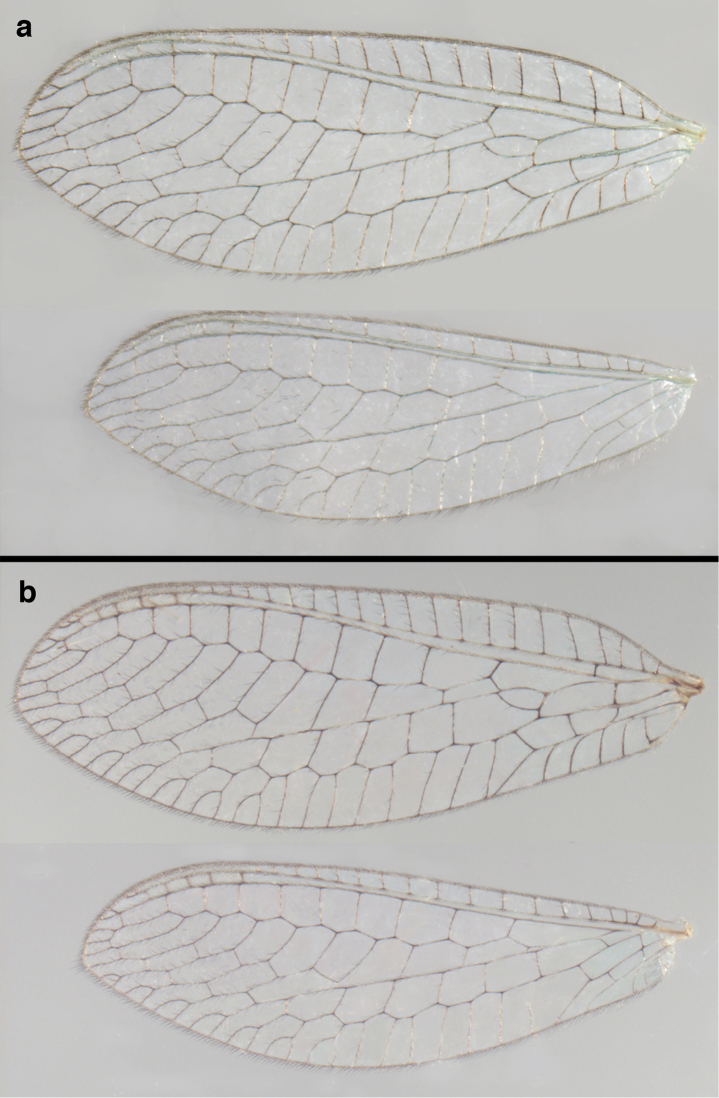
*Ungla confraterna* (Banks): Wings, showing variation, (**a**) longitudinal veins mostly green (**b**) longitudinal veins mostly brown (Argentina; **a** La Rioja, male, USNM; **b** Mendoza, female, CAS).

**Figure 128.**
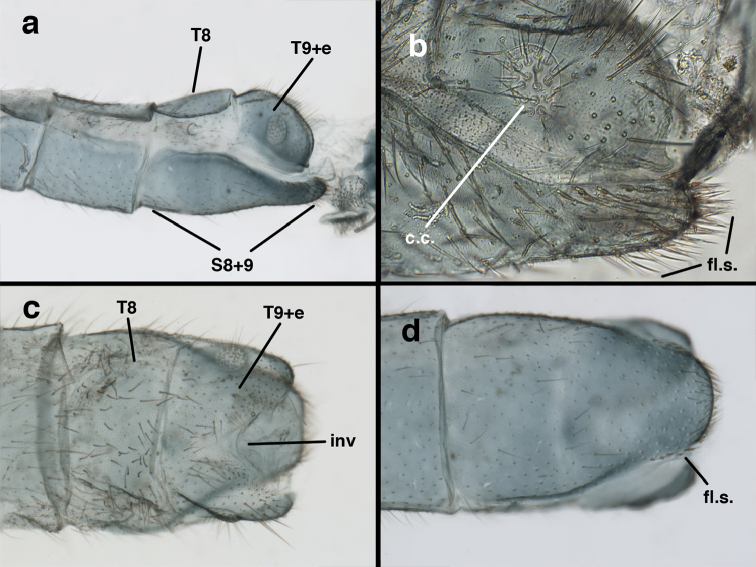
*Ungla confraterna* (Banks): Male abdomen, (**a**) segments A7-terminus, lateral (**b**) tip of terminus, lateral, showing callus cerci and flanged setae on distolateral upper margins of S9 (**c**) T8, T9+ectoproct, dorsal (**d**) fused sternite 8+9, ventral, with large, flanged setae on distolateral margin of S9. **c.c.** callus cerci **fl.s.** flanged setae **inv** invagination of dorsal T9+ectoproct **S8+9** fused eighth and ninth sternites **T8** eighth tergite **T9+e** fused ninth tergite + ectoproct (Argentina; **a, d** Mendoza, USNM; **b, c** Salta, FSCA).

**Figure 129.**
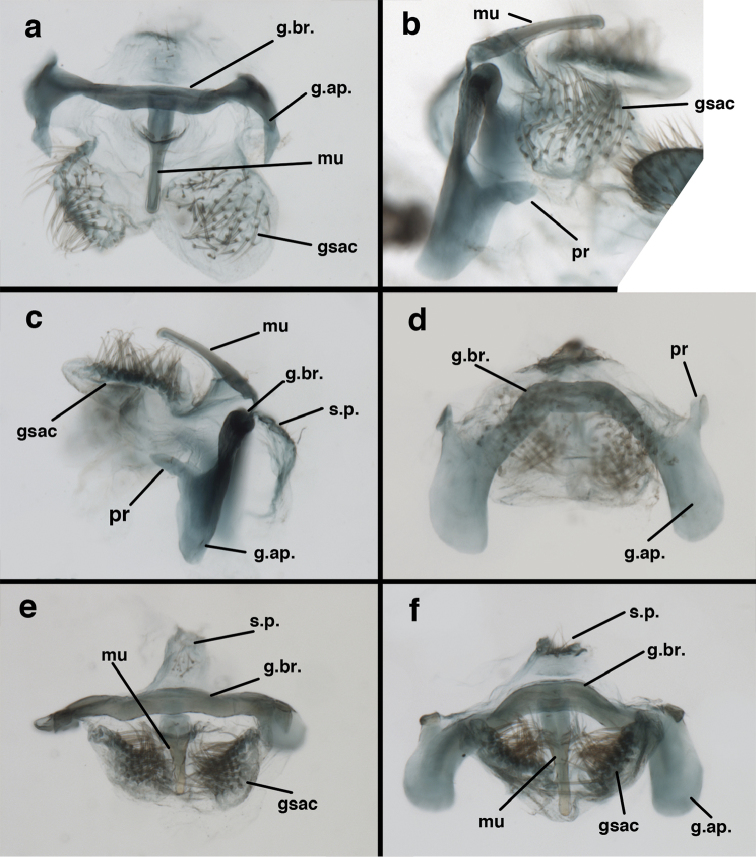
*Ungla confraterna* (Banks): Male genitalia, (**a**) gonarcal complex, dorsal, gonosaccus expanded (**b**) gonarcus, lateral (**c**) gonarcal complex, lateral [Note gonosetae on flat, expanded lobes of gonosaccus.] (**d**) gonarcal complex, posterior (**e**) gonarcal complex, dorsal, gonosaccus withdrawn (**f**) gonarcal complex, frontodorsal, gonosaccus withdrawn. **gsac** gonosaccus **g.ap.** gonarcal apodeme **g.br.** gonarcal bridge **mu** mediuncus **pr** unarticulated process on frontal margin of gonarcal apodeme **s.p.** setose subanal plate (Argentina; **a–c** Mendoza, USNM; **d–f** Salta, FSCA).

##### Variation.

Among the specimens we examined, there was variation in the depth and size of the reddish brown head markings. The body color of most specimens had faded. Also, there was considerable variation in the amount of brown coloration on the venation of the wings – from very little (almost as in *U. chacranella*) to extensive (large sections of the longitudinal veins and all transverse veins brown or brownish).

##### Larvae, biology.

The larvae of *U. confraterna* (as *Ungla binaria*) were described, and some notes on oviposition and development under laboratory conditions are available (Monserrat and Freitas 2005, Reguilón 2010). However, the species identifications of the studied specimens have not been confirmed.

##### Known distribution.

ARGENTINA (north & central western to central eastern): Provinces of Buenos Aires, Catamarca, Córdoba, La Rioja, Mendoza, Rio Negro, Salta, San Juan, Tucumán.

##### Specimens studied

(in addition to types above). Argentina. *Buenos Aires*: Martínez, XII-1956 (1M, CAS). *Catamarca*: Santa Maria, 10-16-II-1972, R. Matthew (1M, 2F, UGCA); Rio Portrero, near Andalgalá, 15-II-1972, W. D. Duckworth (1M, 1F, USNM). *La Rioja*: Mascasin, XI-1961, F. H. Walz (1F, CAS); Santa Cruz, 1600m, 1-XII-2002, L. A. Stange (1M, 2F, FSCA); Patquia, 600 m, XII-1957 (2M, CAS). *Mendoza*: Rio Mendoza, 1600 m, 5-6-XII-1983, L. E. Pena (4M, 2F, CAS); 4 km. SW. Potrerillos, 18-XII-1973, C.M. & O.S. Flint (1M, 1F, 1?, USNM); Unknown locality (1M, IFML). *Salta*: Yacochuya cerca de Cafayate, 15-XII-1973, L. Stange (4M, 7F, FSCA); Alemania, 1-XI-2006, E. González Olazo (1?, IFML). *San Juan*: Iglesia, 4-XII-1983, L. E. Peña (1M, CAS). *Tucumán*: Amaicha del Valle, 29-31-XII-1965, H. & M. Townes (3M, FSCA).

#### Ungla elbergi

Taxon classificationAnimaliaNeuropteraChrysopidae

Tauber sp. n.

http://zoobank.org/06D43FC0-C76F-4C39-8C88-138557819837

**Figure 130.**
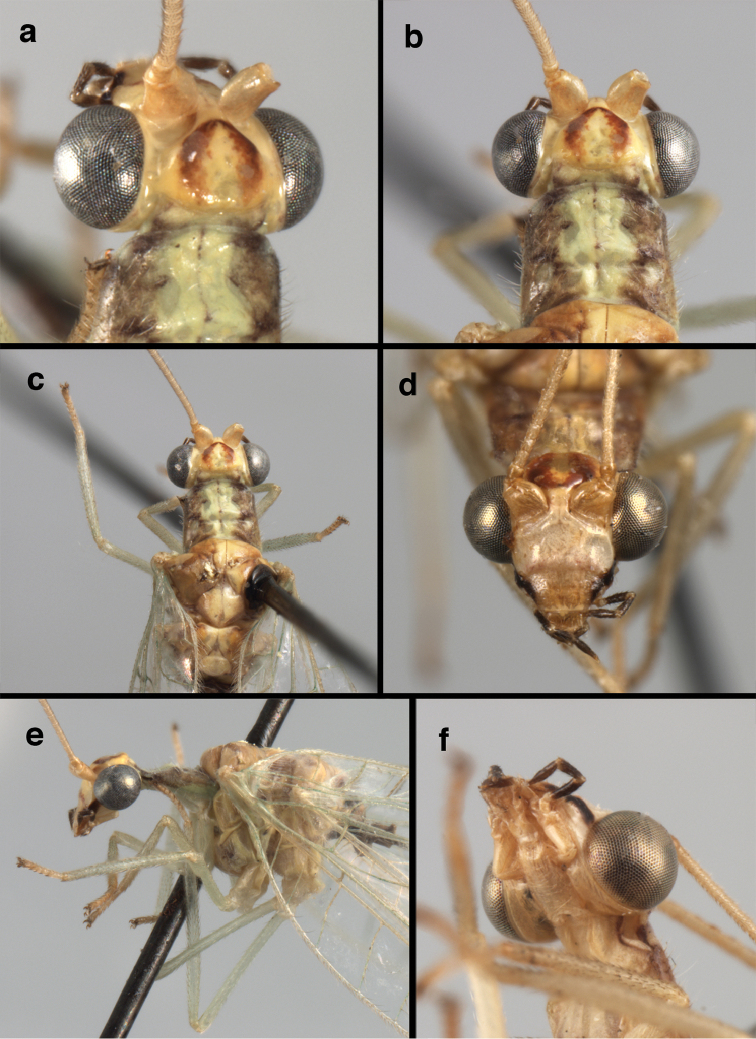
*Ungla elbergi* Tauber, sp. n. External features, (**a**) head, dorsolateral [Note marking in antennal fossa.] (**b**) head, prothorax, dorsal (**c**) head, thorax, dorsal (**d**) head, frontal (**e**) head, thorax, lateral (**f**) head, ventrolateral (Argentina; **a–c, e** La Rioja, paratype, female, EMEC; **d, f** Tucumán, holotype, male, EMEC).

**Figure 131.**
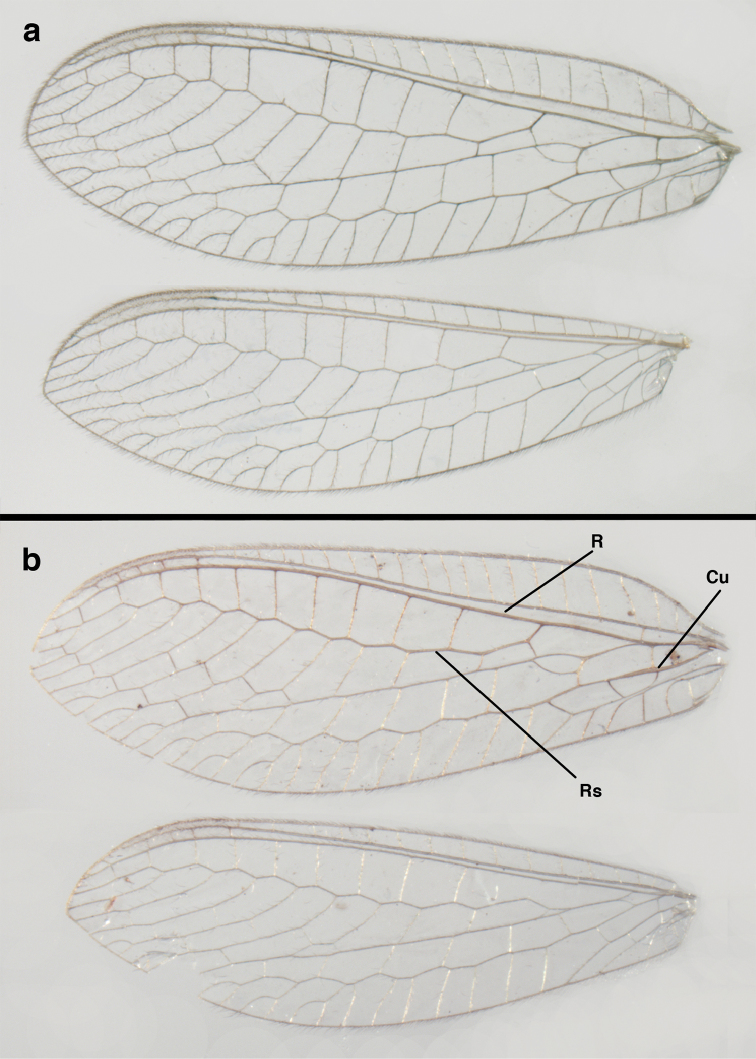
*Ungla elbergi* Tauber, sp. n. Wings, (**a**) female (**b**) male. **Cu** Cubitus **R** Radius **Rs** Radial sector (Argentina; **a** La Rioja, paratype, female, EMEC; **b** Tucumán, holotype, male, EMEC).

**Figure 132.**
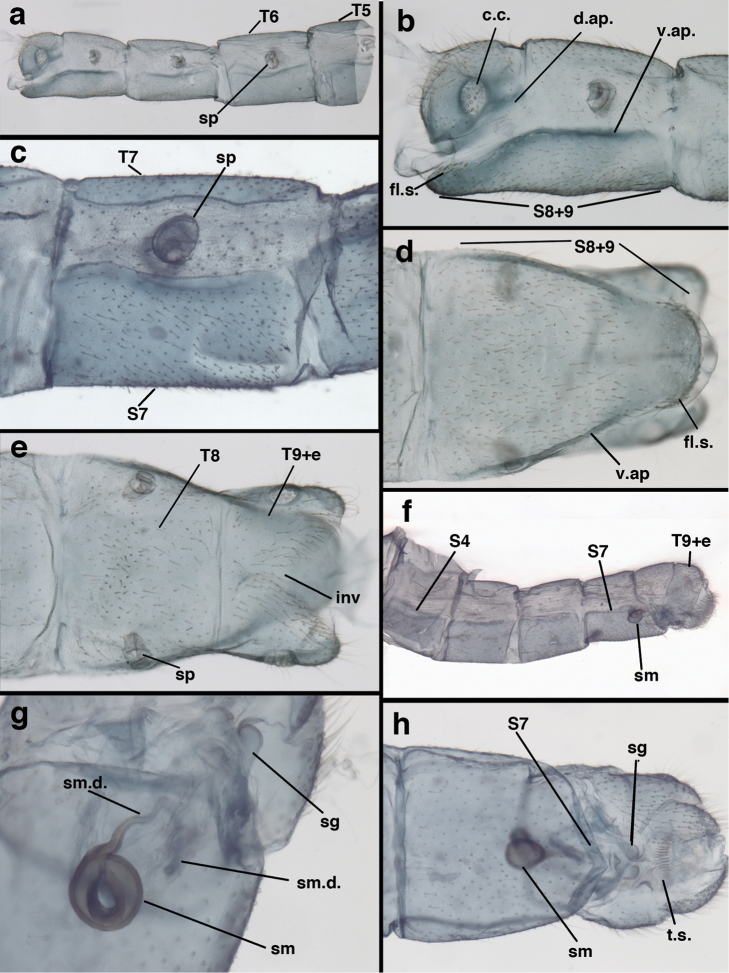
*Ungla elbergi* Tauber, sp. n. Abdomen, (**a-e**) male (**f-h**) female, (**a**) segments A6-terminus, lateral (**b**) terminal segments, lateral (**c**) segment A7 (**d**) fused sternites S8+9 with flanged setae on margins of terminus (**e**) tergites T8 and T9+ectoproct, dorsal (**f**) segments A4-terminus, lateral (**g**) genital structures (**h**) terminus, ventral. **c.c.** callus cerci **d.ap.** apodeme on ventral margin of T9+ectoproct **fl.s.** flanged setae **inv** invagination of dorsal T9+ectoproct **sg** subgenitale **sm** spermatheca **sm.d.** spermathecal duct **sp** spiracle **S4, S7** fourth, seventh sternite **S8+9** fused eighth and ninth sternites **t.s.** transverse sclerite **T5–T8** fifth to eighth tergites **T9+e** fused ninth tergite + ectoproct **v.ap.** apodeme on dorsal margin of S8+9 (Argentina; **a, b, d, e** Tucumán, holotype, EMEC; **c** Tucumán, paratype, FSCA; **f–h** La Rioja, paratype, EMEC).

**Figure 133.**
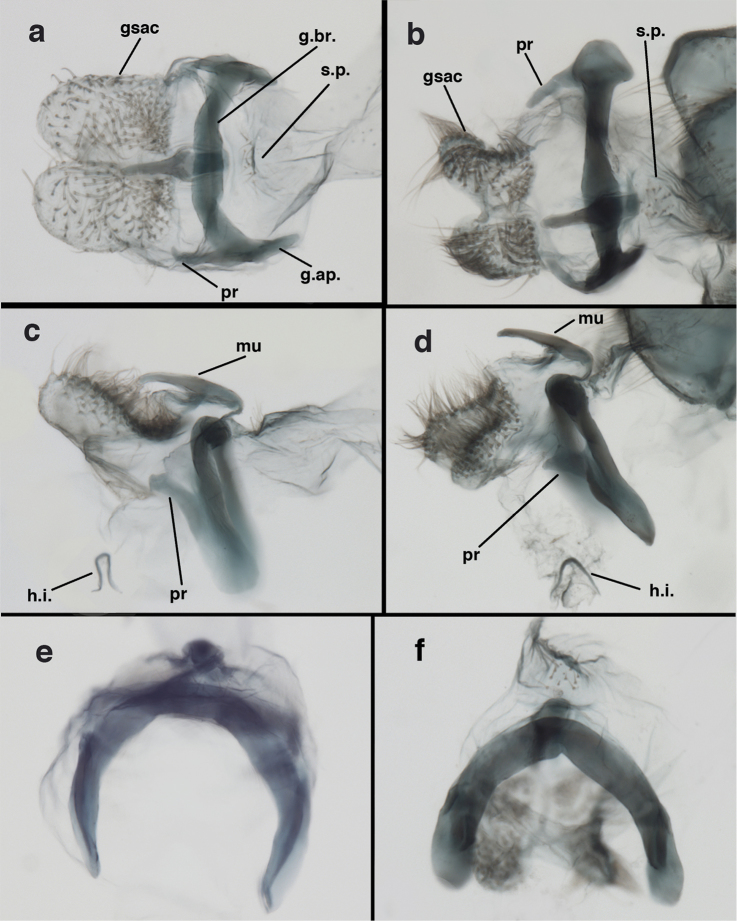
*Ungla elbergi* Tauber, sp. n. Male genitalia, (**a, b**) gonarcal complex, dorsal (**c, d**) gonarcus, lateral (slightly different angles) (**e**) gonarcus, posterior (**f**) gonarcus, posterodorsal. **gsac** gonosaccus **g.ap.** gonarcal apodeme **g.br.** gonarcal bridge **h.i.** hypandrium internum (probably broken in “c”) **mu** mediuncus **pr** unarticulated process on frontal margin of gonarcal apodeme **s.p.** setose subanal plate (Argentina; **a, c, e** Tucumán, holotype, EMEC; **b, d, f** Jujuy, paratype, FSCA).

Figs 130 , 131 , 132 , 133

##### Holotype

(Figs 130d, f, 131b, 132a, b, d, e, 133a, c, e). EMEC, male; “R. A. – Tucumán, Tafi Viejo, Alpa Puyo, 15.08.98, Col. Núñez G.” The specimen bears an ID label – “Ungla binaria. Det. C. Reguilón – G. Olazo”. The right wing is torn and the genitalia are cleared, stained, and preserved in a vial with glycerine on the pin.

##### Etymology.

This species is named in memory of Professor Sanford S. Elberg (1913-2011), University of California, Berkeley. Dean Elberg was Professor of Bacteriology and Public Health and served for 17 years as Dean of the Graduate School. He was a remarkable researcher, teacher, administrator, and friend.

##### Diagnosis.

A prominent black stripe on the gena that extends from the base of the eyes through the entire gena and along the lateral margin of the clypeus of *U. elbergi* is unique among all the small Argentinian species of *Ungla*. In addition, the head markings, largely green longitudinal veins, and reddish brown to brown transverse veins also help to sort *U. elbergi* and *U. confraterna* from other Argentinian species. The most reliable species-specific characters are found in the male. The R and Rs veins of the forewing are heavy in males; the moderately enlarged abdominal spiracles (diameter of A7 spiracle: 0.11 mm, > 0.13× length of segment) are larger than those of *U. confraterna* (diameter ~ 0.08 mm, ~0.1× length of segment) and *U. argentina* (diameter ~0.03 mm, ~0.03× length of segment), and yet considerably smaller than those of *U. chacranella* and *U. annulata* (diameter > 0.15–0.18 mm, > 0.2× length of segment). The shape of the *U. elbergi* gonosaccus and the large number of gonosetae evenly distributed over the entire dorsal surface of the gonosaccus are also unique.

##### Description.

Head cream-colored, with vertex smooth, often shiny; inverted U-shaped marking on vertex reddish brown, prominent, narrowing mesally but contiguous, not extending anteriorly to area between scapes; anteromesal margin of dorsal antennal fossa pale or with reddish brown mark; area between eyes and posterior half of vertex cream-colored, unmarked; frons whitish to cream-colored, sometimes with transverse reddish band along anterior margin; other markings absent; clypeus cream-colored to tan, black laterally; gena with black stripe extending from base of eyes, along lateral margin of clypeus; tentorial pits cream-colored. Antenna with scape cream-colored to light tan, unmarked; pedicel cream-colored to tan, with distal ring of brown; flagellum cream-colored to light tan basally, becoming darker distally; maxillary palp with basal two segments pale, distal three segments dark brown to black, pale at junctions; labial palp with basal two segments pale, distal one dark brown to black.

Prothorax variable in length, green mesally, with wide, brown, lateral stripes, very thin, brown mesal stripe; transverse furrow slightly posterior to middle, reaching lateral margins; with pale, fine setae throughout. Mesothorax, metathorax yellow mesally, reddish brown laterally. Measurements: head width: 1.3 mm; ratio head width : eye width: 2.3–2.5 : 1; prothorax length: 0.6–0.7 mm; prothorax width: 1.0 mm.

Forewing, hindwing clear, without fumose areas, with slender venation, except R, Rs, base of Cu crassate (male only); stigma lightly opaque, light tan, with five tan subcostal crossveins below, area surrounding crossveins unmarked; longitudinal veins mostly green, with brown at intersections; transverse veins, crossveins mostly brown to golden brown. Forewing 12.2–12.8 mm long, 4.2–4.3 mm wide (ratio, L : W = 2.9–3.0 : 1); height of tallest costal cell 0.9 mm (cell number 5–6); length of first intramedian cell 0.8–0.9 mm; 10 radial cells (closed cells between R and Rs); 3–4 Banksian cells (b cells), 4 b’ cells; 3–4 inner gradates, 5–6 outer gradates. Hindwing 11.0–11.6 mm long, 3.5–3.6 mm wide (ratio, L : W = 3.1–3.2 : 1), 10–11 radial cells, 2–4 Banksian (b) cells, 4 b’ cells, 3–4 inner gradates, 5–6 outer gradates.

Male. Abdomen with moderately enlarged spiracles (e.g., A7: spiracle diameter ~0.13× length of sternite); T9+ectoproct short, dome-like, with dorsal invagination shallow, extending approximately one half the distance to anterior margin of T9, lateral margins of invagination straight to slightly convex; dorsal margin rounded distally, with posteroventral margin extended distally in well defined knob; ventral margin well sclerotized along entire length; callus cerci large, ovate, circumference lightly sclerotized, sclerotization contiguous with that on ventral margin of T9+ectoproct. S8+9 fused, with line of fusion not demarcated, but with distal tip well sclerotized (but not as heavily as that on *U. confraterna*); setae on ventral surface short, numerous; dorsal margin heavily sclerotized throughout; terminus slightly extended distally, flat, scoop-like, bearing numerous, heavy, flanged setae along dorsal margins and heavy, unflanged setae distomesally; without the upturned tip of *U. confraterna*. Gonarcus with relatively flat, narrow bridge (dorsal, ventral views), arms extending downward, bowed in broad horseshoe shape; gonarcal arms narrow, rounded distally, with digitiform process extending forward and mesally at an acute angle from arm (lateral, dorsal views); mediuncus medium-length, narrow, convex dorsally, with base containing two elongate, juxtaposed rods; gonosaccus bilobed, with lobes contiguous and gonosetae in contiguous patch basally, lobes juxtaposed and with gonosetae in more discrete, separated patches distally; gonosetae all of somewhat similar size, arising from relatively small setal bases, probably facing mesally when lobes unexpanded, facing upward when lobes inflated; hypandrium internum narrow, broadly U-shaped, with comes faint.

##### Known distribution.

ARGENTINA (northwestern): Provinces of Jujuy, La Rioja, Tucumán.

##### Specimens studied

(in addition to holotype above, all paratypes). Argentina. *Jujuy*: Parque Nacional Calilagua, Mesadas de las Colmenas (1100m), 24/X/2006, E Gonzáles Olazo, G Sziráki (M, IFML). *La Rioja*: Santa Cruz, 1600 m, 1/XII/2002, L. A. Stange (1F, EMEC). *Tucumán*: Tafi Viejo, Alpo Puyo, 15/VIII/1998, Col. C. Reguilón (1M, FSCA), 23/V/1998, Col. Núñez G. (1F, FSCA).

#### Ungla ivancruzi

Taxon classificationAnimaliaNeuropteraChrysopidae

Freitas, 2007

**Figure 134.**
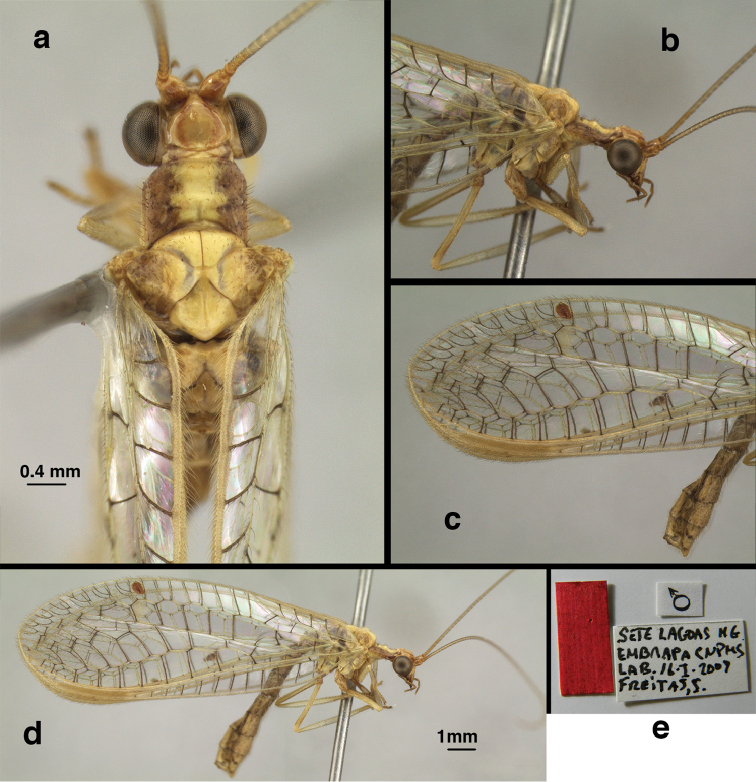
*Ungla ivancruzi* Freitas: External features, (**a**) head, thorax, dorsal (**b**) head, thorax, lateral (**c**) tip of wings and abdomen, lateral (**d**) body, lateral (**e**) labels (Brazil, Minas Gerais, holotype, male, SFC; images FS).

**Figure 135.**
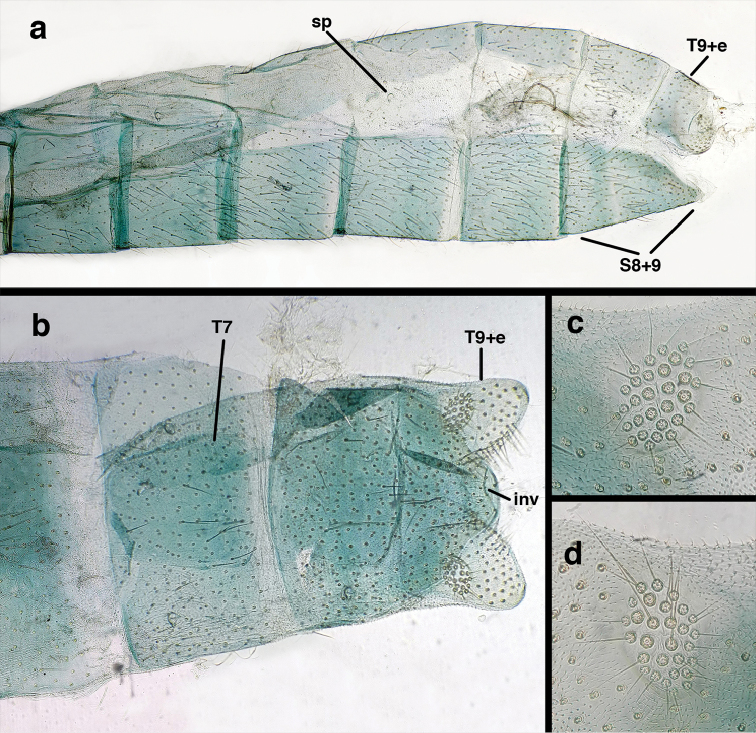
*Ungla ivancruzi* Freitas: Male abdomen, (**a**) segments A3-terminus, lateral (**b**) segments A7-terminus, dorsal [Note shallow invagination.] (**c, d**) callus cerci, showing variation in shape. **inv** invagination in T9+ectoproct **sp** spiracle **S8+9** fused eighth and ninth sternites **T7** seventh tergite **T9+e** fused ninth tergite + ectoproct (Brazil, Minas Gerais, from original type series or their offspring, MJMO; images FS).

**Figure 136.**
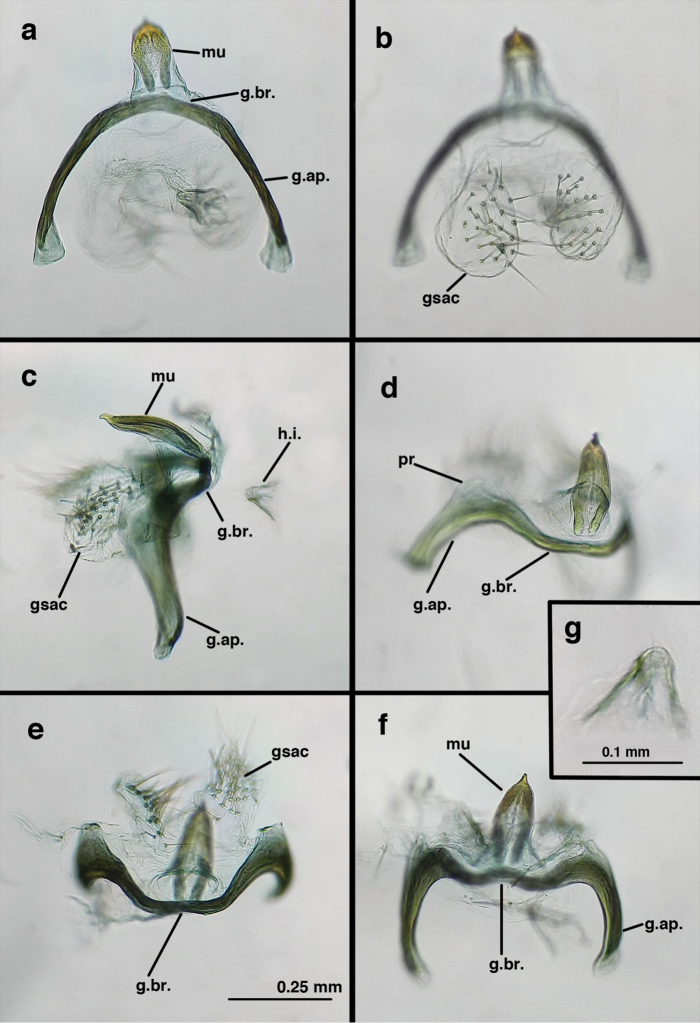
*Ungla ivancruzi* Freitas: Male genitalia, (**a**) gonarcus and mediuncal base, posterior (**b**) setose gonosaccus, expanded, frontal (**c**) gonarcus with hypandrium internum, lateral [Note relative size of hypandrium internum.] (**d**) mediuncus, gonarcus, dorsal to dorsolateral (**e**) gonarcus, frontodorsal (**f**) gonarcus, posterodorsal (**g**) hypandrium internum. **gsac** gonosaccus **g.ap.** gonarcal apodeme **g.br.** gonarcal bridge **h.i.** hypandrium internum **mu** mediuncus **pr** unarticulated process on frontal margin of gonarcal apodeme (Brazil, Minas Gerais, from original type series or their offspring, MJMO; images FS).

Figs 134 , 135 , 136

Ungla ivancruzi Freitas, 2007. *Rev. bras. Entomol.* 51: 414–415; “Minas Gerais, Sete Lagoas, EMBRAPA, CNPMS, Lab., 16.I.2007 (S. de Freitas)”. Tauber et al. 2014: supplementary material (list); Oswald 2015 (catalog). **Holotype.**MZSP (?), male (examined, FS); holotype by original designation. The original description stated that the holotype was in the MZSP, but it has not been found there; it is at the Departamento de Fitossanidade, UNESP, Jaboticabal, São Paulo. The holotype and all the paratypes appear to have been reared in the laboratory; neither the conditions under which the adults were held, nor the age of the adults when they were preserved for study were mentioned.

##### Diagnosis.

Among *Ungla* species *U. ivancruzi* is unique in that the male has a broadly wedge-shaped S8+9 that is more typical of *Chrysopodes* than other *Ungla* males, which have S8+9 elongate and tapering. Nevertheless, we consider *U. ivancruzi* a good species of *Ungla*; it expresses most of the diagnostic features of the genus, including the distinctive inverted U-shaped mark on the vertex, dorsal apodeme on the male T9+ect reduced so that it does not extend beneath T8, gonarcal arch rounded, with a narrow, elongate mediuncus attached dorsally, and a bilobed gonosaccus, with each lobe bearing a patch of elongate gonosetae. *Ungla ivancruzi* can be distinguished from other *Ungla* by its (i) light colored (not black or dark brown) palpi, (ii) reddish genal markings, (iii) narrow gonarcal arch with thin, elongate arms lacking a prominent frontal process, and (iv) gonosaccus with gonosetae relatively well-spaced, not abutting each other. At this time, *U. ivancruzi* is the only *Ungla* species known from Brazil. See original description by Freitas (2007) for additional images.

##### Redescription.

Head with vertex yellow, raised, smooth, shiny; inverted U-shaped mark red to reddish brown, broken anteromesally, not extending forward between antennae. Frons yellow, without marks; gena light reddish brown; maxillary palp pale, ultimate palpomere 1.5 times longer than anterior one; scape yellow with dorsomesal red mark that extends onto pedicel, base of flagellum; flagellomeres yellowish brown, with rows of black setae.

Thorax yellowish green, with distinct longitudinal cream-colored stripe mesally. Prothorax with pair of broad, dark reddish brown stripes laterally; transverse furrow in posterior region of segment, extending to lateral margins, with brown to black setae on areas marked with brown, golden setae on green areas. Mesonotum, metanotum with diffuse brown lateral marks. Measurements: head width: 1.3 mm; ratio head width : eye width: 2.3 : 1; prothorax width: 1.0 mm, length: 0.6 mm.

Forewing, hindwing with rounded tip; membrane clear, hyaline, without fumose areas; stigma slightly opaque to light brown. Forewing with longitudinal veins green, marked with brown at intersections; transverse veins dark brown to black; transverse costal, radial, gradate veins black, without suffusion. Forewing with Rs sinuous; first intramedian cell ovate; basal inner gradate meeting Psm; 7.9–10.2 mm long, 3.4 mm wide (ratio, L : W = 2.9 : 1); height of tallest costal cell 0.63 mm (cell number 5); width of first intramedian cell 0.65 mm; 9 radial cells (closed cells between R and Rs); third gradate cell 0.9 mm long, 0.4 mm wide (ratio, L : W = 2.3 : 1); fourth gradate 0.4 mm long, 0.3 mm wide (ratio, L : W = 1.5 : 1); 4 Banksian cells (b cells), 4 b’ cells; 3 inner gradates, 5 outer gradates. Hindwing 7.2–9.2 mm long, 3.0 mm wide (ratio, L : W = 3.0 : 1), 9-11 radial cells, 3 Banksian (b) cells, 4 b’ cells, 3 inner gradates, 5 outer gradates.

Abdomen yellowish green, without spots. Male. T9+ect relatively long (~0.5 length of T7), almost straight dorsally, angulate at dorsal terminus, with dorsal invagination deep (~0.7× dorsal length of T9+ect); margins, base of invagination rounded; abdominal spiracles small (e.g., A7: spiracle diameter ~0.09× length of sternite); callus cerci large, round to oval, with 27–30 trichobotria; S8+9 fused, with line of fusion perceptible; dorsum tapering slightly throughout basal 3/4ths, abruptly through distal fourth; S8+9 length ~1.8x height, not much longer than S7 (1.4×); apex acute, not upturned, not elongated beyond T9+ect. Gonarcus thin, arched without angular bend (dorsal, frontal view), with S-shaped curve (lateral view); gonarcal bridge, arms slender throughout, thickened mesally, forming very small mesal process; arcessus long, curved dorsally, with pair of lateral rods internally, slightly splayed mesally, fusing distally, terminating in small beak; gonossacus bilobed, with two tufts of elongate, well-spaced gonosetae facing each other mesally; hypandrium internum V-shaped, small.

Female. See Freitas (2007).

##### Larvae and biology.

Immatures were reared, and the three instars are described (Freitas 2007). The larvae are debris carriers; and eggs are laid in clusters.

##### Known distribution.

BRAZIL: State of Minas Gerais.

##### Specimens examined

. Same data as type (1M, 1F, offspring from original collection of type series, MJMO).

#### Ungla steinbachi

Taxon classificationAnimaliaNeuropteraChrysopidae

(Navás, 1925) comb. n.

**Figure 137.**
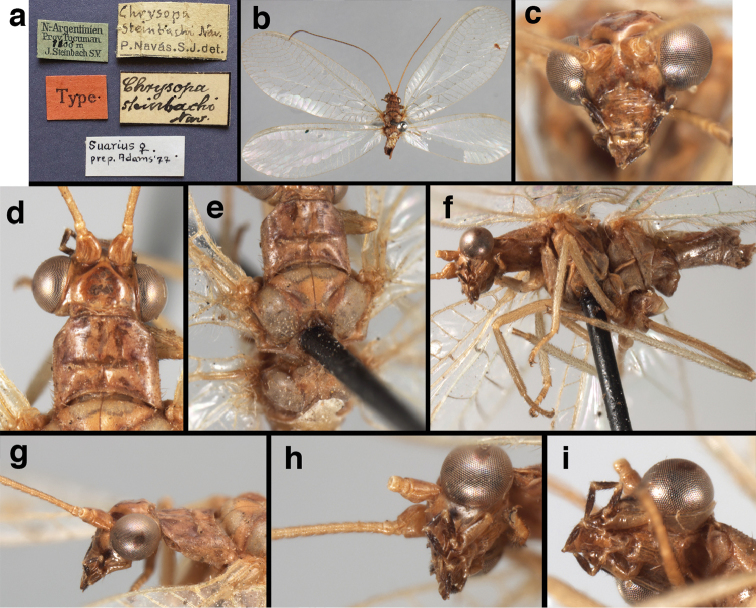
*Chrysopa steinbachi* Navás: External features, (**a**) labels (**b**) habitus, dorsal (**c**) head, frontal (**d**) head, prothorax, dorsal (**e**) thorax, dorsal (**f**) head, thorax, ventrolateral (**g**) head, prothorax, lateral (**h**) head, ventrolateral (**i**) head, ventral (Argentina, Tucumán, holotype, female, ZMB).

**Figure 138.**
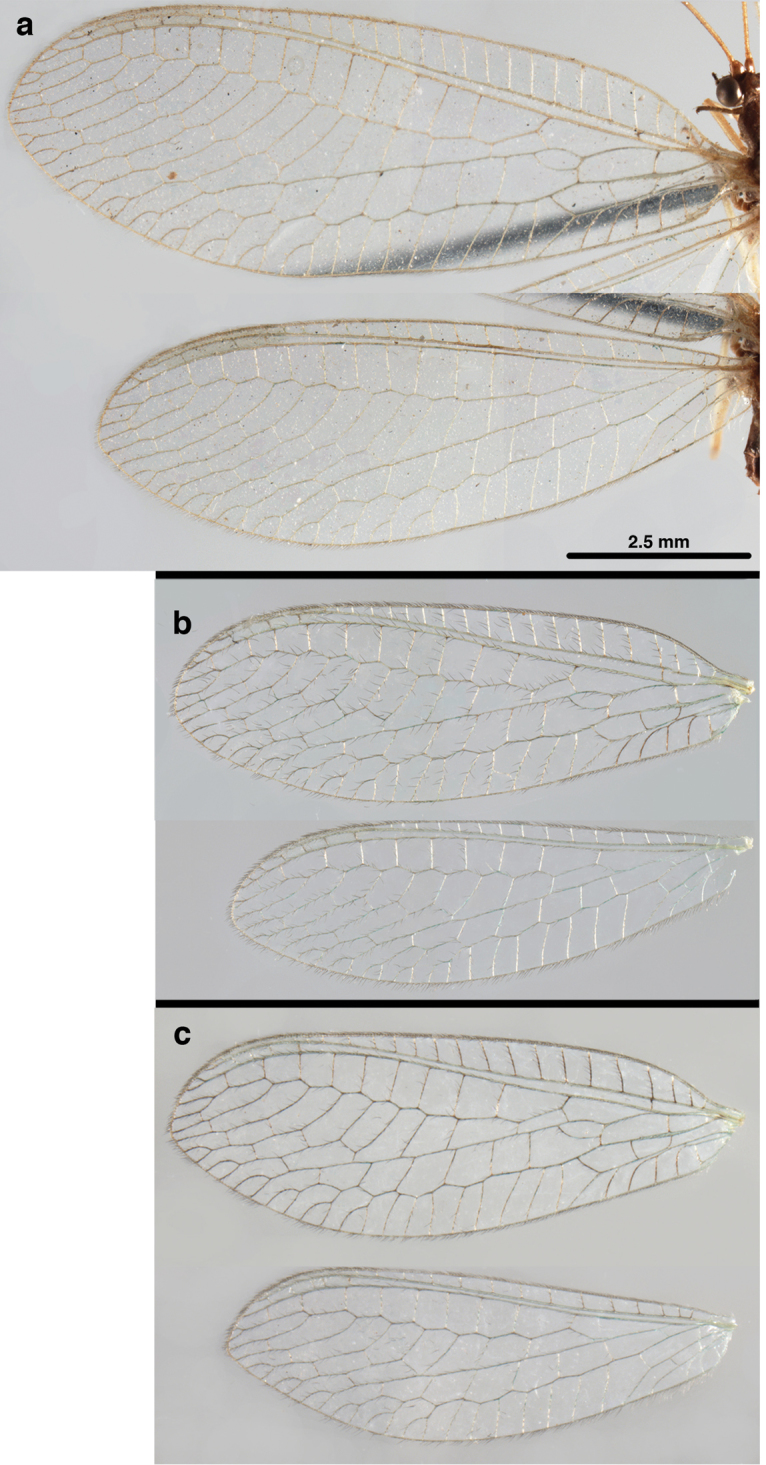
Wings of three *Ungla* species from Argentina (all: same scale), (**a**) *Ungla steinbachi* (Navás) (**b**) *Ungla chacranella* (Banks) (**c**) *Ungla confraterna* (Banks). (Argentina; **a** Tucumán, lectotype of *Chrysopa steinbachi*, female, ZMB; **b** La Rioja, male, FSCA; **c** La Rioja, male, USNM).

**Figure 139.**
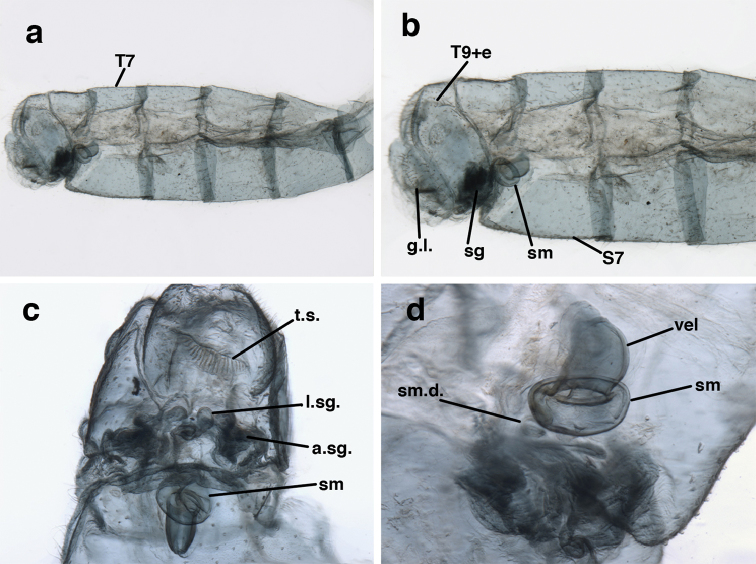
*Chrysopa steinbachi* Navás. Female abdomen and genitalia, (**a**) segments A4-terminus, lateral (**b**) terminalia, lateral (**c**) terminalia, ventral (**d**) spermathecal complex, lateral. **a.sg.** lateral arm of subgenitale **g.l.** gonapophysis lateralis **l.sg.** lobe of subgenitale **sg** subgenitale **sm** spermatheca **sm.d.** spermathecal duct **S7** seventh sternite **t.s.** transverse sclerite **T7** seventh tergite **T9+e** ninth tergite and ectoproct **vel** velum (Argentina, Tucumán, holotype, ZMB).

Figs 137 , 138 , 139

Chrysopa steinbachi Navás, 1925. *Ann. Soc. Sci. Bruxelles*: 567–568. “ ‘N. Argentinien, Prov. Tucuman, 1100 m., I. Steinbach S. V.’ Mus. Zool. de Berlin”. Stange 1967: 37 (catalog); Penny 1977: 21 (list); Brooks and Barnard 1990: 280 (list, as “ ‘Chrysopa’ incertae sedis”); Oswald 2015 (catalog). **Lectotype** (Figs 137, 138a, 139). ZMB, female (examined). Specimen in good condition, but discolored with age; abdomen in vial with glycerin. Stange (1967) listed it as a holotype; however Navás did not mention how many specimens he had; thus the specimen in the ZMB is here designated as the lectotype (des. CAT). A red lectotype label has been applied to the specimen. **Support for generic placement.** Currently, this species is known only from the lectotype, which is a female. Here, we base its generic placement on several features that characterize Ungla species: vertex with U-shaped dorsal marks; dark markings on the terminal segments of the maxillary and labial palpi, and a basal inner gradate that does not meet the Psm. The female genitalia are also typical of Ungla species.

##### Diagnosis.

*Ungla steinbachi* is a pale-bodied Argentinian species known with certainty only from the type. It closely resembles *U. chacranella* in the following external features: vertex with small reddish mark, dark brown to black genal marks; antenna, including pedicel, pale, without dark ring; wings with rounded to subacute tips and with veins, including the crossveins and gradates, light green (an occasional anal vein may appear light brown to brown). However, three features lead us to recognize the two species as distinct from each other. First, the difference in size between the *C. steinbachi* type and the *U. chacranella* specimens that we studied is substantial; the forewings and hindwings of the *C. steinbachi* type are each more than 10% greater in length and width than those of the largest *U. chacranella* specimen we measured (For comparison, see Fig. 138). Second, the number of transverse veins is greater in the *C. steinbachi* type than in the *U. chacranella* specimens: radial cells in both the forewing and hindwing (13 versus a maximum of 12 in *U. chacranella*), inner and outer gradate veins (one more each than the maximum found in *U. chacranella*). Third, on the *C. steinbachi* type, the cells on either side of the inner gradate veins are longer and narrower than on the *U. chacranella* specimens.

We have seen only one other specimen, a somewhat discolored female from Jujuy Province that resembles the type of *U. steinbachi* in size and probable coloration of veins; however its pattern of venation differs considerably, and we cannot identify it with confidence. Given the small number of specimens (the type female) underlying our retention of *U. steinbachi* as a valid species separate from *U. chacranella*, we hope that additional pale-bodied Argentinian *Ungla* species will be collected and studied – especially in regard to the range and pattern of morphological variation.

##### Redescription.

Head: vertex smooth, slightly depressed mesally; inverted U-shaped marking on vertex reddish, large, probably joined mesally, not extending between scapes; anteromesal margin of dorsal antennal fossa dark reddish brown; area between eyes and posterior half of vertex probably reddish; frons cream-colored with broad reddish brown coloration along anterior margin; clypeus cream-colored to tan, reddish brown laterally; gena, posterolateral margin of the clypeus with dark brown stripe; tentorial pits with reddish brown marking. Antenna with scape cream-colored to light tan, perhaps with some reddish coloration dorsally; pedicel cream-colored to tan, without distal ring; flagellum cream-colored basally, becoming darker distally; maxillary palp with basal two segments pale, distal segments brown, articulations pale; labial palp with basal segment pale, distal two segments brown.

Prothorax wide, probably with pair of broad, brown, lateral stripes, thin, brown mesal stripe; transverse furrow in mesal region, not reaching lateral margins; with short to long, mostly pale setae throughout. Mesothorax, metathorax greenish with yellow mesally. Measurements: head width, 1.4 mm; ratio head width : eye width, 2.5 : 1; prothoracic length, 0.8 mm; prothoracic width, 1.1 mm.

Forewing, hindwing clear, hyaline, without fumose areas, with slender venation; stigma lightly opaque, with five brown to dark brown subcostal crossveins below stigma; most veins of forewing cream-colored to light green, basal subcostal crossvein, base of Rs, base of M-Cu crossvein, branches of anal veins darkened, intersections of most veins, crossveins marked with small brown spot; veins of hindwing paler than those on forewing, mostly without brownish marks at intersections. Forewing 14.8 mm long, 5.4 mm wide (ratio, L : W = 2.8 : 1); height of tallest costal cell 1.0 mm (cell number 6); width of first intramedian cell 1.0 mm; 13 radial cells (closed cells between R and Rs); third gradate cell 1.80 mm long, 0.44 mm wide (ratio, L : W = 4.06); fourth gradate cell 1.74 mm long, 0.40 mm wide (ratio, L : W = 4.39); 5 Banksian cells (b cells), 5 b’ cells; 7 inner gradates, 9 outer gradates. Hindwing 13.4 mm long, 4.7 mm wide (ratio, L : W = 2.9 : 1), 13 radial cells, 4 Banksian (b) cells, 5 b’ cells, 7 inner gradates, 8 outer gradates.

Male. Unknown.

Female. See Fig. 139.

##### Variation.

The female specimen from Jujuy, Argentina, is similar in size to *U. steinbachi* (forewing 1.6 mm long, 5.2 mm wide), and it shares many features with this species. However, its forewing has fewer radial cells (n =11) and fewer gradates (inner = 4, outer = 7). Adams’ handprinted note on the specimen indicated that he considered it to be a species near “binaria” (now a synonym of *U. confraterna*); we are uncertain.

##### Known distribution.

ARGENTINA: Provinces of Tucumán and possibly Jujuy.

##### Specimens examined

(in addition to the type): Argentina. Jujuy, no locality, 20/II/1955, Bought F. H. Walz, Phillip A. Adams Collection 1998 bequest to Calif. Acad. Sci. (F, CAS, identification tentative).

### *Species inquirendae*

The following species, at one time or another, were considered to be synonyms of *U. argentina*. However, we were unable to confirm the synonymies, and the species remain in need of further study.

***Chrysopa venulosa* Navás, 1918**. *Serie 2 Physis, Rev. Soc. Arg. Cienc. nat.* 4: 87; “Andalgalá (Catamarca), 1896, BRUCH (Mus. de La Plata)”. Stange 1967: 38 (type); Adams 1975: 171 (probable synonymy with *Suarius argentinus*); Penny 1977: 21 (list, as *Chrysopa*); Brooks and Barnard 1990: 280 [list, as “ ‘*Chrysopa*’ incertae sedis, (? *Ungla*)”]; González Olazo 1996: 381 (synonymy with *U. argentina*); Oswald 2015 (catalog).

**Figure 140.**
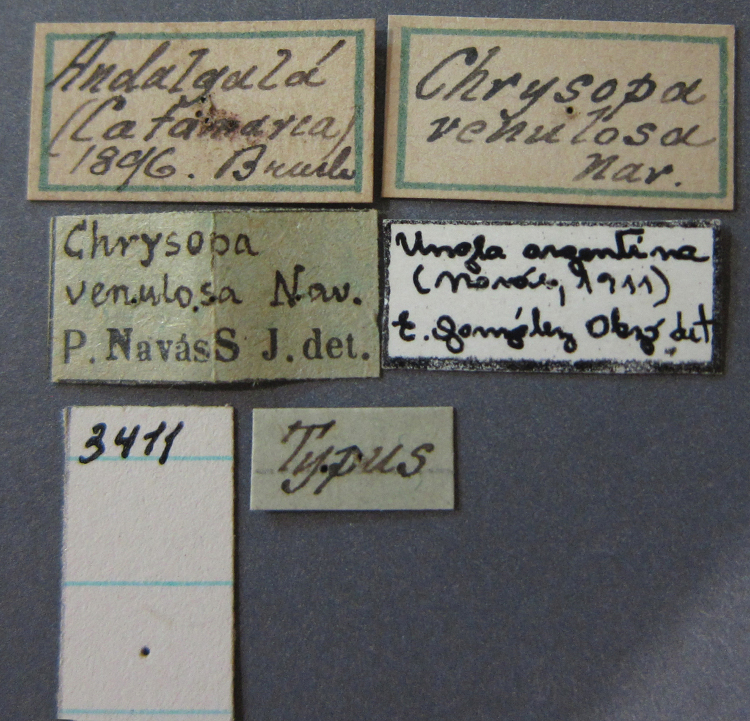
*Chrysopa venulosa* Navás: Labels from lectotype (Argentina, Catamarca, MLPA).

**Lectotype** (Fig. 140). MLPA, abdomen missing (examined briefly). Both Stange (1967: 36) and González Olazo (1996: 381) reported seeing the type in the MLPA; Stange (1967) and Adams' unpublished notes reported the abdomen missing; however, González Olazo (1996) did not. During our visit to the MLPA, we examined a specimen that appeared to be the type; the abdomen indeed is missing. It bears locality data as reported in the original description, an identification label in Navás’ hand, three labels in a hand other than Navás’, a number “3411”, and an identification label (*Ungla argentina*) by González Olazo.

**Status of species.** González Olazo (1996: 381) presented no supporting evidence for his synonymy, nor was it likely that he was aware of the cryptic species that resemble *U. argentina*. In his description, Navás commented that the venation on his specimen was very dark and that the wings were hyaline – both features that typify *U. annulata*. Also, from Adams’ notes, it appears that he considered the species to be similar to *C. annulata* (and *C. nervulosa*). We tend to agree with Adams, but without additional study, we cannot make a confident identification.

***Chrysopa graciana* Navás, 1919**. *Rev. R. Acad. Cienc. exactas fis. nat. Madrid* (1919a) 17: 301; “República Argentina: Alta Gracia, febrero de 1918. C. Bruch (Museo de La Plata)”. Navás 1919b: 190 [1923] (list); Navás 1927: 21 (list); Navás 1929: 222 (list); Stange 1967: 33 (catalog); Adams 1975: 169 (provisional synonymy with *Suarius argentinus*); Penny 1977: 16 (list, as synonym of *C. argentina*); Adams and Penny 1985: 421 [synonymy with *Chrysoperla externa* (Hagen, 1861)]; Brooks 1994: 168 (list); González Olazo 1996: 379 (catalog, synonymy with *U. argentina*); Monserrat and Freitas 2005: 175 [synonymy with *Chrysoperla externa externa* (Hagen)]; Oswald 2015 [catalog, as *C. externa externa*].

**Figure 141.**
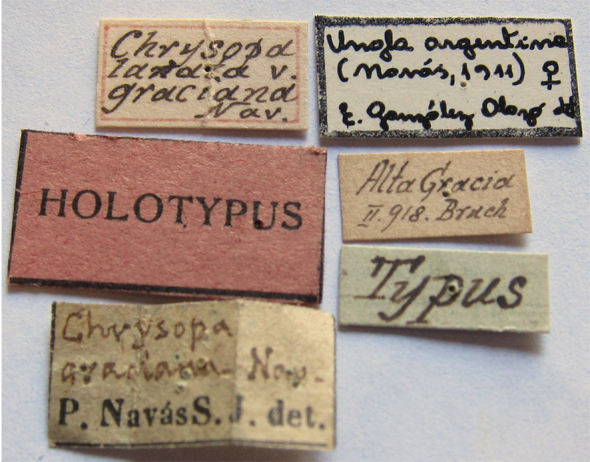
*Chrysopa graciana* Navás: Labels from type (Argentina, Córdoba, MACN).

**Type.** (Fig. 141 – labels only). MACN?, sex unknown (examined very briefly). Navás, in the original description, stated that the type was in the MLPA. Subsequently, Adams (1975: 169) reported seeing a type (male) – “in the Museum de la Plata, Buenos Aires”. [The MLPA is not in Buenos Aires, so this notation is unclear. Adams’ notes also showed some uncertainty – the notation “M(?)” – regarding the sex of the type.]. Stange (1967: 33) and González Olazo (1996: 379) reported a female type in the MACN; they considered this specimen to be the holotype. Presumably the type (sex unspecified) that Adams and Penny (1985: 421) reported in “the Buenos Aires Museum” refers to this specimen as well. We saw this type in the MACN (sex undetermined); Dra. Analia Lanteri assured us that there is no type in the MLPA. Thus, unless a type is discovered at the MLPA, the specimen (presumably, a female) in the MACN probably is the lectotype. We did not study this specimen closely, and we are unsure of its identification.

**Figure 142.**
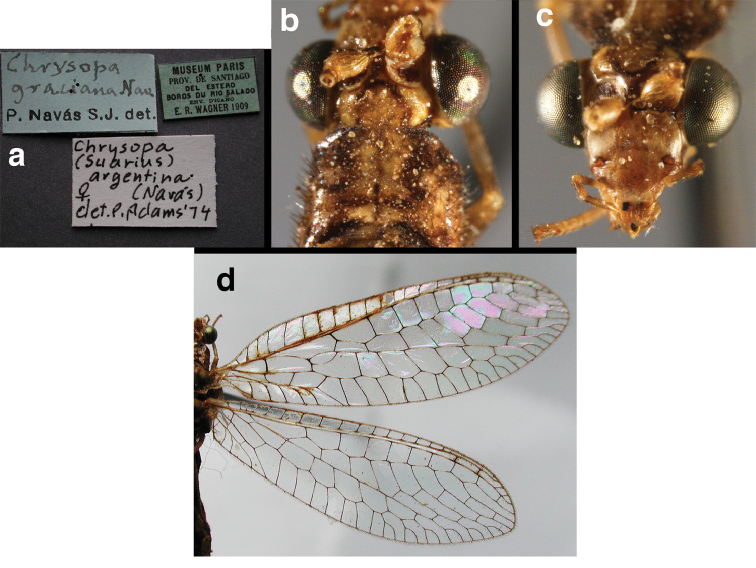
*Chrysopa graciana* Navás: Features of nontype specimen reported by Navás 1927: 21, **a** labels **b** head, dorsum **c** head, frontal **d** wings (Argentina, Santiago del Estero, female, MNHN).

**Status of species.** Based on two female non-type specimens in the MNHN that were determined and reported by Navás (1927: 21) (see Fig. 142a–d here), and a cursory examination of a type in Buenos Aires, Adams (1975: 169) provisionally synonymized *C. graciana* with *Suarius argentinus* [now, *Ungla argentina*] (also see Penny 1977: 16). Subsequently, Adams and Penny (1985: 421) synonymized *C. graciana* with *Chrysoperla externa* (Hagen, 1861) without comment (also see Brooks and Barnard 1990: 271, Brooks 1994: 168, Oswald 2015). Later, González Olazo (1996: 379) re-synonymized the species with *U. argentina*, without comment (also see Legrand et al. 2008: 138). Finally, Monserrat and Freitas (2005) resynonymized *C. graciana* with *Chrysoperla externa externa* (Hagen, 1861), again without comment.

Based on name changes listed above, we conclude that a lectotype should be designated and the taxonomic status of the lectotype should be substantiated with supporting evidence.

***Chrysopa nervulosa* Navás, 1924**. *Rev. Chil. Hist. nat.* 27: 115 (“1923”); “República Argentina: Alta Gracia (Córdoba,) 17 de novembre de 1921. Bruch (col. Bruch)”. Objective replacement name of ***Chrysopa venulosa* Navás, 1923.** (“1919”b: 192), junior objective homonym of *Chrysopa venulosa* Navás, 1918 (above). Stange 1967: 36 (catalog); Adams 1975: 171 (provisional synonymy with *Suarius argentinus*); Penny 1977: 19 (list); Brooks and Barnard 1990: 280 (listed as *C. venulosa*, under ‘*Chrysopa*’ incertae sedis); González Olazo 1996: 380 (catalog, synonymy with *U. argentina*); Oswald 2015 (catalog).

**Lectotype.** Female, MACN (not examined). Stange (1967: 36) reported a type (female) from the MACN, and González Olazo (1996: 380) reported that the abdomen was missing. Adams (1975: 171) indicated that he had not seen the type.

**Status of species.** González Olazo (1996: 380) synonymized *C. nervulosa* with *U. argentina* without comment. However, this action was taken before the cryptic species resembling *U. argentina* were recognized. Navás’ original description indicates that the wings are hyaline – a feature that is consistent with *U. annulata*. We have not examined the type.

***Chrysopa coronata* Navás, 1930**. *Rev. Chil. Hist. Nat.* 34: 64-65; “República Argentina. Buenos Aires, Villa Devoto, Diciembre de 1925. P. Mühor, S. J. leg.”. Stange 1967: 32 (catalog); Penny 1977: 17 (list); Brooks and Barnard 1990: 279 (list, as “ ‘*Chrysopa*’ incertae sedis”); González Olazo 1996: 379 (catalog, synonymy with *U. argentina*). Junior homonym of Chrysopa prasina var. coronata Navás, 1915 (See Oswald 2015).

**Type.** MACN (IIES), sex unknown. Stange (1967: 32) reported a “holotype” (with abdomen missing) in “San Miguel” (= “Observatorio de Física Cósmica, San Miguel, ARGENTINA”); González Olazo (1996: 379) reported the type (with abdomen missing) at INESALT. Later, it was found in the Museo de Ciencias Naturales, Salta, Argentina (von Ellenrieder 2009: 259), but now is in the MACN (von Ellenrieder, personal comunication). It is listed as destroyed by Oswald (2015). Its abdomen is missing; we have not seen the specimen.

**Status of name.** González Olazo (1996: 379) synonymized the name with *U. argentina*. Given the variation now known to surround *U. argentina*, this action is unconfirmed.

### *Species Incertae Sedis* (probably *Ungla*)

We have not seen types or identified specimens of the following two species. The first one was assigned to *Ungla* without comment. The second one is suspected of being in *Ungla*, but has not been moved there. They both are from Argentina.

***Chrysopa reboredina* Navás, 1933**. *Rev. Acad. Cienc exactas fis-quim. nat. Zaragoza* (1933b): 93–94. Stange 1967: 36 (catalog); Penny 1977: 20 (list); Brooks and Barnard 1990: 280 (list, as “ ‘*Chrysopa*’ incertae sedis”); González Olazo 1996: 380 (catalog, as *Ungla* sp.); Oswald 2015 (catalog).

**Type.** MACN (IIES), sex unknown. Stange (1967: 36) reported the type was deposited at “San Miguel”; González Olazo (1996: 380) reported that it was at “INESALT” and that the abdomen and one hindwing were absent. Later, it was found in the Museo de Ciencias Naturales, Salta, Argentina (IIES) (von Ellenrieder 2009: 259), but now is in the MACN (von Ellenrieder, personal comunication). Apparently it is in a vial; its abdomen and one wing are missing, but the other three wings are present. We have not seen it.

**Status of species.** Probably a species of *Ungla*, in need of verification. In our opinion, the illustration and description by Navás are consistent with an *Ungla* species, and Adams’ notes indicate that he considered the species to be associated with other species that later were included within *Ungla*. Apparently, he did not see the type.

***Chrysopa dichroa* Navás, 1923**. *Arxius Inst. Ciènc., Inst. Est. Catalans, Sec. Ciènc.* (“1919”b) 7: 191–192. “República Argentina: Alta Gracia (Córdoba), 26 de desembre de 1921. Bruch (Col. m.)”. Stange 1967: 32 (catalog); Penny 1977: 17 (list); Brooks and Barnard 1990: 279 (list, as “ ‘*Chrysopa*’ incertae sedis”); Oswald 2015 (catalog).

**Type.** Apparently destroyed. According to the original description, the type was deposited in the Navás collection (MZBS), but it is not there now (Monserrat 1985: 237). Adams’ notes state that he saw a series of eight females and two males that were collected from La Paz (Dep. San Javier) Córdoba between January 15 and 31, 1928. These specimens are unlikely to be from the same series as the type. Adams also mentioned a male specimen that was collected from the type locality about a month later than the type; it carries a Navás determination label. At the time of our visit to the MACN, we were unaware of the specimens, and we did not see them in the collection.

**Status of species.** Perhaps a valid Argentinian species of *Ungla*, in need of a lectotype or neotype designation. Adams reported that the sternites on the MACN specimens were similar to those of *C. lambda*, but that the males had enlarged spiracles. Other features noted by Adams include unmodified setae on S9, venation generally pale with few, rather dark crossveins, pronotal marks black, pterothorax with brown marks, abdomen black with green mottling.

**Figure 143.**
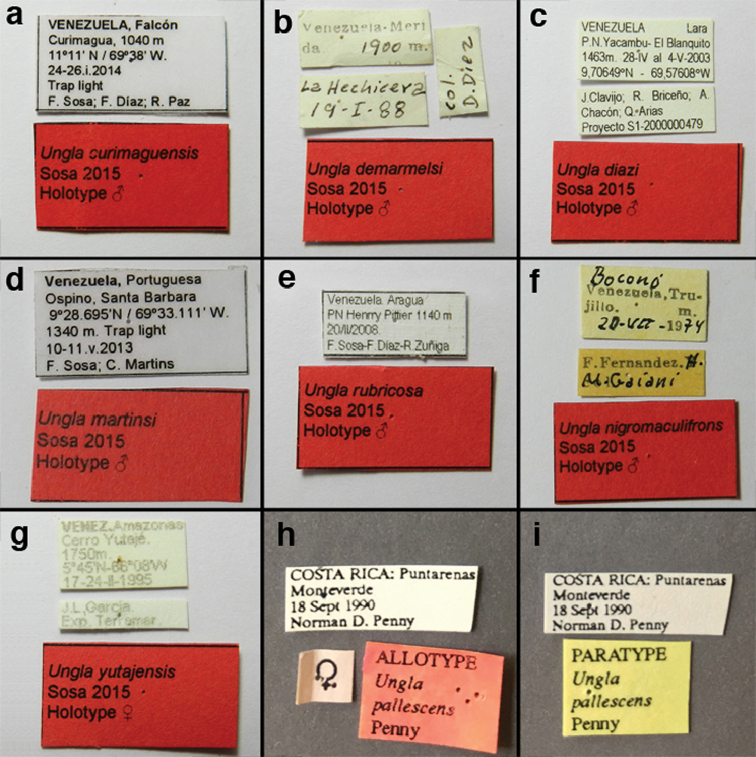
Type labels from *Ungla* species described in recent years before this study, **a–g** Sosa (2015) **h, i** Penny (1998).

**Figure 144.**
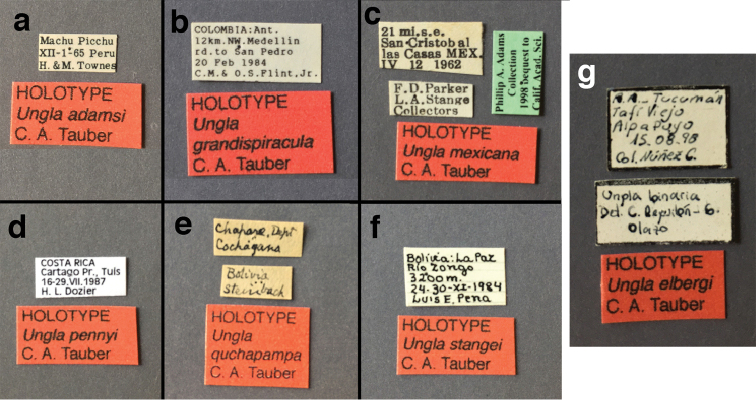
Type labels from *Ungla* species described in this study, **a–f** species from northern South America (**g**) species from southern South America.

## Supplementary Material

XML Treatment for Ungla adamsi

XML Treatment for Ungla banksi

XML Treatment for Ungla bolivari

XML Treatment for Ungla curimaguensis

XML Treatment for Ungla demarmelsi

XML Treatment for Ungla diazi

XML Treatment for Ungla favrei

XML Treatment for Ungla grandispiracula

XML Treatment for Ungla laufferi

XML Treatment for Ungla martinsi

XML Treatment for Ungla mexicana

XML Treatment for Ungla nigromaculifrons

XML Treatment for Ungla pallescens

XML Treatment for Ungla pennyi

XML Treatment for Ungla quchapampa

XML Treatment for Ungla siderocephala

XML Treatment for Ungla stangei

XML Treatment for Ungla yutajensis

XML Treatment for Ungla annulata

XML Treatment for Ungla argentina

XML Treatment for Ungla chacranella

XML Treatment for Ungla confraterna

XML Treatment for Ungla elbergi

XML Treatment for Ungla ivancruzi

XML Treatment for Ungla steinbachi
