# Corrigenda: Revision of the Neotropical green lacewing genus *Ungla* (Neuroptera, Chrysopidae). ZooKeys 674: 1–188. https://doi.org/10.3897/zookeys.674.11435

**DOI:** 10.3897/zookeys.691.19819

**Published:** 2017-08-17

**Authors:** Catherine A. Tauber, Francisco Sosa, Gilberto S. Albuquerque, Maurice J. Tauber

**Affiliations:** 1 Department of Entomology, Comstock Hall, Cornell University, Ithaca, NY 14853-2601 and Department of Entomology, University of California, Davis, CA, USA 95616; 2 Museo Entomológico “Dr. José Manuel Osorio” (MJMO), Universidad Centroccidental “Lisandro Alvarado”, Venezuela; 3 Laboratório de Entomologia e Fitopatologia, CCTA, Universidade Estadual do Norte Fluminense, Campos dos Goytacazes, RJ, Brazil 28013-602

After the publication of our article, we noted that we had neglected to list one of two specimens that we had examined during the description of the new species *Ungla mexicana* Tauber. In addition, in the figure captions, we reported the location of the holotype depository in error. Corrections are as follows:

Page 82, line 6: The paragraph under “Holotype” should read:

“We examined only two female specimens of this species, and we were reluctant to describe it as new on the basis of such limited material. However, the specimens are very well preserved, and the external features (head and body coloration and markings, wings) are notable. The abdomen of the holotype is cleared, stained, and in a vial attached to the specimen. Because of the species’ importance as the northernmost record for the genus, we describe it to facilitate future identifications.”

Page 83, Fig. 61 caption, last line: change CAS to FSCA.

Page 84, Fig. 62 caption, last line: change CAS to FSCA.

Page 85, Fig. 63 caption, last line: change CAS to FSCA.

Page 85, last line should read: “Specimens examined (in addition to holotype). Mexico. Veracruz: Cd. Mendoza, 3.xii.1975, C. M. Flint & O. S. Flint, Jr. (1F, paratype, USNM).”

